# Decarbonylation Products of Binuclear Methylphosphinidene Complexes of Cyclopentadienyliron Carbonyls: Triplet and Quintet Structures Are Favored Energetically over Singlet Structures with Iron–Iron Multiple Bonding

**DOI:** 10.1021/acsomega.3c10460

**Published:** 2024-02-28

**Authors:** Oleg Rudenco, Alexandru Lupan, Radu Silaghi-Dumitrescu, R. Bruce King

**Affiliations:** † Faculty of Chemistry and Chemical Engineering, Babeş-Bolyai University, Cluj-Napoca 400347, Romania; ‡ Department of Chemistry, University of Georgia, Athens, Georgia 30602, United States

## Abstract

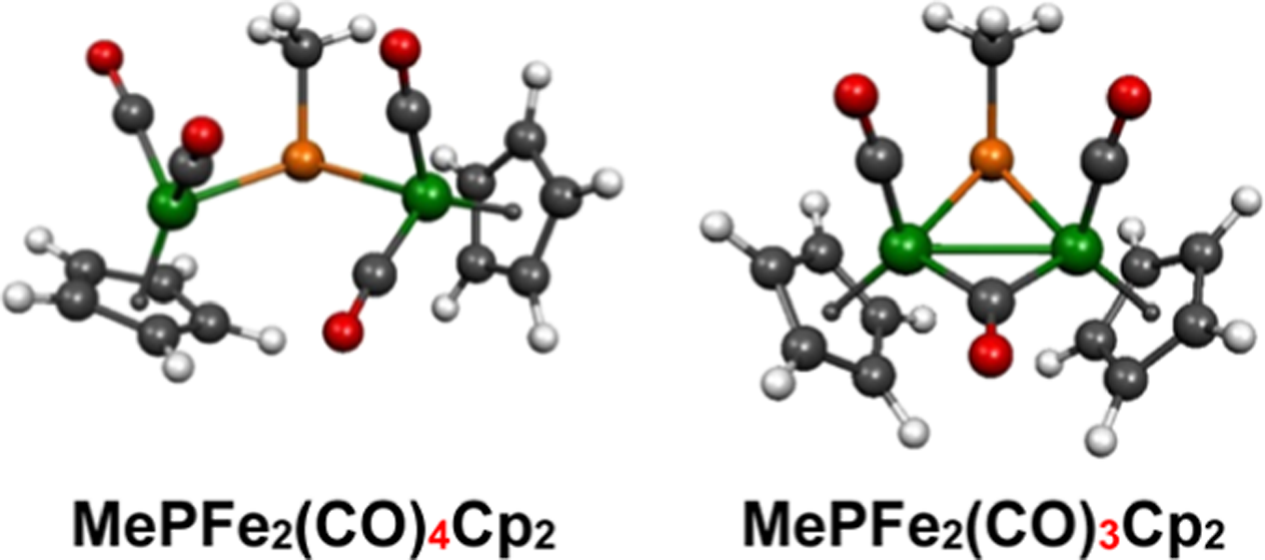

The structures, energetics, and energetically preferred spin states of methylphosphinidene-bridged binuclear cyclopentadienyliron carbonyl complexes MePFe_2_(CO)_*n*_Cp_2_ (*n* = 4, 3, 2, and 1) related to the experimentally known (μ-RP)Fe_2_(μ-CO)(CO)_2_Cp_2_ (R = cyclohexyl, phenyl, mesityl, and 2,4,6-tBu_3_C_6_H_2_) complexes have been investigated by density functional theory. Singlet structures having a pyramidal pseudotetrahedral phosphorus environment with 18-electron iron configurations are energetically preferred in the tricarbonyl and tetracarbonyl systems MePFe_2_(CO)_*n*_Cp_2_ (*n* = 4 and 3) with the lowest energy structures of the tricarbonyl very closely resembling the experimentally determined structures. For the more unsaturated dicarbonyl and monocarbonyl systems MePFe_2_(CO)_*n*_Cp_2_ (*n* = 2 and 1), higher spin state triplet and quintet structures are energetically preferred over singlet structures. These more highly unsaturated structures can be derived from the lowest energy singlet MePFe_2_(CO)_*n*_Cp_2_ (*n* = 4, 3) by the removal of carbonyl groups. The iron atoms giving up carbonyl groups in their 16- and 14-electron configurations bear the spin density of the unpaired electrons in the higher spin states. The lowest energy singlet structure of the monocarbonyl MePFe_2_(CO)Cp_2_, although a relatively high energy isomer, is unusual among the collection of MePFe_2_(CO)_*n*_Cp_2_ (*n* = 4, 3, 2, and 1) structures by having both the formal Fe=Fe double bond and the four-electron donor MeP unit with the planar phosphorus coordination required to allow each of its iron atoms to attain the favored 18-electron configuration.

## 1 Introduction

**Figure 1:**
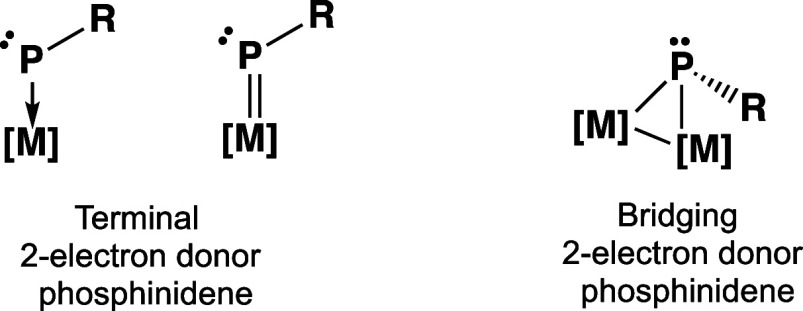
Alkyl- and arylphosphinidene ligands are two-electron donors.

Alkyl- and arylphosphinidenes, although not generally isolable as stable species, are versatile ligands in transition metal chemistry in complexes synthesized by indirect methods using related organophosphorus precursors.^1^ They can function as two-electron donor terminal or bridging ligands without the involvement of the phosphorus lone pair electrons (Figure 1).^2^ As two-electron donors, such phosphinidene ligands bear a relationship to the ubiquitous carbonyl ligand. Terminal two-electron donor phosphinidene ligands have a formal pseudotrigonal sp^2^-hybridized phosphorus atom with the nonbonding lone pair occupying one of the coordination positions and with a bent C–P–Fe angle. The phosphorus atom can act as an effective two-electron donor in such terminal phosphinidene metal complexes either by forming a P → M dative bond or by providing two of the four electrons for a formal double bond with the metal atom having σ and π components similar to the C=C double bond in ethylene. Bridging two-electron donor phosphinidene ligands have a formal trigonal pyramidal sp^3^-hybridized phosphorus atom with the nonbonding lone pair at the apex of the trigonal pyramid. The phosphorus atom in such bridging two-electron donor phosphinidene ligands bridges the pair of metal atoms by forming a formal single bond to each metal atom.

**Figure 2:**
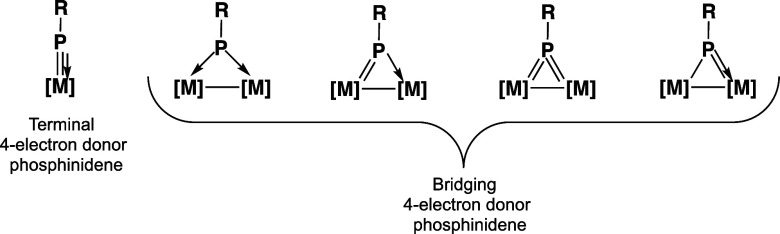
Alkyl- and arylphosphinidene ligands as four-electron donors with the participation of the phosphorus lone pair electrons in the ligand–metal bonding.

Alternative modes of bonding of phosphinidene ligands to metal atoms involve the participation of the phosphorus lone pair in the ligand–metal bonding, so the phosphinidene ligand becomes a formal four-electron donor (Figure 2). Terminal four-electron donor phosphinidene metal complexes have linear C–M–P geometry and a formal dative P≡M triple bond with sp hybridization of the phosphorus atom similar to the linear sp hybridization of alkyne carbon atoms. The phosphorus atoms in bridging four-electron donor phosphinidene ligands can form a P → M dative bond and/or a P=M double bond with each metal atom.

**Figure 3:**
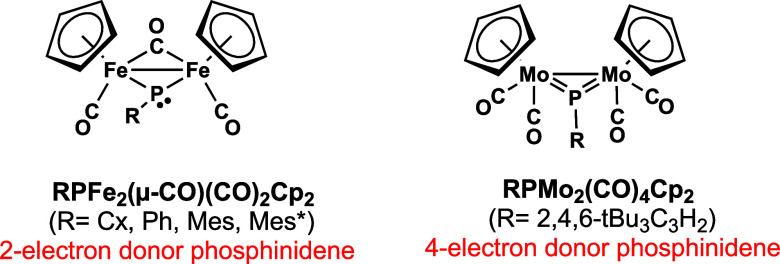
Experimentally known binuclear cyclopentadienyl metal carbonyls with bridging alkyl- and arylphosphinidene ligands.

Binuclear cyclopentadienyl metal carbonyl chemistry provides examples of experimentally characterized molecules stable under normal conditions having bridging phosphinidene ligands as either two-electron or four-electron donors (Figure 3). Thus, the iron complexes (μ-RP)Fe_2_(μ-CO)(CO)_2_Cp_2_ (R = cyclohexyl, phenyl, mesityl, and 2,4,6-tBu_3_C_6_H_2_) have a two-electron donor bridging phosphinidene ligand with clearly pyramidal phosphorus atoms.^3^ The molybdenum complex (μ-2,4,6-tBu_3_C_6_H_2_P)Mo_2_(CO)_4_Cp_2_ is an example of a stable binuclear cyclopentadienyl metal carbonyl derivative with a bridging four-electron donor arylphosphinidene ligand.^4^ In both the iron and molybdenum complexes, the central metal atoms have the favored 18-electron configuration. Bulky substituents on the phosphinidene phosphorus are favored in the chemistry of stable phosphinidene metal complexes in terms of both accessibility of suitable organophosphorus precursors and the stability of the final phosphinidene complexes. Furthermore, stable phosphinidene metal complexes such as the iron derivatives (μ-RP)Fe_2_(μ-CO)(CO)_3_Cp_2_ undergo a variety of reactions with various substrates to give a variety of related iron derivatives with other types of organophosphorus ligands.^5−10^

**Figure 4:**
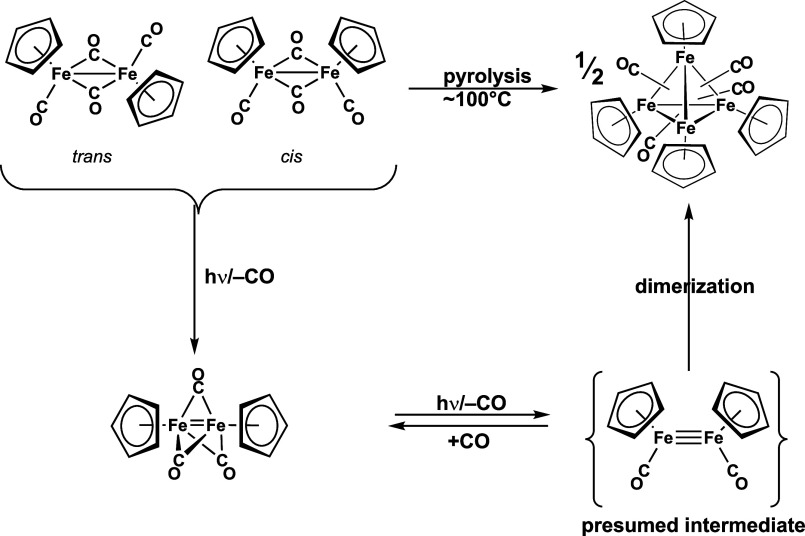
Formation of Cp_2_Fe_2_(μ-CO)_3_ and Cp_4_Fe_4_(μ_3_-CO)_4_ as stable products from the decarbonylation of Cp_2_Fe_2_(μ-CO)_2_(CO)_2_.

The iron complexes (μ-RP)Fe_2_(μ-CO)(CO)_2_Cp_2_ are related to the well-known, readily available, and extensively studied binuclear cyclopentadienyliron carbonyl derivative^11−13^ Cp_2_Fe_2_(μ-CO)_2_(CO)_2_ by replacement of one of its bridging carbonyl groups with a bridging two-electron donor phosphinidene ligand. The chemistry of decarbonylation of Cp_2_Fe_2_(μ-CO)_2_(CO)_2_ has been studied extensively (Figure 4). Photolysis of Cp_2_Fe_2_(μ-CO)_2_(CO)_2_ gives the tricarbonyl Cp_2_Fe_2_(μ-CO)_3_ which is an unusual example of a stable triplet state metal carbonyl derivative with a formal metal=metal double bond.^14−16^ The pyrolysis of Cp_2_Fe_2_(μ-CO)_2_(CO)_2_ gives the very stable tetranuclear complex Cp_4_Fe_4_(μ_3_-CO)_4_ in which each face of a central Fe_4_ tetrahedron is bridged by a carbonyl group.^17^ The tetranuclear Cp_4_Fe_4_(μ_3_-CO)_4_ is at least formally a dimerization product of an intermediate Cp_2_Fe_2_(CO)_2_ with a formal Fe≡Fe triple bond.^18,19^ These experimental results are supported and supplemented by theoretical studies on the Cp_2_Fe_2_(CO)_*n*_ (*n* = 4, 3, 2, and 1) systems using density functional methods.^20^

**Figure 5:**
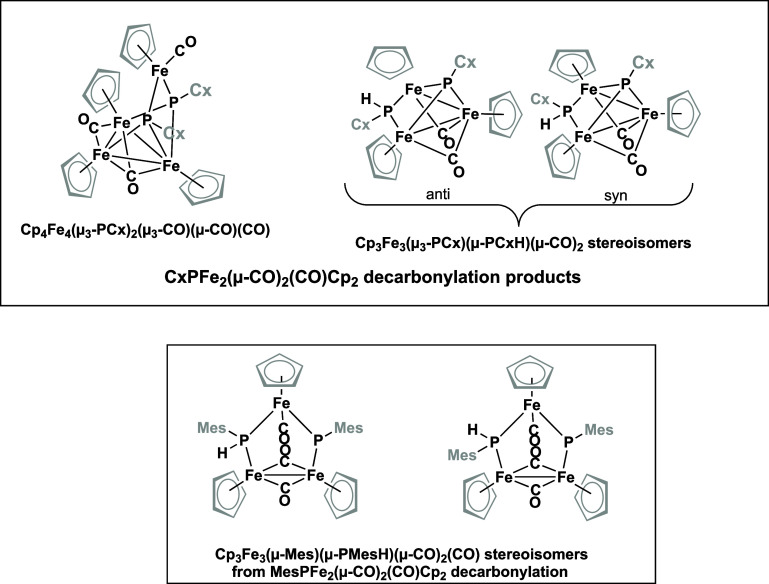
Stable trinuclear and tetranuclear products isolated from the decarbonylation of CxPFe_2_(μ-CO)(CO)_2_ and MesPFe_2_(μ-CO)(CO)_2_.

Experimental studies show the decarbonylation of the binuclear phosphinidene cyclopentadienyliron carbonyls to follow completely different pathways with significant variations depending on the alkyl or aryl group attached to the phosphinidene phosphorus (Figure 5).^21^ Trinuclear and tetranuclear products are found in which upon decarbonylation, the electrons of the nonbonding phosphorus lone pairs in the RPFe_2_(μ-CO)(CO)_2_Cp_2_ precursor become involved in the ligand–iron bonding in the trinuclear and/or tetranuclear products. In order to provide some understanding of such complicated decarbonylation reactions we have investigated the decarbonylation products of the simplest reasonable example of an RPFe_2_(μ-CO)(CO)_2_Cp_2_ complex, namely, the methyl derivative (R = CH_3_). For the formally unsaturated species RPFe_2_(CO)_*n*_Cp_2_ (*n* = 2 and 1) we find triplet and even quintet structures to be energetically favored relative to structures having iron–iron double and triple bonds analogous to the stable Cp_2_Fe_2_(μ-CO)_3_ and the presumed Cp_2_Fe_2_(CO)_2_ intermediate in the formation of Cp_4_Fe_4_(μ_3_-CO)_4_. The appreciable spin density on the iron atoms in such low-energy triplet and quintet RPFe_2_(CO)_*n*_Cp_2_ (*n* = 2 and 1) structures provide reactive sites for the formation of new iron–iron bonds leading ultimately to trinuclear and tetranuclear structures such as those in Figure 5. In addition, the observation of low-energy triplet and quintet structures in the RPFe_2_(CO)_*n*_Cp_2_ (*n* = 2, 1) systems may be relevant for the design of novel magnetic materials.

## 2 Theoretical Methods

The initial MePFe_2_(CO)_*n*_Cp_2_ chemical structures studied in this work have been designed by considering an MePFe_2_Cp_2_ unit followed by systematic placement of carbonyl groups as terminal and/or bridging ligands (coordinating to the metal ions through the carbon as well as both the carbon and oxygen atoms). Various Cp–Fe–CO orientations were also considered. This led to 195 different MePFe_2_(CO)_*n*_Cp_2_ (*n* = 1 to 4) starting structures, computed as singlets, triplets, and quintets.

Full geometry optimizations were performed by using the PBE0 DFT functional^22,23^ and the 6-31G* basis set as implemented in the Gaussian 09 software package.^24^ The lowest energy structures were then reoptimized at the PBE1PBE/def2-TZVP level of theory^25^ applying an ultrafine integration grid and tight convergence criteria and these are the structures presented and discussed. The energies include the zero-point and thermal corrections at 273 K. The nature of the stationary points was characterized by their harmonic vibrational frequencies. Saddle point structures with imaginary vibrational frequencies were reoptimized following the normal modes to ensure that genuine minima were obtained. The ν(CO) frequency values reported in the paper are scaled with a factor of 0.96. All of the lowest-energy structures have substantial HOMO–LUMO gaps ranging from 3.08 to 4.74 eV (Table S8 in the Supporting Information).

Only the lowest energy and thus potentially chemically significant structures are presented in detail in this paper. A larger number of structures of higher energy are presented in the Supporting Information. The optimized structures are designated as **Fe2PCOn-mX**, where **n** designates the number of carbonyl groups, **m** designates the energy ordering in terms of relative energy as compared to the global minimum of each family, and **X** designates the spin state as **S** (singlet), **T** (triplet), or **Q** (quintet).

## 3 Results and Discussion

### 3.1 Structures of the Tetracarbonyl MePFe_2_(CO)_4_Cp_2_

**Figure 6:**
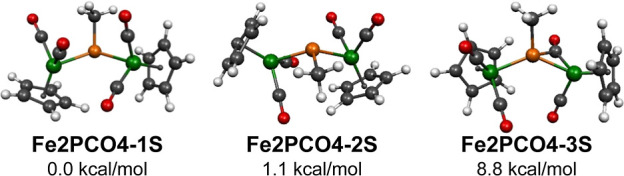
Three lowest energy MePFe_2_(CO)_4_Cp_2_ structures.

Three structures were found for the tetracarbonyl MePFe_2_(CO)_4_Cp_2_ within 16 kcal/mol of the lowest energy structures (Figure 6 and Tables 1 and 2). The two lowest energy such structures, namely, **Fe2PCO4-1S** and **Fe2PCO4-2S**, are very similar and lie within ∼1 kcal/mol of each other. The long Fe···Fe distances of ∼4.07 Å in each of the two structures clearly indicate the lack of direct iron–iron bonds. In each of the two structures, the MeP phosphinidene unit bridges two CpFe(CO)_2_ units with Fe–P distances of ∼2.36 Å. The phosphorus atom lies ∼0.7 Å above the Fe_2_C(methyl) plane (with an average of 110° for the bond angles around the P), indicating pseudotetrahedral coordination with a stereochemically active lone pair. This may be taken to imply that the MeP unit donates one electron to each of the iron atoms thereby giving them the favored 18-electron configuration. The infrared spectra of both **Fe2PCO4-1S** and **Fe2PCO4-2S** are predicted to exhibit four ν(CO) frequencies in the 2070–2123 cm^–1^ range clearly indicative of four terminal carbonyl groups in each structure (Table 2).

**Table 1:** Three MePFe_2_(CO)_4_Cp_2_ Structures Lying within 16 kcal/mol of the Lowest Energy Structure

structure (symmetry)		Fe–Fe interaction		Fe–P distances	P distance (Å) of Fe_2_C plane
	Δ*E*	distance	WBI		
**Fe2PCO4-1S** (*C*_1_)	0.0	4.074	0.02	2.35, 2.37	0.71
**Fe2PCO4-2S** (*C*_1_)	1.1	4.089	0.02	2.36, 2.36	0.73
**Fe2PCO4-3S** (*C*_1_)	8.8	4.133	0.05	2.29, 2.24	0.04

**Table 2:** Harmonic ν(CO) Frequencies of the Three MePFe_2_(CO)_4_Cp_2_ Structures (Scaled Values in cm^–1^ and IR Intensities in km·mol^–1^ in Parentheses) with the Bridging ν(CO) Frequency in Italics

structure	ν(CO) frequencies, cm^–1^
**Fe2PCO4-1S**	1987(511), 1991(414), 2014(574), 2040(1075)
**Fe2PCO4-2S**	1976(387), 2000(548), 2014(1029), 2038(581)
**Fe2PCO4-3S**	*1737*(*534*), 1969(564), 2020(817), 2057(643)

The third MePFe_2_(CO)_4_Cp_2_ structure, **Fe2PCO4-3S**, lying 8.8 kcal/mol in energy above **Fe2PCO4-1S**, has the interesting feature of a carbonyl group bridging an Fe–P bond (Figure 6 and Tables 1 and 2). This bridging carbonyl group is predicted to exhibit a very low ν(CO) frequency of 1737 cm^–1^ which is more than 200 cm^–1^ below the three predicted terminal ν(CO) frequencies at 1969, 2020, and 2057 cm^–1^. The phosphorus coordination in **Fe2PCO4-3S** can be interpreted as distorted pseudotrigonal bipyramidal with the bridging carbonyl group and stereochemically active lone pair in the axial positions. This leaves the methyl carbon and the two iron atoms in the equatorial plane with the central phosphorus atom lying only ∼0.04 Å above the equatorial plane. Moving one of the carbonyl groups from a terminal position in **Fe2PCO4-1S** and **Fe2PCO4-2S** to bridging an Fe–P bond in **Fe2PCO4-3S** while retaining the stereochemically active nonbonding phosphorus lone pair does not affect the net donation of two electrons from the MeP bridge to the iron atoms. Therefore, each iron atom in **Fe2PCO4-3S** retains the favorable 18-electron configuration of the iron atoms in **Fe2PCO4-1S** and **Fe2PCO4-2S**.

**Figure 7:**
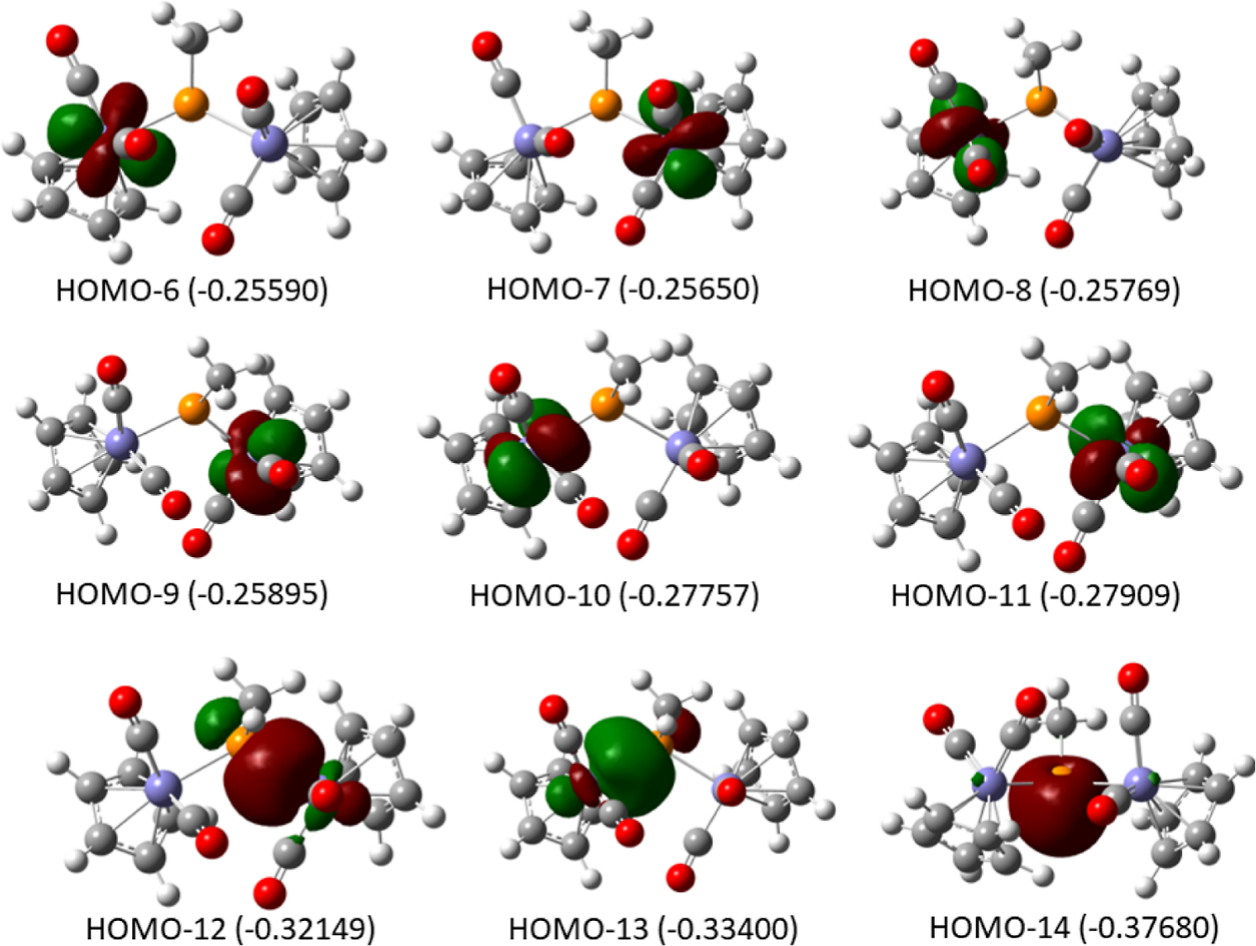
Frontier molecular orbitals with a dominant Fe contribution for **Fe2PCO4-1S**.

In the MePFe_2_(CO)_*n*_Cp_2_ complexes, the two formally anionic Cp ligands imply a di-Fe(I) structure—hence with two paramagnetic centers. However, no unpaired electrons are found in the population analysis of the structures shown in Figure 6. The overall singlet state may then be achieved in one of four manners. First, Fe–Fe covalence is ruled out by the Wiberg bond orders in Table 1. Second, antiferromagnetic coupling is ruled out by the absence of unpaired electrons in the Mulliken or NBO population analysis (not shown) in any of the structures in Figure 6. Moreover, the triplet state of **Fe2PCO4-1S** is found to lie ∼35 kcal/mol above the singlet (much too high for a center involved in antiferromagnetic coupling), and it features one of the unpaired electrons on the phosphorus, rather than an electron on each iron (also rendering a complete broken symmetry calculation impractical). The third explanation would involve quantum admixture of the singly occupied iron orbitals, yielding an apparent diamagnetic state. The frontier molecular orbitals for **Fe2PCO4-1S**, shown in Figure 7, may be consistent with this explanation. The fourth possible explanation would entail covalent bonding between each of the two Fe(I) centers and a triplet phosphinidene. Orbitals HOMO–12 and HOMO–13 in Figure 7 indeed illustrate the two Fe–P σ bonds—with a clear covalent character (59% on Fe, cf. NBO analysis). Owing mostly to the 59% character on Fe, the NBO partial atomic charges on Fe are negative (−0.8 each). This would imply unlikely Fe^–^ character and thus offers an instructive caveat on interpreting gross atomic populations from such analyses. The above-discussed pseudotetrahedral sp^3^-type geometry around the phosphorus (where the fourth vertex is the phosphorus lone pair, orbital HOMO–14 in Figure 7) and the Mulliken partial atomic charge on phosphorus of −0.04 are consistent with the two-electron donor description depicted in Figure 1 for the bridging phosphinidene. On the other hand, the strong covalence of the two single Fe–P bonds makes it difficult to assign the origin of the four electrons in these two bonds. Thus, a pseudotetrahedral geometry could be attained in any of the four scenarios: two Fe(II) coordinated by CH_3_P^2–^, two Fe(I) bound to singlet or to triplet CH_3_P, or di-Fe(0) coordinating to CH_3_P^2+^—nor would this strong Fe–P covalence allow a resolution via multiconfigurational calculations such as CASSCF. Calculations of this type would also be impractical owing to the size of the active space required in order to include all relevant Fe- and P-centered molecular orbitals.

### 3.2 Structures of the Tricarbonyl MePFe_2_(CO)_3_Cp_2_

**Figure 8:**
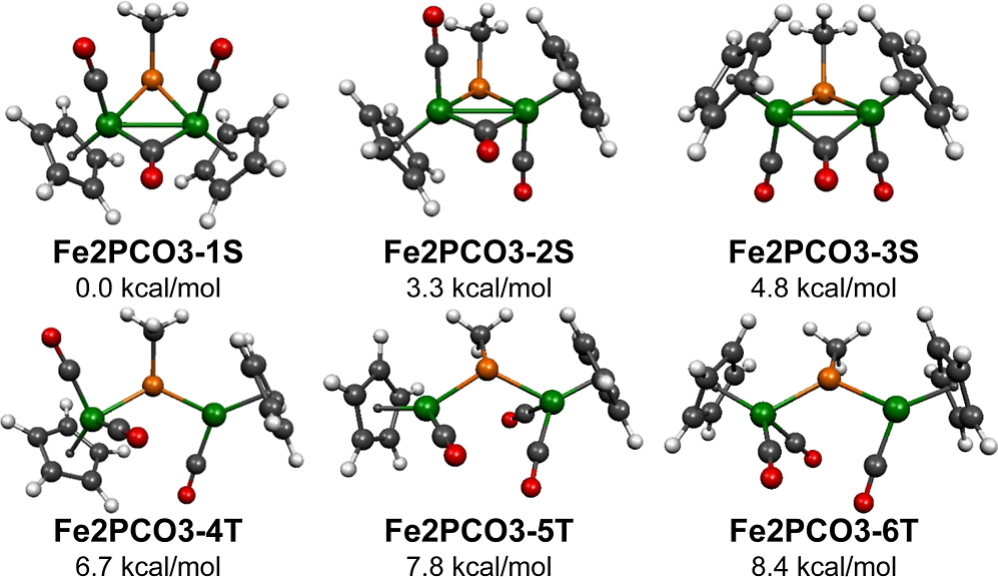
Six lowest energy MePFe_2_(CO)_3_Cp_2_ structures.

Six MePFe_2_(CO)_3_Cp_2_ structures were found within 16 kcal/mol of the lowest energy structure (Figure 8 and Tables 3 and 4). The three lowest energy structures **Fe2PCO3-1S**, **Fe2PCO3-2S**, and **Fe2PCO3-3S** are singlet structures with each structure having a bridging MeP unit with Fe–P distances of ∼2.25 Å, a bridging carbonyl group with Fe–C distances of ∼1.91 Å, and an iron–iron distance of ∼2.58 Å with a WBI of 0.3 suggesting a formal single bond. These interatomic distances are within 0.01 Å of the experimental Fe–P distances of 2.26 Å, Fe–C distances of 1.92 Å to the bridging carbonyl group, and an Fe–Fe distance of 2.59 Å found by X-ray crystallography^3^ in the phenyl derivative PhPFe_2_(CO)_3_Cp_2_. The bridging carbonyl group is predicted to exhibit an ν(CO) frequency of ∼1830 cm^–1^ in contrast to the two terminal ν(CO) frequencies ranging from 1992 to 2037 cm^–1^. The phosphorus atom lies ∼1.0 Å above the Fe_2_C plane, indicating pseudotetrahedral phosphorus coordination with a stereochemically active lone pair. Thus, the MeP bridge donates a single electron to each iron atom in each of these structures thereby giving each iron atom the favored 18-electron configuration after considering the iron–iron single bond.

**Table 3:** Six MePFe_2_(CO)_3_Cp_2_ Structures Are within 16 kcal/mol of the Lowest Energy Structure

structure (symmetry)		Fe–Fe interaction		Fe–P distances	spin densities		P distance (Å) out of Fe_2_C plane
	Δ*E*	distance	WBI		2Fe1	P	
**Fe2PCO3-1S** (*C*_s_)	0.0	2.581	0.30	2.24, 2.25			0.91
**Fe2PCO3-2S** (*C*_1_)	3.3	2.591	0.30	2.25, 2.26			0.97
**Fe2PCO3-3S** (*C*_s_)	4.8	2.588	0.31	2.26, 2.26			1.01
**Fe2PCO3-4T** (*C*_1_)	6.7	3.942	0.03	2.23, 2.33	0.08, 2.18	–0.06	0.62
**Fe2PCO3-5T** (*C*_1_)	7.8	3.976	0.03	2.27, 2.34	0.28, 2.12	–0.07	0.62
**Fe2PCO3-6T** (*C*_1_)	8.4	4.093	0.03	2.22, 2.32	0.04, 2.08	0.05	0.55

**Table 4:** Harmonic ν(CO) Frequencies of the Six MePFe_2_(CO)_3_Cp_2_ Structures (Scaled Values in cm^–1^ and IR Intensities in km·mol^–1^ in Parentheses) with the Bridging ν(CO) Frequency in Italics

structure	ν(CO) frequencies, cm^–1^
**Fe2PCO3-1S**	*1833*(*580*), 1992(214), 2021(1220)
**Fe2PCO3-2S**	*1830*(*600*), 1992(1032), 2005(315)
**Fe2PCO3-3S**	*1828*(*595*), 2007(177), 2037(1316)
**Fe2PCO3-4T**	1970(752), 1996(752), 2028(633)
**Fe2PCO3-5T**	1985(58), 2002(1121), 2038(762)
**Fe2PCO3-6T**	1972(356), 2005(703), 2043(824)

The three MePFe_2_(CO)_3_Cp_2_ structures **Fe2PCO3-1S**, **Fe2PCO3-2S**, and **Fe2PCO3-3S**, which lie within 5 kcal/mol of each other, differ only in the positions of the Cp rings and terminal carbonyl groups relative to the central MePFe_2_(μ-CO) unit (Figure 8 and Tables 3 and 4). The lowest energy structure **Fe2PCO3-1S** is a cis structure, with both Cp rings on opposite sides of the MeP bridge. Structure **Fe2PCO3-2S**, lying 3.3 kcal/mol in energy above **Fe2PCO3-1S**, is a trans structure with one Cp ring on the same side as the MeP unit and the other Cp ring on the opposite side of the MeP unit. Finally, the highest energy of these three singlet closely related structures, namely, **Fe2PCO3-3S**, lying 4.8 kcal/mol in energy above **Fe2PCO3-1S**, is another cis structure with both Cp rings on the same side as the MeP bridge. Thus, the relative energies of the three singlet MePFe_2_(CO)_3_Cp_2_ isomers appear related to steric repulsion between the Cp rings and the MeP bridge with Cp rings on the same side of the bridge leading to slightly higher energies.

**Figure 9:**
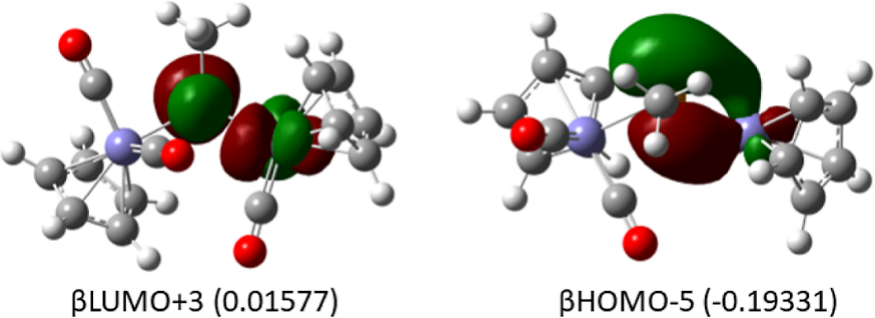
Frontier molecular orbitals in **Fe2PCO3-4T** illustrating Fe–P π bonding; the remaining Fe- and P-localized orbitals (not shown) are similar to those in Figure 7, with due differences to the spin states identified in Table 3, i.e., an S = 0 d^6^ Fe and an S = 1 d^6^ Fe, each engaged in one additional covalent σ bond with phosphorus consistent with a di-Fe(I) center and a four-electron phosphorus donor.

The next three MePFe_2_(CO)_3_Cp_2_ structures, namely, **Fe2P(CO)3-4T**, **Fe2P(CO)3-5T**, and **Fe2P(CO)3-6T**, are closely related triplet structures closely spaced in energy at 6.7, 7.8, and 8.4 kcal/mol above **Fe2P(CO)3-1S** (Figure 8 and Tables 3 and 4). These structures can be derived from the lowest energy MePFe_2_(CO)_4_Cp_2_ structures by the removal of a carbonyl group from one of the iron atoms, giving it only a 16-electron configuration. The spin density corresponding to the two unpaired electrons of the triplet spin state resides on this iron atom bearing only a single carbonyl group and thus indicating a high-spin 16-electron configuration. The frontier molecular orbitals for **Fe2P(CO)3-4T** are consistent with this general picture except for the suggestion of π-bonding between the phosphorus atom and the iron atom bearing a single carbonyl group in βHOMO–5 (Figure 9).

### 3.3 Structures of the Dicarbonyl MePFe_2_(CO)_2_Cp_2_

**Figure 10:**
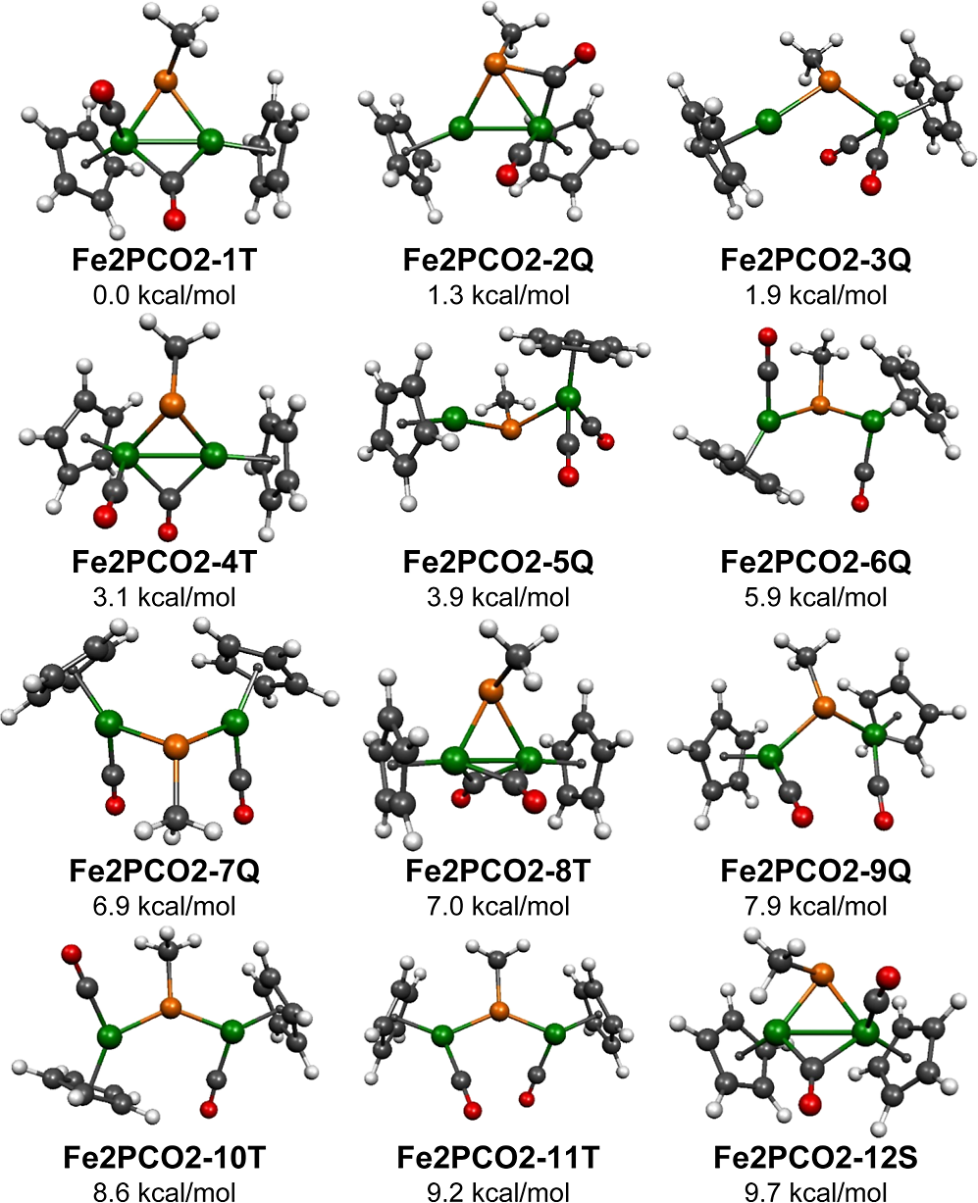
12 lowest energy MePFe_2_(CO)_2_Cp_2_ structures.

The potential energy surface for the dicarbonyl MePFe_2_(CO)_2_Cp_2_ was found to be the most complicated of the four MePFe_2_(CO)_*n*_Cp_2_ (*n* = 4, 3, 2, and 1) systems investigated in this study with 12 structures lying within 10 kcal/mol of the lowest energy structure **Fe2PCO2-1T** (Figure 10 and Tables 5 and 6). Furthermore, the lowest energy singlet MePFe_2_(CO)_2_Cp_2_ structure **Fe2PCO2-12S** is predicted to have an energy higher than 11 lower energy triplet and quintet spin state structures. Most of these lower energy, higher spin MePFe_2_(CO)_2_Cp_2_ dicarbonyl structures can be derived from either one of the singlet MePFe_2_(CO)_4_Cp_2_ tetracarbonyl structures by the removal of two of the four carbonyl groups or from one of the singlet MePFe_2_(CO)_3_Cp_2_ tricarbonyl structures by the removal of one of the terminal carbonyl groups. Such a carbonyl group removal from iron atoms with 18-electron configurations in the original tetracarbonyl or tricarbonyl structure leads to iron atoms with high spin 16- and 14-electron configurations as sites of two or four unpaired electrons, respectively, as indicated by spin densities. Thus, the triplet lowest energy MePFe_2_(CO)_2_Cp_2_ structure **Fe2PCO2-1T** as well as the 3.1 kcal/mol higher energy triplet structure **Fe2PCO2-4T** can be derived from the MePFe_2_(CO)_3_Cp_2_ tricarbonyl structures **Fe2PCO3-1S** and **Fe2PCO3-3S**, respectively, by the loss of a single carbonyl group. The iron atoms losing the CO group acquire spin densities of ∼2.5, corresponding to the two unpaired electrons of the triplet spin state. Similarly the closely energetically spaced five quintet structures **Fe2PCO2-3Q**, **Fe2PCO2-5Q**, **Fe2PCO2-6Q**, **Fe2PCO2-7Q**, and **Fe2PCO2-9Q**, lying 1,9, 3.9, 5.9, 6.9, and 7.9 kcal/mol above **Fe2PCO2-1T**, are obtained by the removal of two carbonyl groups from the MePFe_2_(CO)_4_Cp_2_ structures **Fe2PCO4-1S** and **Fe2PCO4-2S**. In generating **Fe2PCO2-3Q** and **Fe2PCO2-5Q** from the MePFe_2_(CO)_4_Cp_2_ structures, both carbonyl groups are removed from the same iron atom, which in the quintet dicarbonyl structures bears a spin density of ∼3.8 corresponding to all four unpaired electrons of the quintet spin state. However, in generating **Fe2PCO2-6Q**, **Fe2PCO2-7Q**, and **Fe2PCO2-9Q** from the MePFe_2_(CO)_4_Cp_2_ structures, each iron atom loses one carbonyl group and thus bears a spin density of 2.25 corresponding to two of the four unpaired electrons of the quintet spin state. The MePFe_2_(CO)_2_Cp_2_ structure **Fe2PCO2-2Q**, lying 1.3 kcal/mol in energy above **Fe2PCO2-1T**, has a carbonyl group bridging an Fe–P bond predicted to exhibit a low ν(CO) frequency of 1798 cm^–1^ (Table 6). It can be derived from the MePFe_2_(CO)_4_Cp_2_ structure **Fe2PCO4-3S** (Figure 6) by loss of the two terminal carbonyl groups from the iron atom not bearing the bridging carbonyl group, leading to a spin density of 3.63 on this iron atom corresponding to the four unpaired electrons of the quintet spin state. In all nine of these lowest energy MePFe_2_(CO)_4_Cp_2_ structures from **Fe2PCO2-1T** to **FePCO2-9Q** generated by carbonyl loss from carbonyl-richer structures, the phosphorus retains a pseudotetrahedral geometry lying 0.75 to 1.09 Å out of the Fe_2_C plane with a stereochemically active lone pair. Thus, the bridging MeP ligands in these nine structures form P–Fe single bonds and thus donate only one electron to each iron atom.

**Table 5:** 12 MePFe_2_(CO)_2_Cp_2_ Structures within 10 kcal/mol of the Lowest Energy Structure

structure (symmetry)		Fe–Fe interaction		Fe–P distances	spin densities		P distance (Å) out of Fe_2_C plane
	Δ*E*	distance	WBI		2Fe	P	
**Fe2PCO2-1T** (*C*_1_)	0.0	2.511	0.29	2.25, 2.25	–0.23, 2.58	–0.25	1.04
**Fe2PCO2-2Q** (*C*_1_)	1.3	2.511	0.26	2.37, 2.41	0.23, 3.63	–0.24	1.04
**Fe2PCO2-3Q** (*C*_1_)	1.9	3.911	0.03	2.32, 2.34	0.07, 3.78	–0.04	0.76
**Fe2PCO2-4T** (*C*_1_)	3.1	2.529	0.28	2.25, 2.27	–0.16, 2.52	–0.27	1.04
**Fe2PCO2-5Q** (*C*_1_)	3.9	3.597	0.04	2.30, 2.36	0.44, 3.77	–0.01	0.83
**Fe2PCO2-6Q** (*C*_1_)	5.9	3.491	0.06	2.35, 2.36	2.20, 2.23	–0.07	0.75
**Fe2PCO2-7Q** (*C*_1_)	6.9	3.591	0.06	2.25, 2.26	2.18, 2.21	–0.08	0.68
**Fe2PCO2-8T** (*C*_1_)	7.0	2.260	0.42	2.25, 2.25	1.14, 1.15	–0.03	1.09
**Fe2PCO2-9Q** (*C*_1_)	7.9	3.551	0.06	2.25, 2.26	2.21, 2.24	–0.10	0.77
**Fe2PCO2-10T** (*C*_1_)	8.6	3.976	0.05	2.15, 2.19	0.57, 1.99	–0.36	0.10
**Fe2PCO2-11T** (*C*_2_)	9.2	3.989	0.10	2.15, 2.18	0.54, 2.01	–0.37	0.03
**Fe2PCO2-12S** (*C*_1_)	9.7	2.569	0.31	2.18, 2.23			1.28

**Table 6:** Harmonic ν(CO) Frequencies of the 12 MePFe_2_(CO)_2_Cp_2_ Structures (Scaled Values in cm^–1^ and IR Intensities in km·mol^–1^ in Parentheses) with the Bridging ν(CO) Frequency in Italics

structure	ν(CO) frequencies, cm–^1^
**Fe2PCO2-1T**	*1829*(*585*), 2009(807)
**Fe2PCO2-2Q**	*1798*(*819*), 1989(723)
**Fe2PCO2-3Q**	1987(680), 2017(469)
**Fe2PCO2-4T**	*1828*(*604*), 1934(826)
**Fe2PCO2-5Q**	2004(669), 1940(772)
**Fe2PCO2-6Q**	1989(1495), 1998(137)
**Fe2PCO2-7Q**	1982(362), 2017(1468)
**Fe2PCO2-8T**	1848(1043),1878(330)
**Fe2PCO2-9Q**	1987(180), 2033(1403)
**Fe2PCO2-10T**	1956(1433), 1998(896)
**Fe2PCO2-11T**	1959(533), 2018(1197)
**Fe2PCO2-12S**	*1827*(*576*), 1992(880)

The triplet structure **Fe2PCO2-8T** is the only one of the 12 lowest energy MePFe_2_(CO)_2_Cp_2_ structures in which both carbonyl groups are bridging carbonyl groups (Figure 10 and Tables 5 and 6). It can be considered to be an analogue of the experimentally known triplet structures (η^5^-R_5_C_5_)_2_Fe_2_(μ-CO)_3_ (R = H and Me)^14−16^ in which one of the bridging CO groups has been replaced by a pyramidal two-electron donor bridging MeP group. The predicted Fe=Fe distance of 2.26 Å with a WBI value of 0.42 clearly indicates a formal double bond. This Fe=Fe distance in **Fe2PCO2-8T** is essentially identical to the experimental Fe=Fe distance of 2.265 Å in (η^5^-Me_5_C_5_)_2_Fe_2_(μ-CO)_3_ as determined by X-ray crystallography.

The triplet MePFe_2_(CO)_2_Cp_2_ structures **Fe2PCO2-10T** and **Fe2PCO2-11T**, lying ∼9 kcal/mol in energy above **Fe2PCO2-1T**, represent a trans–cis isomer pair in which the phosphorus atom lies only 0.10 and 0.03 Å outside the Fe_2_C(methyl) plane (Figure 10 and Tables 5 and 6). This essential Fe_2_CP coplanarity suggests that the bridging MeP unit is a four-electron rather than the usual two-electron donor in these structures. Thus, the MeP bridge in **Fe2PCO2-10T** and **Fe2PCO2-11T** donates two electrons to each iron atom, which, when combined with two electrons from the terminal CO groups and five electrons from a neutral Cp ring, gives each iron atom a 17-electron configuration consistent with a binuclear triplet.

The lowest energy singlet MePFe_2_(CO)_2_Cp_2_ structure **Fe2PCO2-12S**, lying 9.7 kcal/mol in energy above **Fe2PCO2-1T**, has a bridging carbonyl group, a terminal carbonyl group, and a highly pyramidalized phosphorus atom lying 1.28 Å outside the Fe_2_C(methyl) plane (Figure 10 and Tables 5 and 6). This bridging MeP group in **Fe2PCO2-12S** is bent toward one of the iron atoms because of an agostic C–H–Fe interaction of one of the methyl hydrogen atoms with the iron atom as indicated by short Fe–H and Fe–C distances of 1.731 and 2.223 Å, respectively. Because of the two-electron donation of this agostic C–H–Fe interaction, the bridging MeP group in **Fe2CO2-12S** becomes effectively a four-electron donor, despite the lack of involvement of the lone-pair of the pyramidal phosphorus atom in the phosphorus–iron bonding. The Fe–Fe distance of ∼2.57 Å with a WBI of 0.31 is comparable to the iron–iron single bonds found in numerous other MePFe_2_(CO)_*n*_Cp_2_ (*n* = 3 and 2) structures. We therefore conclude that the Fe–Fe bond is only a single bond in **Fe2PCO2-12S**, thereby giving each iron the favored 18-electron configuration.

**Figure 11:**
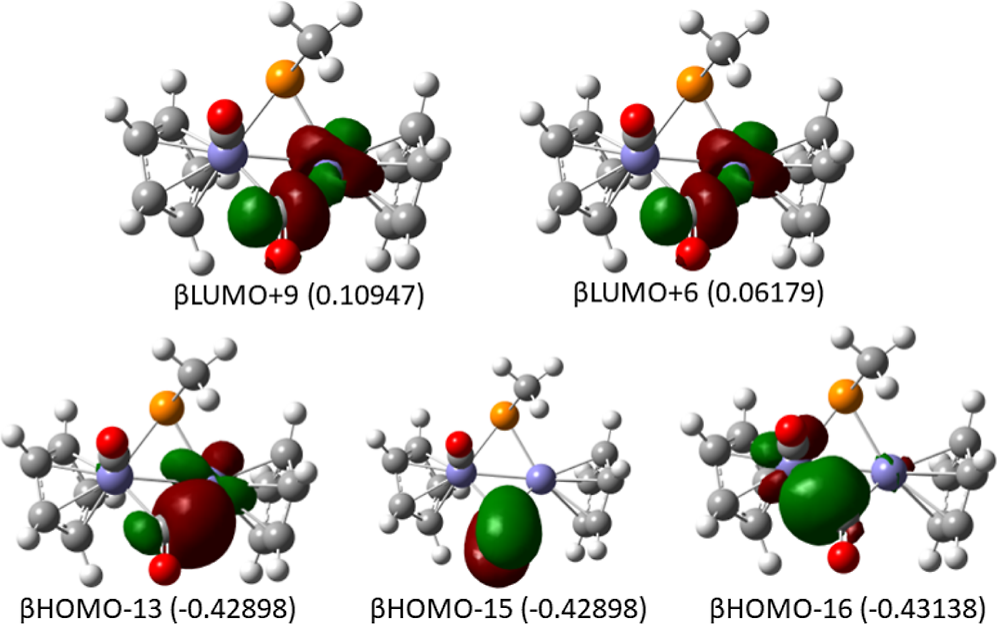
Frontier molecular orbitals in **Fe2PCO2-1T** illustrate σ donation from the bridging CO π orbital to each of the two iron atoms. Also shown in the second CO π orbital, which lies in the same energy region but does not mix with the iron orbitals. The remaining Fe- and P-localized orbitals (not shown) are similar to those in Figure 7, with due differences to the spin states identified in Table 3, i.e., a diamagnetic d^6^ Fe and an S = 4 d^6^ Fe, each engaged in one additional covalent σ bond with P and thus consistent with a di-Fe(I) center and a two-electron P donor.

The molecular orbitals of **Fe2PCO2-1T** are similar to those of the tetracarbonyl MePFe_2_(CO)_4_Cp_2_ illustrated in Figure 7, with one notable exception, illustrated in Figure 11. Namely, the bridging CO unit uses one of its π orbitals as a donor to each of the two iron atoms, thereby allowing supplementation of the electron counts beyond those rationalized above for this representative dicarbonyl complex.

### 3.4 Structures of the Monocarbonyl MePFe_2_(CO)Cp_2_

**Figure 12:**
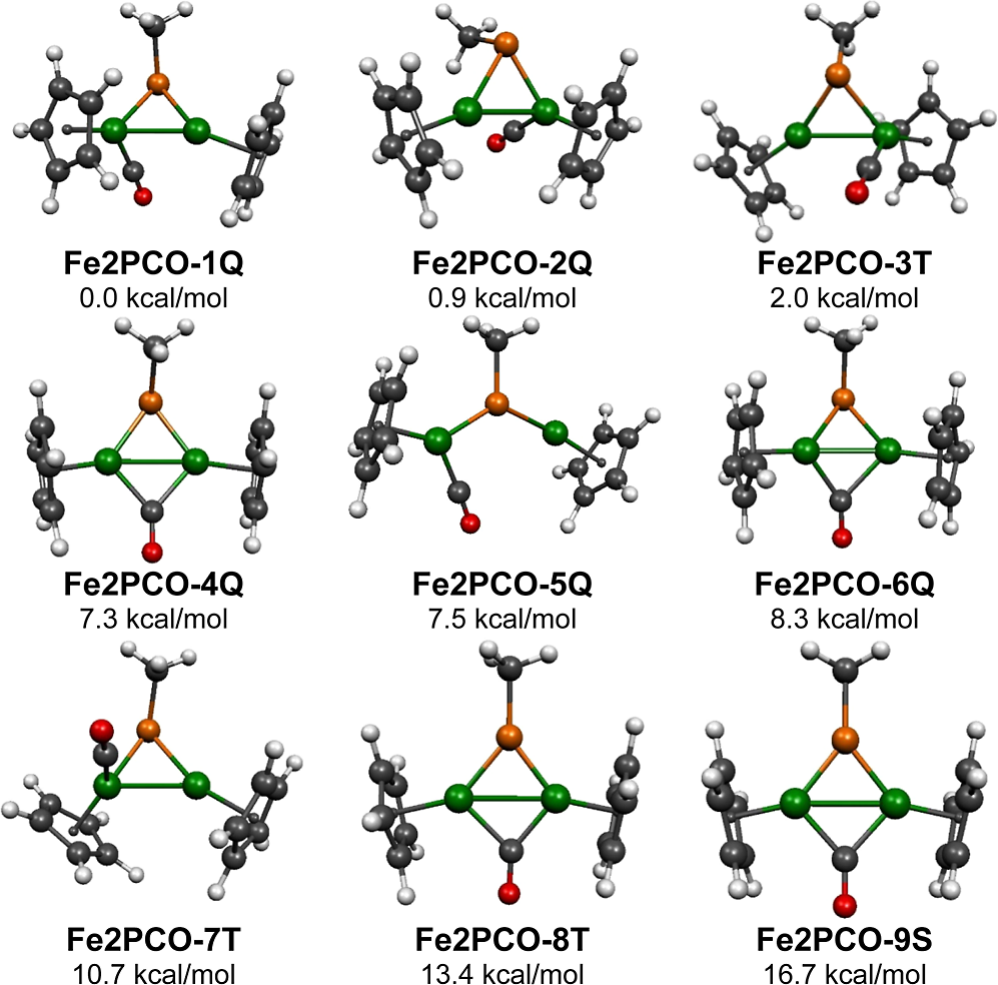
Nine lowest energy MePFe_2_(CO)Cp_2_ structures.

Nine structures for monocarbonyl MePFe_2_(CO)Cp_2_ were found within 17 kcal/mol of the lowest energy structure **Fe2PCO-1Q** (Figure 12 and Tables 7 and 8). The two lowest energy structures, namely, the quintet structures **Fe2PCO-1Q** and **Fe2PCO-2Q**, lying within 1 kcal/mol of energy, can be derived by removal of the bridging carbonyl group and one of the terminal carbonyl groups from the lowest energy MePFe_2_(CO)_3_Cp_2_ structures, leaving one of the iron atoms without any carbonyl groups bonded to it. This “carbonyl bare” iron atom has a spin density of ∼3.5 corresponding to the four unpaired electrons of the quintet spin state. The quintet **Fe2PCO-6Q** structure, lying 8.3 kcal/mol in energy above **Fe2PCO-1Q**, is also derived from the lowest energy MePFe_2_(CO)_3_Cp_2_ structures but by the removal of both terminal carbonyl groups, retaining the bridging carbonyl between two equivalent iron atoms sharing equally the spin density. The triplet structure **Fe2PCO-3T**, lying 2.0 kcal/mol above **Fe2PCO-1Q**, resembles **Fe2PCO-1Q** except for the spin state. All four of these MePFe_2_(CO)Cp_2_ structures have formal Fe–Fe single bonds of lengths around ∼2.5 Å, with WBI values around 0.3. In contrast, the quintet structure **Fe2PCO-5Q**, lying 7.5 kcal/mol in energy above **Fe2PCO-1Q**, is derived from the lowest energy structures of the tetracarbonyl MePFe_2_(CO)_3_Cp_2_ by the removal of three of the four carbonyl groups but leaving the pair of iron atoms at a nonbonding distance of 3.96 Å with a near-zero WBI of 0.04. The first four low-energy MePFe_2_(CO)Cp_2_ structures have pseudotetrahedrally coordinated phosphorus atoms lying 0.75 to 1.06 Å outside the Fe_2_C(methyl) plane.

**Table 7:** Nine MePFe_2_(CO)Cp_2_ Structures within 16 kcal/mol of the Lowest Energy Structure

structure (symmetry)		Fe–Fe interaction		Fe–P distances	spin densities		P distance (Å) out of Fe_2_C plane
	Δ*E*	distance	WBI		2Fe	P	
**Fe2PCO-1Q** (*C*_1_)	0.0	2.454	0.34	2.19.2/32	0.76,3.52	–0.29	1.01
**Fe2PCO-2Q** (*C*_1_)	0.9	2.432	0.35	2.21, 2.34	0.86,3.52	0.05	1.06
**Fe2PCO-3T** (*C*_1_)	2.0	2.614	0.25	2.26, 2.31	–1.90,3.65	–0.07	1.06
**Fe2PCO-4Q** (*C*_s_)	7.3	2.536	0.26	2.18, 2.18	2.53,2.53	–0.87	0.79
**Fe2PCO-5Q** (*C*_1_)	7.5	3.964	0.04	2.13, 2.28	0.51,3.74	–0.41	0.04
**Fe2PCO-6Q** (*C*_s_)	8.3	2.415	0.34	2.18, 2.25	1.93,2.11	–0.59	0.92
**Fe2PCO-7T** (*C*_1_)	10.7	2.553	0.32	2.13, 2.32	0.79,1.95	–0.62	0.75
**Fe2PCO-8T** (*C*_s_)	13.4	2.591	0.29	2.04, 2.12	0.04,2.25	–0.17	0.03
**Fe2PCO-9S** (*C*_2v_)	16.7	2.533	0.47	2.06, 2.06			0.00

**Table 8:** Harmonic ν(CO) Frequencies of the Nine MePFe_2_(CO)Cp_2_ Structures (Scaled Values in cm^–1^ and IR Intensities in km·mol^–1^ in Parentheses) with the Bridging ν(CO) Frequency in Italics

structure	ν(CO) frequencies, cm^–1^
**Fe2PCO-1Q**	1975(812)
**Fe2PCO-2Q**	1968(862)
**Fe2PCO-3T**	1993(777)
**Fe2PCO-4Q**	*1818*(*723*)
**Fe2PCO-5Q**	1962(803)
**Fe2PCO-6Q**	*1861*(*744*)
**Fe2PCO-7T**	1954(742)
**Fe2PCO-8T**	*1824*(*637*)
**Fe2PCO-9S**	1*802*(*613*)

Two of the remaining low-energy MePFe_2_(CO)Cp_2_ structures, namely, **Fe2PCO-8T** and **Fe2PCO-9S** lying 13.4 and 16.7 kcal/mol above **Fe2PCO-1Q**, have trigonal phosphorus atoms located no more than 0.04 Å outside the Fe_2_C(methyl) plane (Figure 12 and Tables 7 and 8). The quintet structure **Fe2PCO-4Q** is similar to **Fe2PCO-8T** and **Fe2PCO-9S** except that its MeP unit is clearly pyramidal with the phosphorus atom lying 0.79 Å outside the Fe_2_P plane. Each of these three structures has a bridging carbonyl group predicted to exhibit ν(CO) frequencies of 1818, 1824, and 1802 cm^–1^ for the quintet, triplet, and singlet structures, respectively. The iron–iron distances of ∼2.5 Å are similar for all three structures with WBI values of 0.26 and 0.29 for the quintet and triplet structures, respectively, suggesting formal single bonds. However, the WBI for the iron–iron interaction in the singlet structure **Fe2PCO-9S** is significantly higher at 0.47, suggesting a formal double bond. Such an Fe=Fe double bond in **Fe2PCO-9S** would give each iron atom the favored 18-electron configuration for a singlet structure by receiving five electrons from a neutral Cp ring, two electrons through the Fe=Fe bond, one electron from its share of the bridging CO group, and two electrons from its share of the four-electron donor bridging MeP unit.

The remaining of the nine low-energy MePFe_2_(CO)Cp_2_ structures, namely, the triplet structure **Fe2PCO-7T** lying 10.7 kcal/mol in energy above **Fe2PCO-1Q**, has a bridging two-electron donor MeP group with a pyramidal phosphorus atom, a terminal carbonyl group bonded to one iron atom, and an Fe–Fe formal single bond of length 2.55 Å with a WBI of 0.32 (Figure 12 and Tables 7 and 8). The iron atom in **Fe2PCO-7T** bearing the terminal carbonyl group attains a 17-electron configuration by receiving two electrons from the carbonyl group, as well as one electron from its share of the bridging MeP unit, one electron from the Fe–Fe single bond, and five electrons from the neutral Cp ring. However, the iron atom in **Fe2PCO-7T** lacking a terminal carbonyl group has only a 15-electron configuration. The localization of the two unpaired electrons on the carbonyl free iron atom in **Fe2PCO-7T** as indicated by its spin density of 1.95 suggests a formal negative charge on the other iron atom bearing the carbonyl group, giving it an 18-electron configuration with no unpaired electrons. This leaves the carbonyl-free iron atom with a formal positive charge and thus a 14-electron configuration consistent with its bearing essentially all of the spin density of the two unpaired electrons of the triplet spin state.

### 3.5 Carbonyl Dissociation Energies

The carbonyl dissociation energies (Δ*H* and Δ*G*) for the processes MePFe_2_(CO)_*n*_Cp_2_ → MePFe_2_(CO)_*n*−1_Cp_2_ + CO (*n* = 4, 3, and 2) based on the lowest energy structures are listed in Table 9. The listings include some examples of such processes involving slightly higher energy structures in which the spin state is preserved upon CO dissociation, thereby avoiding intersystem crossing. All of these carbonyl dissociation processes are seen to be endothermic with the most endothermic processes being those converting the tricarbonyl MePFe_2_(CO)_3_Cp_2_ to the dicarbonyl MePFe_2_(CO)_2_Cp_2._ This is consistent with the experimental observation of the isolation of several RPFe_2_(CO)_3_Cp_2_ tricarbonyls as stable molecules. Note also that carbonyl dissociation from the tetracarbonyl MePFe_2_(CO)_4_Cp_2_ is endothermic. This suggests that the tetracarbonyls RPFe_2_(CO)_4_Cp_2_ might be isolable or at least detectable species. In this connection, Lorenz and co-workers reported ^31^P NMR evidence for the generation of PhPFe_2_(CO)_4_Cp_2_ from the deprotonation of [Cp_2_Fe_2_(CO)_4_(μ-P(H)Ph)]^+^Cl^–^ with the strong base DBU.^26^

**Table 9:** Carbonyl Dissociation Energies of the MePFe_2_(CO)_*n*_Cp_2_ Derivatives (kcal/mol)

reaction	Δ*H*	Δ*G*
MePFe_2_(CO)_4_Cp_2_(**Fe2PCO4-1S**) → MePFe_2_(CO)_3_Cp_2_(**Fe2PCO3-1S**) + CO	17.4	7.2
MePFe_2_(CO)_3_Cp_2_(**Fe2PCO3-1S**) → MePFe_2_(CO)_2_Cp_2_(**Fe2PCO2-1T**) + CO	25.0	11.9
MePFe_2_(CO)_3_Cp_2_(**Fe2PCO3-1S**) → MePFe_2_(CO)_2_Cp_2_(**Fe2PCO2-12S**) + CO	33.4	24.5
MePFe_2_(CO)_2_Cp_2_(**Fe2PCO2-1T**) → MePFe_2_(CO)Cp_2_(**Fe2PCO2-1Q**) + CO	21.2	7.5
MePFe_2_(CO)_2_Cp_2_(**Fe2PCO2-2Q**) → MePFe_2_(CO)Cp_2_(**Fe2PCO2-1Q**) + CO	19.5	7.5

## 4 Summary

Our density functional theory results clearly show that the lowest energy structures of the tricarbonyl MePFe_2_(CO)_3_Cp_2_ are three stereoisomeric singlet structures each having one bridging carbonyl group, an iron–iron bond, and a terminal carbonyl group bonded to each iron atom. These structures are gratifyingly very similar to the experimental PhPFe_2_(CO)_3_Cp_2_ structure as determined by X-ray crystallography.^3^ The lowest energy structures for the carbonyl-richer MePFe_2_(CO)_4_Cp_2_ are two stereoisomers, each consisting of two CpFe(CO)_2_ units bridged only by the MeP group without an iron–iron bond. In all of these MePFe_2_(CO)_*n*_Cp_2_ structures, the phosphorus atom in the MeP group is pseudotetrahedral with a stereochemically active lone pair and thus a donor of only a single electron to each iron atom that it bridges.

In the unsaturated dicarbonyl and monocarbonyl systems MePFe_2_(CO)_*n*_Cp_2_ (*n* = 2 and 1), higher spin state triplet and quintet structures are energetically favored over singlet structures. Such triplet and quintet structures are generated from the singlet structures of the tetracarbonyl and tricarbonyl MePFe_2_(CO)_*n*_Cp_2_ (*n* = 4 and 3) by the loss of carbonyl groups but retaining the pseudotetrahedral phosphorus configuration of the MeP group with a stereoactive lone pair. However, several slightly higher energy structures are found for the highly unsaturated monocarbonyl MePFe_2_(CO)Cp_2_ in which the MeP phosphorus atom has become essentially planar and thus a two-electron donor rather than a one-electron donor to each iron atom. The lowest energy singlet MePFe_2_(CO)Cp_2_ structure, lying nearly 17 kcal/mol above its lowest energy higher spin isomer, has both the planar phosphorus in its bridging MeP unit and the formal Fe=Fe double bond required to give each iron atom the favored 18-electron configuration.

## Supporting Information Available

Distance table for the lowest-lying MePFe_2_Cp_2_ structures; distance table for the lowest-lying MePFe_2_(CO)Cp_2_ structures; distance table for the lowest-lying MePFe_2_(CO)_2_Cp_2_ structures; distance table for the lowest-lying MePFe_2_(CO)_3_Cp_2_ structures; distance table for the lowest-lying MePFe_2_(CO)_4_Cp_2_ structures; orbital energies and HOMO/LUMO gaps; and complete Gaussian reference (ref (24)) (PDF)
Cartesian coordinates of the optimized lowest energy structures (XYZ)

## Supplementary Material

ao3c10460_si_001.pdf

ao3c10460_si_002.xyz
