# Supplementary material for: Decarbonylation Products of Binuclear Methylphosphinidene Complexes of Cyclopentadienyliron Carbonyls: Triplet and Quintet Structures Are Favored Energetically over Singlet Structures with Iron–Iron Multiple Bonding
Source: ACS Omega. 2024 Feb 28;9(10):12125–34. doi: 10.1021/acsomega.3c10460 (PMC10938326; doi:10.1021/acsomega.3c10460)
Supplement: Supplementary file 1 — ao3c10460_si_001.pdf [file ao3c10460_si_001.pdf]

Decarbonylation Products of Binuclear Methylphosphinidine Complexes of Cyclopentadienyliron Carbonyls: Triplet and Quintet Structures Are Favored Energetically over Singlet Structures with Iron-Iron Multiple Bonding

Oleg Rudenco,[1] Alexandru Lupan,[1]*, Radu Silaghi-Dumitrescu, R. Bruce King*[2]

[1]*Faculty of Chemistry and Chemical Engineering, Babeş-Bolyai University, Cluj-Napoca, Romania*

[2]*Department of Chemistry, University of Georgia, Athens, Georgia, 30602*

Supporting Information

Table S1. Distance table for the lowest-lying Cp_2Fe_2PMe structures.
Table S2. Distance table for the lowest-lying $Cp_2Fe_2PMe(CO)$ structures.
Table S3. Distance table for the lowest-lying $Cp_2Fe_2PMe(CO)_2$ structures.
Table S4. Distance table for the lowest-lying $Cp_2Fe_2PMe(CO)_3$ structures.
Table S5. Distance table for the lowest-lying $Cp_2Fe_2PMe(CO)_4$ structures.
Table S6. Orbital energies and HOMO/LUMO gaps.

Complete Gaussian09 Reference (reference 24)

Gaussian 09, Revision E.01, M. J. Frisch, G. W. Trucks, H. B. Schlegel, G. E. Scuseria, M. A. Robb, J. R. Cheeseman, G. Scalmani, V. Barone, B. Mennucci, G. A. Petersson, H. Nakatsuji, M. Caricato, X. Li, H. P. Hratchian, A. F. Izmaylov, J. Bloino, G. Zheng, J. L. Sonnenberg, M. Hada, M. Ehara, K. Toyota, R. Fukuda, J. Hasegawa, M. Ishida, T. Nakajima, Y. Honda, O. Kitao, H. Nakai, T. Vreven, J. A. Montgomery, Jr., J. E. Peralta, F. Ogliaro, M. Bearpark, J. J. Heyd, E. Brothers, K. N. Kudin, V. N. Staroverov, T. Keith, R. Kobayashi, J. Normand, K. Raghavachari, A. Rendell, J. C. Burant, S. S. Iyengar, J. Tomasi, M. Cossi, N. Rega, J. M. Millam, M. Klene, J. E. Knox, J. B. Cross, V. Bakken, C. Adamo, J. Jaramillo, R. Gomperts, R. E. Stratmann, O. Yazyev, A. J. Austin, R. Cammi, C. Pomelli, J. W. Ochterski, R. L. Martin, K. Morokuma, V. G. Zakrzewski, G. A. Voth, P. Salvador, J. J. Dannenberg, S. Dapprich, A. D. Daniels, O. Farkas, J. B. Foresman, J. V. Ortiz, J. Cioslowski, and D. J. Fox, Gaussian, Inc., Wallingford CT, 2013.

Table S1. Distance matrix for the lowest energy Cp_2Fe_2PMe structures as optimized at the PBE0/def2TZVP level. Included are the ranking order, spin multiplicity (S, T or Q), total energy (in a.u.), relative energy (in kcal/mol), symmetry point group Wiberg bond indices for the Fe-Fe bonds, Mulliken charges and the spin density (Cp moieties are omitted for clarity).

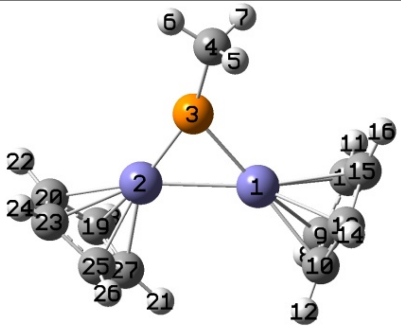

1T. -3294.668393 0.0 Cs
WBI 0.43

Charge and spin density

		1	2
1	Fe	0.311190	1.817096
2	Fe	0.302564	0.481163
3	P	-0.278041	-0.193721
4	C	0.017795	0.000014
9	C	-0.051050	0.012969
10	C	-0.058770	0.000985
13	C	-0.031994	-0.089317
15	C	-0.008124	0.042176
17	C	-0.005018	-0.027203
19	C	-0.048411	-0.011689
20	C	-0.019637	0.039526
23	C	-0.023819	-0.102088
25	C	-0.039182	0.099099
27	C	-0.067502	-0.069012

		1	2	3	4	5
1	Fe	0.000000				
2	Fe	2.576634	0.000000			
3	P	2.272247	2.058816	0.000000		
4	C	3.684110	3.820191	1.831032	0.000000	
5	H	3.886418	4.333997	2.426910	1.091867	0.000000
6	H	4.568298	4.140285	2.433100	1.090709	1.778705
7	H	3.930281	4.451832	2.432413	1.091443	1.771301
8	H	2.925591	4.573886	5.005066	6.443657	6.724730
9	C	2.216485	4.350224	4.423703	5.714600	5.878310
10	C	2.220122	4.351791	4.435322	5.674470	5.603280
11	H	3.010076	5.145965	4.493156	5.275057	5.594660
12	H	2.929756	4.575086	5.023243	6.375117	6.263390
13	C	2.298640	4.696647	4.163816	4.988263	4.722412
14	H	3.017357	5.148668	4.526936	5.140640	4.655023
15	C	2.340728	4.893495	3.973438	4.563709	4.452910
16	H	3.072741	5.478918	4.181637	4.319331	4.111880
17	C	2.293964	4.695501	4.144823	5.061941	5.232634
18	H	4.309330	2.877672	4.542694	6.258610	6.967873
19	C	4.010459	2.137496	4.061665	5.811074	6.432757
20	C	4.668125	2.214587	4.013218	5.556024	6.208225
21	H	3.483877	2.852566	4.577928	6.387650	6.704993
22	H	5.389907	2.959615	4.423320	5.764204	6.533599
23	C	4.654845	2.207053	3.961211	5.426819	5.866098
24	H	5.369505	2.950484	4.335740	5.527987	5.904354
25	C	3.977034	2.116813	3.969829	5.601223	5.878221
26	H	4.254785	2.853249	4.392875	5.892073	5.973905
27	C	3.539110	2.099981	4.060380	5.863137	6.262438

		6	7	8	9	10
6	H	0.000000				
7	H	1.779603	0.000000			
8	H	7.319482	6.431323	0.000000		
9	C	6.661003	5.706586	1.080011	0.000000	
10	C	6.629052	5.866336	2.237845	1.424174	0.000000
11	H	6.238087	4.844491	2.686918	2.229178	3.339391
12	H	7.263194	6.697178	2.697246	2.237808	1.080101
13	C	6.015226	5.124991	3.338854	2.294722	1.416296
14	H	6.132684	5.394461	4.350479	3.339241	2.228725
15	C	5.640820	4.406672	3.334020	2.290413	2.290265
16	H	5.407064	4.005358	4.343721	3.333699	3.333455

17	C	6.072356	4.826867	2.230897	1.416880	2.294937
18	H	6.527600	6.583491	4.467781	4.910424	5.466208
19	C	6.037483	6.299985	4.700768	4.963539	5.246164
20	C	5.532590	6.178232	5.941162	6.048509	6.195079
21	H	6.896616	6.899720	3.567823	3.829160	3.747491
22	H	5.578619	6.319924	6.684158	6.811464	7.078898
23	C	5.395484	6.237757	6.282720	6.226251	6.047823
24	H	5.320117	6.433115	7.254645	7.110517	6.833056
25	C	5.825250	6.387894	5.371140	5.303401	4.950843
26	H	6.158574	6.750018	5.726005	5.546049	4.917564
27	C	6.230562	6.453344	4.260553	4.414629	4.361123
		11	12	13	14	15
11	H	0.000000				
12	H	4.350751	0.000000			
13	C	3.332081	2.230430	0.000000		
14	H	4.341328	2.686433	1.080127	0.000000	
15	C	2.225568	3.333998	1.412482	2.225662	0.000000
16	H	2.679674	4.343552	2.224643	2.679569	1.079947
17	C	1.080079	3.339191	2.288710	3.332051	1.412276
18	H	5.716582	5.555898	6.345580	7.088177	6.396979
19	C	5.963152	5.248383	6.046801	6.658630	6.264497
20	C	6.874840	6.218942	6.784081	7.260378	6.994396
21	H	5.555791	3.398065	4.831606	5.382093	5.445975
22	H	7.372656	7.189561	7.599391	8.102916	7.664338
23	C	7.288204	5.941712	6.533428	6.813329	6.970286
24	H	8.094448	6.728973	7.170576	7.328333	7.624040
25	C	6.709701	4.688887	5.570577	5.829429	6.212734
26	H	7.119952	4.507413	5.459480	5.534490	6.305066
27	C	5.860923	4.153201	5.237488	5.736132	5.748783
		16	17	18	19	20
16	H	0.000000				
17	C	2.224533	0.000000			
18	H	7.170706	5.568842	0.000000		
19	C	7.024911	5.651625	1.079756	0.000000	
20	C	7.623353	6.569355	2.228510	1.418677	0.000000
21	H	6.395521	4.934538	2.698568	2.237066	3.343218
22	H	8.211556	7.197158	2.678983	2.228463	1.079907
23	C	7.583921	6.798651	3.329535	2.288599	1.404488
24	H	8.143699	7.592920	4.338255	3.331923	2.218216
25	C	6.947052	6.070847	3.337671	2.291817	2.286343
26	H	7.030731	6.355124	4.350850	3.336767	3.327911
27	C	6.595402	5.310229	2.235066	1.421236	2.299903
		21	22	23	24	25
21	H	0.000000				
22	H	4.352212	0.000000			
23	C	3.346085	2.217892	0.000000		

24	H	4.355809	2.672787	1.079604	0.000000	
25	C	2.240134	3.330024	1.419429	2.230191	0.000000
26	H	2.700159	4.337447	2.230054	2.683141	1.079594
27	C	1.079171	3.341973	2.303254	3.345897	1.425296

		26	27
26	H	0.000000	
27	C	2.237907	0.000000

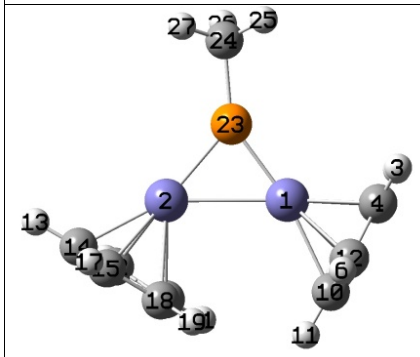

2Q. -3294.661215 4.50 C2v
WBI 0.52

Charge and spin density

		1	2
1	Fe	0.246445	0.390744
2	Fe	0.320988	3.514182
4	C	-0.164037	-0.058354
5	C	0.199670	0.066188
8	C	-0.051820	-0.005178
10	C	-0.128238	-0.055208
12	C	-0.029668	0.019182
14	C	-0.067218	0.003798
15	C	-0.084746	0.002362
18	C	-0.067169	0.041701
20	C	-0.072298	0.037995
22	C	-0.085106	0.002971
23	P	-0.612207	-0.037664
24	C	0.595404	0.077281

		1	2	3	4	5
1	Fe	0.000000				
2	Fe	2.632388	0.000000			
3	H	2.835401	5.290181	0.000000		
4	C	2.099875	4.681402	1.079261	0.000000	
5	C	2.094927	4.631271	2.242521	1.431312	0.000000
6	H	2.934511	4.731339	2.689005	2.229717	3.346095
7	H	2.829202	5.207129	2.700028	2.242689	1.079160
8	C	2.172451	4.280489	3.341534	2.298894	1.415547
9	H	2.915312	4.567156	4.354755	3.344564	2.230252
10	C	2.207881	4.091949	3.328327	2.284604	2.287349
11	H	2.960139	4.217794	4.337536	3.327445	3.330184
12	C	2.191020	4.378988	2.229484	1.415546	2.300913
13	H	5.501066	2.886451	8.140465	7.532796	7.449589
14	C	4.657494	2.161687	7.334745	6.657282	6.577062
15	C	4.291765	2.215202	6.715257	6.073341	6.264088
16	H	4.811997	2.943937	7.644917	6.814996	6.265193
17	H	4.885609	2.944433	7.034000	6.512062	6.901442
18	C	3.552057	2.272407	5.965142	5.182794	5.308100
19	H	3.565571	3.013352	5.584486	4.815052	5.142926
20	C	3.518821	2.271868	6.204463	5.307619	5.044533
21	H	3.506712	3.015145	6.062053	5.069829	4.617347
22	C	4.247882	2.215035	7.059505	6.246768	5.900541
23	P	2.047584	2.183023	3.964576	3.689480	3.700007
24	C	3.729244	3.705115	4.851279	4.913853	4.936693
25	H	3.983826	4.510469	4.404638	4.697173	4.877058
26	H	4.199968	4.130636	5.440292	5.429908	5.175006
27	H	4.446286	3.858896	5.663014	5.742774	5.884570

		6	7	8	9	10
6	H	0.000000				
7	H	4.355683	0.000000			
8	C	3.337819	2.230579	0.000000		
9	H	4.344559	2.691536	1.079678	0.000000	
10	C	2.223592	3.331665	1.414428	2.226873	0.000000
11	H	2.673920	4.341244	2.224649	2.677928	1.080232
12	C	1.079746	3.343176	2.295676	3.337342	1.410739
13	H	7.221384	7.995544	6.890203	6.941950	6.604193
14	C	6.276746	7.197654	5.930369	6.001982	5.578234
15	C	5.288259	7.038639	5.635215	5.913745	4.972117

16	H	6.935832	6.699821	5.438922	5.112737	5.534842
17	H	5.507382	7.703189	6.389789	6.785225	5.600680
18	C	4.337554	6.169904	4.496316	4.764082	3.735899
19	H	3.580359	6.111329	4.336320	4.757941	3.291273
20	C	4.959195	5.776035	4.068878	4.027598	3.703591
21	H	4.877385	5.336894	3.449088	3.224604	3.224428
22	C	6.112244	6.473882	5.080962	4.945880	4.933282
23	P	4.548534	3.984215	4.055859	4.565595	4.235266
24	C	6.021252	4.896160	5.597354	6.070464	5.933490
25	H	5.940828	4.760464	5.771129	6.360183	6.105828
26	H	6.760010	4.950883	5.810071	6.115838	6.366222
27	H	6.561726	5.932552	6.446790	6.924665	6.632356
		11	12	13	14	15
11	H	0.000000				
12	C	2.220988	0.000000			
13	H	6.383604	7.045244	0.000000		
14	C	5.313951	6.083446	1.080564	0.000000	
15	C	4.643328	5.300967	2.234654	1.422286	0.000000
16	H	5.293764	6.421642	2.693913	2.236322	3.346110
17	H	5.294011	5.707959	2.693714	2.236428	1.079634
18	C	3.282255	4.265687	3.331033	2.287224	1.410549
19	H	2.737215	3.692653	4.341510	3.331038	2.221719
20	C	3.281978	4.583588	3.331119	2.287222	2.291851
21	H	2.738490	4.357835	4.341387	3.330852	3.333844
22	C	4.643414	5.716376	2.234553	1.422011	2.302051
23	P	4.858071	4.051386	4.767108	4.266904	4.233999
24	C	6.642178	5.574713	5.703131	5.500886	5.641902
25	H	6.922729	5.535076	6.683232	6.419798	6.398823
26	H	7.081554	6.180956	5.963503	5.820438	6.187686
27	H	7.242867	6.248776	5.383937	5.357428	5.521266
		16	17	18	19	20
16	H	0.000000				
17	H	4.357206	0.000000			
18	C	3.335646	2.225114	0.000000		
19	H	4.343847	2.678605	1.079436	0.000000	
20	C	2.225121	3.335683	1.417310	2.229128	0.000000
21	H	2.678258	4.343860	2.229213	2.684077	1.079302
22	C	1.079637	3.346012	2.291881	3.333945	1.410750
23	P	4.689903	4.687126	4.156818	4.535826	4.157433
24	C	5.934937	5.932900	5.838287	6.265555	5.838571
25	H	6.896537	6.618575	6.493922	6.772846	6.586933
26	H	5.885822	6.602870	6.353556	6.879153	6.119646
27	H	6.086665	5.663661	5.973560	6.460056	6.107034
		21	22	23	24	25
21	H	0.000000				
22	C	2.221691	0.000000			

23	P	4.540659	4.235852	0.000000		
24	C	6.269737	5.643224	1.828480	0.000000	
25	H	6.944736	6.552287	2.431777	1.091067	0.000000
26	H	6.468805	5.792959	2.416495	1.093005	1.774289
27	H	6.695510	5.754309	2.438303	1.090871	1.783063

		26	27
26	H	0.000000	
27	H	1.773091	0.000000

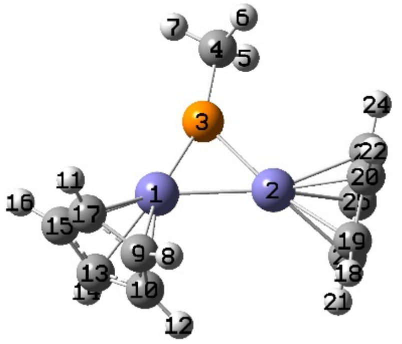

3Q. -3294.660133 5.18 Cs
WBI 0.40

Charge and spin density

1	Fe	0.247572	2.151001
2	Fe	0.292197	2.810433
3	P	-0.112932	-0.897007
4	C	0.061477	-0.027114
9	C	-0.065296	-0.014873
10	C	-0.035572	-0.033226
13	C	-0.054476	0.016347
15	C	0.009311	-0.002877
17	C	-0.047136	-0.008326
19	C	-0.035953	0.002697
20	C	-0.108885	0.007101
23	C	-0.016651	-0.041494
25	C	-0.053257	0.034647
27	C	-0.080399	0.002691

		1	2	3	4	5
1	Fe	0.000000				
2	Fe	2.521330	0.000000			
3	P	2.036717	2.190642	0.000000		
4	C	3.810091	3.582015	1.827317	0.000000	
5	H	4.378216	3.746871	2.425224	1.091936	0.000000
6	H	4.376048	3.860486	2.421110	1.092335	1.772757
7	H	4.145052	4.469621	2.432158	1.091107	1.782874
8	H	2.858494	3.850925	4.502680	6.254078	6.886979
9	C	2.115482	3.720255	4.020864	5.803804	6.421121
10	C	2.126472	3.573928	4.048520	5.847364	6.258626
11	H	2.897071	5.020910	4.367033	5.794634	6.670813
12	H	2.875875	3.582391	4.551938	6.333012	6.600769
13	C	2.192707	4.224728	4.023114	5.657176	6.069632
14	H	2.929718	4.691815	4.454240	5.937649	6.194747
15	C	2.197909	4.671552	3.952578	5.463825	6.091363
16	H	2.943293	5.442487	4.341683	5.593528	6.244783
17	C	2.160222	4.410046	3.961539	5.567680	6.319866
18	H	4.657111	2.887161	5.017117	6.369174	6.477076
19	C	4.355082	2.160131	4.326527	5.516071	5.524789
20	C	4.549778	2.187371	3.966935	4.806079	4.855468
21	H	4.722006	2.963234	4.864433	6.040621	5.765671
22	H	4.987086	2.917418	4.406035	5.152887	5.354669
23	C	4.692639	2.246959	3.614732	4.052243	3.832816
24	H	5.222189	2.994711	3.776293	3.714720	3.428739
25	C	4.622335	2.284919	3.822590	4.452543	4.040788
26	H	5.072290	3.027181	4.115427	4.472148	3.828337
27	C	4.406214	2.226408	4.246247	5.327596	5.100041

		6	7	8	9	10
6	H	0.000000				
7	H	1.780098	0.000000			
8	H	6.480987	6.705364	0.000000		
9	C	6.191372	6.146500	1.079272	0.000000	
10	C	6.427341	6.210765	2.239937	1.427235	0.000000
11	H	6.084527	5.806996	2.692522	2.231819	3.341761
12	H	6.904558	6.818816	2.699826	2.240347	1.079389
13	C	6.399211	5.792364	3.341835	2.298819	1.417104
14	H	6.798232	6.023785	4.353464	3.343143	2.229875

15	C	6.120169	5.420621	3.337236	2.293485	2.289266
16	H	6.301694	5.337476	4.346794	3.336294	3.332183
17	C	5.996938	5.664484	2.231845	1.417359	2.296384
18	H	6.418501	7.305278	4.076258	4.544820	4.486585
19	C	5.576452	6.499546	4.455417	4.740622	4.590339
20	C	4.629730	5.848691	4.962243	5.255857	5.345412
21	H	6.449697	6.923420	5.371953	5.303947	4.528178
22	H	4.771414	6.162902	5.059087	5.507254	5.853969
23	C	3.962082	5.135678	5.825794	5.886598	5.769894
24	H	3.435085	4.797410	6.569340	6.598811	6.566819
25	C	4.688030	5.461580	5.928431	5.840588	5.383548
26	H	4.845090	5.407321	6.720648	6.496579	5.887741
27	C	5.604623	6.285975	5.141517	5.153201	4.624580
		11	12	13	14	15
11	H	0.000000				
12	H	4.353393	0.000000			
13	C	3.333083	2.229370	0.000000		
14	H	4.340795	2.686313	1.079809	0.000000	
15	C	2.227302	3.331680	1.410236	2.221895	0.000000
16	H	2.680130	4.340703	2.222161	2.674339	1.079807
17	C	1.079642	3.340129	2.291504	3.334039	1.416734
18	H	6.381199	3.954735	5.716511	6.253702	6.384492
19	C	6.441029	4.149945	5.644198	6.102140	6.298765
20	C	6.557682	5.142984	6.257221	6.777271	6.668158
21	H	7.235167	3.801425	5.335551	5.400191	6.380617
22	H	6.591940	5.751274	6.811296	7.448038	7.049778
23	C	7.070516	5.603782	6.427966	6.776751	6.876781
24	H	7.519314	6.511595	7.098344	7.439957	7.410202
25	C	7.289295	5.047276	5.975523	6.131274	6.680092
26	H	7.877221	5.562306	6.275793	6.260644	7.030126
27	C	6.915172	4.084489	5.457089	5.667989	6.310942
		16	17	18	19	20
16	H	0.000000				
17	C	2.228167	0.000000			
18	H	7.381619	5.783566	0.000000		
19	C	7.220038	5.831367	1.080482	0.000000	
20	C	7.489581	6.122736	2.233811	1.419888	0.000000
21	H	7.241723	6.373499	2.693767	2.237940	3.345338
22	H	7.863456	6.318687	2.695297	2.235376	1.079549
23	C	7.573429	6.582502	3.334776	2.289592	1.418071
24	H	8.003789	7.131406	4.346201	3.333914	2.229662
25	C	7.389221	6.617094	3.333188	2.290558	2.296885
26	H	7.637928	7.163590	4.343974	3.335048	3.340125
27	C	7.159242	6.161527	2.236310	1.424812	2.301657
		21	22	23	24	25
21	H	0.000000				

22	H	4.357255	0.000000			
23	C	3.332447	2.231806	0.000000		
24	H	4.340773	2.686911	1.080071	0.000000	
25	C	2.222088	3.339858	1.416323	2.228307	0.000000
26	H	2.675658	4.349354	2.228756	2.682957	1.080246
27	C	1.079910	3.346342	2.288579	3.331055	1.408195

		26	27
26	H	0.000000	
27	C	2.220498	0.000000

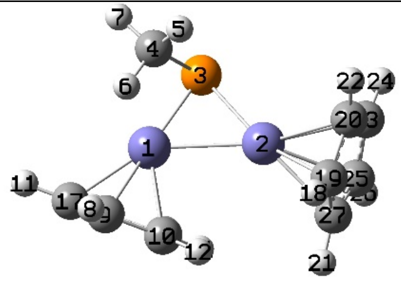

4Q. -3294.658929 5.94 C1
WBI 0.30

Charge and spin density

1	Fe	0.190442	0.469155
2	Fe	0.426132	3.625987
3	P	-0.351451	-0.243082
4	C	0.089078	-0.003605
9	C	0.009171	0.024569
10	C	-0.051060	-0.070352
13	C	0.038992	0.041445
15	C	-0.021372	-0.050483
17	C	-0.044481	0.048437
19	C	-0.075284	0.009818
20	C	-0.068859	0.047227
23	C	0.003127	0.050652
25	C	-0.063103	0.024505
27	C	-0.081331	0.025727

		1	2	3	4	5
1	Fe	0.000000				
2	Fe	2.528464	0.000000			
3	P	2.162784	2.341036	0.000000		
4	C	2.260807	3.211644	1.880563	0.000000	
5	H	3.033961	3.320198	2.530727	1.089098	0.000000
6	H	1.804250	3.368936	2.577600	1.148660	1.746416
7	H	2.976352	4.239102	2.494279	1.090431	1.784720
8	H	2.892746	4.508043	4.924725	4.141523	4.498622
9	C	2.127206	3.930602	4.274161	3.866201	4.411795
10	C	2.112379	3.057276	4.010010	4.144914	4.620683
11	H	2.858584	5.339566	4.660577	4.145974	5.003339
12	H	2.905319	3.010842	4.528590	4.671202	4.932307
13	C	2.048694	3.120524	3.622445	4.285315	4.983661
14	H	2.815680	3.125098	3.857639	4.902814	5.558196
15	C	2.063511	4.039936	3.717033	4.141302	5.040817
16	H	2.804418	4.682977	3.977888	4.620527	5.613947
17	C	2.102135	4.447064	4.113745	3.868258	4.690119
18	H	4.682706	2.960642	5.139417	5.255162	4.883993
19	C	4.401559	2.244594	4.509269	4.966321	4.675224
20	C	4.683818	2.301182	4.135114	4.672587	4.237890
21	H	4.617751	2.950064	5.137278	6.013309	6.094060
22	H	5.142652	3.016585	4.468576	4.676191	4.015787
23	C	4.814567	2.327777	3.883700	4.953434	4.742344
24	H	5.350427	3.042049	3.999283	5.178065	4.955999
25	C	4.626063	2.288402	4.129469	5.390043	5.404564
26	H	5.050811	3.004421	4.467797	5.971272	6.127645
27	C	4.363360	2.235329	4.505912	5.399104	5.370912

		6	7	8	9	10
6	H	0.000000				
7	H	1.757656	0.000000			
8	H	3.035875	4.715795	0.000000		
9	C	2.871386	4.476134	1.080153	0.000000	
10	C	3.411884	4.960247	2.224183	1.413132	0.000000
11	H	3.271714	4.221642	2.680948	2.228582	3.338434
12	H	3.997562	5.601153	2.679763	2.226491	1.079611
13	C	3.793161	4.984142	3.339258	2.294491	1.431462

14	H	4.600629	5.639653	4.350365	3.338345	2.244113
15	C	3.589711	4.554155	3.340625	2.297935	2.308700
16	H	4.246330	4.860145	4.350916	3.341747	3.351419
17	C	3.005056	4.206632	2.229900	1.418489	2.296978
18	H	5.056791	6.337536	4.969674	4.896969	4.014236
19	C	4.956185	6.051415	5.308572	5.033664	4.017405
20	C	4.978119	5.694932	6.074660	5.779938	4.901508
21	H	5.837248	7.050248	5.495486	4.944948	3.580465
22	H	5.058413	5.635828	6.392500	6.238804	5.550273
23	C	5.416364	5.900594	6.717019	6.212530	5.209474
24	H	5.833764	6.005614	7.500119	6.969215	6.047022
25	C	5.654054	6.363082	6.446246	5.812804	4.606340
26	H	6.270201	6.867376	7.054855	6.302955	5.053788
27	C	5.387914	6.451945	5.574174	5.057073	3.793816
		11	12	13	14	15
11	H	0.000000				
12	H	4.346518	0.000000			
13	C	3.337588	2.247105	0.000000		
14	H	4.347663	2.708326	1.079929	0.000000	
15	C	2.231383	3.352510	1.421205	2.231889	0.000000
16	H	2.693306	4.362469	2.231799	2.685493	1.078949
17	C	1.080051	3.339600	2.292879	3.336034	1.417747
18	H	6.952277	3.170598	4.870562	4.939803	6.033801
19	C	6.938475	3.324152	4.573506	4.476429	5.766253
20	C	7.430720	4.424352	5.265500	5.149374	6.299860
21	H	6.818708	2.870134	3.768652	3.288407	5.175880
22	H	7.824127	5.102539	6.028994	6.038122	6.943906
23	C	7.652942	4.884894	5.188537	4.835933	6.212523
24	H	8.203734	5.836085	5.887850	5.513116	6.777517
25	C	7.322085	4.246713	4.430413	3.869176	5.612049
26	H	7.635663	4.811617	4.588916	3.822838	5.724834
27	C	6.866592	3.176207	3.985459	3.586263	5.306548
		16	17	18	19	20
16	H	0.000000				
17	C	2.233475	0.000000			
18	H	6.939057	6.040016	0.000000		
19	C	6.563833	5.991098	1.080037	0.000000	
20	C	6.980178	6.564258	2.229921	1.415745	0.000000
21	H	5.876138	5.750524	2.695302	2.235403	3.335706
22	H	7.642157	7.048615	2.686443	2.228631	1.080316
23	C	6.711284	6.760299	3.334846	2.291291	1.412686
24	H	7.154659	7.375952	4.343910	3.333999	2.224924
25	C	6.092883	6.337205	3.340362	2.295948	2.288051
26	H	6.042350	6.650716	4.352339	3.340670	3.331348
27	C	5.989837	5.837350	2.236285	1.421704	2.290995
		21	22	23	24	25

21	H	0.000000				
22	H	4.347731	0.000000			
23	C	3.333152	2.224877	0.000000		
24	H	4.342873	2.678467	1.079730	0.000000	
25	C	2.231246	3.331160	1.412077	2.224078	0.000000
26	H	2.689378	4.340125	2.224820	2.678457	1.080100
27	C	1.080593	3.335877	2.289200	3.332401	1.417687

		26	27
26	H	0.000000	
27	C	2.231732	0.000000

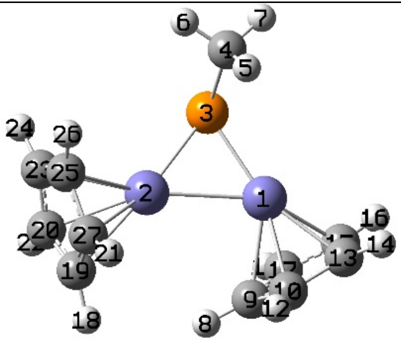

5Q. -3294.651941 10.32 C1
WBI 0.32

Charge and spin density

1	Fe	0.236055	0.352524
2	Fe	0.451887	3.562271
3	P	-0.287955	-0.054752
4	C	-0.002719	0.023723
9	C	-0.008510	-0.106043
10	C	-0.051152	-0.003156
13	C	-0.025334	-0.004147
15	C	-0.021342	-0.032008
17	C	-0.035035	0.116505
19	C	-0.023867	0.010618
20	C	-0.078467	0.054808
23	C	0.022065	0.033073
25	C	-0.077352	0.021237
27	C	-0.098273	0.025346

		1	2	3	4	5
1	Fe	0.000000				
2	Fe	2.565077	0.000000			
3	P	2.186673	2.302382	0.000000		
4	C	3.374075	3.478897	1.866250	0.000000	
5	H	3.364151	3.607457	2.511581	1.089653	0.000000
6	H	4.250858	3.730618	2.456381	1.091738	1.774621
7	H	3.844144	4.420067	2.439995	1.092494	1.771237
8	H	2.954971	3.234277	4.453001	5.976036	5.963086
9	C	2.191496	3.323609	4.045300	5.472225	5.411691
10	C	2.151809	3.640035	4.253890	5.258027	4.927656
11	H	2.900469	4.325028	4.133834	5.807419	6.101226
12	H	2.900442	3.824995	4.807842	5.602532	5.110447
13	C	2.125169	4.386318	4.248421	5.061883	4.742831
14	H	2.856536	5.093307	4.790467	5.237987	4.755960
15	C	2.118779	4.530379	4.002714	5.129734	5.104794
16	H	2.853228	5.331408	4.378877	5.371232	5.429279
17	C	2.171334	3.940488	3.885092	5.396703	5.511174
18	H	4.629401	2.977983	5.192432	6.422947	6.373876
19	C	4.379892	2.251968	4.525426	5.639517	5.646659
20	C	4.647889	2.308450	4.215831	5.445379	5.714054
21	H	4.682459	2.929158	4.971698	5.412138	5.017971
22	H	5.064622	3.032036	4.636738	6.068247	6.460524
23	C	4.823169	2.300959	3.853184	4.687754	4.995221
24	H	5.360511	3.016193	3.986570	4.711200	5.202628
25	C	4.684476	2.245998	3.972617	4.402811	4.407584
26	H	5.148109	2.956775	4.230461	4.188038	4.104744
27	C	4.411338	2.219609	4.394442	5.050372	4.873069

		6	7	8	9	10
6	H	0.000000				
7	H	1.764662	0.000000			
8	H	6.595325	6.634110	0.000000		
9	C	6.229448	6.018425	1.079552	0.000000	
10	C	6.097704	5.797389	2.234678	1.422716	0.000000
11	H	6.579341	6.058337	2.679226	2.222816	3.335225
12	H	6.360051	6.252864	2.688906	2.233444	1.079598

13	C	6.049232	5.347387	3.338743	2.295921	1.415188
14	H	6.261337	5.427996	4.349869	3.340423	2.228970
15	C	6.118345	5.266584	3.335749	2.291998	2.292791
16	H	6.396633	5.286546	4.343384	3.333656	3.336997
17	C	6.242029	5.710241	2.223356	1.409767	2.291537
18	H	6.618744	7.375789	2.884077	3.743119	4.296188
19	C	5.715841	6.633714	3.346046	4.027912	4.506318
20	C	5.367439	6.385386	3.960208	4.597601	5.300989
21	H	5.481233	6.470461	4.435677	4.769332	4.549127
22	H	6.016388	6.916324	4.073678	4.797484	5.735901
23	C	4.376010	5.666478	4.933413	5.353943	5.830671
24	H	4.221079	5.596574	5.742458	6.102553	6.634987
25	C	4.125390	5.476981	5.030458	5.339652	5.472187
26	H	3.740021	5.256661	5.914021	6.097411	6.049414
27	C	5.041861	6.116395	4.147252	4.565884	4.636818
		11	12	13	14	15
11	H	0.000000				
12	H	4.343509	0.000000			
13	C	3.338389	2.229582	0.000000		
14	H	4.348951	2.687814	1.079975	0.000000	
15	C	2.229979	3.337343	1.420397	2.233341	0.000000
16	H	2.683179	4.349032	2.235374	2.694101	1.079366
17	C	1.079610	3.333603	2.295773	3.339892	1.419749
18	H	5.233761	4.088289	5.604012	6.424619	5.905162
19	C	5.377519	4.348532	5.692529	6.468023	5.969691
20	C	5.399142	5.370662	6.308287	7.140530	6.312074
21	H	6.572362	3.974205	5.667265	6.117521	6.405685
22	H	5.235335	5.939533	6.705625	7.624592	6.507179
23	C	6.120715	5.867090	6.679498	7.382119	6.748305
24	H	6.566157	6.767897	7.350467	8.043451	7.284728
25	C	6.519656	5.293321	6.349401	6.900488	6.706923
26	H	7.277740	5.820847	6.798001	7.213251	7.236882
27	C	6.105593	4.293372	5.727014	6.310392	6.237821
		16	17	18	19	20
16	H	0.000000				
17	C	2.230623	0.000000			
18	H	6.917162	4.910915	0.000000		
19	C	6.927179	5.066001	1.080248	0.000000	
20	C	7.149308	5.327031	2.228912	1.415047	0.000000
21	H	7.373407	5.937706	2.691613	2.234424	3.337397
22	H	7.292956	5.370859	2.685405	2.228212	1.080307
23	C	7.504090	5.981654	3.332129	2.288482	1.410756
24	H	7.934253	6.543956	4.341344	3.331397	2.223359
25	C	7.519071	6.145724	3.337646	2.293365	2.289714
26	H	7.987447	6.847503	4.349609	3.338182	3.332519
27	C	7.173022	5.617968	2.233810	1.420936	2.293591

	21	22	23	24	25
21 H	0.000000				
22 H	4.348599	0.000000			
23 C	3.337108	2.223183	0.000000		
24 H	4.346892	2.677057	1.079574	0.000000	
25 C	2.232514	3.333244	1.416636	2.228239	0.000000
26 H	2.691613	4.341874	2.228957	2.683181	1.079872
27 C	1.080005	3.338002	2.292712	3.335688	1.418013

	26	27
26 H	0.000000	
27 C	2.231988	0.000000

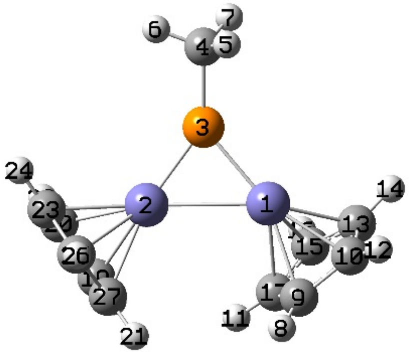

6T. -3294.632884 22.28 C1
WBI 0.41

Charge and spin density

	1	2
1 Fe	0.287468	1.350121
2 Fe	0.264965	1.259704
3 P	-0.257513	-0.406426
4 C	0.043895	-0.014167
9 C	-0.060804	-0.001673
10 C	-0.043516	-0.019533
13 C	-0.036799	-0.005222
15 C	-0.008043	0.002376
17 C	-0.017811	-0.044249
19 C	-0.026939	-0.016018
20 C	-0.030763	-0.043835
23 C	-0.006365	0.001265
25 C	-0.045529	-0.046277
27 C	-0.062248	-0.016065

	1	2	3	4	5
1 Fe	0.000000				
2 Fe	2.631332	0.000000			
3 P	2.157936	2.084612	0.000000		
4 C	3.684990	3.705343	1.831560	0.000000	
5 H	3.834038	4.248380	2.428908	1.092009	0.000000
6 H	4.499037	3.891810	2.442035	1.090291	1.781769
7 H	4.097400	4.409482	2.424178	1.092428	1.768409
8 H	2.877717	4.148649	4.773248	6.301536	6.231105
9 C	2.136918	3.873946	4.214946	5.774849	5.789943
10 C	2.123355	4.561613	4.224436	5.495651	5.380955
11 H	2.945774	3.319666	4.385445	6.199826	6.584048
12 H	2.857938	5.310008	4.788069	5.807843	5.491126
13 C	2.136659	4.572355	3.972278	5.221260	5.309891
14 H	2.874880	5.324547	4.356460	5.303387	5.356754
15 C	2.173809	3.911289	3.818918	5.368079	5.701581
16 H	2.905379	4.185276	4.055991	5.540296	6.039607
17 C	2.186838	3.426447	3.987840	5.718180	5.994257
18 H	4.498879	2.877027	4.701034	6.396453	7.054233
19 C	4.215440	2.134384	4.145149	5.759215	6.370948
20 C	4.672404	2.128288	3.893447	5.200015	5.922038
21 H	4.059112	2.914088	4.756793	6.395492	6.663820
22 H	5.279784	2.863624	4.266990	5.406123	6.262715
23 C	4.766767	2.194918	3.817189	4.873544	5.420811
24 H	5.408614	2.933891	4.100120	4.759317	5.298794
25 C	4.367978	2.220856	4.006747	5.254466	5.581860
26 H	4.708948	2.959222	4.414340	5.459171	5.589543
27 C	3.986332	2.170805	4.189389	5.768516	6.156135

	6	7	8	9	10
6 H	0.000000				
7 H	1.779638	0.000000			
8 H	6.985241	6.884372	0.000000		
9 C	6.545134	6.222051	1.079713	0.000000	
10 C	6.421171	5.779296	2.231480	1.418642	0.000000
11 H	6.771348	6.590197	2.689277	2.232916	3.340288

12	H	6.762364	6.108240	2.688507	2.231836	1.080166
13	C	6.188725	5.263896	3.338060	2.294188	1.419158
14	H	6.335188	5.146268	4.350424	3.339135	2.235102
15	C	6.193606	5.453441	3.331489	2.288309	2.291928
16	H	6.309767	5.473647	4.341069	3.331537	3.334434
17	C	6.426898	6.053640	2.230737	1.419245	2.297421
18	H	6.539905	6.889352	4.859904	4.673588	5.778885
19	C	5.810757	6.380153	4.716771	4.629312	5.712029
20	C	5.011647	5.833415	5.744035	5.592299	6.489956
21	H	6.617797	7.171239	3.227340	3.631840	4.939552
22	H	5.129861	5.892581	6.628847	6.359661	7.163203
23	C	4.558215	5.706950	5.734721	5.750685	6.606791
24	H	4.219441	5.616752	6.596807	6.600993	7.336799
25	C	5.148789	6.169256	4.697316	4.927559	5.915440
26	H	5.342597	6.463261	4.778596	5.170198	6.097822
27	C	5.865213	6.551623	3.921434	4.119377	5.293370
		11	12	13	14	15
11	H	0.000000				
12	H	4.350971	0.000000			
13	C	3.337427	2.233193	0.000000		
14	H	4.344662	2.695526	1.079503	0.000000	
15	C	2.225000	3.336657	1.417397	2.228504	0.000000
16	H	2.679443	4.345716	2.227166	2.679646	1.079622
17	C	1.078353	3.341272	2.295788	3.337451	1.413124
18	H	2.769018	6.746200	5.655685	6.541790	4.431565
19	C	3.136176	6.592554	5.728407	6.616315	4.672881
20	C	4.323286	7.316992	6.403824	7.168056	5.444448
21	H	2.905480	5.626181	5.491826	6.523968	4.748261
22	H	4.941761	8.032712	6.887451	7.557363	5.862715
23	C	4.971501	7.274811	6.749994	7.517142	6.028855
24	H	5.971157	7.933250	7.459112	8.143801	6.835789
25	C	4.420527	6.507935	6.326170	7.201421	5.704907
26	H	5.073686	6.529201	6.700746	7.566089	6.272488
27	C	3.208798	6.026044	5.655850	6.619129	4.844729
		16	17	18	19	20
16	H	0.000000				
17	C	2.224823	0.000000			
18	H	4.396994	3.662462	0.000000		
19	C	4.779403	3.856236	1.079777	0.000000	
20	C	5.442319	4.870300	2.234658	1.421329	0.000000
21	H	5.296619	3.483396	2.696250	2.232964	3.336191
22	H	5.666985	5.490002	2.693838	2.235057	1.079640
23	C	6.225465	5.362145	3.343878	2.300163	1.421992
24	H	7.005104	6.278573	4.354010	3.343430	2.234152
25	C	6.120515	4.789554	3.340105	2.295688	2.288360
26	H	6.815550	5.315590	4.349066	3.337954	3.332464

27	C	5.247440	3.778377	2.233820	1.417573	2.290357

		21	22	23	24	25
21	H	0.000000				
22	H	4.348903	0.000000			
23	C	3.329499	2.232934	0.000000		
24	H	4.338095	2.688904	1.079522	0.000000	
25	C	2.226309	3.330346	1.406627	2.220130	0.000000
26	H	2.676742	4.340725	2.219875	2.674928	1.079998
27	C	1.079906	3.335055	2.288563	3.331915	1.417296

		26	27
26	H	0.000000	
27	C	2.227714	0.000000

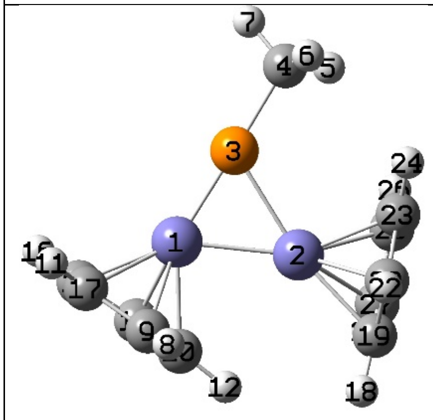

7S. -3294.596918 44.85 Cs
WBI 0.72

Charge and spin density

1	Fe	0.159137
2	Fe	0.177737
3	P	-0.210483
4	C	0.029539
9	C	-0.039924
10	C	0.046352
13	C	-0.061243
15	C	-0.016939
17	C	-0.016179
19	C	0.009036
20	C	-0.056500
23	C	0.052084
25	C	-0.021179
27	C	-0.051438

		1	2	3	4	5
1	Fe	0.000000				
2	Fe	2.267387	0.000000			
3	P	1.997319	2.265880	0.000000		
4	C	3.827173	3.507676	1.830196	0.000000	
5	H	4.343361	3.568265	2.444049	1.091425	0.000000
6	H	4.332143	3.844272	2.430439	1.092384	1.777712
7	H	4.301162	4.419926	2.421767	1.091483	1.777221
8	H	2.799934	3.621617	4.303544	5.956415	6.543490
9	C	2.043202	3.336952	3.855950	5.635083	6.188830
10	C	2.032456	2.833738	3.853033	5.635138	5.957630
11	H	2.812455	4.854474	4.260084	5.869420	6.682015
12	H	2.846267	2.802027	4.386194	6.051522	6.224756
13	C	2.037953	3.431635	3.833413	5.595763	5.905597
14	H	2.791551	3.784081	4.263733	5.884212	6.026347
15	C	2.055581	4.134250	3.824528	5.570709	6.103614
16	H	2.805373	4.928590	4.233401	5.822492	6.372588
17	C	2.058189	4.085731	3.837201	5.593629	6.273112
18	H	4.414705	2.811622	5.048535	6.195082	6.107245
19	C	4.056370	2.063030	4.297445	5.260157	5.094621
20	C	4.094042	2.049172	3.854685	4.526118	4.459252
21	H	4.456331	2.809434	4.682359	5.572965	5.127754
22	H	4.498150	2.804045	4.332231	4.984968	5.082577
23	C	4.142605	2.034647	3.271932	3.476826	3.196692
24	H	4.593878	2.792444	3.316299	3.004376	2.754741
25	C	4.122991	2.032375	3.422546	3.756194	3.220852
26	H	4.542114	2.776900	3.573899	3.570056	2.793761
27	C	4.065902	2.049759	4.062473	4.873536	4.485392

		6	7	8	9	10
6	H	0.000000				
7	H	1.772233	0.000000			
8	H	6.032003	6.578813	0.000000		
9	C	5.901528	6.175897	1.079365	0.000000	
10	C	6.077487	6.238773	2.238359	1.429939	0.000000

11	H	6.133922	6.051401	2.681640	2.226769	3.348925
12	H	6.450043	6.777890	2.701135	2.245158	1.080219
13	C	6.246104	5.995142	3.346313	2.302985	1.430844
14	H	6.654466	6.252858	4.356317	3.346199	2.239423
15	C	6.178178	5.776387	3.340121	2.297005	2.306294
16	H	6.517823	5.832358	4.348306	3.339042	3.347980
17	C	5.963863	5.891132	2.228676	1.416320	2.307129
18	H	6.327972	7.179065	4.080332	4.192271	3.488161
19	C	5.401471	6.272336	4.301130	4.321270	3.633989
20	C	4.417713	5.593101	4.331801	4.527261	4.232591
21	H	6.087268	6.426762	5.596907	5.196059	4.022487
22	H	4.671294	6.031207	4.165377	4.607472	4.609092
23	C	3.444267	4.560534	5.170535	5.163164	4.796270
24	H	2.718176	4.077600	5.703633	5.745136	5.565950
25	C	4.102583	4.732812	5.589158	5.337162	4.615563
26	H	4.087934	4.417534	6.396543	6.024721	5.254609
27	C	5.249475	5.819446	5.110836	4.848845	3.899646
		11	12	13	14	15
11	H	0.000000				
12	H	4.358761	0.000000			
13	C	3.340610	2.245624	0.000000		
14	H	4.349667	2.701981	1.079242	0.000000	
15	C	2.234393	3.348871	1.415971	2.228760	0.000000
16	H	2.691430	4.357755	2.225699	2.680808	1.079171
17	C	1.079093	3.349791	2.298570	3.341480	1.421792
18	H	6.337261	2.603939	4.634465	4.901948	5.693496
19	C	6.302425	2.952328	4.612548	4.838945	5.614156
20	C	6.237095	3.825779	5.215055	5.606176	5.942938
21	H	7.034457	3.377836	4.396002	4.099074	5.646146
22	H	6.236422	4.256005	5.733172	6.273708	6.312132
23	C	6.586243	4.560768	5.457298	5.712192	6.119428
24	H	6.894652	5.464143	6.156153	6.462720	6.636157
25	C	6.838577	4.309280	5.031059	5.022159	5.898244
26	H	7.327972	5.052214	5.407372	5.248830	6.226135
27	C	6.667121	3.326956	4.471993	4.412237	5.573333
		16	17	18	19	20
16	H	0.000000				
17	C	2.233792	0.000000			
18	H	6.685841	5.478321	0.000000		
19	C	6.537269	5.469354	1.080591	0.000000	
20	C	6.833944	5.583810	2.222106	1.413833	0.000000
21	H	6.363106	6.051343	2.691646	2.235195	3.343883
22	H	7.243477	5.714367	2.676245	2.227524	1.079202
23	C	6.855322	5.959136	3.333410	2.290708	1.422949
24	H	7.294904	6.403677	4.341280	3.332945	2.233301
25	C	6.559283	6.061567	3.334335	2.290302	2.298861

	26 H 6.739129 6.566060 4.343026 3.333001 3.342401<br>27 C 6.349614 5.764566 2.231139 1.419560 2.301192<br>21 22 23 24 25<br>21 H 0.000000<br>22 H 4.352067 0.000000<br>23 C 3.342851 2.236427 0.000000<br>24 H 4.352419 2.691857 1.078856 0.000000<br>25 C 2.232997 3.342129 1.419636 2.233180 0.000000<br>26 H 2.685120 4.352457 2.232893 2.692162 1.079554<br>27 C 1.079326 3.343340 2.299861 3.342770 1.419737<br>26 27<br>26 H 0.000000<br>27 C 2.228972 0.000000

Table S2. Distance matrix for the lowest energy $Cp_2Fe_2PMe(CO)$ structures as optimized at the PBE0/def2TZVP level. Included are the ranking order, spin multiplicity (S, T or Q), total energy (in a.u.), relative energy (in kcal/mol), symmetry point group Wiberg bond indices for the Fe-Fe bonds, Mulliken charges and the spin density (Cp moieties are omitted for clarity).

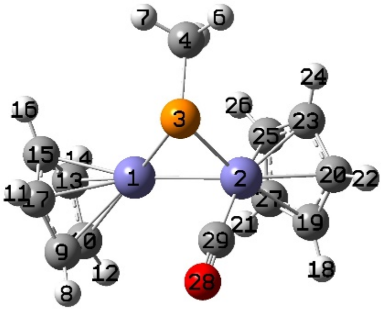

1Q. -3407.950753 0.00 C1
WBI 0.34

Charge and spin density

		1	2
1	Fe	0.417041	3.522624
2	Fe	0.072828	0.755765
3	P	-0.160424	-0.298015
4	C	-0.048046	0.007556
9	C	-0.041725	0.044899
10	C	-0.051302	0.016997
13	C	-0.090851	0.024199
15	C	-0.036550	0.012446
17	C	0.004764	0.037853
19	C	0.002333	-0.003742
20	C	-0.025729	0.018142
23	C	0.021856	-0.033431
25	C	-0.021333	0.016155
27	C	-0.017259	-0.053032
28	O	-0.122561	-0.018886
29	C	0.096956	-0.049531

		1	2	3	4	5
1	Fe	0.000000				
2	Fe	2.454168	0.000000			
3	P	2.323958	2.187623	0.000000		
4	C	3.427464	3.351842	1.863692	0.000000	
5	H	3.456226	3.379650	2.521622	1.090889	0.000000
6	H	4.364466	3.771238	2.441873	1.093961	1.771869
7	H	3.783116	4.257276	2.449844	1.092837	1.775303
8	H	3.035487	4.670259	4.781459	6.268896	6.463766
9	C	2.315495	4.369769	4.308995	5.577715	5.684766
10	C	2.258956	4.241239	4.573194	5.552876	5.383731
11	H	3.004278	5.100734	4.007523	5.103240	5.532928
12	H	2.982894	4.482694	5.258246	6.257622	5.986701
13	C	2.216073	4.434071	4.346252	4.874662	4.594704
14	H	2.933032	4.839982	4.893030	5.088369	4.588542
15	C	2.222164	4.645098	3.883823	4.412574	4.405035
16	H	2.932418	5.190546	4.075175	4.197543	4.206171
17	C	2.290732	4.612126	3.867621	4.909976	5.139578
18	H	4.690806	2.879750	5.044849	6.114326	5.921206
19	C	4.110622	2.132179	4.302414	5.167302	4.903923
20	C	4.477429	2.099775	3.910165	4.532525	4.367428
21	H	3.464353	2.922969	4.706751	5.366502	4.831175
22	H	5.295457	2.842715	4.410002	5.080112	5.068898
23	C	4.068906	2.089059	3.372951	3.467111	3.108119
24	H	4.632643	2.832630	3.473991	3.107396	2.798940
25	C	3.352970	2.112355	3.491102	3.600068	2.979434
26	H	3.381751	2.868201	3.692151	3.384466	2.522908
27	C	3.399125	2.154968	4.087775	4.704277	4.223873
28	O	3.463511	2.902240	3.703505	5.512358	5.885606
29	C	2.733109	1.755876	2.833548	4.540824	4.826133

		6	7	8	9	10
6	H	0.000000				
7	H	1.760041	0.000000			
8	H	7.141817	6.442622	0.000000		
9	C	6.531595	5.689153	1.080168	0.000000	
10	C	6.550164	5.764427	2.229130	1.417119	0.000000
11	H	6.007319	4.844516	2.674945	2.220426	3.330511
12	H	7.207066	6.593060	2.683064	2.228920	1.080259
13	C	5.941666	4.930863	3.339588	2.296459	1.418854
14	H	6.140768	5.165234	4.350182	3.340379	2.233626

15	C	5.487749	4.215944	3.332867	2.288897	2.289891
16	H	5.263516	3.762639	4.340970	3.331038	3.335098
17	C	5.897099	4.780588	2.219563	1.406518	2.286906
18	H	6.384430	7.081643	5.803734	5.838034	5.458071
19	C	5.434129	6.161238	5.774605	5.623817	5.170208
20	C	4.536329	5.601913	6.543152	6.329790	6.013256
21	H	5.990150	6.159622	5.049161	4.658824	3.723520
22	H	4.888204	6.147617	7.191464	7.082320	6.906562
23	C	3.505669	4.543392	6.644904	6.209600	5.782278
24	H	2.860641	4.176613	7.372311	6.882819	6.525368
25	C	4.021694	4.543591	5.954283	5.398992	4.722222
26	H	3.943495	4.180181	6.159009	5.441520	4.641347
27	C	5.188744	5.609305	5.377094	4.999504	4.272233
28	O	5.945626	6.119569	3.412500	3.832194	4.421593
29	C	4.983037	5.247051	3.670716	3.769457	4.092984
		11	12	13	14	15
11	H	0.000000				
12	H	4.339051	0.000000			
13	C	3.340259	2.232041	0.000000		
14	H	4.351686	2.691608	1.079963	0.000000	
15	C	2.231733	3.334902	1.419241	2.234241	0.000000
16	H	2.685737	4.347636	2.233484	2.694325	1.079946
17	C	1.079494	3.329446	2.297799	3.342342	1.421003
18	H	7.266929	5.098055	6.116222	6.374372	6.793143
19	C	6.888164	4.951190	5.595369	5.778380	6.227628
20	C	7.199248	5.995828	6.219723	6.400749	6.622361
21	H	6.289199	3.303987	4.065983	4.012818	5.087524
22	H	7.826699	6.904860	7.171592	7.417191	7.472968
23	C	6.875027	5.912467	5.662123	5.706484	6.006080
24	H	7.258836	6.770203	6.236766	6.251688	6.413351
25	C	6.327669	4.782785	4.538292	4.451430	5.118742
26	H	6.250623	4.795907	4.151504	3.870668	4.742370
27	C	6.350304	4.061537	4.497206	4.516218	5.291747
28	O	4.638164	4.599131	5.284450	6.095563	5.291740
29	C	4.576739	4.314751	4.734847	5.431186	4.811207
		16	17	18	19	20
16	H	0.000000				
17	C	2.232256	0.000000			
18	H	7.555437	6.640683	0.000000		
19	C	6.887674	6.245188	1.079679	0.000000	
20	C	7.114335	6.690194	2.227161	1.417062	0.000000
21	H	5.810962	5.392326	2.684075	2.229766	3.341432
22	H	7.949620	7.424157	2.679288	2.227875	1.079185
23	C	6.326024	6.336515	3.335482	2.292636	1.420025
24	H	6.568232	6.815561	4.346426	3.336662	2.234637
25	C	5.507120	5.615061	3.330823	2.287317	2.294070

		21	22	23	24	25
26	H	4.984375	5.493861	4.340342	3.330418	3.337561
27	C	5.920119	5.564836	2.227105	1.416100	2.298800
28	O	6.100242	4.454393	3.651614	3.788399	4.211141
29	C	5.548011	4.254955	3.086782	2.934968	3.255219

		21	22	23	24	25
21	H	0.000000				
22	H	4.348553	0.000000			
23	C	3.343625	2.235463	0.000000		
24	H	4.351700	2.698012	1.078799	0.000000	
25	C	2.232908	3.338836	1.419434	2.230571	0.000000
26	H	2.687312	4.349771	2.231112	2.685667	1.079471
27	C	1.079129	3.340189	2.300164	3.341813	1.418175
28	O	4.545491	4.453664	4.854324	5.565505	4.869645
29	C	3.732463	3.655195	3.745799	4.464832	3.758050

		26	27	28	29
26	H	0.000000			
27	C	2.228454	0.000000		
28	O	5.594019	4.258707	0.000000	
29	C	4.487515	3.297543	1.147382	0.000000

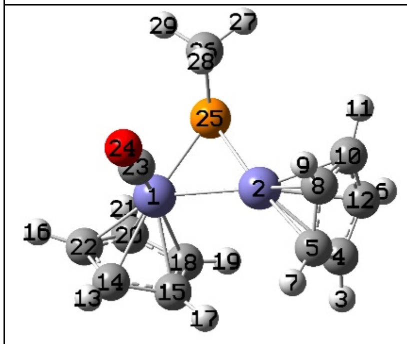

2Q. -3407.949382 0.86 C1
WBI 0.35

Charge and spin density

		1	2
1	Fe	0.070024	0.869417
2	Fe	0.445888	3.523988
4	C	-0.057794	0.054317
5	C	-0.045635	0.029857
8	C	-0.058938	0.027307
10	C	-0.040066	0.010243
12	C	-0.018627	0.020249
14	C	0.015023	0.010752
15	C	-0.033620	-0.069063
18	C	0.059256	0.021390
20	C	0.027674	-0.022197
22	C	-0.068099	0.011975

		1	2	3	4	5
1	Fe	0.000000				
2	Fe	2.432371	0.000000			
3	H	4.722637	3.058403	0.000000		
4	C	4.386122	2.324576	1.080668	0.000000	
5	C	4.282577	2.292161	2.228682	1.415809	0.000000
6	H	5.060020	2.984879	2.679489	2.224183	3.332611
7	H	4.551856	3.020242	2.685186	2.229652	1.080375
8	C	4.432156	2.232770	3.336791	2.293016	1.415488
9	H	4.823751	2.940358	4.346537	3.336616	2.229017
10	C	4.600948	2.208558	3.334690	2.289859	2.289819
11	H	5.125025	2.913251	4.343235	3.332203	3.334417
12	C	4.576810	2.271489	2.221818	1.408875	2.288404
13	H	2.869139	4.489260	5.628238	5.614987	5.208796
14	C	2.124482	3.912129	5.107359	5.127619	4.961484
15	C	2.152584	3.112467	3.772117	3.915972	4.020043
16	H	2.847871	5.250721	6.987240	6.925746	6.835341
17	H	2.932988	3.121667	3.050675	3.308425	3.368704
18	C	2.134876	3.166383	3.991643	4.145618	4.607108
19	H	2.893441	3.198866	3.530086	3.785155	4.559744
20	C	2.114094	3.991452	5.368651	5.413288	5.730093
21	H	2.847671	4.609133	6.063497	6.092546	6.525893
22	C	2.101764	4.379293	5.927842	5.912152	5.910448
23	C	1.740488	2.879139	5.516350	4.941269	4.337219
24	O	2.889347	3.642958	6.259524	5.590652	4.745444
25	P	2.208691	2.343588	5.028091	4.456062	4.628843
26	C	3.410431	3.266810	6.240846	5.433527	5.244754

23	C	0.040205	-0.070989
24	O	-0.127979	-0.021608
25	P	-0.195090	-0.402180
26	C	-0.012224	0.006542

27	H	4.324805	3.713196	6.478083	5.621687	5.548540
28	H	3.496763	3.174145	6.184137	5.299190	4.832852
29	H	3.769692	4.195358	7.188906	6.435017	6.232626
		6	7	8	9	10
6	H	0.000000				
7	H	4.342646	0.000000			
8	C	3.340618	2.227055	0.000000		
9	H	4.352473	2.682776	1.079934	0.000000	
10	C	2.233465	3.333959	1.419301	2.234167	0.000000
11	H	2.687907	4.345517	2.233834	2.694834	1.079710
12	C	1.079592	3.331942	2.297759	3.342715	1.422556
13	H	7.073580	4.845292	5.849792	6.083592	6.544103
14	C	6.350978	4.800302	5.588286	5.972241	6.066795
15	C	5.145605	3.987605	4.821383	5.421755	5.161913
16	H	7.677317	6.830417	7.174714	7.469538	7.442143
17	H	4.899987	3.185883	4.409154	5.053463	4.891682
18	C	4.742019	4.869226	5.226354	5.942767	5.181907
19	H	4.034436	4.981805	5.189258	6.041481	4.907490
20	C	5.826671	5.972434	6.155371	6.733605	6.102655
21	H	6.147564	6.877773	6.833607	7.429165	6.595196
22	C	6.707960	5.938564	6.347133	6.746778	6.585968
23	C	5.837238	4.462306	4.166543	4.152754	4.681460
24	O	6.572316	4.766613	4.394391	4.089902	5.092105
25	P	4.135188	5.325201	4.264896	4.720587	3.800383
26	C	5.091086	5.934428	4.413132	4.478751	4.045562
27	H	4.899175	6.363175	4.569484	4.665277	3.927927
28	H	5.314127	5.418789	3.901815	3.743320	3.846092
29	H	6.092584	6.851083	5.448851	5.446833	5.137610
		11	12	13	14	15
11	H	0.000000				
12	C	2.233807	0.000000			
13	H	7.311740	6.415094	0.000000		
14	C	6.809507	5.809807	1.079496	0.000000	
15	C	5.994625	4.663440	2.228145	1.416689	0.000000
16	H	7.961143	7.296689	2.681908	2.229446	3.340390
17	H	5.841232	4.319269	2.685063	2.229884	1.079264
18	C	5.876523	4.538465	3.336428	2.292492	1.423586
19	H	5.583737	4.037302	4.348161	3.337039	2.237190
20	C	6.651568	5.653086	3.337138	2.294052	2.298104
21	H	7.018336	6.132705	4.348565	3.338389	3.339633
22	C	7.177979	6.332148	2.229629	1.419133	2.298980
23	C	5.098454	5.129819	3.123932	2.960810	3.360345
24	O	5.421317	5.775407	3.731477	3.843478	4.351283
25	P	3.906560	3.940883	5.073990	4.291523	3.937841
26	C	3.774254	4.759952	6.035735	5.494592	5.386286
27	H	3.389572	4.705946	7.020457	6.435862	6.169628

28	H	3.642987	4.776302	5.797956	5.430901	5.393407
29	H	4.850360	5.807956	6.285082	5.767936	5.881408

		16	17	18	19	20
16	H	0.000000				
17	H	4.348905	0.000000			
18	C	3.338370	2.238173	0.000000		
19	H	4.347789	2.698760	1.079883	0.000000	
20	C	2.236892	3.341105	1.413654	2.223948	0.000000
21	H	2.700093	4.348875	2.222482	2.672204	1.079095
22	C	1.079144	3.341694	2.294216	3.336985	1.420701
23	C	3.600928	3.846082	3.800551	4.541109	3.737660
24	O	4.400006	4.703689	4.925831	5.660762	4.849251
25	P	4.560990	4.525743	3.306030	3.412283	3.340074
26	C	5.595024	5.881795	5.035349	5.235616	4.947140
27	H	6.597293	6.586292	5.717644	5.736328	5.740380
28	H	5.760121	5.768654	5.329115	5.630548	5.345126
29	H	5.445456	6.512440	5.474183	5.778811	5.100286

		21	22	23	24	25
21	H	0.000000				
22	C	2.235384	0.000000			
23	C	4.423380	3.226365	0.000000		
24	O	5.516273	4.192845	1.149090	0.000000	
25	P	3.449868	3.967894	2.955275	3.837403	0.000000
26	C	5.048299	5.233348	3.151871	3.508819	1.865421
27	H	5.753340	6.186160	4.171174	4.483139	2.446417
28	H	5.634508	5.400652	2.728267	2.786291	2.532774
29	H	5.064873	5.288164	3.452746	3.727984	2.440712

		26	27	28	29
26	C	0.000000			
27	H	1.092566	0.000000		
28	H	1.091051	1.776403	0.000000	
29	H	1.093806	1.761968	1.765869	0.000000

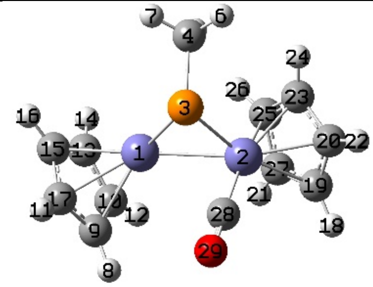

3T. -3407.947629 1.96 C1
WBI 0.25

Charge and spin density

		1	2
1	Fe	0.433845	3.648181

		1	2	3	4	5
1	Fe	0.000000				
2	Fe	2.614273	0.000000			
3	P	2.313993	2.258455	0.000000		
4	C	3.348818	3.368925	1.871475	0.000000	
5	H	3.345546	3.401417	2.534715	1.091276	0.000000
6	H	4.300012	3.748047	2.456597	1.093472	1.772788
7	H	3.703055	4.296366	2.446874	1.093332	1.771964
8	H	3.047910	4.456133	4.922822	6.344585	6.363323
9	C	2.322456	4.262154	4.445125	5.658366	5.613628
10	C	2.266222	4.134922	4.561571	5.392765	5.079057
11	H	3.017228	5.221097	4.422555	5.665485	5.957143
12	H	2.991701	4.260586	5.151406	5.920044	5.476213
13	C	2.216630	4.519926	4.364497	4.795749	4.432639

2	Fe	0.209696	-1.896520
3	P	-0.227126	-0.072549
4	C	-0.054325	0.013664
9	C	-0.021421	0.043078
10	C	-0.053385	0.008615
13	C	-0.088269	0.021717
15	C	-0.030777	0.018107
17	C	-0.037357	0.042609
19	C	-0.014124	0.021072
20	C	-0.031067	-0.016935
23	C	0.038090	0.027914
25	C	-0.044550	-0.006673
27	C	-0.036383	0.010185
28	C	0.059137	0.092500
29	O	-0.101984	0.045035

14	H	2.930654	4.961153	4.829470	4.863130	4.296776
15	C	2.226597	4.837609	4.094856	4.695989	4.633332
16	H	2.933941	5.487250	4.342925	4.657828	4.665074
17	C	2.300951	4.696450	4.158535	5.265193	5.373917
18	H	5.133836	2.946355	5.164307	6.130054	5.968492
19	C	4.488477	2.200242	4.411620	5.174574	4.943679
20	C	4.723589	2.185735	3.957949	4.454629	4.337704
21	H	3.909345	2.944379	4.899812	5.516311	4.987307
22	H	5.511193	2.918274	4.404159	4.937945	4.992433
23	C	4.165038	2.115435	3.381447	3.373296	3.059618
24	H	4.610432	2.849341	3.417069	2.918401	2.662544
25	C	3.519125	2.128175	3.598832	3.655707	3.056072
26	H	3.460208	2.873168	3.819860	3.506521	2.660097
27	C	3.780368	2.199340	4.249537	4.795781	4.330923
28	C	2.915400	1.773999	2.829251	4.523814	4.819691
29	O	3.605042	2.917885	3.635668	5.448255	5.840436

		6	7	8	9	10
6	H	0.000000				
7	H	1.759802	0.000000			
8	H	7.218323	6.667733	0.000000		
9	C	6.612042	5.894563	1.080327	0.000000	
10	C	6.374314	5.697186	2.229510	1.417137	0.000000
11	H	6.605414	5.554565	2.674953	2.221319	3.332278
12	H	6.833307	6.359879	2.684393	2.229532	1.080240
13	C	5.856810	4.886801	3.338945	2.295342	1.418768
14	H	5.904582	4.929591	4.350197	3.339683	2.234227
15	C	5.772522	4.556696	3.331862	2.287945	2.291253
16	H	5.727870	4.271289	4.340667	3.330774	3.336104
17	C	6.265466	5.252037	2.220416	1.407777	2.288708
18	H	6.275723	7.144124	5.662988	5.898746	5.691484
19	C	5.325019	6.208828	5.566592	5.604359	5.255214
20	C	4.348885	5.536980	6.386047	6.296926	5.983253
21	H	6.027353	6.390093	4.627315	4.516826	3.750298
22	H	4.630950	6.000775	7.107199	7.092251	6.914458
23	C	3.330786	4.458926	6.378102	6.051274	5.548786
24	H	2.602342	3.977118	7.124371	6.704233	6.210579
25	C	3.998271	4.632835	5.577979	5.175548	4.437332
26	H	4.002028	4.341348	5.729719	5.149405	4.211176
27	C	5.171497	5.758148	5.034909	4.878123	4.233452
28	C	4.942131	5.245858	3.619483	3.790483	4.233477
29	O	5.866556	6.059581	3.495356	3.924594	4.685188

		11	12	13	14	15
11	H	0.000000				
12	H	4.340953	0.000000			
13	C	3.340881	2.232038	0.000000		
14	H	4.351451	2.692761	1.079897	0.000000	

15	C	2.230851	3.336314	1.420638	2.234486	0.000000
16	H	2.686120	4.348507	2.233999	2.692824	1.080094
17	C	1.079781	3.331420	2.297648	3.341452	1.418966
18	H	7.397883	5.280516	6.557404	6.941105	7.194594
19	C	7.040872	4.916396	5.932690	6.222753	6.595174
20	C	7.402777	5.825449	6.397887	6.624121	6.910612
21	H	6.400394	3.101093	4.532217	4.691952	5.548475
22	H	8.029783	6.805865	7.333283	7.607444	7.727476
23	C	7.069593	5.482530	5.672673	5.734575	6.221226
24	H	7.477668	6.256680	6.118191	6.098514	6.552381
25	C	6.499766	4.241088	4.609131	4.591866	5.402369
26	H	6.439616	4.057605	4.111594	3.876026	5.009600
27	C	6.503581	3.808814	4.828820	4.988235	5.692306
28	C	4.521507	4.485109	4.908911	5.652196	4.917760
29	O	4.418700	4.988191	5.474381	6.339960	5.309166
		16	17	18	19	20
16	H	0.000000				
17	C	2.231180	0.000000			
18	H	8.039572	6.834709	0.000000		
19	C	7.368683	6.417576	1.079959	0.000000	
20	C	7.524429	6.857325	2.224595	1.413403	0.000000
21	H	6.429958	5.539998	2.677205	2.226682	3.338083
22	H	8.300523	7.592135	2.676833	2.224844	1.079643
23	C	6.713209	6.442668	3.328327	2.285283	1.414260
24	H	6.885638	6.903474	4.339308	3.329294	2.229289
25	C	5.999906	5.713533	3.329783	2.287254	2.299318
26	H	5.516124	5.573548	4.342238	3.332684	3.340817
27	C	6.479228	5.719488	2.226611	1.416413	2.296922
28	C	5.656332	4.269001	3.305493	3.114980	3.414262
29	O	6.059057	4.386143	3.960115	4.019276	4.401431
		21	22	23	24	25
21	H	0.000000				
22	H	4.344860	0.000000			
23	C	3.343572	2.229660	0.000000		
24	H	4.351665	2.691666	1.078611	0.000000	
25	C	2.230201	3.344739	1.429457	2.238019	0.000000
26	H	2.694472	4.353267	2.239010	2.690859	1.079629
27	C	1.079603	3.338848	2.297419	3.338210	1.413574
28	C	3.833768	3.815021	3.802788	4.494794	3.809918
29	O	4.688996	4.656830	4.919987	5.593214	4.930971
		26	27	28	29	
26	H	0.000000				
27	C	2.230201	0.000000			
28	C	4.512610	3.420649	0.000000		
29	O	5.617940	4.415019	1.143988	0.000000	

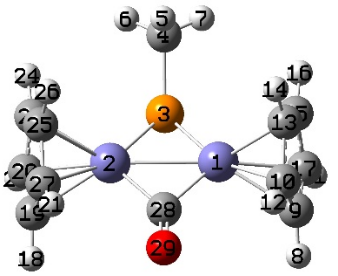

4Q. -3407.939079 7.33 Cs
WBI 0.26

Charge and spin density

		1	2
1	Fe	0.294034	2.535117
2	Fe	0.294034	2.535117
3	P	-0.204095	-0.869540
4	C	0.025840	0.015089
9	C	-0.030385	-0.023558
10	C	-0.041593	-0.025708
13	C	-0.060547	0.042580
15	C	-0.015279	-0.054377
17	C	-0.065546	0.025673
19	C	-0.030385	-0.023558
20	C	-0.065546	0.025673
23	C	-0.015279	-0.054377
25	C	-0.060547	0.042580
27	C	-0.041593	-0.025708
28	C	0.102600	-0.113279
29	O	-0.085712	-0.031724

		1	2	3	4	5
1	Fe	0.000000				
2	Fe	2.535942	0.000000			
3	P	2.181288	2.181288	0.000000		
4	C	3.494737	3.494737	1.849162	0.000000	
5	H	3.609880	3.609880	2.479771	1.090417	0.000000
6	H	4.389013	3.845580	2.439085	1.092696	1.777099
7	H	3.845580	4.389013	2.439085	1.092696	1.777099
8	H	2.900649	4.928156	4.838016	6.248024	6.418904
9	C	2.169574	4.511415	4.183626	5.399978	5.499111
10	C	2.226203	4.386801	4.344874	5.280748	5.093653
11	H	2.841715	5.075422	3.808489	4.843311	5.300662
12	H	2.964801	4.689201	5.082076	6.040616	5.743511
13	C	2.248982	4.426402	3.913524	4.386095	4.088621
14	H	2.985316	4.741181	4.349084	4.459637	3.909706
15	C	2.141000	4.503344	3.345964	3.814232	3.834053
16	H	2.882635	4.949097	3.411600	3.396286	3.460789
17	C	2.125753	4.592930	3.582079	4.582768	4.835050
18	H	4.928156	2.900649	4.838016	6.248024	6.418904
19	C	4.511415	2.169574	4.183626	5.399978	5.499111
20	C	4.592930	2.125753	3.582079	4.582768	4.835050
21	H	4.689201	2.964801	5.082076	6.040616	5.743511
22	H	5.075422	2.841715	3.808489	4.843311	5.300662
23	C	4.503344	2.141000	3.345964	3.814232	3.834053
24	H	4.949097	2.882635	3.411600	3.396286	3.460789
25	C	4.426402	2.248982	3.913524	4.386095	4.088621
26	H	4.741181	2.985316	4.349084	4.459637	3.909706
27	C	4.386801	2.226203	4.344874	5.280748	5.093653
28	C	1.916003	1.916003	3.198544	4.652688	4.629197
29	O	2.897930	2.897930	4.364385	5.802313	5.708428

		6	7	8	9	10
6	H	0.000000				
7	H	1.764610	0.000000			
8	H	7.157705	6.339377	0.000000		
9	C	6.371760	5.450903	1.079636	0.000000	
10	C	6.294510	5.447813	2.232951	1.425492	0.000000
11	H	5.733228	4.534785	2.699529	2.230141	3.339161
12	H	7.013661	6.325137	2.678002	2.231519	1.079593
13	C	5.455733	4.432934	3.335519	2.296240	1.404500
14	H	5.500336	4.562176	4.343934	3.339859	2.217730
15	C	4.887157	3.600311	3.341190	2.293551	2.281754
16	H	4.449538	2.921803	4.350350	3.334846	3.325181
17	C	5.566091	4.409875	2.229530	1.410202	2.291986
18	H	6.339377	7.157705	6.259196	6.307337	6.344425
19	C	5.450903	6.371760	6.307337	6.169624	6.065725
20	C	4.409875	5.566091	6.773774	6.505105	6.487005

21	H	6.325137	7.013661	6.304625	6.090164	5.521716
22	H	4.534785	5.733228	7.136973	6.928046	7.102153
23	C	3.600311	4.887157	7.064339	6.591457	6.330830
24	H	2.921803	4.449538	7.672726	7.105966	6.856639
25	C	4.432934	5.455733	6.855740	6.374544	5.853712
26	H	4.562176	5.500336	7.235675	6.645351	5.921153
27	C	5.447813	6.294510	6.344425	6.065725	5.634216
28	C	5.315637	5.315637	3.405673	3.146668	2.950117
29	O	6.460659	6.460659	3.480012	3.482414	3.205891
		11	12	13	14	15
11	H	0.000000				
12	H	4.346321	0.000000			
13	C	3.343483	2.219728	0.000000		
14	H	4.354012	2.674858	1.080060	0.000000	
15	C	2.238049	3.326005	1.416870	2.229506	0.000000
16	H	2.688887	4.336782	2.231435	2.689838	1.079243
17	C	1.079341	3.332099	2.302822	3.347262	1.430495
18	H	7.136973	6.304625	6.855740	7.235675	7.064339
19	C	6.928046	6.090164	6.374544	6.645351	6.591457
20	C	6.837227	6.717344	6.539714	6.795063	6.519509
21	H	7.428669	5.204598	5.895599	5.915931	6.578465
22	H	6.973568	7.428669	7.151845	7.500957	6.940591
23	C	6.940591	6.578465	6.122382	6.171150	6.189506
24	H	7.192656	7.212668	6.446529	6.432462	6.376927
25	C	7.151845	5.895599	5.731644	5.641762	6.122382
26	H	7.500957	5.915931	5.641762	5.349764	6.171150
27	C	7.102153	5.521716	5.853712	5.921153	6.330830
28	C	4.451299	3.033058	3.523148	4.018023	3.938127
29	O	5.153360	2.914175	4.100017	4.562572	4.728394
		16	17	18	19	20
16	H	0.000000				
17	C	2.239026	0.000000			
18	H	7.672726	6.773774	0.000000		
19	C	7.105966	6.505105	1.079636	0.000000	
20	C	6.835796	6.536496	2.229530	1.410202	0.000000
21	H	7.212668	6.717344	2.678002	2.231519	3.332099
22	H	7.192656	6.837227	2.699529	2.230141	1.079341
23	C	6.376927	6.519509	3.341190	2.293551	1.430495
24	H	6.381840	6.835796	4.350350	3.334846	2.239026
25	C	6.446529	6.539714	3.335519	2.296240	2.302822
26	H	6.432462	6.795063	4.343934	3.339859	3.347262
27	C	6.856639	6.487005	2.232951	1.425492	2.291986
28	C	4.743095	3.764917	3.405673	3.146668	3.764917
29	O	5.639054	4.433520	3.480012	3.482414	4.433520
		21	22	23	24	25
21	H	0.000000				

22	H	4.346321	0.000000			
23	C	3.326005	2.238049	0.000000		
24	H	4.336782	2.688887	1.079243	0.000000	
25	C	2.219728	3.343483	1.416870	2.231435	0.000000
26	H	2.674858	4.354012	2.229506	2.689838	1.080060
27	C	1.079593	3.339161	2.281754	3.325181	1.404500
28	C	3.033058	4.451299	3.938127	4.743095	3.523148
29	O	2.914175	5.153360	4.728394	5.639054	4.100017

		26	27	28	29
26	H	0.000000			
27	C	2.217730	0.000000		
28	C	4.018023	2.950117	0.000000	
29	O	4.562572	3.205891	1.169406	0.000000

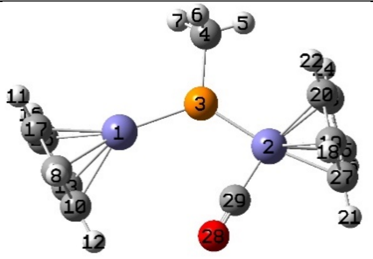

5Q. -3407.938828 7.48 C1
WBI 0.04

Charge and spin density

		1	2
1	Fe	0.389488	3.739396
2	Fe	0.231464	0.507910
3	P	-0.078005	-0.409396
4	C	-0.019742	0.005946
9	C	-0.077187	0.025649
10	C	-0.031659	0.031745
13	C	-0.041333	0.057861
15	C	-0.058452	0.034672
17	C	-0.053833	0.017419
19	C	-0.044387	-0.057235
20	C	-0.010670	0.012554
23	C	0.012811	-0.043754
25	C	-0.056543	0.071105
27	C	-0.004405	-0.008868
28	O	-0.132930	0.010010
29	C	-0.024617	0.004989

		1	2	3	4	5
1	Fe	0.000000				
2	Fe	3.964215	0.000000			
3	P	2.280933	2.134607	0.000000		
4	C	3.462859	3.434022	1.852373	0.000000	
5	H	4.405451	3.407180	2.459126	1.090768	0.000000
6	H	3.776084	3.979477	2.455724	1.092813	1.770927
7	H	3.495316	4.261726	2.461881	1.091872	1.771715
8	H	2.921251	5.997989	4.800628	5.940689	6.933147
9	C	2.211623	5.719238	4.359515	5.511030	6.510025
10	C	2.270478	5.342592	4.279939	5.709170	6.595926
11	H	2.936972	6.778747	4.901439	5.316717	6.401809
12	H	2.980893	5.260622	4.628347	6.253879	7.052038
13	C	2.315465	5.586153	4.290044	5.502576	6.362776
14	H	3.040875	5.712615	4.638835	5.885538	6.626092
15	C	2.281199	6.077995	4.369210	5.153556	6.116002
16	H	3.007065	6.616191	4.798243	5.275765	6.193701
17	C	2.218559	6.158176	4.413869	5.159217	6.211663
18	H	6.075169	2.869204	4.448428	5.303798	5.147386
19	C	5.767183	2.118671	3.906375	4.718740	4.434998
20	C	5.506980	2.105123	3.354987	3.664074	3.190283
21	H	6.497273	2.860521	4.979701	6.205613	5.949163
22	H	5.593760	2.848441	3.486553	3.362620	2.906638
23	C	5.603899	2.102291	3.388416	3.670437	3.004562
24	H	5.791039	2.855622	3.565144	3.393343	2.520758
25	C	5.920152	2.114329	3.957405	4.733546	4.226999
26	H	6.336649	2.856371	4.523642	5.320902	4.798773
27	C	6.005400	2.111998	4.231434	5.267752	4.936204
28	O	3.808631	2.899372	3.661795	5.479018	5.886917
29	C	3.577084	1.751830	2.791756	4.546105	4.839565

		6	7	8	9	10
6	H	0.000000				
7	H	1.767962	0.000000			

8	H	5.830791	6.087292	0.000000		
9	C	5.567030	5.519312	1.080076	0.000000	
10	C	6.012531	5.717150	2.231918	1.418818	0.000000
11	H	5.199534	5.006316	2.695799	2.236188	3.340716
12	H	6.585726	6.396477	2.687812	2.231144	1.080119
13	C	5.972188	5.269670	3.334868	2.291441	1.412570
14	H	6.509945	5.614243	4.344567	3.334885	2.224528
15	C	5.492508	4.736809	3.339324	2.294630	2.289570
16	H	5.674509	4.650781	4.351117	3.339259	3.333003
17	C	5.222013	4.908449	2.236248	1.422257	2.296346
18	H	5.328229	6.355425	7.198828	7.332617	7.234021
19	C	4.929630	5.736394	7.307144	7.282067	7.070514
20	C	3.898677	4.687492	7.420267	7.289557	7.182456
21	H	6.671257	7.064656	7.971344	7.851023	7.256679
22	H	3.325668	4.426384	7.410220	7.342193	7.437125
23	C	4.254862	4.503171	7.898479	7.581179	7.271386
24	H	4.083903	4.066277	8.308970	7.898267	7.615678
25	C	5.391888	5.499447	8.080008	7.751690	7.217898
26	H	6.109729	5.937409	8.610836	8.182177	7.493417
27	C	5.728972	6.158735	7.720122	7.562726	7.082815
28	O	5.853989	6.096687	4.587084	4.482899	3.815580
29	C	4.985762	5.238608	4.979234	4.783743	4.229066
		11	12	13	14	15
11	H	0.000000				
12	H	4.351819	0.000000			
13	C	3.336381	2.226186	0.000000		
14	H	4.346440	2.679886	1.080163	0.000000	
15	C	2.232848	3.333029	1.413219	2.225730	0.000000
16	H	2.689566	4.342398	2.226365	2.680799	1.080290
17	C	1.080083	3.340392	2.292690	3.336217	1.418722
18	H	8.490038	6.969968	7.877128	8.179591	8.333469
19	C	8.361642	6.863804	7.534849	7.737139	7.995806
20	C	8.014291	7.181120	7.445274	7.664526	7.699227
21	H	9.315073	6.785065	7.700999	7.650530	8.502868
22	H	7.822865	7.549831	7.710896	8.046836	7.781514
23	C	8.280627	7.287790	7.299534	7.332769	7.617437
24	H	8.349346	7.756256	7.460425	7.456277	7.646409
25	C	8.776324	7.043812	7.303167	7.200286	7.872146
26	H	9.237033	7.291501	7.441410	7.181255	8.096104
27	C	8.813987	6.765281	7.437569	7.444808	8.089201
28	O	6.338446	3.212567	4.601400	4.776251	5.546731
29	C	6.338496	3.875923	4.781892	4.940052	5.558355
		16	17	18	19	20
16	H	0.000000				
17	C	2.231361	0.000000			
18	H	9.014649	8.024455	0.000000		

19	C	8.589272	7.853754	1.078830	0.000000	
20	C	8.147991	7.609686	2.228385	1.412919	0.000000
21	H	9.137181	8.592661	2.691822	2.235214	3.332781
22	H	8.196621	7.560084	2.683941	2.224303	1.079345
23	C	7.944614	7.788994	3.340876	2.297005	1.421475
24	H	7.828750	7.916277	4.350479	3.339646	2.234916
25	C	8.280073	8.137348	3.341715	2.300149	2.296236
26	H	8.438002	8.531152	4.350495	3.343018	3.340591
27	C	8.656893	8.165438	2.236952	1.424825	2.290005
28	O	6.415125	5.492192	4.074432	3.990253	4.705474
29	C	6.310462	5.564575	3.382841	3.083706	3.632426

		21	22	23	24	25
21	H	0.000000				
22	H	4.343350	0.000000			
23	C	3.332832	2.232393	0.000000		
24	H	4.342061	2.690446	1.079266	0.000000	
25	C	2.224169	3.338957	1.418957	2.231082	0.000000
26	H	2.677461	4.350116	2.233188	2.690339	1.079569
27	C	1.079592	3.334011	2.289484	3.332358	1.412498
28	O	3.657373	5.328295	4.937098	5.721694	4.417290
29	C	3.072960	4.292474	3.806779	4.580242	3.405059

		26	27	28	29
26	H	0.000000			
27	C	2.224365	0.000000		
28	O	4.829196	3.771065	0.000000	
29	C	3.915221	2.907392	1.148195	0.000000

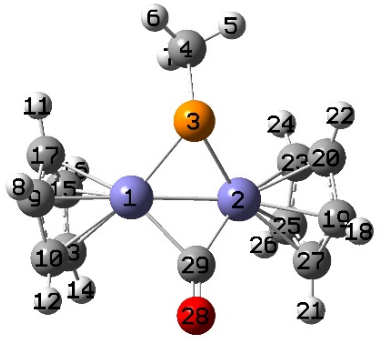

6Q. -3407.937611 8.25 Cs
WBI 0.34

Charge and spin density

		1	2
1	Fe	0.297673	1.935527
2	Fe	0.335393	3.107061
3	P	-0.179137	-0.594912
4	C	0.063950	0.009499
9	C	-0.039957	-0.004778

		1	2	3	4	5
1	Fe	0.000000				
2	Fe	2.415425	0.000000			
3	P	2.185237	2.253777	0.000000		
4	C	3.458675	3.437147	1.853746	0.000000	
5	H	4.329899	3.753505	2.443310	1.093236	0.000000
6	H	3.905851	4.367534	2.434216	1.093349	1.761835
7	H	3.504079	3.520765	2.496180	1.089988	1.776731
8	H	2.836602	5.200800	4.146795	5.085516	6.099262
9	C	2.120661	4.534525	3.803697	4.694191	5.733983
10	C	2.190453	4.322546	4.317939	5.417590	6.392249
11	H	2.866779	4.780427	3.528256	3.435240	4.522522
12	H	2.919251	4.822813	4.998571	6.297903	7.226723
13	C	2.268969	3.932921	4.340780	5.153816	6.091165
14	H	3.021191	4.133475	5.026082	5.859137	6.710938
15	C	2.262785	3.928948	3.861516	4.203682	5.193286
16	H	3.008290	4.120876	4.210795	4.166123	5.068456
17	C	2.135094	4.274447	3.448442	3.810660	4.891046
18	H	4.993337	2.892327	4.284211	5.490690	5.305258
19	C	4.498169	2.165535	3.866970	4.889902	4.743251

10	C	0.073133	0.020780
13	C	-0.103454	0.009718
15	C	-0.076207	-0.013632
17	C	-0.083696	0.009767
19	C	0.138250	0.004114
20	C	-0.044559	-0.028262
23	C	-0.052717	0.022377
25	C	-0.073169	-0.054117
27	C	-0.046317	0.048975
28	O	-0.143343	-0.119415
29	C	-0.065844	-0.352702

20	C	4.416320	2.123329	3.312760	3.848465	3.533178
21	H	4.787140	2.944090	5.145506	6.301052	6.426618
22	H	4.883231	2.854691	3.318915	3.613354	3.041572
23	C	4.286221	2.179436	3.627512	3.818927	3.679330
24	H	4.633226	2.915304	3.865111	3.528806	3.333309
25	C	4.283228	2.243483	4.286229	4.839135	4.905150
26	H	4.584211	2.977564	4.957017	5.386008	5.565535
27	C	4.382465	2.205673	4.389612	5.382763	5.427136
28	O	2.949938	2.912743	4.195648	5.844450	6.390483
29	C	2.020319	1.854865	3.118956	4.701564	5.257984
		6	7	8	9	10
6	H	0.000000				
7	H	1.778019	0.000000			
8	H	4.897368	5.252029	0.000000		
9	C	4.688405	4.678955	1.079778	0.000000	
10	C	5.652376	5.290707	2.230164	1.411554	0.000000
11	H	3.191561	3.209205	2.694230	2.239442	3.334551
12	H	6.547825	6.253087	2.695315	2.228704	1.079839
13	C	5.560030	4.768214	3.338332	2.291982	1.422042
14	H	6.392013	5.398088	4.346300	3.333222	2.229641
15	C	4.524048	3.676083	3.346405	2.304223	2.294202
16	H	4.574333	3.397729	4.357178	3.348376	3.337691
17	C	3.822874	3.560739	2.240458	1.429681	2.292307
18	H	6.392447	5.872656	7.472357	7.023771	6.718777
19	C	5.877362	5.110440	7.172960	6.600657	6.281731
20	C	4.883159	4.040469	7.101566	6.511923	6.431277
21	H	7.285771	6.252890	7.408128	6.686197	5.916871
22	H	4.560226	4.009727	7.375570	6.897161	7.012164
23	C	4.911795	3.609620	7.065578	6.307793	6.136515
24	H	4.592865	3.114569	7.281761	6.501695	6.469583
25	C	5.902717	4.548605	7.108832	6.267008	5.783727
26	H	6.415552	4.902126	7.315774	6.378972	5.769176
27	C	6.404667	5.344579	7.142213	6.417221	5.844929
28	O	6.504730	5.966742	4.813287	4.288000	3.427261
29	C	5.401770	4.810848	4.380841	3.767038	3.162955
		11	12	13	14	15
11	H	0.000000				
12	H	4.349402	0.000000			
13	C	3.325003	2.231107	0.000000		
14	H	4.336853	2.677947	1.080233	0.000000	
15	C	2.232395	3.334218	1.403854	2.218065	0.000000
16	H	2.690544	4.342676	2.217978	2.673639	1.079609
17	C	1.079438	3.339333	2.281938	3.326782	1.418943
18	H	7.498624	6.890623	6.558723	6.578487	6.782846
19	C	6.895789	6.579863	5.924586	5.908023	6.051044
20	C	6.339657	6.938397	5.961348	6.083223	5.753800

21	H	7.408140	6.009126	5.406985	5.007844	5.949297
22	H	6.531117	7.558199	6.648187	6.897962	6.299483
23	C	5.955491	6.753704	5.376856	5.385419	5.072699
24	H	5.780420	7.211902	5.606618	5.679942	5.051165
25	C	6.310922	6.260696	4.939199	4.691511	4.967680
26	H	6.415838	6.261054	4.728443	4.303028	4.800038
27	C	6.847147	6.111465	5.278707	5.033087	5.571991
28	O	5.756399	3.212703	3.610103	3.555860	4.526254
29	C	4.876826	3.321714	3.157218	3.296782	3.764670
		16	17	18	19	20
16	H	0.000000				
17	C	2.231689	0.000000			
18	H	6.992563	7.030727	0.000000		
19	C	6.141896	6.434313	1.079142	0.000000	
20	C	5.684286	6.072521	2.235630	1.419061	0.000000
21	H	6.062648	6.680044	2.680341	2.225795	3.329728
22	H	6.237949	6.421445	2.696890	2.232515	1.078974
23	C	4.785335	5.660366	3.349258	2.303918	1.428846
24	H	4.576221	5.638604	4.358476	3.345926	2.238853
25	C	4.743775	5.789793	3.340223	2.297900	2.290899
26	H	4.447644	5.837399	4.346967	3.339303	3.336214
27	C	5.598124	6.237493	2.227952	1.415390	2.286157
28	O	5.177287	4.860656	3.971276	3.835507	4.670294
29	C	4.340534	4.071408	3.609431	3.266279	3.809879
		21	22	23	24	25
21	H	0.000000				
22	H	4.341135	0.000000			
23	C	3.328795	2.238291	0.000000		
24	H	4.340201	2.692199	1.079454	0.000000	
25	C	2.229347	3.331687	1.405677	2.222404	0.000000
26	H	2.680203	4.343916	2.220009	2.680864	1.080115
27	C	1.079805	3.330321	2.286904	3.331867	1.417743
28	O	3.120527	5.430330	4.823837	5.661813	4.130185
29	C	3.062532	4.533178	3.902223	4.660493	3.433996
		26	27	28	29	
26	H	0.000000				
27	C	2.227499	0.000000			
28	O	4.463202	3.400283	0.000000		
29	C	3.858707	2.961961	1.162421	0.000000	

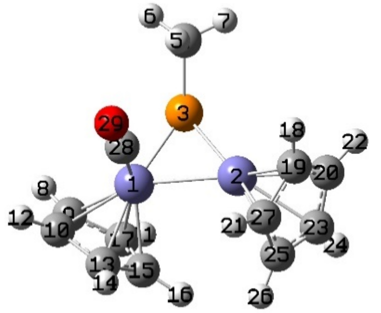

7T. -3407.933637 10.74 C1
WBI 0.32

Charge and spin density

		1	2
1	Fe	0.133084	0.793690
2	Fe	0.420629	1.947585
3	P	-0.220308	-0.616653
4	C	-0.008447	-0.001785
9	C	-0.033357	0.011250
10	C	-0.040219	-0.020481
13	C	-0.051146	0.011080
15	C	0.009667	-0.066021
17	C	0.071338	0.013024
19	C	-0.055302	0.008680
20	C	-0.024654	0.023500
23	C	-0.046886	-0.076735
25	C	-0.033813	0.044780
27	C	-0.015905	-0.016395
28	C	0.016739	-0.049638
29	O	-0.121421	-0.005884

		1	2	3	4	5
1	Fe	0.000000				
2	Fe	2.553370	0.000000			
3	P	2.130545	2.321121	0.000000		
4	C	3.604824	3.499109	1.841996	0.000000	
5	H	3.749068	3.547413	2.453743	1.091838	0.000000
6	H	4.020156	4.441046	2.437804	1.091215	1.777913
7	H	4.429450	3.778680	2.433950	1.091163	1.774964
8	H	2.882426	5.296871	4.016614	5.242551	5.657445
9	C	2.134010	4.547549	3.707898	5.139760	5.456511
10	C	2.115782	4.499654	4.124705	5.398847	5.434408
11	H	2.858354	4.207328	3.655245	5.417443	6.006057
12	H	2.864406	5.226979	4.735767	5.726768	5.627875
13	C	2.107779	3.821756	4.209815	5.659083	5.632312
14	H	2.860113	4.094991	4.882051	6.190951	5.993734
15	C	2.120344	3.404594	3.857545	5.573330	5.773247
16	H	2.883802	3.332528	4.301730	6.049894	6.252066
17	C	2.117844	3.900418	3.507053	5.236890	5.651650
18	H	5.082159	3.000672	4.537476	4.515388	3.979053
19	C	4.582156	2.283869	4.231690	4.641990	4.287062
20	C	4.777988	2.225598	4.133487	4.773130	4.734405
21	H	4.354691	3.048522	5.001396	5.668074	5.134468
22	H	5.428470	2.929745	4.380931	4.784741	4.860780
23	C	4.527343	2.244969	4.370413	5.473062	5.536989
24	H	5.008680	2.961055	4.802009	6.044421	6.268058
25	C	4.138427	2.298628	4.586477	5.742647	5.616677
26	H	4.301086	3.024304	5.153205	6.488619	6.389724
27	C	4.180829	2.326906	4.509535	5.275974	4.901080
28	C	1.742261	2.925259	2.627553	3.220439	2.855323
29	O	2.892863	3.680082	3.466347	3.502012	2.803512

		6	7	8	9	10
6	H	0.000000				
7	H	1.774404	0.000000			
8	H	5.053261	6.124348	0.000000		
9	C	5.173436	6.010790	1.078707	0.000000	
10	C	5.545091	6.348501	2.224074	1.409867	0.000000
11	H	5.636246	5.964805	2.687840	2.230747	3.327329
12	H	5.764088	6.751669	2.681547	2.223378	1.079412
13	C	6.060434	6.487890	3.346352	2.302523	1.431122
14	H	6.678216	7.005715	4.353967	3.343579	2.242385
15	C	6.033759	6.246225	3.347601	2.305580	2.299375
16	H	6.639933	6.584173	4.355995	3.347699	3.344210
17	C	5.484904	5.927653	2.233729	1.420620	2.284971
18	H	5.569792	4.461515	7.917780	7.210153	6.819802
19	C	5.713611	4.641660	7.443252	6.660314	6.322451
20	C	5.843134	4.573904	7.483065	6.731995	6.664546

21	H	6.598901	6.048731	7.128177	6.204962	5.447553
22	H	5.836597	4.343639	8.005699	7.351164	7.425526
23	C	6.475433	5.435884	7.094867	6.244816	6.200457
24	H	6.992096	5.938804	7.310023	6.489618	6.623017
25	C	6.711800	5.943732	6.791226	5.833698	5.497978
26	H	7.388323	6.789074	6.716545	5.693264	5.293408
27	C	6.274726	5.515896	7.021832	6.115088	5.588411
28	C	3.670366	4.164459	3.994751	3.434910	2.873990
29	O	3.904924	4.427675	4.906026	4.437307	3.716688
		11	12	13	14	15
11	H	0.000000				
12	H	4.338724	0.000000			
13	C	3.332016	2.239490	0.000000		
14	H	4.343266	2.695226	1.078964	0.000000	
15	C	2.229597	3.340813	1.414897	2.229149	0.000000
16	H	2.685914	4.352428	2.228905	2.688780	1.079035
17	C	1.079532	3.329706	2.289763	3.334198	1.419957
18	H	7.197999	7.240154	6.122658	5.963660	6.123065
19	C	6.449149	6.869171	5.482281	5.349744	5.329902
20	C	6.052377	7.375920	5.807785	5.863299	5.301527
21	H	6.479863	5.840750	4.425293	3.898448	4.720734
22	H	6.527968	8.144124	6.678904	6.826835	6.088457
23	C	5.344653	7.027425	5.164091	5.221939	4.473328
24	H	5.233449	7.540169	5.590109	5.757093	4.664763
25	C	5.335352	6.242171	4.310220	4.133211	3.907326
26	H	5.191709	6.067350	3.963194	3.695754	3.546028
27	C	6.044236	6.138384	4.551442	4.238575	4.525703
28	C	4.590169	3.048375	2.978193	3.242831	3.578890
29	O	5.735227	3.601581	3.865928	3.898748	4.643371
		16	17	18	19	20
16	H	0.000000				
17	C	2.232856	0.000000			
18	H	5.973757	6.800057	0.000000		
19	C	5.057851	6.087963	1.079905	0.000000	
20	C	4.876596	5.923843	2.234390	1.420468	0.000000
21	H	4.516108	5.816291	2.676797	2.221886	3.332039
22	H	5.690956	6.534645	2.691819	2.233344	1.080299
23	C	3.831633	5.243360	3.343421	2.299341	1.420340
24	H	3.894454	5.324656	4.354448	3.343344	2.234668
25	C	3.292580	4.969481	3.331521	2.287792	2.287367
26	H	2.780962	4.775547	4.339886	3.330437	3.332481
27	C	4.193918	5.535195	2.223030	1.409533	2.287924
28	C	4.249367	3.808350	4.406162	4.244699	4.902152
29	O	5.276447	4.935150	4.362229	4.457292	5.360276
		21	22	23	24	25
21	H	0.000000				

22	H	4.342137	0.000000			
23	C	3.337162	2.232774	0.000000		
24	H	4.347563	2.691664	1.079742	0.000000	
25	C	2.227977	3.331835	1.415085	2.230605	0.000000
26	H	2.680934	4.343969	2.227838	2.687523	1.080350
27	C	1.080116	3.331549	2.294135	3.338021	1.415152
28	C	3.834499	5.564441	5.041131	5.797402	4.487591
29	O	3.933077	5.997844	5.699186	6.564941	5.079804

		26	27	28	29
26	H	0.000000			
27	C	2.226531	0.000000		
28	C	4.840700	3.953617	0.000000	
29	O	5.502219	4.252705	1.151735	0.000000

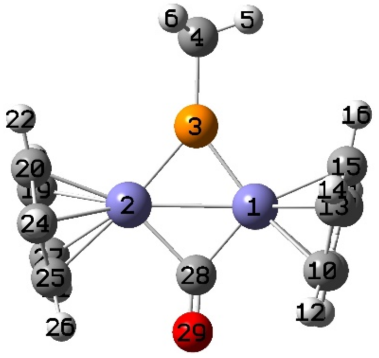

8T. -3407.929403 13.40 Cs
WBI 0.29

Charge and spin density

		1	2
1	Fe	0.192751	0.044472
2	Fe	0.203478	2.249947
3	P	-0.321784	-0.173601
4	C	0.060761	-0.016626
9	C	0.026149	0.021649
10	C	0.024457	0.022614
13	C	-0.024148	-0.019853
15	C	0.032834	0.013418
17	C	-0.018331	-0.020533
19	C	-0.052514	-0.005631
20	C	-0.020350	-0.054044
23	C	-0.042150	0.085778
25	C	0.034484	-0.048688
27	C	-0.051018	0.011590
28	C	0.148085	-0.074236
29	O	-0.192704	-0.036254

		1	2	3	4	5
1	Fe	0.000000				
2	Fe	2.591628	0.000000			
3	P	2.041104	2.121658	0.000000		
4	C	3.659241	3.716455	1.824492	0.000000	
5	H	3.825255	4.514094	2.428551	1.091285	0.000000
6	H	4.275016	4.020265	2.426149	1.091933	1.777786
7	H	4.312332	4.029234	2.424890	1.091939	1.777085
8	H	2.831810	4.640808	4.718172	6.268249	6.272882
9	C	2.080424	4.331309	4.036644	5.494944	5.412578
10	C	2.080084	4.301709	4.040844	5.496634	5.430818
11	H	2.816532	4.910011	3.898781	4.804942	4.494786
12	H	2.832578	4.590636	4.726349	6.272228	6.303347
13	C	2.071335	4.440941	3.557107	4.660623	4.422699
14	H	2.816577	4.830526	3.912882	4.811756	4.562383
15	C	2.054551	4.542914	3.201787	4.040734	3.620399
16	H	2.812577	5.033819	3.312012	3.628688	2.960548
17	C	2.071472	4.486995	3.549264	4.657326	4.386381
18	H	5.041589	2.858768	3.869101	4.578785	5.560255
19	C	4.600586	2.136973	3.630962	4.634625	5.629370
20	C	4.654618	2.159367	3.500778	4.357025	5.395837
21	H	4.782798	2.895013	4.727087	6.162697	6.984943
22	H	5.144629	2.898215	3.649108	4.047029	5.119514
23	C	4.604945	2.253593	3.998826	5.180706	6.124104
24	H	4.992938	2.985572	4.460140	5.544076	6.435745
25	C	4.424443	2.203544	4.303556	5.778213	6.652862
26	H	4.704649	2.935438	4.994936	6.592751	7.390349
27	C	4.461135	2.168337	4.133152	5.514884	6.410005
28	C	1.849727	1.995289	3.045067	4.869182	5.332317
29	O	2.871271	2.913768	4.212162	6.036634	6.492201

		6	7	8	9	10
6	H	0.000000				
7	H	1.773165	0.000000			

	8 H 7.029543 6.728683 0.000000<br>9 C 6.221250 6.068418 1.078931 0.000000<br>10 C 6.008549 6.267286 2.235837 1.427959 0.000000<br>11 H 5.770875 5.127900 2.682897 2.223034 3.340308<br>12 H 6.673781 7.064450 2.685566 2.235832 1.078917<br>13 C 5.104382 5.551907 3.337541 2.297558 1.410695<br>14 H 5.037935 5.812467 4.346570 3.340960 2.223315<br>15 C 4.721528 4.797197 3.335590 2.291129 2.290762<br>16 H 4.302813 4.389489 4.344785 3.333216 3.332927<br>17 C 5.499803 5.183462 2.225828 1.410345 2.296719<br>18 H 4.882562 4.170233 6.704145 6.599662 6.941719<br>19 C 4.836841 4.475259 6.315612 6.199896 6.373076<br>20 C 4.247691 4.416246 6.779856 6.487224 6.352790<br>21 H 6.585563 6.080941 5.443651 5.708148 6.105859<br>22 H 3.725309 4.061554 7.516291 7.109286 6.916658<br>23 C 5.042940 5.462471 6.506573 6.267638 5.934838<br>24 H 5.234986 5.970116 6.985592 6.661146 6.101646<br>25 C 5.880049 6.005145 5.753172 5.735091 5.589037<br>26 H 6.711696 6.907707 5.620049 5.709280 5.480663<br>27 C 5.799774 5.504347 5.654281 5.726496 5.916532<br>28 C 5.343739 5.353632 2.896093 2.752739 2.747953<br>29 O 6.492507 6.493883 2.878850 3.086554 3.093418<br>11 12 13 14 15<br>11 H 0.000000<br>12 H 4.346061 0.000000<br>13 C 3.344776 2.225802 0.000000<br>14 H 4.353997 2.682740 1.078997 0.000000<br>15 C 2.234533 3.335151 1.422393 2.234575 0.000000<br>16 H 2.691113 4.344412 2.233902 2.691236 1.078870<br>17 C 1.079119 3.336782 2.302083 3.344806 1.422556<br>18 H 6.482061 7.326902 7.037129 7.493110 6.752341<br>19 C 6.435756 6.633812 6.541298 6.932793 6.468115<br>20 C 6.904700 6.538050 6.342638 6.512896 6.460063<br>21 H 6.277197 6.204201 6.732718 7.330260 6.748318<br>22 H 7.343424 7.172241 6.698143 6.759820 6.749829<br>23 C 7.133894 5.892553 6.104168 6.206028 6.515408<br>24 H 7.688197 5.949183 6.188773 6.110654 6.779316<br>25 C 6.747481 5.478201 6.065234 6.363086 6.463254<br>26 H 7.023447 5.176302 6.150245 6.444431 6.716891<br>27 C 6.335877 6.012928 6.378812 6.847894 6.473054<br>28 C 4.070000 2.889355 3.442367 4.060501 3.806505<br>29 O 4.725514 2.894358 4.122359 4.739355 4.638532<br>16 17 18 19 20<br>16 H 0.000000<br>17 C 2.234117 0.000000<br>18 H 7.009474 6.484898 0.000000

19	C	6.830890	6.267234	1.079350	0.000000	
20	C	6.757593	6.558341	2.239417	1.431969	0.000000
21	H	7.381525	6.144445	2.699002	2.228945	3.341992
22	H	6.887299	7.015769	2.689629	2.240099	1.079442
23	C	6.979372	6.618725	3.341999	2.302158	1.411498
24	H	7.225099	7.057807	4.352450	3.346665	2.224911
25	C	7.090588	6.281158	3.336207	2.288696	2.277573
26	H	7.460070	6.476804	4.343229	3.328601	3.321532
27	C	7.039404	6.092768	2.228227	1.408498	2.293975
28	C	4.653096	3.448307	4.515262	3.832530	4.037113
29	O	5.583835	4.113853	5.170659	4.428923	4.720357

		21	22	23	24	25
21	H	0.000000				
22	H	4.350969	0.000000			
23	C	3.339491	2.225965	0.000000		
24	H	4.347778	2.683899	1.079977	0.000000	
25	C	2.233822	3.321710	1.407600	2.220820	0.000000
26	H	2.678096	4.332859	2.222418	2.678410	1.079453
27	C	1.079452	3.335059	2.300295	3.343694	1.426193
28	C	3.406324	4.846026	3.616439	4.112930	2.986415
29	O	3.440397	5.639831	4.059411	4.513326	3.120489

		26	27	28	29
26	H	0.000000			
27	C	2.231440	0.000000		
28	C	3.034069	3.185307	0.000000	
29	O	2.779882	3.446448	1.169312	0.000000

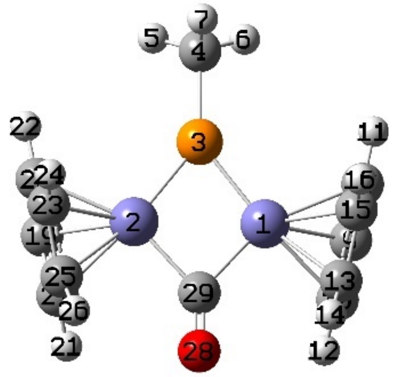

09S -3407.925586 15.79
WBI 0.42

		1	2
1	Fe	0.189169	-0.002118
2	Fe	0.189099	-0.002140
3	P	-0.411756	0.000955
4	C	0.058965	0.001945
9	C	-0.022194	-0.042391
10	C	0.026177	0.012611

		1	2	3	4	5
1	Fe	0.000000				
2	Fe	2.539735	0.000000			
3	P	2.062405	2.062380	0.000000		
4	C	3.675462	3.675383	1.824033	0.000000	
5	H	4.425225	3.882803	2.429142	1.091162	0.000000
6	H	3.881487	4.426727	2.429310	1.091149	1.779031
7	H	4.147809	4.144536	2.419217	1.092381	1.774203
8	H	2.813706	4.828571	3.936667	4.851529	5.501989
9	C	2.071801	4.418547	3.591157	4.705861	5.488138
10	C	2.076643	4.256419	4.060352	5.525253	6.280762
11	H	2.824282	5.012661	3.386160	3.722767	4.716779
12	H	2.822734	4.547397	4.726583	6.283720	6.930159
13	C	2.076831	4.253444	4.062664	5.525842	6.394934
14	H	2.822477	4.541361	4.729775	6.284268	7.123610
15	C	2.071924	4.413498	3.595153	4.706887	5.696718
16	H	2.813837	4.819788	3.943528	4.853378	5.889678
17	C	2.065398	4.509675	3.263989	4.111590	5.071533
18	H	4.828567	2.813647	3.936877	4.851825	4.417991
19	C	4.418592	2.071828	3.591440	4.706198	4.413994

13	C	0.030618	-0.012862
15	C	-0.025676	0.044081
17	C	0.027537	-0.000111
19	C	-0.022264	-0.042438
20	C	0.027514	-0.000114
23	C	-0.025805	0.044134
25	C	0.030679	-0.012885
27	C	0.026221	0.012632
28	O	-0.211174	-0.000270
29	C	0.112892	-0.001026

20	C	4.509855	2.065419	3.264403	4.112061	3.807564
21	H	4.546966	2.822710	4.726495	6.283704	6.256935
22	H	5.012981	2.824280	3.386698	3.723435	3.204989
23	C	4.413521	2.071952	3.595403	4.707182	4.674123
24	H	4.819988	2.813948	3.943906	4.853795	4.898527
25	C	4.253220	2.076872	4.062729	5.525961	5.592183
26	H	4.541023	2.822533	4.729754	6.284302	6.473463
27	C	4.256208	2.076683	4.060433	5.525392	5.459604
28	O	2.873586	2.873664	4.202901	6.026898	6.489664
29	C	1.893443	1.893637	3.029693	4.853689	5.334212

		6	7	8	9	10
6	H	0.000000				
7	H	1.774258	0.000000			
8	H	4.418763	5.610062	0.000000		
9	C	4.412744	5.311145	1.079324	0.000000	
10	C	5.458297	6.053554	2.225289	1.413336	0.000000
11	H	3.199224	4.024115	2.690068	2.233438	3.336391
12	H	6.256982	6.893035	2.685077	2.228617	1.078839
13	C	5.588685	5.806660	3.338397	2.295274	1.424073
14	H	6.469829	6.480259	4.344316	3.335662	2.232237
15	C	4.668656	4.846006	3.340207	2.297327	2.295539
16	H	4.891331	4.746356	4.349380	3.340168	3.338620
17	C	3.803142	4.472978	2.232568	1.420840	2.293930
18	H	5.508570	5.604702	6.062956	6.125793	6.156711
19	C	5.493105	5.305573	6.125869	5.997217	5.881657
20	C	5.075135	4.466057	6.584316	6.322277	6.297483
21	H	6.933733	6.889237	6.127604	5.913990	5.325457
22	H	4.720982	4.016017	6.941441	6.744701	6.916579
23	C	5.697900	4.839875	6.885510	6.413850	6.145660
24	H	5.889435	4.740174	7.448164	6.878804	6.627163
25	C	6.396265	5.801911	6.635846	6.148237	5.613567
26	H	7.123815	6.476287	7.006034	6.403915	5.662704
27	C	6.284141	6.049005	6.156626	5.881489	5.435084
28	O	6.489864	6.469433	4.737021	4.121714	3.083281
29	C	5.334377	5.314739	4.117439	3.506396	2.811099

		11	12	13	14	15
11	H	0.000000				
12	H	4.347336	0.000000			
13	C	3.336030	2.232403	0.000000		
14	H	4.346866	2.682004	1.078827	0.000000	
15	C	2.233133	3.335950	1.413200	2.228360	0.000000
16	H	2.689552	4.344569	2.225241	2.684887	1.079289
17	C	1.078849	3.337785	2.293737	3.337564	1.420983
18	H	6.941206	6.127881	6.635940	7.006193	6.885462
19	C	6.744521	5.914353	6.148409	6.404154	6.413865
20	C	6.625729	6.542999	6.292245	6.532704	6.313411

21	H	7.258816	5.004106	5.668213	5.668217	6.406869
22	H	6.753387	7.259306	6.911388	7.249239	6.735705
23	C	6.735484	6.407215	5.868155	5.896060	5.979418
24	H	6.924806	7.003627	6.136983	6.103866	6.100727
25	C	6.911029	5.668418	5.424785	5.310077	5.867977
26	H	7.248825	5.668331	5.309980	4.983153	5.895794
27	C	6.916243	5.325671	5.613565	5.662787	6.145495
28	O	5.593072	2.872571	3.076321	2.857639	4.112913
29	C	4.711237	2.938329	2.805741	2.927828	3.499062
		16	17	18	19	20
16	H	0.000000				
17	C	2.232700	0.000000			
18	H	7.447971	6.584168	0.000000		
19	C	6.878657	6.322192	1.079326	0.000000	
20	C	6.568159	6.328246	2.232569	1.420837	0.000000
21	H	7.003160	6.542567	2.685047	2.228608	3.337781
22	H	6.924854	6.625838	2.690076	2.233438	1.078849
23	C	6.100545	6.313297	3.340204	2.297322	1.420992
24	H	6.029499	6.568222	4.349383	3.340167	2.232711
25	C	6.136639	6.291972	3.338381	2.295264	2.293746
26	H	6.103440	6.532362	4.344295	3.335651	3.337575
27	C	6.626847	6.297226	2.225280	1.413336	2.293933
28	O	4.722493	4.645334	4.736764	4.121485	4.645188
29	C	4.105581	3.869664	4.117410	3.506430	3.869777
		21	22	23	24	25
21	H	0.000000				
22	H	4.347330	0.000000			
23	C	3.335943	2.233145	0.000000		
24	H	4.344567	2.689568	1.079289	0.000000	
25	C	2.232392	3.336041	1.413205	2.225248	0.000000
26	H	2.681987	4.346880	2.228367	2.684902	1.078827
27	C	1.078838	3.336394	2.295531	3.338615	1.424056
28	O	2.871986	5.592994	4.112699	4.722414	3.075921
29	C	2.938025	4.711401	3.499100	4.105743	2.805618
		26	27	28	29	
26	H	0.000000				
27	C	2.232216	0.000000			
28	O	2.857167	3.082870	0.000000		
29	C	2.927634	2.810973	1.173210	0.000000	

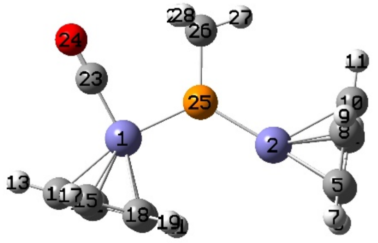

10Q. -3407.924130 16.71 C1
WBI 0.12

Charge and spin density

		1	2
1	Fe	0.267456	1.384961
2	Fe	0.235441	3.282293
4	C	-0.062293	-0.003093
5	C	-0.068542	0.023104
8	C	-0.078338	-0.014908
10	C	-0.007483	0.058284
12	C	-0.087232	-0.009164
14	C	-0.012661	-0.017347
15	C	-0.036396	-0.021257
18	C	-0.054389	-0.016303
20	C	0.013192	-0.019941
22	C	-0.034971	0.028809
23	C	-0.053969	-0.082686
24	O	-0.112530	-0.032848
25	P	0.061821	-0.549694
26	C	0.030893	-0.010210

		1	2	3	4	5
1	Fe	0.000000				
2	Fe	3.865486	0.000000			
3	H	6.064173	2.876798	0.000000		
4	C	5.761123	2.138237	1.080160	0.000000	
5	C	5.735218	2.133579	2.235921	1.419592	0.000000
6	H	6.237159	2.971070	2.690827	2.234912	3.338775
7	H	6.017606	2.870412	2.699504	2.235735	1.080148
8	C	5.846433	2.223274	3.341790	2.295963	1.425059
9	H	6.166248	2.962827	4.353089	3.339646	2.235918
10	C	5.956701	2.298742	3.338750	2.297327	2.297173
11	H	6.338958	3.053663	4.348454	3.340992	3.341069
12	C	5.884890	2.228147	2.236372	1.424097	2.294971
13	H	2.922435	6.626857	8.361850	8.294932	8.298012
14	C	2.181514	5.658923	7.317759	7.264467	7.263241
15	C	2.170846	5.118367	6.887136	6.743706	6.490466
16	H	2.937903	5.595705	6.697417	6.850881	7.282766
17	H	2.907372	5.738124	7.640527	7.419109	6.986335
18	C	2.152398	3.978565	5.509180	5.415688	5.209118
19	H	2.878333	3.619703	5.074777	4.918475	4.490008
20	C	2.160079	3.908614	5.091619	5.163815	5.339839
21	H	2.896119	3.484449	4.180013	4.387173	4.778433
22	C	2.208878	5.048548	6.355249	6.428789	6.670927
23	C	1.765884	4.982718	7.501843	7.045995	6.942033
24	O	2.908651	5.873808	8.496603	7.973056	7.831062
25	P	2.189307	2.179033	4.855715	4.261924	4.243093
26	C	3.418644	3.484604	6.188191	5.437721	5.399581
27	H	4.351381	3.401486	5.955236	5.109887	5.097495
28	H	3.643472	4.135738	6.963412	6.164185	5.904498
29	H	3.579646	4.237625	6.755872	6.103997	6.253046

		6	7	8	9	10
6	H	0.000000				
7	H	4.352070	0.000000			
8	C	3.325491	2.237221	0.000000		
9	H	4.334518	2.692071	1.080112	0.000000	
10	C	2.222183	3.338596	1.409219	2.221920	0.000000
11	H	2.676022	4.348647	2.222268	2.676135	1.080107
12	C	1.080239	3.340718	2.282168	3.325487	1.409609
13	H	8.895064	8.367954	8.617253	8.906292	8.820860
14	C	7.980365	7.315728	7.651404	7.979422	7.896891
15	C	7.765615	6.408049	6.876549	7.078680	7.347732
16	H	7.187035	7.514437	7.794539	8.386204	7.724689
17	H	8.528787	6.821018	7.281863	7.330914	7.875103
18	C	6.597888	5.116642	5.751801	6.071327	6.250791
19	H	6.378534	4.253173	5.085915	5.329506	5.787360
20	C	6.048968	5.426141	5.965719	6.501188	6.185883

21	H	5.264946	4.928875	5.540091	6.220585	5.665219
22	C	7.025712	6.813552	7.180877	7.686444	7.278222
23	C	7.196957	7.315544	6.733019	6.883867	6.729869
24	O	7.965972	8.241999	7.467969	7.525793	7.408853
25	P	4.439916	4.824547	4.011234	4.386702	3.903856
26	C	5.018342	6.124650	4.712000	4.892714	4.306395
27	H	4.447256	5.935271	4.217007	4.404950	3.611988
28	H	5.952349	6.521761	5.129055	5.087180	4.937604
29	H	5.418602	7.010594	5.646352	5.916436	5.084766
		11	12	13	14	15
11	H	0.000000				
12	C	2.222451	0.000000			
13	H	9.252762	8.610107	0.000000		
14	C	8.398028	7.650887	1.079705	0.000000	
15	C	7.904895	7.255441	2.234505	1.424633	0.000000
16	H	8.240400	7.134951	2.678477	2.223705	3.341897
17	H	8.393511	7.938076	2.686523	2.234304	1.079233
18	C	6.925255	6.048457	3.332103	2.289620	1.411501
19	H	6.531422	5.685332	4.345861	3.335977	2.228534
20	C	6.860822	5.708760	3.328550	2.285096	2.295475
21	H	6.413318	4.990354	4.339483	3.329298	3.337217
22	C	7.836227	6.814152	2.224321	1.411407	2.298415
23	C	6.851454	6.902295	3.292111	3.097895	3.252567
24	O	7.387080	7.703712	3.960510	4.009274	4.204485
25	P	4.173553	4.041010	5.107744	4.330065	4.030082
26	C	4.095191	4.780342	6.100149	5.560629	5.393829
27	H	3.200007	4.239778	7.124697	6.523451	6.290310
28	H	4.687771	5.597177	6.136404	5.687411	5.389861
29	H	4.827159	5.376084	5.986555	5.577466	5.683121
		16	17	18	19	20
16	H	0.000000				
17	H	4.348233	0.000000			
18	C	3.340489	2.229696	0.000000		
19	H	4.347272	2.694944	1.079510	0.000000	
20	C	2.226802	3.341453	1.425193	2.232850	0.000000
21	H	2.686496	4.350375	2.235319	2.684381	1.079572
22	C	1.080102	3.339215	2.295900	3.336793	1.413249
23	C	4.047191	3.562692	3.736889	4.373587	3.889146
24	O	4.979625	4.325923	4.826987	5.423305	5.023150
25	P	4.525243	4.621608	3.403353	3.526190	3.354895
26	C	5.777218	5.811027	5.071732	5.223529	5.052627
27	H	6.599105	6.717835	5.803114	5.816109	5.752208
28	H	6.279143	5.607423	5.266461	5.374064	5.502943
29	H	5.556521	6.171281	5.492733	5.828957	5.266489
		21	22	23	24	25
21	H	0.000000				

22	C	2.228400	0.000000			
23	C	4.623569	3.555289	0.000000		
24	O	5.755693	4.585953	1.142835	0.000000	
25	P	3.449227	3.990169	2.878202	3.720767	0.000000
26	C	5.199774	5.394405	3.087506	3.382117	1.843802
27	H	5.731828	6.243310	4.174177	4.442576	2.431703
28	H	5.813553	5.787045	2.942650	2.991643	2.452657
29	H	5.432325	5.357530	3.000237	3.141028	2.458065

		26	27	28	29
26	C	0.000000			
27	H	1.090024	0.000000		
28	H	1.092889	1.774363	0.000000	
29	H	1.092096	1.776853	1.767706	0.000000

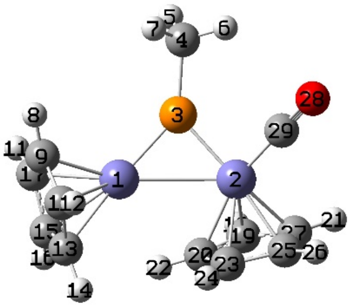

11T. -3407.920435 19.02 C1
WBI 0.22

Charge and spin density

		1	2
1	Fe	0.337764	2.463491
2	Fe	0.228396	0.203007
3	P	-0.229287	-0.598113
4	C	0.042955	-0.016589
9	C	-0.029106	-0.068790
10	C	-0.052719	0.069204
13	C	-0.022262	-0.055275
15	C	-0.054207	0.031124
17	C	-0.074453	-0.018161
19	C	0.019589	0.034641
20	C	0.093450	-0.043743
23	C	-0.072392	0.016761
25	C	0.034101	-0.011984
27	C	-0.039609	-0.041378
28	O	-0.132164	0.015414
29	C	-0.050056	0.020390

		1	2	3	4	5
1	Fe	0.000000				
2	Fe	2.795624	0.000000			
3	P	2.109485	2.087360	0.000000		
4	C	3.663846	3.569971	1.827550	0.000000	
5	H	4.164431	4.266659	2.417039	1.092727	0.000000
6	H	4.430911	3.646460	2.442443	1.090989	1.775213
7	H	3.794212	4.198449	2.427662	1.091749	1.770234
8	H	2.867157	5.235264	3.792722	4.167150	4.562058
9	C	2.133792	4.763223	3.608350	4.446574	4.869762
10	C	2.239606	4.546450	4.008304	5.120385	5.741653
11	H	2.824779	5.416988	4.036866	4.893119	4.843113
12	H	2.983648	4.802562	4.448747	5.403500	6.163375
13	C	2.231440	4.472385	4.311580	5.765445	6.297081
14	H	2.989265	4.693642	4.976617	6.535812	7.133553
15	C	2.185757	4.713660	4.200329	5.637934	5.932351
16	H	2.935217	5.137777	4.804992	6.330508	6.522807
17	C	2.093539	4.859533	3.735394	4.814095	5.002288
18	H	4.021413	2.845721	3.800617	5.335127	5.511919
19	C	3.646649	2.101126	3.545675	5.180531	5.567974
20	C	2.763944	2.109148	3.419909	5.216643	5.738542
21	H	5.397130	2.853460	4.611519	5.787067	6.243711
22	H	2.518840	2.955962	3.738658	5.563823	5.994373
23	C	3.328688	2.109680	3.842098	5.482587	6.216995
24	H	3.470466	2.864263	4.320928	5.888622	6.706790
25	C	4.320224	2.101485	4.175722	5.600766	6.336734
26	H	5.141026	2.848373	4.868855	6.090066	6.910599
27	C	4.480440	2.099896	4.012521	5.421489	5.952131
28	O	5.125283	2.885499	3.520785	3.566350	4.375576
29	C	4.103353	1.736644	2.673443	3.264064	4.087575

		6	7	8	9	10
6	H	0.000000				
7	H	1.783448	0.000000			

8	H	5.103707	3.547934	0.000000		
9	C	5.372695	4.050811	1.079346	0.000000	
10	C	5.884044	4.786112	2.224020	1.408146	0.000000
11	H	5.945758	4.805866	2.698307	2.244716	3.339878
12	H	6.037864	4.960043	2.684510	2.223392	1.079825
13	C	6.523665	5.664714	3.326059	2.281171	1.413439
14	H	7.193752	6.493500	4.335020	3.323924	2.222915
15	C	6.518592	5.643202	3.344397	2.302883	2.299858
16	H	7.195351	6.464066	4.358385	3.349710	3.342589
17	C	5.804961	4.682064	2.244630	1.435558	2.299351
18	H	5.574077	6.178443	6.792783	6.101922	6.055602
19	C	5.357803	5.937729	6.480999	5.770830	5.520458
20	C	5.557429	5.748129	5.593975	4.770745	4.301039
21	H	5.529676	6.651013	8.040149	7.491968	7.195774
22	H	6.081447	6.000038	5.252797	4.316200	3.875569
23	C	5.645169	5.902677	5.880314	5.135771	4.382739
24	H	6.096460	6.125597	5.676339	4.922691	3.928522
25	C	5.504654	6.169334	6.855824	6.232037	5.598640
26	H	5.840255	6.599770	7.468315	6.919262	6.202561
27	C	5.325079	6.192651	7.187639	6.571602	6.195734
28	O	2.798243	4.153737	6.653158	6.600771	6.520691
29	C	2.819883	3.908692	5.945390	5.751475	5.625635
		11	12	13	14	15
11	H	0.000000				
12	H	4.352818	0.000000			
13	C	3.329785	2.225123	0.000000		
14	H	4.341802	2.673601	1.081170	0.000000	
15	C	2.232101	3.341334	1.415178	2.226551	0.000000
16	H	2.695400	4.349254	2.229951	2.683185	1.080328
17	C	1.079458	3.345200	2.284251	3.328771	1.415747
18	H	5.742236	6.673904	5.381321	5.496741	5.033039
19	C	5.812342	6.008477	4.896151	4.903300	4.832083
20	C	5.108295	4.774673	3.578969	3.493434	3.737606
21	H	7.765545	7.456883	6.795501	6.734473	6.921716
22	H	4.437191	4.521809	2.898690	2.837761	2.864703
23	C	6.000688	4.528890	3.895232	3.611915	4.506901
24	H	6.106360	3.889048	3.516157	3.072444	4.432421
25	C	7.031045	5.670823	5.244687	5.013702	5.768272
26	H	7.922742	6.101665	5.983052	5.694424	6.653538
27	C	6.935285	6.472005	5.745739	5.657451	5.932999
28	O	7.522220	6.445407	6.907212	7.189442	7.272730
29	C	6.603527	5.656008	5.877782	6.145269	6.210536
		16	17	18	19	20
16	H	0.000000				
17	C	2.231923	0.000000			
18	H	4.851457	5.487839	0.000000		

	19 C 4.801307 5.368622 1.079277 0.000000<br>20 C 3.814260 4.462887 2.237358 1.428814 0.000000<br>21 H 6.993026 7.325644 2.674670 2.220349 3.343324<br>22 H 2.791539 3.790765 2.691861 2.236415 1.079918<br>23 C 4.789619 5.201374 3.346562 2.303296 1.430741<br>24 H 4.834208 5.183006 4.357725 3.347235 2.241430<br>25 C 6.022560 6.325948 3.337867 2.293508 2.296027<br>26 H 6.979140 7.174291 4.345100 3.334584 3.340090<br>27 C 6.031619 6.415560 2.217693 1.405625 2.299327<br>28 O 7.856836 7.069328 5.147905 4.576711 4.935992<br>29 C 6.751648 6.110901 4.155974 3.526872 3.801973<br>21 22 23 24 25<br>21 H 0.000000<br>22 H 4.343490 0.000000<br>23 C 3.339728 2.245696 0.000000<br>24 H 4.349276 2.708606 1.079087 0.000000<br>25 C 2.243083 3.334001 1.404674 2.217076 0.000000<br>26 H 2.697538 4.345724 2.217433 2.670720 1.079376<br>27 C 1.079001 3.333706 2.299085 3.342679 1.432098<br>28 O 3.825470 5.836870 4.473760 4.979873 3.750184<br>29 C 3.190466 4.688707 3.438240 4.019486 2.875662<br>26 27 28 29<br>26 H 0.000000<br>27 C 2.241443 0.000000<br>28 O 3.665163 3.827599 0.000000<br>29 C 3.059638 2.943468 1.149226 0.000000
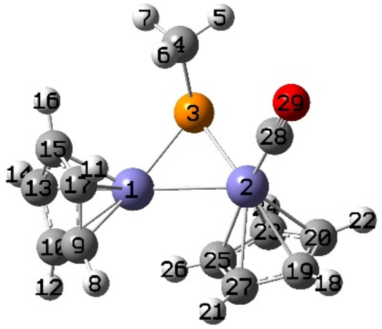<br><br>12T. -3407.916281 21.63 C1<br>WBI 0.23<br>Charge and spin density<br>1 2<br>1 Fe 0.244769 0.148655<br>2 Fe 0.208538 2.029121<br>3 P -0.259876 0.030266<br>4 C -0.023230 0.013746<br>9 C -0.009032 -0.076609<br>10 C -0.045031 -0.007089	1 2 3 4 5<br>1 Fe 0.000000<br>2 Fe 2.617999 0.000000<br>3 P 2.192596 2.208310 0.000000<br>4 C 3.455763 3.491256 1.851624 0.000000<br>5 H 4.360710 3.806620 2.454169 1.091955 0.000000<br>6 H 3.524288 3.635224 2.488673 1.090968 1.773411<br>7 H 3.826739 4.394963 2.426936 1.092673 1.764892<br>8 H 2.922573 4.195556 4.872823 5.732442 6.611139<br>9 C 2.163887 4.024323 4.206876 5.017564 5.978532<br>10 C 2.152738 4.522563 4.300265 5.332822 6.340317<br>11 H 2.871526 4.018173 3.917995 3.878211 4.769069<br>12 H 2.911278 5.072654 5.029796 6.252548 7.226118<br>13 C 2.114195 4.729594 3.820179 4.592282 5.651612<br>14 H 2.852589 5.413313 4.225168 5.009649 6.039859<br>15 C 2.101010 4.391238 3.372256 3.639268 4.725763<br>16 H 2.839298 4.850324 3.437435 3.209770 4.285450<br>17 C 2.132615 3.932958 3.648302 3.976084 4.972694<br>18 H 5.070264 2.955301 5.155758 6.287737 6.403385<br>19 C 4.205067 2.214030 4.348355 5.690195 5.897987

13	C	-0.045461	0.050100
15	C	0.048770	-0.108367
17	C	-0.072538	0.101268
19	C	-0.002856	-0.029199
20	C	-0.045248	-0.011523
23	C	0.017094	0.012911
25	C	0.229150	-0.017443
27	C	-0.103629	0.031422
28	C	-0.033687	-0.113641
29	O	-0.107732	-0.053617

20	C	4.288617	2.202922	3.916676	5.400488	5.485974
21	H	3.480503	2.946635	4.688810	5.982261	6.536917
22	H	5.202130	2.942277	4.460278	5.790788	5.676548
23	C	3.402999	2.171451	3.258807	5.004710	5.286522
24	H	3.731468	2.882592	3.274307	5.042730	5.279855
25	C	2.544696	2.146960	3.327657	5.043994	5.572456
26	H	2.291086	2.978258	3.588293	5.312310	5.990076
27	C	3.255999	2.218619	4.071592	5.526283	5.985943
28	C	3.823384	1.761162	2.812668	3.117903	3.068921
29	O	4.784147	2.906810	3.618088	3.356592	3.047035
		6	7	8	9	10
6	H	0.000000				
7	H	1.775305	0.000000			
8	H	5.294295	6.218818	0.000000		
9	C	4.649888	5.371210	1.079972	0.000000	
10	C	5.210855	5.480031	2.230956	1.417462	0.000000
11	H	3.112829	4.311403	2.680751	2.226972	3.335795
12	H	6.209408	6.399462	2.688746	2.231455	1.080671
13	C	4.574517	4.493329	3.336643	2.292942	1.415153
14	H	5.166597	4.715792	4.347225	3.336877	2.229170
15	C	3.391439	3.589940	3.336306	2.292847	2.293956
16	H	2.982208	2.909933	4.345327	3.335547	3.336981
17	C	3.459807	4.276200	2.225751	1.414154	2.292649
18	H	6.236150	7.275964	5.195368	5.620708	6.333185
19	C	5.754147	6.607988	4.705706	4.989531	5.528708
20	C	5.731090	6.246986	5.562268	5.606603	5.879763
21	H	5.799701	6.762535	2.741206	3.313056	3.997197
22	H	6.200002	6.652417	6.599927	6.627015	6.903662
23	C	5.451910	5.674407	5.098015	4.948735	4.924177
24	H	5.676438	5.563107	5.831485	5.524868	5.274872
25	C	5.270935	5.682045	3.742710	3.671100	3.694941
26	H	5.546771	5.776254	3.480796	3.269274	2.929516
27	C	5.521622	6.326462	3.456791	3.757591	4.234921
28	C	3.034130	4.201117	5.143451	4.997455	5.759389
29	O	3.123936	4.433303	5.958786	5.824360	6.681338
		11	12	13	14	15
11	H	0.000000				
12	H	4.346387	0.000000			
13	C	3.340333	2.227740	0.000000		
14	H	4.350637	2.684683	1.079689	0.000000	
15	C	2.232119	3.338503	1.421682	2.233803	0.000000
16	H	2.689079	4.348170	2.234482	2.691502	1.078972
17	C	1.079402	3.336878	2.296830	3.340404	1.419923
18	H	5.909844	6.573279	7.045014	7.822724	6.855555
19	C	5.485609	5.741042	6.191199	6.900645	6.120562
20	C	6.085723	6.079570	6.308677	6.841177	6.316417

21	H	4.354390	4.126966	5.014869	5.864246	5.081910
22	H	6.930634	7.123438	7.240096	7.725212	7.184515
23	C	5.720181	5.058885	5.263775	5.665204	5.486309
24	H	6.291248	5.380800	5.399544	5.605000	5.717644
25	C	4.782558	3.792873	4.299127	4.848087	4.615866
26	H	4.768741	2.855976	3.594259	4.043923	4.210275
27	C	4.669866	4.385069	5.053986	5.797568	5.141914
28	C	4.159867	6.485208	5.785136	6.522712	5.042130
29	O	4.611503	7.476296	6.619242	7.364989	5.704450

		16	17	18	19	20
16	H	0.000000				
17	C	2.232328	0.000000			
18	H	7.502055	5.985410	0.000000		
19	C	6.785525	5.397756	1.079632	0.000000	
20	C	6.861384	5.891611	2.239533	1.429928	0.000000
21	H	5.975040	4.127722	2.669246	2.215907	3.340465
22	H	7.633629	6.808073	2.691554	2.238795	1.079195
23	C	6.058578	5.300637	3.330950	2.289887	1.401699
24	H	6.181008	5.790310	4.344565	3.336394	2.219884
25	C	5.376414	4.265384	3.333194	2.289259	2.296021
26	H	5.049689	4.040646	4.346043	3.335000	3.337933
27	C	5.946277	4.399430	2.214282	1.401616	2.296008
28	C	5.233028	4.493218	3.479217	3.238001	3.332278
29	O	5.742115	5.143165	4.218789	4.191408	4.311928

		21	22	23	24	25
21	H	0.000000				
22	H	4.345608	0.000000			
23	C	3.347216	2.218476	0.000000		
24	H	4.354423	2.682649	1.079166	0.000000	
25	C	2.235856	3.341714	1.432907	2.236925	0.000000
26	H	2.703780	4.349960	2.245971	2.693515	1.089361
27	C	1.079571	3.335548	2.303082	3.344145	1.426349
28	C	4.149493	3.646829	3.747963	4.334251	3.893587
29	O	5.117170	4.445700	4.845237	5.378020	5.036878

		26	27	28	29
26	H	0.000000			
27	C	2.245612	0.000000		
28	C	4.709445	3.640048	0.000000	
29	O	5.843326	4.698333	1.145870	0.000000

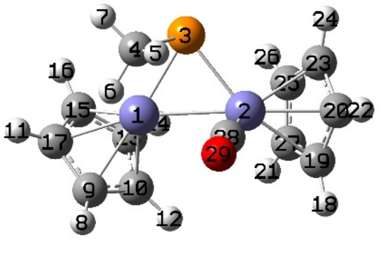

		1	2	3	4	5
1	Fe	0.000000				
2	Fe	2.504985	0.000000			
3	P	2.190769	2.257826	0.000000		
4	C	2.255981	3.315198	1.859370	0.000000	
5	H	3.019544	3.521345	2.512433	1.090280	0.000000
6	H	1.788590	3.496876	2.570040	1.151215	1.738358

13T. -3407.914481 22.76 C1
WBI 0.44

Charge and spin density

		1	2
1	Fe	0.308887	0.472866
2	Fe	0.267518	2.350095
3	P	-0.183170	-0.302227
4	C	0.036704	-0.013384
9	C	-0.040059	-0.033217
10	C	-0.041251	0.040034
13	C	-0.033828	-0.041245
15	C	0.006250	-0.067901
17	C	-0.017392	-0.006809
19	C	-0.012712	-0.026442
20	C	0.091758	-0.005548
23	C	0.010393	0.017548
25	C	-0.030470	-0.031868
27	C	-0.042783	-0.006894
28	C	0.015547	-0.239100
29	O	-0.335393	-0.105908

7	H	2.975382	4.298782	2.463079	1.089917	1.789551
8	H	2.881572	4.647986	5.025216	4.412327	4.836045
9	C	2.120648	4.120064	4.309839	3.973264	4.598118
10	C	2.078171	3.405534	4.028073	4.309553	4.967297
11	H	2.881902	5.379269	4.556621	3.736461	4.535865
12	H	2.835261	3.391994	4.575669	4.993061	5.504991
13	C	2.057445	3.496511	3.523818	4.169615	5.038659
14	H	2.802594	3.556562	3.701582	4.758849	5.621389
15	C	2.130110	4.277224	3.570988	3.768866	4.764486
16	H	2.885377	4.893712	3.760464	4.050465	5.121988
17	C	2.130628	4.574923	4.034133	3.600276	4.446335
18	H	4.855546	2.901725	5.155844	6.031868	6.017670
19	C	4.365560	2.160374	4.335067	5.451088	5.524030
20	C	4.663494	2.159910	3.968057	5.159193	5.087495
21	H	4.073334	2.928029	4.550416	5.828745	6.285786
22	H	5.342482	2.893133	4.557460	5.516645	5.231598
23	C	4.452120	2.189590	3.301802	4.858657	4.993617
24	H	4.960337	2.914241	3.364606	4.935962	5.023034
25	C	3.964842	2.182356	3.275392	4.951940	5.352850
26	H	4.133042	2.921405	3.340901	5.133497	5.701758
27	C	3.945465	2.197338	3.979480	5.353307	5.697323
28	C	2.738938	1.740681	2.969199	2.986507	2.746180
29	O	3.460014	2.889914	3.836227	3.331642	2.788896

		6	7	8	9	10
6	H	0.000000				
7	H	1.767588	0.000000			
8	H	3.333902	4.997748	0.000000		
9	C	3.005488	4.522204	1.079724	0.000000	
10	C	3.613882	4.989483	2.232155	1.415215	0.000000
11	H	2.835095	3.717858	2.678953	2.227208	3.330944
12	H	4.347339	5.796654	2.693799	2.229927	1.079323
13	C	3.732194	4.660451	3.342642	2.296318	1.427713
14	H	4.528606	5.250020	4.352803	3.338755	2.238668
15	C	3.270330	3.956823	3.345582	2.304830	2.308465
16	H	3.769885	3.983829	4.353820	3.347542	3.351865
17	C	2.734494	3.825549	2.231235	1.419445	2.288587
18	H	5.952698	7.081939	5.762198	5.546734	4.578643
19	C	5.525425	6.455533	5.798366	5.406653	4.374601
20	C	5.512384	6.119896	6.542236	6.122290	5.243872
21	H	5.717805	6.677938	5.347825	4.707963	3.349674
22	H	5.918719	6.486297	7.095665	6.795348	6.051267
23	C	5.385419	5.668203	6.788936	6.172423	5.248430
24	H	5.660078	5.616193	7.501532	6.858441	6.040576
25	C	5.288883	5.716701	6.221850	5.478312	4.367686
26	H	5.511867	5.734338	6.530237	5.660677	4.549336
27	C	5.415288	6.244276	5.608396	4.993847	3.765721

28	C	2.944020	4.075418	4.141306	3.994715	3.795538
29	O	3.158286	4.358909	4.254584	4.377802	4.476091
		11	12	13	14	15
11	H	0.000000				
12	H	4.342361	0.000000			
13	C	3.325977	2.239752	0.000000		
14	H	4.335242	2.696292	1.078953	0.000000	
15	C	2.222532	3.350602	1.420943	2.230440	0.000000
16	H	2.681870	4.360820	2.234990	2.689300	1.078756
17	C	1.080799	3.333519	2.281495	3.324298	1.412119
18	H	7.446800	3.828517	5.178550	5.066653	6.363660
19	C	7.118481	3.812217	4.694617	4.468868	5.849483
20	C	7.533892	4.897287	5.369076	5.141421	6.327430
21	H	6.442473	2.794299	3.373113	2.843671	4.748727
22	H	8.170216	5.690317	6.286287	6.147016	7.165489
23	C	7.300996	5.117535	4.992861	4.603214	5.854105
24	H	7.728916	6.034621	5.646021	5.257639	6.329162
25	C	6.688750	4.245295	3.947562	3.374167	4.965174
26	H	6.627730	4.599607	3.797031	3.043615	4.706522
27	C	6.605951	3.326646	3.751604	3.291531	4.998839
28	C	5.216428	3.788499	4.341409	4.763794	4.845071
29	O	5.488023	4.464139	5.197511	5.749497	5.550621
		16	17	18	19	20
16	H	0.000000				
17	C	2.228526	0.000000			
18	H	7.168338	6.527863	0.000000		
19	C	6.545126	6.190829	1.079447	0.000000	
20	C	6.884513	6.705986	2.238966	1.429707	0.000000
21	H	5.402241	5.385960	2.687481	2.224784	3.340528
22	H	7.736610	7.415228	2.690716	2.238521	1.079331
23	C	6.216031	6.481205	3.336622	2.295442	1.408964
24	H	6.546192	6.998311	4.348973	3.340805	2.224843
25	C	5.329770	5.766606	3.332991	2.288051	2.289054
26	H	4.877716	5.723396	4.344381	3.331977	3.330430
27	C	5.601608	5.605989	2.228846	1.411479	2.295283
28	C	5.586986	4.623934	3.364422	3.108487	3.060680
29	O	6.321998	5.063684	4.048194	4.015169	3.937841
		21	22	23	24	25
21	H	0.000000				
22	H	4.348445	0.000000			
23	C	3.337496	2.225531	0.000000		
24	H	4.343778	2.687796	1.079136	0.000000	
25	C	2.225240	3.334318	1.420482	2.228936	0.000000
26	H	2.683976	4.342215	2.229686	2.678649	1.079610
27	C	1.080071	3.336708	2.294172	3.335105	1.413841
28	C	4.267120	3.269229	3.588970	4.145759	3.895675

29	O	5.289272	3.890379	4.616069	5.094261	5.030139

		26	27	28	29
26	H	0.000000			
27	C	2.229244	0.000000		
28	C	4.656461	3.662715	0.000000	
29	O	5.804357	4.730345	1.150568	0.000000

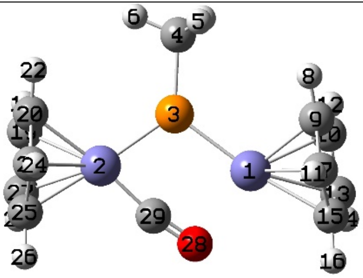

14T. -3407.912277 24.14 Cs
WBI 0.08

Charge and spin density

		1	2
1	Fe	0.323307	2.114868
2	Fe	0.338918	0.620567
3	P	-0.160899	-0.617341
4	C	-0.010867	-0.016729
9	C	-0.015448	-0.075552
10	C	-0.069423	-0.018646
13	C	-0.016669	0.036968
15	C	0.039310	-0.032505
17	C	-0.079133	0.041611
19	C	-0.036360	0.012966
20	C	0.042537	-0.022955
23	C	-0.081838	0.041582
25	C	0.000534	-0.021371
27	C	-0.047270	-0.048686
28	O	-0.225061	-0.006122
29	C	-0.001641	-0.008655

		1	2	3	4	5
1	Fe	0.000000				
2	Fe	3.493571	0.000000			
3	P	2.208299	2.110030	0.000000		
4	C	3.577682	3.535127	1.839342	0.000000	
5	H	3.776174	4.136050	2.437613	1.092902	0.000000
6	H	4.480658	3.561273	2.445056	1.090569	1.775979
7	H	3.811610	4.285066	2.442989	1.092302	1.770151
8	H	2.797681	5.371418	3.458129	3.379582	2.866236
9	C	2.063426	5.171573	3.395506	3.832712	3.555197
10	C	2.123263	5.416963	3.662518	4.243088	4.292294
11	H	2.918229	5.515440	4.387433	5.175101	4.708770
12	H	2.858799	5.776491	3.907555	4.177292	4.350810
13	C	2.223680	5.620742	4.271648	5.282286	5.392021
14	H	2.969937	6.093289	4.915166	6.005246	6.249468
15	C	2.219880	5.505890	4.393605	5.539618	5.468752
16	H	2.965696	5.894001	5.115685	6.429754	6.376609
17	C	2.177899	5.284551	3.949105	4.793296	4.487782
18	H	5.718257	2.850642	3.809113	4.218117	5.136773
19	C	5.383277	2.109072	3.565578	4.271425	5.063117
20	C	5.321120	2.091491	3.424390	3.938375	4.472087
21	H	5.770159	2.875916	4.647510	5.893427	6.724990
22	H	5.640513	2.847918	3.590326	3.587575	4.000232
23	C	5.318196	2.103261	3.863415	4.782023	5.155478
24	H	5.604569	2.844279	4.327844	5.156828	5.312892
25	C	5.364911	2.114909	4.213982	5.474116	6.006046
26	H	5.689546	2.861430	4.910159	6.329595	6.800009
27	C	5.410076	2.125255	4.053548	5.210724	5.961896
28	O	2.162406	2.883703	3.062380	4.877942	5.312837
29	C	2.497842	1.709968	2.416455	4.241886	4.754528

		6	7	8	9	10
6	H	0.000000				
7	H	1.778407	0.000000			
8	H	4.429603	3.409772	0.000000		
9	C	4.904381	3.853342	1.079481	0.000000	
10	C	5.305593	3.975375	2.245962	1.438644	0.000000
11	H	6.098981	5.576852	2.699057	2.236625	3.358977
12	H	5.201460	3.641876	2.701258	2.248882	1.079311
13	C	6.297444	5.142724	3.333222	2.292968	1.403843
14	H	6.974897	5.774684	4.348112	3.340233	2.220167

15	C	6.530499	5.658819	3.322305	2.277566	2.284745
16	H	7.370182	6.624198	4.334589	3.322049	3.324945
17	C	5.793838	5.026498	2.234598	1.420500	2.313279
18	H	3.616557	4.662547	7.171515	7.177774	7.174690
19	C	3.762958	4.912566	6.979714	6.939390	7.072278
20	C	3.381105	4.815643	6.556880	6.650385	7.070945
21	H	5.656504	6.392376	8.019248	7.686111	7.560089
22	H	2.845593	4.500616	6.408081	6.675930	7.207769
23	C	4.453150	5.729123	6.810398	6.774932	7.286447
24	H	4.884820	6.184431	6.860657	6.877602	7.572933
25	C	5.251919	6.285982	7.345314	7.116012	7.403194
26	H	6.210485	7.137801	7.838601	7.503500	7.785925
27	C	4.910425	5.846634	7.448067	7.218480	7.278169
28	O	5.468842	5.268285	4.924348	4.177988	4.038909
29	C	4.649688	4.785661	5.018792	4.490060	4.533522
		11	12	13	14	15
11	H	0.000000				
12	H	4.367823	0.000000			
13	C	3.344125	2.222361	0.000000		
14	H	4.348431	2.684748	1.080121	0.000000	
15	C	2.223535	3.330757	1.419069	2.228481	0.000000
16	H	2.676429	4.337091	2.226054	2.673209	1.080674
17	C	1.079310	3.354763	2.301310	3.340979	1.407847
18	H	8.053956	7.132063	7.636307	7.949741	7.901113
19	C	7.528684	7.192431	7.425568	7.797146	7.504768
20	C	6.987620	7.305877	7.506445	8.052172	7.379090
21	H	8.111857	7.756838	7.525759	7.640755	7.618850
22	H	7.103823	7.382402	7.818355	8.449660	7.706786
23	C	6.674679	7.708777	7.502772	8.054006	7.145147
24	H	6.476063	8.092679	7.780448	8.422813	7.244762
25	C	7.040404	7.828622	7.407041	7.789179	7.116912
26	H	7.173860	8.309234	7.605784	7.937775	7.193025
27	C	7.560053	7.522647	7.363292	7.629855	7.347305
28	O	4.304966	4.675527	3.543462	3.784815	3.359871
29	C	4.692884	5.062262	4.362475	4.720085	4.197189
		16	17	18	19	20
16	H	0.000000				
17	C	2.219097	0.000000			
18	H	8.424881	7.685732	0.000000		
19	C	7.940419	7.267924	1.079406	0.000000	
20	C	7.829365	6.909756	2.233810	1.422235	0.000000
21	H	7.813394	7.770597	2.678679	2.225008	3.338985
22	H	8.256742	7.066947	2.692009	2.235245	1.079100
23	C	7.417926	6.736998	3.341259	2.298026	1.421596
24	H	7.470627	6.710702	4.352414	3.342142	2.235283
25	C	7.262997	6.986440	3.334327	2.290751	2.290898

		1	2	3	4	5
26	H	7.177261	7.179142	4.342724	3.332950	3.334188
27	C	7.601953	7.313556	2.222047	1.410960	2.295560
28	O	3.454834	3.831463	5.285073	4.758379	4.968663
29	C	4.429483	4.334043	4.229318	3.622510	3.790635

		21	22	23	24	25
21	H	0.000000				
22	H	4.347971	0.000000			
23	C	3.340484	2.234170	0.000000		
24	H	4.349423	2.693888	1.079302	0.000000	
25	C	2.236851	3.333781	1.411917	2.223978	0.000000
26	H	2.692063	4.343708	2.223490	2.677242	1.079355
27	C	1.078907	3.337843	2.298825	3.341738	1.424667
28	O	4.361514	5.664790	4.611086	5.032655	4.127158
29	C	3.489031	4.523392	3.483630	4.003316	3.074360

		26	27	28	29
26	H	0.000000			
27	C	2.235202	0.000000		
28	O	4.161585	4.234229	0.000000	
29	C	3.308247	3.177720	1.183317	0.000000

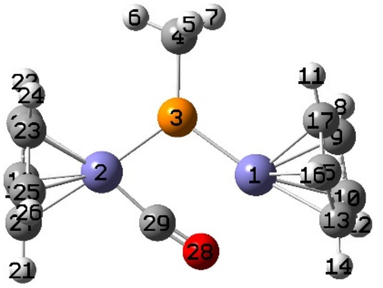

15T. -3407.912176 24.21 C1
WBI 0.08

Charge and spin density

		1	2
1	Fe	0.326296	2.119246
2	Fe	0.350050	0.595072
3	P	-0.164609	-0.591663
4	C	-0.010870	-0.015766
9	C	-0.067101	-0.018876
10	C	-0.018705	0.038271
13	C	0.041220	-0.033284
15	C	-0.083289	0.041937
17	C	-0.006167	-0.077723
19	C	-0.063204	-0.079249
20	C	0.006092	0.030850
23	C	0.000030	0.002925
25	C	-0.066915	-0.003267

		1	2	3	4	5
1	Fe	0.000000				
2	Fe	3.478096	0.000000			
3	P	2.207197	2.110715	0.000000		
4	C	3.577334	3.526443	1.839346	0.000000	
5	H	3.821873	4.016771	2.426755	1.092355	0.000000
6	H	4.477968	3.578121	2.443546	1.090401	1.771344
7	H	3.774025	4.351629	2.456424	1.092005	1.770334
8	H	2.856870	5.767881	3.896942	4.211998	4.548378
9	C	2.123032	5.406004	3.657430	4.261809	4.449216
10	C	2.223913	5.606891	4.267353	5.297112	5.509390
11	H	2.799037	5.360717	3.462694	3.362130	3.001222
12	H	2.969238	6.080117	4.907457	6.029695	6.373978
13	C	2.219272	5.485606	4.393508	5.532407	5.531343
14	H	2.965587	5.872181	5.116837	6.419384	6.413681
15	C	2.178467	5.264792	3.953662	4.770934	4.536348
16	H	2.920712	5.495922	4.397847	5.138470	4.710573
17	C	2.064059	5.157578	3.397243	3.823631	3.671523
18	H	5.801302	2.874572	4.399834	5.435757	6.230472
19	C	5.420136	2.124874	3.904897	4.934623	5.595380
20	C	5.371401	2.114768	3.459472	4.022866	4.602547
21	H	5.682901	2.842286	4.898228	6.345091	6.812423
22	H	5.714405	2.860454	3.623859	3.748889	4.430140
23	C	5.294421	2.109475	3.540569	4.114463	4.369046
24	H	5.579306	2.854602	3.771799	3.935225	3.960101
25	C	5.277749	2.099097	4.006487	5.045517	5.271889
26	H	5.566119	2.850652	4.585370	5.639637	5.687820

27	C	-0.003583	0.010764
28	O	-0.222631	-0.006336
29	C	-0.016613	-0.012902

27	C	5.352475	2.100760	4.204739	5.475320	5.939920
28	O	2.165499	2.884761	3.072638	4.889969	5.304627
29	C	2.485187	1.710185	2.418874	4.241400	4.697988
		6	7	8	9	10
6	H	0.000000				
7	H	1.779379	0.000000			
8	H	5.176386	3.611630	0.000000		
9	C	5.292614	3.911099	1.079323	0.000000	
10	C	6.286237	5.102428	2.222142	1.403688	0.000000
11	H	4.438921	3.214300	2.701463	2.246384	3.333786
12	H	6.956309	5.773448	2.684741	2.220289	1.080285
13	C	6.531206	5.576524	3.330469	2.284423	1.419187
14	H	7.373425	6.551067	4.336884	3.324726	2.226218
15	C	5.804493	4.890435	3.354967	2.313493	2.302020
16	H	6.121599	5.415947	4.368290	3.359465	3.345020
17	C	4.907517	3.710322	2.249222	1.439133	2.293569
18	H	5.056468	6.028650	7.556135	7.469830	7.597820
19	C	4.561213	5.681780	7.415207	7.225681	7.397722
20	C	3.463382	4.865050	7.204696	7.062506	7.478617
21	H	6.239123	7.150252	8.164122	7.720640	7.517697
22	H	2.975509	4.471401	7.157635	7.166739	7.745650
23	C	3.701460	5.128421	7.437147	7.121860	7.504931
24	H	3.478287	5.000162	7.597102	7.279820	7.796264
25	C	4.848551	6.036085	7.763809	7.303142	7.423260
26	H	5.550683	6.662783	8.209451	7.629748	7.663301
27	C	5.275444	6.329095	7.746729	7.362333	7.353727
28	O	5.477104	5.307264	4.664756	4.032459	3.532394
29	C	4.656447	4.831681	5.046047	4.519036	4.345215
		11	12	13	14	15
11	H	0.000000				
12	H	4.348829	0.000000			
13	C	3.322675	2.228712	0.000000		
14	H	4.335014	2.673433	1.080741	0.000000	
15	C	2.234878	3.341812	1.408281	2.219389	0.000000
16	H	2.699605	4.349446	2.224247	2.676989	1.079519
17	C	1.079620	3.341004	2.277665	3.322173	1.420447
18	H	7.844652	7.745874	7.794730	8.109900	7.855083
19	C	7.339340	7.684439	7.431397	7.749518	7.345332
20	C	6.789508	7.923272	7.495547	7.957808	7.154289
21	H	7.984465	7.745324	7.253106	7.258092	7.359610
22	H	6.842081	8.187927	7.910653	8.484368	7.514310
23	C	6.536461	8.085219	7.264498	7.664952	6.766330
24	H	6.361393	8.484787	7.494034	7.959951	6.802773
25	C	6.940667	7.936530	7.030237	7.235321	6.703531
26	H	7.145169	8.229892	7.075358	7.174051	6.702567
27	C	7.416393	7.683933	7.135276	7.289634	7.067831

28	O	4.932463	3.765481	3.356085	3.450946	3.837890
29	C	5.013709	4.699559	4.181273	4.414003	4.324060
		16	17	18	19	20
16	H	0.000000				
17	C	2.236827	0.000000			
18	H	8.239384	7.606593	0.000000		
19	C	7.612874	7.163904	1.078743	0.000000	
20	C	7.356649	6.819871	2.231376	1.417110	0.000000
21	H	7.482481	7.600031	2.686363	2.227481	3.328477
22	H	7.787429	6.981444	2.685031	2.227130	1.079590
23	C	6.764890	6.614829	3.341153	2.298483	1.416759
24	H	6.697723	6.600243	4.349200	3.340433	2.228287
25	C	6.643925	6.827655	3.346795	2.304065	2.294550
26	H	6.483137	7.017290	4.355022	3.345865	3.338537
27	C	7.186841	7.158532	2.232212	1.417438	2.285209
28	O	4.319293	4.181950	4.701344	4.423961	4.916265
29	C	4.688943	4.480601	3.762081	3.340354	3.753106
		21	22	23	24	25
21	H	0.000000				
22	H	4.338144	0.000000			
23	C	3.332472	2.228241	0.000000		
24	H	4.343646	2.682108	1.079357	0.000000	
25	C	2.229999	3.337258	1.418223	2.230833	0.000000
26	H	2.686275	4.348129	2.233867	2.692834	1.078827
27	C	1.079476	3.328536	2.289552	3.333901	1.420573
28	O	4.041944	5.551315	4.914870	5.550827	4.403061
29	C	3.216677	4.438765	3.746604	4.430087	3.309785
		26	27	28	29	
26	H	0.000000				
27	C	2.233417	0.000000			
28	O	4.679240	4.065881	0.000000		
29	C	3.725219	3.024818	1.183167	0.000000	

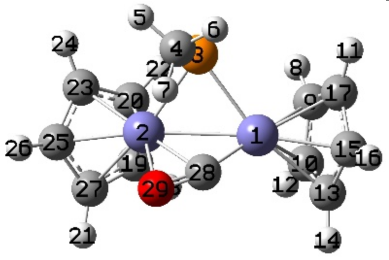

16T. -3407.906312 27.89 C1
WBI 0.37

Charge and spin density

		1	2
1	Fe	0.372086	1.423527

		1	2	3	4	5
1	Fe	0.000000				
2	Fe	2.605771	0.000000			
3	P	2.280886	2.248878	0.000000		
4	C	3.389680	3.214422	1.865229	0.000000	
5	H	4.335652	3.650399	2.436535	1.093682	0.000000
6	H	3.687719	4.142481	2.442865	1.093743	1.760625
7	H	3.477975	3.159547	2.536487	1.090759	1.775313
8	H	2.904573	4.217248	3.431317	5.171928	5.808129
9	C	2.165711	4.043219	3.367740	4.940683	5.738220
10	C	2.225580	3.998625	4.056031	5.526358	6.391043
11	H	2.836725	5.035904	3.436922	4.392925	5.229492
12	H	2.981542	4.122017	4.626321	6.188104	6.975268
13	C	2.157107	4.384265	4.383705	5.461679	6.457984

2	Fe	0.195782	1.269828
3	P	-0.218575	-0.332072
4	C	-0.037355	0.015894
9	C	0.030680	-0.013301
10	C	-0.034996	-0.009127
13	C	-0.003309	-0.018844
15	C	-0.073439	-0.017396
17	C	0.021488	-0.016774
19	C	-0.014807	-0.034901
20	C	-0.011657	-0.020246
23	C	0.007800	-0.021079
25	C	-0.024029	-0.026416
27	C	0.025714	0.024060
28	C	-0.035205	-0.177032
29	O	-0.200178	-0.046122

14	H	2.897922	4.828016	5.175788	6.092994	7.106584
15	C	2.094714	4.690718	4.014769	4.861053	5.896084
16	H	2.822983	5.358182	4.590514	5.060780	6.133830
17	C	2.110593	4.506141	3.362696	4.514342	5.426501
18	H	3.810523	2.954615	4.508760	5.993227	6.481753
19	C	3.758630	2.193239	3.954815	5.326968	5.689659
20	C	3.758969	2.068146	3.188935	4.679955	4.871253
21	H	4.798726	2.934035	5.174134	5.958687	6.300581
22	H	3.842333	2.799841	3.190383	4.893680	5.090322
23	C	4.401946	2.066155	3.287748	4.299500	4.227696
24	H	4.972807	2.803509	3.386561	4.197554	3.870889
25	C	4.670741	2.095966	4.005089	4.694427	4.711337
26	H	5.410538	2.833861	4.618133	4.919233	4.799322
27	C	4.323066	2.189518	4.369839	5.316914	5.590352
28	C	1.709456	2.033145	2.713369	3.115306	4.000795
29	O	2.874597	2.093750	3.311603	3.310243	3.994171
		6	7	8	9	10
6	H	0.000000				
7	H	1.772102	0.000000			
8	H	5.342287	5.742022	0.000000		
9	C	5.058676	5.357958	1.079362	0.000000	
10	C	5.775842	5.690009	2.226667	1.411521	0.000000
11	H	4.020001	4.961855	2.675078	2.228349	3.335206
12	H	6.574173	6.310608	2.683953	2.224760	1.079706
13	C	5.580167	5.440309	3.327774	2.284108	1.410790
14	H	6.257285	5.894867	4.338117	3.327700	2.225122
15	C	4.720920	4.961948	3.334254	2.292930	2.296151
16	H	4.756271	5.056020	4.347124	3.338740	3.337159
17	C	4.353160	4.918620	2.227781	1.420443	2.295224
18	H	6.762063	6.011697	4.030824	4.006742	3.361362
19	C	6.196210	5.349967	4.255872	4.300271	3.906897
20	C	5.539690	4.959096	3.821751	4.128794	4.217799
21	H	6.920902	5.579731	6.195761	5.978417	5.298872
22	H	5.611487	5.367635	3.124450	3.676621	4.032476
23	C	5.296373	4.505187	4.967402	5.218155	5.371599
24	H	5.162839	4.562755	5.367122	5.725453	6.096332
25	C	5.756387	4.562855	5.814913	5.850756	5.696941
26	H	6.010187	4.648533	6.797835	6.788562	6.633426
27	C	6.298193	5.110418	5.456925	5.375526	4.913426
28	C	3.701711	2.689325	4.469232	3.820902	3.547245
29	O	4.077059	2.606569	5.495793	4.939102	4.610111
		11	12	13	14	15
11	H	0.000000				
12	H	4.344032	0.000000			
13	C	3.341449	2.222660	0.000000		
14	H	4.351648	2.679584	1.079455	0.000000	

15	C	2.236615	3.339443	1.424352	2.233534	0.000000
16	H	2.704715	4.346180	2.234122	2.685509	1.079239
17	C	1.078919	3.338678	2.295153	3.337538	1.418288
18	H	5.985478	2.670034	4.283377	4.542328	5.272983
19	C	6.038433	3.445612	4.742644	5.075148	5.532640
20	C	5.601804	3.988938	5.161329	5.733806	5.637982
21	H	7.511069	4.866076	5.673124	5.630430	6.553172
22	H	5.150869	3.858945	5.143573	5.863107	5.510076
23	C	6.333941	5.255639	6.113004	6.634026	6.445518
24	H	6.567150	6.081708	6.837255	7.444479	6.989600
25	C	7.038496	5.502444	6.238115	6.541716	6.729616
26	H	7.807385	6.485782	7.043726	7.281460	7.474014
27	C	6.883948	4.539194	5.464916	5.629102	6.220190
28	C	4.383931	4.006937	3.236800	3.493016	3.392674
29	O	5.509477	4.910712	4.333074	4.427960	4.562274
		16	17	18	19	20
16	H	0.000000				
17	C	2.235712	0.000000			
18	H	6.208880	5.139543	0.000000		
19	C	6.417687	5.308938	1.078836	0.000000	
20	C	6.535525	5.088979	2.236180	1.424592	0.000000
21	H	7.223048	6.726212	2.671861	2.214283	3.325205
22	H	6.468175	4.730465	2.694251	2.236942	1.079203
23	C	7.216035	5.945582	3.353935	2.312007	1.425838
24	H	7.719930	6.347971	4.361194	3.353174	2.237037
25	C	7.417932	6.518725	3.335845	2.292064	2.284627
26	H	8.065665	7.335402	4.340788	3.331771	3.331400
27	C	6.964360	6.178701	2.215606	1.399998	2.280454
28	C	3.778999	3.761180	3.948229	3.645695	3.897217
29	O	4.860854	4.940119	4.271478	3.780231	4.114127
		21	22	23	24	25
21	H	0.000000				
22	H	4.334455	0.000000			
23	C	3.341703	2.232689	0.000000		
24	H	4.355074	2.686523	1.078676	0.000000	
25	C	2.231413	3.326925	1.415847	2.237234	0.000000
26	H	2.679281	4.341112	2.230730	2.702111	1.080068
27	C	1.080401	3.322949	2.301219	3.347336	1.424137
28	C	3.874131	4.406156	4.095599	4.739910	3.885357
29	O	3.398886	4.842489	3.958113	4.596621	3.402068
		26	27	28	29	
26	H	0.000000				
27	C	2.232624	0.000000			
28	C	4.375027	3.617934	0.000000		
29	O	3.632111	3.297824	1.194200	0.000000	

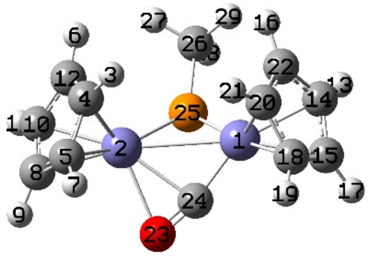

17S. -3407.897832 33.21 C1
WBI 0.36

Charge and spin density

		1
1	Fe	0.352836
2	Fe	0.357304
4	C	-0.095868
5	C	-0.065560
8	C	0.029619
10	C	0.024081
12	C	-0.063508
14	C	-0.064132
15	C	-0.061531
18	C	0.045388
20	C	-0.179886
22	C	0.395025
23	O	-0.527922
24	C	-0.142516
25	P	-0.714638
26	C	0.711307

		1	2	3	4	5
1	Fe	0.000000				
2	Fe	2.765279	0.000000			
3	H	3.798933	2.787157	0.000000		
4	C	3.848311	2.046396	1.079158	0.000000	
5	C	4.205256	2.076314	2.224323	1.413001	0.000000
6	H	4.524884	2.801153	2.687591	2.235760	3.342819
7	H	4.469205	2.834538	2.682967	2.227893	1.079131
8	C	4.733887	2.068964	3.332203	2.288587	1.424613
9	H	5.360878	2.811207	4.340467	3.330780	2.234567
10	C	4.754345	2.057985	3.340899	2.298950	2.302204
11	H	5.407319	2.806971	4.351475	3.342924	3.344358
12	C	4.217048	2.032930	2.231219	1.423229	2.301304
13	H	2.818468	5.279070	5.984970	6.237026	6.892448
14	C	2.073718	4.720836	5.205099	5.529475	6.133740
15	C	2.067229	4.795135	5.448078	5.720061	6.023006
16	H	2.835041	4.521915	4.000405	4.570025	5.603281
17	H	2.820619	5.417229	6.385560	6.561800	6.714556
18	C	2.082725	4.426745	4.563244	4.957059	5.195948
19	H	2.832881	4.774426	4.894018	5.248353	5.234499
20	C	2.076536	4.070746	3.562494	4.153443	4.733799
21	H	2.830924	4.141627	2.931535	3.707743	4.335720
22	C	2.076044	4.275546	4.073313	4.582477	5.388617
23	O	2.894808	2.151951	4.392344	3.815957	3.178872
24	C	1.734654	2.105500	3.994858	3.661712	3.421249
25	P	2.100468	2.125176	3.959412	3.612729	4.127998
26	C	3.506897	3.629488	4.798370	4.589488	5.450182
27	H	4.228993	3.706902	4.623456	4.344565	5.302615
28	H	4.143634	4.324361	5.828147	5.533303	6.263900
29	H	3.531306	4.258607	4.927283	4.955618	5.952191

		6	7	8	9	10
6	H	0.000000				
7	H	4.352576	0.000000			
8	C	3.335790	2.238325	0.000000		
9	H	4.344811	2.693011	1.079884	0.000000	
10	C	2.239512	3.343890	1.411086	2.220671	0.000000
11	H	2.700747	4.351750	2.223278	2.673052	1.079157
12	C	1.078815	3.344552	2.292487	3.334805	1.425393
13	H	6.206121	7.230483	7.341239	8.007225	7.033670
14	C	5.833968	6.370871	6.733767	7.413933	6.577606
15	C	6.474997	6.046490	6.680522	7.218513	6.812547
16	H	4.553692	6.003344	6.341481	7.251648	5.942529
17	H	7.319551	6.690796	7.261123	7.675766	7.457469
18	C	6.122716	5.053335	6.088677	6.662060	6.402881
19	H	6.713777	4.877079	6.185022	6.640999	6.723538
20	C	5.165400	4.750976	5.729142	6.489688	5.849287

21	H	4.968165	4.272711	5.506967	6.320577	5.698779
22	C	4.956280	5.651970	6.164995	6.981873	5.972486
23	O	4.951606	3.300335	3.143444	3.212333	3.781901
24	C	4.715698	3.577369	3.705452	4.057640	4.099566
25	P	3.305823	4.827811	4.092499	4.756566	3.570870
26	C	3.369442	6.283537	5.350027	6.109309	4.418503
27	H	2.672689	6.241389	5.109570	5.923570	3.976770
28	H	4.262197	7.098682	5.991334	6.628152	5.045581
29	H	3.854238	6.706727	6.075736	6.904073	5.213490
		11	12	13	14	15
11	H	0.000000				
12	C	2.238530	0.000000			
13	H	7.479332	6.322971	0.000000		
14	C	7.155575	5.826012	1.079320	0.000000	
15	C	7.461081	6.235498	2.226828	1.417791	0.000000
16	H	6.587957	4.814976	2.688798	2.231223	3.340677
17	H	8.038394	7.033023	2.682574	2.230739	1.078691
18	C	7.208206	5.756925	3.336347	2.293773	1.423000
19	H	7.576802	6.203633	4.345279	3.336573	2.233642
20	C	6.697048	4.945560	3.338919	2.295268	2.297453
21	H	6.639320	4.703529	4.348773	3.338237	3.340010
22	C	6.666351	4.998670	2.229463	1.416964	2.297567
23	O	4.337191	4.142747	5.470911	4.870377	4.363037
24	C	4.734692	4.066672	4.361424	3.705251	3.247309
25	P	3.895040	3.216558	3.610321	3.413868	3.928781
26	C	4.494768	3.832233	3.779740	3.971019	4.963744
27	H	3.958688	3.346269	4.781254	4.882497	5.861858
28	H	4.930291	4.691784	4.043862	4.455513	5.411852
29	H	5.417384	4.398895	3.187968	3.459169	4.662263
		16	17	18	19	20
16	H	0.000000				
17	H	4.349510	0.000000			
18	C	3.337346	2.236419	0.000000		
19	H	4.346811	2.692358	1.079278	0.000000	
20	C	2.234973	3.339725	1.413475	2.225165	0.000000
21	H	2.692374	4.348660	2.225373	2.679455	1.078974
22	C	1.079043	3.339814	2.295123	3.338266	1.423465
23	O	5.554965	4.606180	4.089611	4.087161	4.441214
24	C	4.472080	3.620693	3.026563	3.222836	3.361742
25	P	3.495333	4.507155	4.174544	4.897821	3.832891
26	C	3.498466	5.585030	5.397182	6.293902	4.782577
27	H	4.013873	6.550429	6.115441	6.968958	5.345466
28	H	4.361950	5.861505	6.053886	6.942271	5.617577
29	H	2.749480	5.360425	5.143868	6.134272	4.417750
		21	22	23	24	25
21	H	0.000000				

	22 C 2.235706 0.000000<br>23 O 4.741696 4.910899 0.000000<br>24 C 3.809774 3.765017 1.179144 0.000000<br>25 P 4.344571 3.338278 3.147032 2.513986 0.000000<br>26 C 5.274516 3.822377 4.966922 4.329067 1.835037<br>27 H 5.649634 4.493166 5.380654 4.883643 2.442713<br>28 H 6.214341 4.601108 5.383592 4.831453 2.418169<br>29 H 4.950915 3.246274 5.549474 4.729297 2.452491<br>26 27 28 29<br>26 C 0.000000<br>27 H 1.091689 0.000000<br>28 H 1.093161 1.767273 0.000000<br>29 H 1.091259 1.785989 1.771638 0.000000

Table S3. Distance matrix for the lowest energy $Cp_2Fe_2PMe(CO)_2$ structures as optimized at the PBE0/def2TZVP level. Included are the ranking order, spin multiplicity (S, T or Q), total energy (in a.u.), relative energy (in kcal/mol), symmetry point group Wiberg bond indices for the Fe-Fe bonds, Mulliken charges and the spin density (Cp moieties are omitted for clarity).

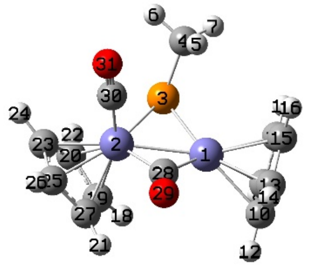

1T -3521.208162 0.00 C1
WBI 0.29

Charge and spin density

		1	2
1	Fe	0.300873	2.584996
2	Fe	-0.060909	-0.235780
3	P	-0.197216	-0.259934
4	C	-0.002284	0.014949
9	C	-0.041231	0.024381
10	C	-0.052619	0.029645
13	C	0.003732	-0.031583
15	C	-0.048308	0.033911
17	C	0.013474	-0.029045
19	C	0.047138	0.005785
20	C	0.078181	-0.016778
23	C	0.002490	0.012836
25	C	0.001967	0.000201
27	C	0.026497	0.011569
28	C	0.202243	-0.138859
29	O	-0.164735	-0.015507
30	C	-0.011771	0.006403
31	O	-0.097522	0.002811

		1	2	3	4	5
1	Fe	0.000000				
2	Fe	2.510907	0.000000			
3	P	2.251548	2.255240	0.000000		
4	C	3.222126	3.448928	1.864334	0.000000	
5	H	3.154012	3.574003	2.535811	1.091036	0.000000
6	H	4.142888	3.767044	2.451258	1.093613	1.769709
7	H	3.674504	4.368218	2.427196	1.093095	1.774771
8	H	2.885797	4.851580	3.918835	5.111700	5.392371
9	C	2.165384	4.472118	3.663406	4.629192	4.712888
10	C	2.210416	4.424659	4.293620	5.229484	5.071038
11	H	2.890803	5.012268	3.340348	3.467734	3.636630
12	H	2.944497	4.758679	4.988402	6.115081	5.977859
13	C	2.236891	4.483006	4.390295	4.848399	4.411410
14	H	2.966143	4.838182	5.127600	5.476558	4.878992
15	C	2.233065	4.590969	3.871025	3.928527	3.505489
16	H	2.960994	5.027207	4.258869	3.825471	3.186817
17	C	2.153723	4.548101	3.326770	3.717201	3.699059
18	H	3.410626	2.886713	3.523925	5.355511	5.777246
19	C	3.433714	2.119162	3.303461	5.030626	5.401902
20	C	4.000122	2.117571	3.016546	4.585331	5.117009
21	H	3.903417	2.857736	4.752643	6.172537	6.160455
22	H	4.402572	2.863371	2.962659	4.500478	5.229423
23	C	4.511377	2.078967	3.633777	4.811763	5.151419
24	H	5.276501	2.828476	4.089726	4.954323	5.321217
25	C	4.370278	2.091885	4.223368	5.398834	5.492360
26	H	5.034678	2.836444	5.038408	5.978054	5.917508
27	C	3.713542	2.109234	4.045694	5.514045	5.633195
28	C	1.910600	1.924424	3.258468	4.079009	3.748314
29	O	2.911360	2.894713	4.409733	5.054541	4.553729
30	C	3.368484	1.744331	2.837328	2.997961	2.815862
31	O	4.258568	2.887518	3.659852	3.246195	2.870274

		6	7	8	9	10
6	H	0.000000				
7	H	1.762096	0.000000			
8	H	6.066774	5.006538	0.000000		
9	C	5.653777	4.597053	1.079492	0.000000	
10	C	6.239384	5.412926	2.229382	1.410019	0.000000
11	H	4.504739	2.978544	2.685151	2.235432	3.332637
12	H	7.074893	6.375392	2.696260	2.228241	1.080001

13	C	5.869221	5.075080	3.338851	2.292253	1.423078
14	H	6.433884	5.816888	4.346816	3.333177	2.231762
15	C	4.998956	3.948266	3.342250	2.300815	2.292939
16	H	4.837569	3.807464	4.353446	3.345689	3.336708
17	C	4.799740	3.526497	2.234816	1.426844	2.291594
18	H	5.835701	5.886422	3.825552	4.063256	4.197553
19	C	5.373602	5.727941	4.569898	4.614023	4.637880
20	C	4.655343	5.331822	5.350545	5.395639	5.631962
21	H	6.612338	6.984222	5.422262	5.179185	4.558485
22	H	4.483908	5.098333	5.394802	5.575523	6.060306
23	C	4.705328	5.739698	6.405381	6.260958	6.286843
24	H	4.607672	5.902809	7.266309	7.101959	7.194237
25	C	5.479293	6.370087	6.421423	6.158053	5.865494
26	H	6.000656	7.007941	7.283815	6.918848	6.475231
27	C	5.839156	6.357245	5.364934	5.181733	4.825610
28	C	4.719251	4.880382	4.535963	3.836092	3.254394
29	O	5.658656	5.868028	5.365497	4.597561	3.709290
30	C	3.025840	4.081867	6.074460	5.507750	5.437396
31	O	3.042074	4.311880	7.016780	6.380028	6.300547
		11	12	13	14	15
11	H	0.000000				
12	H	4.347181	0.000000			
13	C	3.328767	2.230887	0.000000		
14	H	4.340171	2.679367	1.079651	0.000000	
15	C	2.234606	3.333106	1.405643	2.219890	0.000000
16	H	2.695256	4.341705	2.218359	2.673740	1.080157
17	C	1.079032	3.338695	2.285387	3.329366	1.419470
18	H	5.406465	4.083479	5.097328	5.678533	5.497345
19	C	5.733546	4.605506	5.285083	5.752092	5.648424
20	C	6.023002	5.787870	6.107802	6.614307	6.200170
21	H	6.682906	4.213421	5.005955	5.061260	5.825417
22	H	5.943316	6.306929	6.590516	7.220287	6.499464
23	C	6.802007	6.443830	6.512314	6.829823	6.648608
24	H	7.387159	7.428350	7.308254	7.611817	7.314509
25	C	7.035077	5.864172	6.042835	6.170935	6.452567
26	H	7.776645	6.447052	6.487761	6.447132	6.960569
27	C	6.416963	4.676266	5.251326	5.451442	5.832699
28	C	4.734025	3.554074	2.995593	3.072922	3.499959
29	O	5.622185	3.824163	3.263344	2.938406	4.030242
30	C	5.498544	5.944552	5.049400	5.237693	4.901857
31	O	6.101753	6.871391	5.714037	5.803885	5.441278
		16	17	18	19	20
16	H	0.000000				
17	C	2.233093	0.000000			
18	H	6.349973	4.912739	0.000000		
19	C	6.376057	5.244073	1.079258	0.000000	

20	C	6.776607	5.742832	2.228070	1.414929	0.000000
21	H	6.519471	5.897981	2.693428	2.235108	3.336747
22	H	7.062576	5.847329	2.682450	2.225664	1.078298
23	C	7.075546	6.461840	3.333161	2.289808	1.415212
24	H	7.623449	7.149801	4.342037	3.332388	2.226362
25	C	6.915228	6.488368	3.340395	2.298503	2.302588
26	H	7.325091	7.182494	4.350246	3.341960	3.345480
27	C	6.479261	5.767834	2.237396	1.424253	2.294192
28	C	3.958625	3.948053	3.643305	3.226891	3.853247
29	O	4.389258	4.747278	4.480103	4.044750	4.759265
30	C	4.963297	5.159306	4.622961	3.811454	3.428230
31	O	5.280729	5.842745	5.763053	4.935013	4.440397

		21	22	23	24	25
21	H	0.000000				
22	H	4.345837	0.000000			
23	C	3.332171	2.227336	0.000000		
24	H	4.341886	2.681694	1.079410	0.000000	
25	C	2.219054	3.345169	1.427659	2.239186	0.000000
26	H	2.675081	4.354989	2.241790	2.700336	1.079017
27	C	1.079328	3.336781	2.288437	3.331475	1.409163
28	C	2.750328	4.636302	3.804444	4.578760	3.160160
29	O	2.886782	5.639785	4.500262	5.239457	3.554063
30	C	4.204680	3.990238	2.827444	2.994154	2.942374
31	O	5.212111	4.918192	3.681049	3.564032	3.824562

		26	27	28	29	30
26	H	0.000000				
27	C	2.223565	0.000000			
28	C	3.519417	2.732970	0.000000		
29	O	3.598049	3.198686	1.168644	0.000000	
30	C	3.184395	3.555984	2.584621	3.247985	0.000000
31	O	3.827208	4.608943	3.468141	3.920265	1.143560

		31
31	O	0.000000

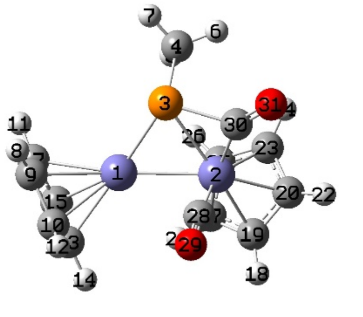

2Q -3521.206102 1.29 C1
WBI 0.26

		1	2	3	4	5
1	Fe	0.000000				
2	Fe	2.511317	0.000000			
3	P	2.366400	2.408815	0.000000		
4	C	3.567369	3.440446	1.858892	0.000000	
5	H	3.504938	3.518905	2.503707	1.090054	0.000000
6	H	4.404336	3.664871	2.458254	1.092031	1.777944
7	H	4.102743	4.411553	2.423726	1.092181	1.769072
8	H	3.073281	5.333753	4.023050	5.163069	5.380865
9	C	2.353858	4.783721	3.901536	5.045266	5.086215
10	C	2.309425	4.441417	4.243582	5.651367	5.675599
11	H	3.013946	5.333694	4.283017	4.579444	4.257717
12	H	3.025401	4.761327	4.655920	6.261178	6.419531

Charge and spin density

		1	2
1	Fe	0.471900	3.628438
2	Fe	-0.047611	0.229583
3	P	-0.027591	-0.024580
4	C	-0.022270	0.002613
9	C	-0.000386	0.071538
10	C	-0.048976	0.030910
13	C	-0.074596	0.029333
15	C	-0.095714	0.019507
17	C	-0.044672	0.032380
19	C	-0.001496	-0.026009
20	C	0.026165	-0.005879
23	C	0.003193	0.014302
25	C	-0.030595	-0.010482
27	C	0.047400	0.030311
28	C	0.094654	-0.017877
29	O	-0.114890	-0.026470
30	C	0.068364	0.007508
31	O	-0.202881	0.014871

13	C	2.235337	4.208333	4.568322	5.741123	5.516171
14	H	2.943055	4.378461	5.233086	6.440566	6.176420
15	C	2.224837	4.420059	4.442061	5.189700	4.776242
16	H	2.941301	4.776285	5.037869	5.497427	4.884768
17	C	2.295118	4.765089	4.024505	4.718735	4.470839
18	H	4.434840	2.833656	5.202012	6.187830	6.045134
19	C	3.902103	2.085938	4.476964	5.251919	5.046999
20	C	4.439476	2.080848	4.256410	4.742276	4.645551
21	H	3.087321	2.924272	4.649745	5.295771	4.783976
22	H	5.298665	2.820120	4.829405	5.343177	5.393504
23	C	4.104824	2.098282	3.702889	3.692237	3.425234
24	H	4.762496	2.846523	3.871687	3.430592	3.228822
25	C	3.267027	2.123023	3.605300	3.654498	3.104671
26	H	3.325365	2.887267	3.701366	3.358090	2.545916
27	C	3.123621	2.136689	4.128921	4.699648	4.266337
28	C	2.745910	1.747226	3.043553	4.646433	4.930402
29	O	3.467305	2.892322	3.898561	5.630438	5.985603
30	C	3.515628	1.825905	1.922126	2.895406	3.490785
31	O	4.678221	2.886239	2.848947	3.480664	4.201476

		6	7	8	9	10
6	H	0.000000				
7	H	1.765927	0.000000			
8	H	6.140085	5.033918	0.000000		
9	C	6.039691	5.106476	1.079909	0.000000	
10	C	6.540736	5.941095	2.221702	1.409973	0.000000
11	H	5.665642	4.488398	2.680265	2.225897	3.330269
12	H	7.068239	6.562724	2.677032	2.223885	1.080132
13	C	6.612646	6.188695	3.332637	2.289600	1.418191
14	H	7.223249	7.005405	4.341435	3.332411	2.230328
15	C	6.148460	5.544916	3.336654	2.293455	2.295670
16	H	6.415478	5.908050	4.346618	3.336774	3.340514
17	C	5.769652	4.812350	2.224630	1.412354	2.286711
18	H	6.317019	7.195854	7.232358	6.460039	5.571194
19	C	5.363475	6.287921	6.874297	6.116027	5.434348
20	C	4.577797	5.823443	7.386952	6.762309	6.288061
21	H	5.790387	6.175523	5.938370	4.979774	4.368670
22	H	5.003143	6.403771	8.124363	7.574145	7.063223
23	C	3.532915	4.781927	7.042658	6.443962	6.249334
24	H	2.994707	4.481222	7.525552	7.031385	7.000817
25	C	3.892194	4.667988	6.271689	5.541321	5.368593
26	H	3.746703	4.252853	6.109666	5.391800	5.454488
27	C	5.033090	5.676274	6.167014	5.317235	4.800262
28	C	4.951525	5.431349	4.951020	4.483197	3.791566
29	O	5.949199	6.322187	5.104731	4.720235	3.833140
30	C	2.760795	3.743839	5.654785	5.437715	5.370308
31	O	3.000660	4.224779	6.610213	6.498721	6.455932

		11	12	13	14	15
11	H	0.000000				
12	H	4.339929	0.000000			
13	C	3.337437	2.230100	0.000000		
14	H	4.350331	2.684662	1.080296	0.000000	
15	C	2.232171	3.339445	1.421068	2.235958	0.000000
16	H	2.690000	4.351405	2.236546	2.697818	1.080397
17	C	1.080392	3.330385	2.292777	3.338287	1.420050
18	H	7.216166	5.675303	4.916195	4.417302	5.531348
19	C	6.612816	5.703027	4.763166	4.460184	5.126156
20	C	7.110058	6.565036	5.786575	5.661977	5.985690
21	H	5.118070	4.953150	3.267645	2.965998	3.342324
22	H	8.053684	7.220559	6.673641	6.518718	6.970960
23	C	6.384744	6.721979	5.725540	5.807196	5.589463
24	H	6.807412	7.492438	6.575845	6.759143	6.319163
25	C	5.293498	5.996893	4.649228	4.757318	4.352261
26	H	4.722060	6.229276	4.710577	4.976952	4.088951
27	C	5.469424	5.317072	3.928550	3.758264	3.988359
28	C	5.731537	3.719408	3.894904	3.950463	4.620127
29	O	6.313111	3.438402	4.148574	4.102301	5.132842
30	C	6.028274	5.556229	5.536337	5.890781	5.689271
31	O	7.091071	6.555733	6.690946	7.022075	6.860956
		16	17	18	19	20
16	H	0.000000				
17	C	2.234000	0.000000			
18	H	5.646824	6.437178	0.000000		
19	C	5.185607	5.946912	1.079727	0.000000	
20	C	6.061494	6.586257	2.227061	1.417581	0.000000
21	H	3.136515	4.462978	2.693798	2.238864	3.350157
22	H	7.103897	7.516194	2.684252	2.231568	1.078737
23	C	5.570347	6.053066	3.331341	2.288273	1.418146
24	H	6.297795	6.614650	4.341592	3.331944	2.230299
25	C	4.210824	4.956910	3.329064	2.285539	2.295693
26	H	3.813456	4.581952	4.340672	3.330258	3.338085
27	C	3.894705	4.883806	2.233424	1.423762	2.307653
28	C	5.241706	4.933753	2.911453	2.793314	3.207498
29	O	5.856755	5.423631	3.421716	3.618701	4.145451
30	C	6.178779	5.625245	4.297505	3.634748	3.003242
31	O	7.344640	6.740567	5.001054	4.400834	3.497546
		21	22	23	24	25
21	H	0.000000				
22	H	4.359328	0.000000			
23	C	3.344682	2.231806	0.000000		
24	H	4.352773	2.689113	1.079081	0.000000	
25	C	2.232057	3.339012	1.419304	2.231326	0.000000
26	H	2.687349	4.347780	2.229236	2.683714	1.079784

		1	2	3	4	5
27	C	1.079301	3.350167	2.300922	3.342490	1.415244
28	C	3.617767	3.625138	3.741444	4.494146	3.719237
29	O	4.397241	4.405652	4.841164	5.593779	4.819599
30	C	4.731167	3.236769	2.866382	2.994004	3.458509
31	O	5.778555	3.383880	3.457112	3.305094	4.353972

		26	27	28	29	30
26	H	0.000000				
27	C	2.225915	0.000000			
28	C	4.466625	3.182879	0.000000		
29	O	5.566188	4.121614	1.146929	0.000000	
30	C	4.012032	3.897623	2.529447	3.431447	0.000000
31	O	4.921680	4.869084	3.422241	4.150057	1.177360

		31
31	O	0.000000

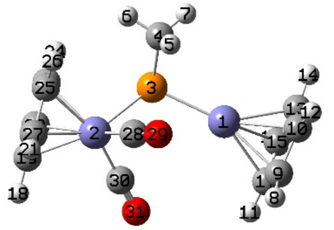

3Q -3521.205183 1.87 C1
WBI 0.03

Charge and spin density

		1	2
1	Fe	0.449432	3.780363
2	Fe	0.201135	0.067038
3	P	-0.226669	-0.040987
4	C	-0.047388	0.019895
9	C	-0.070867	0.021361
10	C	-0.081512	0.028530
13	C	-0.029804	0.026840
15	C	-0.036181	0.072098
17	C	-0.032592	0.027173
19	C	-0.009393	0.002067
20	C	0.030526	-0.002369
23	C	0.073692	0.003955
25	C	0.028672	-0.001225
27	C	0.031314	-0.001993
28	C	-0.005979	-0.010675
29	O	-0.129405	0.004570
30	C	-0.021640	0.003144
31	O	-0.123340	0.000216

		1	2	3	4	5
1	Fe	0.000000				
2	Fe	3.910744	0.000000			
3	P	2.317470	2.339556	0.000000		
4	C	3.318123	3.375892	1.870306	0.000000	
5	H	3.498938	3.554587	2.526470	1.092258	0.000000
6	H	4.318101	3.510811	2.469489	1.093475	1.770869
7	H	3.397575	4.357676	2.445250	1.093336	1.770115
8	H	2.943981	5.563979	4.945420	5.755428	5.456725
9	C	2.232796	5.500506	4.464178	5.317807	5.182952
10	C	2.221078	5.917590	4.426768	4.860493	4.724178
11	H	3.014481	5.577608	4.796028	6.239886	6.406017
12	H	2.932089	6.329736	4.887800	4.937196	4.607808
13	C	2.276750	6.179395	4.330877	4.898816	5.063048
14	H	2.996949	6.769069	4.701091	4.985312	5.224496
15	C	2.326310	5.954217	4.315181	5.376213	5.677040
16	H	3.050641	6.351101	4.648314	5.821599	6.288744
17	C	2.295869	5.524538	4.393297	5.612598	5.740687
18	H	6.376351	2.843536	5.003814	6.142829	6.325808
19	C	5.883298	2.095509	4.241567	5.216307	5.439726
20	C	5.485880	2.112755	3.567326	4.628333	5.124627
21	H	6.492391	2.840120	5.017194	5.360660	5.164491
22	H	5.666258	2.866723	3.882527	5.173463	5.804343
23	C	5.307241	2.119792	3.098952	3.627118	4.147251
24	H	5.300193	2.856499	2.990938	3.303042	4.039016
25	C	5.593821	2.099419	3.576165	3.697327	3.844894
26	H	5.873126	2.855290	3.906692	3.484868	3.466610
27	C	5.951365	2.094391	4.251514	4.742281	4.752039
28	C	3.562292	1.747111	2.740822	3.170990	2.804714
29	O	3.817694	2.891585	3.505368	3.541918	2.842820
30	C	3.471088	1.749208	2.849412	4.423776	4.617075
31	O	3.658319	2.892650	3.636745	5.323449	5.518130

		6	7	8	9	10
6	H	0.000000				
7	H	1.761514	0.000000			
8	H	6.775820	5.949042	0.000000		
9	C	6.367293	5.362030	1.080117	0.000000	
10	C	5.950492	4.651428	2.234425	1.420838	0.000000
11	H	7.150619	6.370736	2.687486	2.230140	3.338442
12	H	6.022495	4.687220	2.692763	2.234417	1.080136
13	C	5.933824	4.490069	3.339427	2.294823	1.419855
14	H	5.967598	4.354803	4.351077	3.339390	2.232845
15	C	6.344537	5.135508	3.334892	2.291300	2.292737
16	H	6.697093	5.523657	4.344753	3.334860	3.336516
17	C	6.594525	5.623926	2.231819	1.417947	2.294313
18	H	6.047634	7.124850	7.472576	7.593543	8.337433
19	C	5.031780	6.212947	7.301231	7.335519	7.914245
20	C	4.359037	5.505575	7.409345	7.233343	7.657488
21	H	5.149229	6.453163	7.481429	7.728225	8.213220
22	H	4.960076	5.921385	7.670595	7.405420	7.870020
23	C	3.137348	4.523937	7.482339	7.249509	7.427748
24	H	2.669167	4.021305	7.773218	7.402975	7.407775
25	C	3.217984	4.738132	7.409303	7.346933	7.534356
26	H	2.874533	4.499548	7.681535	7.625251	7.652691
27	C	4.476274	5.815452	7.307372	7.410687	7.847224
28	C	3.603659	4.140387	4.633141	4.767776	5.107553
29	O	4.088675	4.400028	4.347849	4.636277	4.892321
30	C	4.849903	5.208189	4.640239	4.615142	5.382687
31	O	5.850962	5.991917	4.346217	4.354555	5.346123

		11	12	13	14	15
11	H	0.000000				
12	H	4.349578	0.000000			
13	C	3.332262	2.234201	0.000000		
14	H	4.341687	2.691815	1.080222	0.000000	
15	C	2.225280	3.336667	1.413335	2.226559	0.000000
16	H	2.679439	4.347098	2.225779	2.680998	1.080227
17	C	1.080276	3.338601	2.288622	3.332078	1.411832
18	H	7.072294	8.854452	8.596679	9.296352	8.055596
19	C	7.024797	8.374889	8.134084	8.747164	7.722211
20	C	6.831133	8.183639	7.639865	8.133101	7.211500
21	H	7.976352	8.410483	8.744547	9.353044	8.624402
22	H	6.704950	8.508124	7.691072	8.173659	7.104522
23	C	7.229323	7.813547	7.418311	7.775183	7.240602
24	H	7.423163	7.788812	7.234111	7.450527	7.126376
25	C	7.629414	7.765016	7.775433	8.182191	7.747646
26	H	8.184162	7.740543	7.954693	8.276026	8.113412
27	C	7.518563	8.132087	8.218329	8.780665	8.040661
28	C	5.479050	5.281627	5.727974	6.358052	5.800085

29	O	5.749638	4.857046	5.748251	6.376682	6.019692
30	C	4.184253	6.011403	5.627839	6.408763	5.074288
31	O	3.471352	6.096559	5.563746	6.444416	4.784488
		16	17	18	19	20
16	H	0.000000				
17	C	2.224358	0.000000			
18	H	8.304039	7.404341	0.000000		
19	C	7.983770	7.208203	1.079530	0.000000	
20	C	7.327842	6.943333	2.239597	1.428452	0.000000
21	H	9.116776	8.000909	2.677576	2.223109	3.342542
22	H	7.069396	6.909499	2.700340	2.242325	1.078809
23	C	7.423853	7.129616	3.335147	2.292429	1.410788
24	H	7.223339	7.227348	4.343172	3.334413	2.218499
25	C	8.106834	7.481477	3.336555	2.292366	2.292483
26	H	8.538636	7.912451	4.345494	3.334660	3.334822
27	C	8.439172	7.537762	2.221529	1.409327	2.298394
28	C	6.461127	5.234809	4.091092	3.493490	3.823094
29	O	6.818619	5.394942	5.061741	4.531050	4.952461
30	C	5.441310	4.395306	3.030980	2.857624	2.961857
31	O	5.104393	3.933005	3.618037	3.720673	3.847128
		21	22	23	24	25
21	H	0.000000				
22	H	4.351763	0.000000			
23	C	3.339676	2.223671	0.000000		
24	H	4.349863	2.672019	1.078841	0.000000	
25	C	2.238939	3.334620	1.415092	2.227092	0.000000
26	H	2.696615	4.343031	2.228826	2.685484	1.079039
27	C	1.079289	3.340691	2.296662	3.339933	1.426681
28	C	3.133088	4.613078	3.526119	4.119598	2.938633
29	O	3.749144	5.757920	4.567450	5.098875	3.815788
30	C	3.984534	3.215584	3.561065	4.189957	3.800671
31	O	4.923504	3.861766	4.613561	5.192488	4.927724
		26	27	28	29	30
26	H	0.000000				
27	C	2.238450	0.000000			
28	C	3.165010	2.927349	0.000000		
29	O	3.788785	3.801584	1.144928	0.000000	
30	C	4.599176	3.419678	2.547308	3.486598	0.000000
31	O	5.740966	4.440787	3.481049	4.250666	1.143605
		31				
31	O	0.000000				

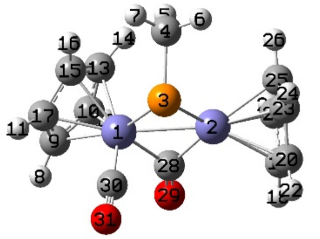

4T -3521.203157 3.14 C1
WBI 0.28

Charge and spin density

		1	2
1	Fe	-0.098100	-0.161965
2	Fe	0.291734	2.520002
3	P	-0.151876	-0.265727
4	C	-0.057798	0.020503
9	C	0.008662	-0.000562
10	C	0.045009	0.012949
13	C	-0.028184	-0.008714
15	C	0.088136	-0.001290
17	C	0.047244	0.003072
19	C	0.014067	0.004464
20	C	0.016272	0.037149
23	C	0.014127	-0.031436
25	C	-0.088702	0.041972
27	C	-0.050264	-0.016870
28	C	0.204461	-0.119088
29	O	-0.197076	-0.047256
30	C	0.049266	0.006682
31	O	-0.106978	0.006116

		1	2	3	4	5
1	Fe	0.000000				
2	Fe	2.528674	0.000000			
3	P	2.266453	2.254633	0.000000		
4	C	3.411507	3.288670	1.862708	0.000000	
5	H	3.434456	3.318974	2.529020	1.090705	0.000000
6	H	4.300726	3.590632	2.421052	1.093600	1.772806
7	H	3.862124	4.256941	2.458448	1.093838	1.778886
8	H	2.846720	5.049440	5.053614	6.039011	5.875700
9	C	2.098718	4.423102	4.264473	5.056444	4.843566
10	C	2.129507	3.853087	4.139381	4.619738	4.144597
11	H	2.819546	5.286254	4.099064	4.833555	4.990249
12	H	2.879457	4.052895	4.843670	5.330519	4.752436
13	C	2.117875	3.612789	3.435779	3.434180	2.863832
14	H	2.887794	3.650182	3.691461	3.244672	2.392746
15	C	2.095932	4.105957	3.108544	3.189470	2.997248
16	H	2.856552	4.533130	3.092141	2.729738	2.684997
17	C	2.073992	4.561517	3.678074	4.313561	4.283380
18	H	4.840776	2.951264	5.095632	6.186833	6.104503
19	C	4.489170	2.222790	4.365361	5.296931	5.237004
20	C	4.614470	2.241000	3.847952	4.862177	5.071885
21	H	4.757568	2.923388	4.993832	5.281661	4.772498
22	H	5.042460	2.964669	4.211398	5.453945	5.823493
23	C	4.579167	2.170842	3.333288	3.862020	4.068509
24	H	5.039645	2.906860	3.344523	3.693623	4.116400
25	C	4.493056	2.166595	3.683521	3.793254	3.602366
26	H	4.873971	2.888947	3.960275	3.535525	3.170473
27	C	4.428744	2.191988	4.292133	4.738618	4.432059
28	C	1.936614	1.896990	3.274646	4.504210	4.378816
29	O	2.895572	2.902491	4.429967	5.656180	5.460833
30	C	1.755998	3.278596	2.759839	4.425491	4.793738
31	O	2.896677	4.137745	3.555360	5.304963	5.799285

		6	7	8	9	10
6	H	0.000000				
7	H	1.756279	0.000000			
8	H	7.031628	6.247762	0.000000		
9	C	6.075417	5.254068	1.078981	0.000000	
10	C	5.582657	5.006194	2.224184	1.410080	0.000000
11	H	5.864753	4.601712	2.699832	2.238678	3.332174
12	H	6.203785	5.847883	2.674616	2.219097	1.079127
13	C	4.429596	3.755466	3.337679	2.294946	1.417600
14	H	4.109380	3.687722	4.344065	3.335669	2.228207
15	C	4.280613	3.141840	3.343871	2.301012	2.293651
16	H	3.798145	2.359073	4.352104	3.342482	3.336177
17	C	5.376984	4.259223	2.240755	1.426666	2.288964
18	H	6.299773	7.193355	6.412803	6.190588	5.595550

19	C	5.315004	6.337257	6.475578	6.082052	5.409060
20	C	4.696194	5.861724	6.949556	6.491672	5.988491
21	H	5.401286	6.326897	6.497271	5.957496	4.856730
22	H	5.273073	6.367232	7.278773	6.920306	6.610192
23	C	3.501377	4.892581	7.210307	6.561326	5.936704
24	H	3.092045	4.619923	7.798870	7.099766	6.570622
25	C	3.572178	4.885774	6.975613	6.258466	5.366649
26	H	3.222876	4.585654	7.369419	6.546371	5.552087
27	C	4.753789	5.816001	6.507550	5.944019	5.002516
28	C	5.122201	5.270694	3.496990	3.141845	2.731981
29	O	6.265927	6.416420	3.526130	3.481470	3.119548
30	C	5.165958	4.750191	3.173935	2.931749	3.573085
31	O	5.957237	5.553904	3.803854	3.802515	4.618792
		11	12	13	14	15
11	H	0.000000				
12	H	4.341849	0.000000			
13	C	3.334256	2.228356	0.000000		
14	H	4.345157	2.681543	1.078952	0.000000	
15	C	2.225560	3.336298	1.419850	2.234507	0.000000
16	H	2.678157	4.345929	2.232939	2.695219	1.077631
17	C	1.079362	3.332024	2.291186	3.335104	1.414619
18	H	7.559609	5.255312	5.925199	5.934665	6.694522
19	C	7.281360	5.203622	5.480264	5.380629	6.210412
20	C	7.279388	6.015395	5.845358	5.783481	6.302792
21	H	7.489197	4.416512	4.827238	4.385569	5.941654
22	H	7.536477	6.692814	6.542470	6.606199	6.840913
23	C	7.169896	6.083868	5.465315	5.222011	5.885223
24	H	7.400434	6.850792	5.939540	5.696575	6.156611
25	C	7.168429	5.372007	4.858919	4.401618	5.570038
26	H	7.378623	5.602111	4.824193	4.169469	5.555118
27	C	7.229659	4.759427	4.861628	4.511054	5.776300
28	C	4.566210	2.748384	3.220955	3.644127	3.840591
29	O	5.201707	2.789343	3.973783	4.411474	4.703193
30	C	2.950200	4.229717	3.814641	4.635813	3.402081
31	O	3.498659	5.233464	4.931840	5.772354	4.404628
		16	17	18	19	20
16	H	0.000000				
17	C	2.224914	0.000000			
18	H	7.324574	6.835342	0.000000		
19	C	6.719761	6.540639	1.079578	0.000000	
20	C	6.633208	6.674153	2.220965	1.405386	0.000000
21	H	6.481796	6.539846	2.681478	2.233622	3.334067
22	H	7.157270	7.058577	2.677282	2.219110	1.080115
23	C	6.026597	6.532230	3.329463	2.285082	1.417910
24	H	6.118786	6.864987	4.338188	3.327090	2.229869
25	C	5.783955	6.367556	3.334024	2.293157	2.298829

26	H	5.638076	6.547330	4.347714	3.339985	3.341081
27	C	6.230615	6.364946	2.233313	1.425639	2.293956
28	C	4.645658	3.793676	3.073291	2.984418	3.551255
29	O	5.609872	4.451390	2.963194	3.267090	4.113529
30	C	3.979347	2.800353	5.100531	4.917159	4.780200
31	O	4.896168	3.639792	5.605622	5.524308	5.254133

		21	22	23	24	25
21	H	0.000000				
22	H	4.343694	0.000000			
23	C	3.339196	2.229218	0.000000		
24	H	4.349841	2.684638	1.079060	0.000000	
25	C	2.230081	3.342468	1.425090	2.237190	0.000000
26	H	2.696532	4.350707	2.234578	2.690275	1.080019
27	C	1.079790	3.338236	2.292210	3.334841	1.411233
28	C	3.427115	4.046948	3.966939	4.763287	3.783970
29	O	3.646806	4.533943	4.778196	5.668387	4.533940
30	C	5.846476	4.825071	5.065475	5.407994	5.420799
31	O	6.734746	5.064962	5.694450	5.956048	6.249165

		26	27	28	29	30
26	H	0.000000				
27	C	2.229347	0.000000			
28	C	4.462387	3.170631	0.000000		
29	O	5.267960	3.605829	1.168900	0.000000	
30	C	6.008629	5.328734	2.623352	3.320014	0.000000
31	O	6.913551	6.147654	3.515389	4.023297	1.141290

		31
31	O	0.000000

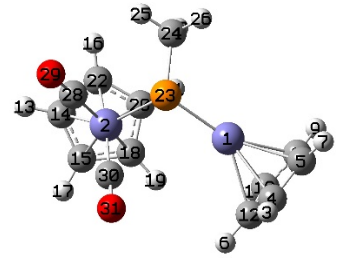

5Q -3521.201946 3.90 C1
WBI 0.04

Charge and spin density

		1	2
1	Fe	0.439605	3.765647
2	Fe	0.090965	0.093801
4	C	-0.025088	0.052615
5	C	-0.059891	0.027006
8	C	-0.061724	0.013439
10	C	-0.078495	0.026723

		1	2	3	4	5
1	Fe	0.000000				
2	Fe	3.596735	0.000000			
3	H	3.049855	5.860008	0.000000		
4	C	2.327476	5.464959	1.080172	0.000000	
5	C	2.278864	5.850302	2.225808	1.412855	0.000000
6	H	3.024517	4.839943	2.675068	2.223226	3.331341
7	H	2.996138	6.546565	2.680925	2.225884	1.080204
8	C	2.225329	5.577717	3.336527	2.292670	1.419521
9	H	2.941282	6.104650	4.346826	3.336283	2.233635
10	C	2.240260	4.977763	3.334007	2.290706	2.294240
11	H	2.961425	5.025060	4.344768	3.334966	3.338463
12	C	2.301018	4.899769	2.223520	1.411724	2.288441
13	H	6.311210	2.840925	8.632961	8.201491	8.561487
14	C	5.357277	2.094858	7.775888	7.270892	7.584277
15	C	4.875547	2.099581	7.012677	6.495576	6.968510
16	H	5.235927	2.852172	8.092430	7.493621	7.408945
17	H	5.520808	2.849068	7.287333	6.855832	7.502675
18	C	3.759033	2.114331	6.111720	5.445156	5.764199

12	C	-0.064626	0.033250
14	C	0.005466	0.001997
15	C	0.008290	-0.002253
18	C	0.000039	0.009187
20	C	0.038900	-0.001466
22	C	0.052227	-0.004420
23	P	-0.169030	-0.014985
24	C	-0.045289	0.016076
28	C	0.029325	-0.003183
29	O	-0.092414	-0.003617
30	C	0.026467	-0.005288
31	O	-0.094727	-0.004529

19	H	3.510746	2.875855	5.556281	4.836423	5.234259
20	C	3.633289	2.116393	6.440236	5.720173	5.733931
21	H	3.227242	2.864632	6.201151	5.386898	5.152244
22	C	4.701249	2.097268	7.458393	6.851902	6.910033
23	P	2.296922	2.361101	4.613689	4.286479	4.354758
24	C	3.437198	3.367623	6.072897	5.613969	5.252403
25	H	4.404369	3.550815	6.912091	6.543785	6.275435
26	H	3.615982	4.352407	5.998195	5.579001	5.024449
27	H	3.601329	3.487061	6.504467	5.885940	5.416520
28	C	4.672404	1.753770	6.685532	6.494441	6.861223
29	O	5.579438	2.895062	7.429225	7.340010	7.687473
30	C	3.593451	1.755051	4.924357	4.780332	5.556584
31	O	4.035794	2.896518	4.595540	4.656449	5.668100
		6	7	8	9	10
6	H	0.000000				
7	H	4.340159	0.000000			
8	C	3.340411	2.232350	0.000000		
9	H	4.352056	2.690922	1.080119	0.000000	
10	C	2.232690	3.338878	1.421400	2.235465	0.000000
11	H	2.693318	4.349964	2.233753	2.692020	1.080478
12	C	1.080416	3.331768	2.295123	3.339424	1.417926
13	H	7.217879	9.290805	8.068192	8.438304	7.344035
14	C	6.308424	8.342052	7.028519	7.387073	6.299416
15	C	5.215810	7.841336	6.390139	6.864683	5.443978
16	H	7.136280	7.963422	6.804323	6.895748	6.500312
17	H	5.308721	8.422865	6.987842	7.554310	5.903694
18	C	4.325631	6.656968	5.052756	5.472493	4.143516
19	H	3.492066	6.212545	4.468199	4.965181	3.366638
20	C	5.116023	6.479734	4.980458	5.192044	4.433457
21	H	5.121011	5.826621	4.296653	4.344129	3.994741
22	C	6.257400	7.578149	6.280212	6.484492	5.787574
23	P	4.704011	4.756249	4.442505	4.936741	4.427243
24	C	6.249383	5.448743	5.120976	5.240291	5.415400
25	H	7.076200	6.447377	6.194842	6.331924	6.418991
26	H	6.585780	5.006981	5.043223	5.076342	5.608980
27	H	6.358871	5.700422	4.996468	4.954466	5.253578
28	C	6.185205	7.369578	6.830734	7.344051	6.437542
29	O	7.176864	8.091376	7.767468	8.264219	7.470452
30	C	3.927960	6.298864	5.583838	6.364233	4.823031
31	O	3.670883	6.411709	5.880473	6.780136	5.054453
		11	12	13	14	15
11	H	0.000000				
12	C	2.233139	0.000000			
13	H	7.073191	7.435772	0.000000		
14	C	6.010922	6.468475	1.079463	0.000000	
15	C	5.088362	5.523261	2.221952	1.408631	0.000000

16	H	6.312700	6.952537	2.696070	2.238999	3.336385
17	H	5.529413	5.810761	2.676981	2.221477	1.079367
18	C	3.753455	4.443058	3.339586	2.295275	1.427571
19	H	2.879132	3.672278	4.348087	3.337206	2.239382
20	C	4.144328	4.955607	3.337874	2.294958	2.294052
21	H	3.755210	4.742025	4.346959	3.337615	3.337374
22	C	5.545601	6.175781	2.237589	1.426305	2.294231
23	P	4.909140	4.327832	4.996084	4.274733	4.332476
24	C	5.771500	5.705730	5.303497	4.659360	5.142420
25	H	6.739322	6.625815	5.053464	4.614366	5.344270
26	H	6.095896	5.909630	6.395382	5.742920	6.158707
27	H	5.432078	5.792258	5.127987	4.402545	4.908393
28	C	6.635580	6.217019	3.065726	2.884682	3.455771
29	O	7.726609	7.196000	3.653822	3.743704	4.477301
30	C	5.049543	4.256507	4.022252	3.446152	2.881643
31	O	5.372210	4.193150	4.964862	4.466354	3.740639
		16	17	18	19	20
16	H	0.000000				
17	H	4.347010	0.000000			
18	C	3.335144	2.239157	0.000000		
19	H	4.344854	2.696578	1.079153	0.000000	
20	C	2.228458	3.336782	1.412200	2.226919	0.000000
21	H	2.681011	4.346793	2.223218	2.681585	1.078752
22	C	1.078864	3.338192	2.292848	3.336092	1.415022
23	P	3.893622	5.091476	3.731029	4.102205	3.234024
24	C	3.325597	6.094317	4.516727	5.077011	3.457912
25	H	3.159305	6.274339	4.990768	5.702725	3.921079
26	H	4.354505	7.093971	5.426368	5.861889	4.393298
27	H	2.812860	5.937771	4.162725	4.744371	2.910581
28	C	3.182127	4.036565	3.823787	4.627283	3.550619
29	O	3.810868	4.982836	4.947391	5.767857	4.594394
30	C	4.604601	3.066578	2.957576	3.209642	3.548857
31	O	5.745086	3.653514	3.835359	3.844390	4.595375
		21	22	23	24	25
21	H	0.000000				
22	C	2.224570	0.000000			
23	P	3.177939	3.611803	0.000000		
24	C	3.100790	3.555634	1.862451	0.000000	
25	H	3.780076	3.608190	2.457886	1.093059	0.000000
26	H	3.865260	4.618329	2.435775	1.093196	1.761983
27	H	2.368636	3.103940	2.522408	1.092488	1.772198
28	C	4.179076	2.943323	2.680358	3.372144	3.043810
29	O	5.169676	3.818434	3.415183	3.838137	3.223098
30	C	4.180309	3.807508	2.842636	4.468400	4.737340
31	O	5.175174	4.931983	3.627780	5.391951	5.688492
		26	27	28	29	30

26	H	0.000000				
27	H	1.770441	0.000000			
28	C	4.307248	3.842743	0.000000		
29	O	4.657530	4.442654	1.141773	0.000000	
30	C	5.248684	4.829563	2.553226	3.490322	0.000000
31	O	6.058507	5.833537	3.485991	4.254835	1.141819

		31
31	O	0.000000

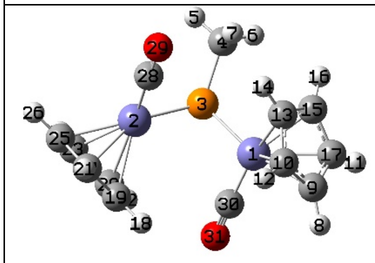

6Q -3521.198720 5.92 C1
WBI 0.06

Charge and spin density

		1	2
1	Fe	0.332777	2.195073
2	Fe	0.324635	2.225038
3	P	-0.276242	-0.067739
4	C	-0.058610	0.000509
9	C	0.005580	-0.034494
10	C	-0.036061	0.064813
13	C	0.067468	-0.048222
15	C	-0.056230	0.007052
17	C	-0.030371	-0.008535
19	C	-0.054540	0.045688
20	C	0.085884	-0.029936
23	C	-0.028033	0.060552
25	C	-0.049386	-0.036887
27	C	0.004491	-0.026995
28	C	0.023143	-0.129725
29	O	-0.120308	-0.032981
30	C	-0.003177	-0.133717
31	O	-0.131019	-0.049492

		1	2	3	4	5
1	Fe	0.000000				
2	Fe	3.491218	0.000000			
3	P	2.251899	2.256410	0.000000		
4	C	3.366125	3.434965	1.861470	0.000000	
5	H	4.326558	3.554612	2.451662	1.092822	0.000000
6	H	3.619691	4.377243	2.436234	1.092612	1.762627
7	H	3.469811	3.705289	2.520188	1.091481	1.770572
8	H	2.961693	6.089314	5.209298	6.142182	7.177959
9	C	2.217815	5.467055	4.437994	5.176064	6.229736
10	C	2.226407	4.793153	4.155901	4.729142	5.719792
11	H	2.940595	6.375267	4.625863	5.058840	6.119391
12	H	2.957755	4.875657	4.729932	5.404863	6.310101
13	C	2.145608	4.458799	3.492916	3.577122	4.580548
14	H	2.874419	4.278537	3.611753	3.349394	4.214853
15	C	2.137876	5.043188	3.446295	3.405341	4.493552
16	H	2.868420	5.367515	3.532938	2.997453	4.038590
17	C	2.208013	5.641719	4.086699	4.517068	5.603893
18	H	3.795477	2.983175	4.174308	5.806381	6.266705
19	C	4.071906	2.241867	3.843967	5.428057	5.716117
20	C	4.263311	2.192900	3.471173	5.139389	5.287491
21	H	5.292941	2.937725	5.014399	6.088228	6.240457
22	H	4.199857	2.930706	3.529965	5.312861	5.533513
23	C	5.173834	2.179863	3.851570	5.183372	5.041903
24	H	5.801570	2.893305	4.184084	5.363294	5.050832
25	C	5.478111	2.163987	4.337110	5.433176	5.282042
26	H	6.332768	2.894540	5.035646	5.849561	5.534487
27	C	4.860222	2.199565	4.328536	5.576151	5.690197
28	C	3.899676	1.767468	2.839375	3.047193	3.123860
29	O	4.518957	2.911560	3.653624	3.327344	3.369729
30	C	1.785182	3.684961	2.921641	4.577935	5.353471
31	O	2.927623	4.255963	3.771051	5.530427	6.209711

		6	7	8	9	10
6	H	0.000000				
7	H	1.772014	0.000000			
8	H	6.200021	6.004058	0.000000		
9	C	5.239738	4.973166	1.080092	0.000000	
10	C	5.037151	4.303769	2.225337	1.414527	0.000000

11	H	4.657414	5.110716	2.679139	2.228251	3.340616
12	H	5.863436	4.919824	2.677158	2.225754	1.079825
13	C	3.857007	3.022017	3.324901	2.282092	1.409858
14	H	3.819247	2.541896	4.336506	3.327074	2.225089
15	C	3.269760	3.123252	3.326145	2.283368	2.297434
16	H	2.594691	2.763557	4.339261	3.329130	3.337237
17	C	4.315178	4.416158	2.227909	1.417768	2.299484
18	H	6.530838	5.960398	5.039822	4.944919	4.542694
19	C	6.236260	5.661113	5.827538	5.582291	5.083130
20	C	5.858590	5.632511	6.389212	6.095234	5.787685
21	H	7.082057	5.992435	6.905924	6.548446	5.615749
22	H	5.859542	5.935132	6.214077	6.003855	5.948302
23	C	6.008879	5.687507	7.567869	7.147337	6.669368
24	H	6.110438	6.005141	8.336079	7.881982	7.476339
25	C	6.403103	5.686355	7.753514	7.291652	6.564222
26	H	6.864483	6.037946	8.669747	8.148465	7.316617
27	C	6.532987	5.666407	6.748937	6.380524	5.607161
28	C	4.124003	2.823886	6.403512	5.596585	4.597660
29	O	4.364144	2.762509	6.831476	5.951020	4.808144
30	C	4.829118	4.948446	3.287633	3.107266	3.481534
31	O	5.765624	5.994270	3.910131	3.987884	4.475574
		11	12	13	14	15
11	H	0.000000				
12	H	4.347356	0.000000			
13	C	3.344675	2.225983	0.000000		
14	H	4.353828	2.686723	1.079622	0.000000	
15	C	2.228295	3.343429	1.431338	2.241617	0.000000
16	H	2.693687	4.349972	2.238420	2.691730	1.079016
17	C	1.079514	3.341391	2.298148	3.339851	1.410445
18	H	6.416782	4.238929	5.092786	5.338169	5.794987
19	C	6.876060	4.862156	5.367319	5.458077	6.049331
20	C	6.965947	5.797675	5.865914	5.994545	6.270651
21	H	8.157831	5.099293	5.810007	5.537239	6.863430
22	H	6.622521	6.087409	6.068976	6.358192	6.251057
23	C	7.957283	6.666761	6.525563	6.440268	6.985475
24	H	8.454449	7.571711	7.209644	7.112709	7.525693
25	C	8.396457	6.382873	6.433891	6.172599	7.154864
26	H	9.250892	7.106415	7.076568	6.673108	7.850103
27	C	7.774466	5.268280	5.724885	5.548634	6.598280
28	C	6.591496	4.644384	3.948638	3.361493	4.773797
29	O	6.964276	4.824369	4.005976	3.164700	4.934135
30	C	3.742221	3.913808	3.853471	4.564978	3.797457
31	O	4.529819	4.785869	4.975308	5.684299	4.896401
		16	17	18	19	20
16	H	0.000000				
17	C	2.227520	0.000000			

		16	17	18	19	20
18	H	6.553508	5.731494	0.000000		
19	C	6.671940	6.186201	1.079549	0.000000	
20	C	6.728102	6.426225	2.228708	1.417728	0.000000
21	H	7.472274	7.274391	2.680872	2.222371	3.330042
22	H	6.689263	6.236183	2.683926	2.229786	1.078857
23	C	7.298172	7.373040	3.338654	2.296098	1.418521
24	H	7.710995	7.953884	4.346286	3.338265	2.228188
25	C	7.524262	7.663958	3.340021	2.294966	2.287952
26	H	8.144565	8.484504	4.346870	3.335589	3.333915
27	C	7.148800	6.975233	2.220178	1.405775	2.284725
28	C	4.986968	5.714606	4.190392	3.638098	3.931684
29	O	5.063353	6.034776	5.134456	4.668599	5.065656
30	C	4.482099	3.373656	3.013855	3.428287	3.428663
31	O	5.560605	4.325987	3.042855	3.508577	3.358746

		21	22	23	24	25
21	H	0.000000				
22	H	4.338984	0.000000			
23	C	3.336002	2.229170	0.000000		
24	H	4.349448	2.680392	1.079916	0.000000	
25	C	2.238563	3.329717	1.410117	2.226626	0.000000
26	H	2.689739	4.342911	2.228140	2.691809	1.079575
27	C	1.079629	3.326717	2.294677	3.341170	1.430213
28	C	3.339062	4.683343	3.715071	4.315402	3.187755
29	O	3.999979	5.821450	4.797874	5.354610	4.127576
30	C	5.236749	3.036094	4.657944	5.279691	5.248537
31	O	5.552799	2.718237	4.710481	5.284843	5.457384

		26	27	28	29	30
26	H	0.000000				
27	C	2.239118	0.000000			
28	C	3.450691	3.129782	0.000000		
29	O	4.188627	4.032638	1.144275	0.000000	
30	C	6.259858	4.616729	4.765051	5.644399	0.000000
31	O	6.518879	4.857121	5.583574	6.547418	1.142976

		31
31	O	0.000000

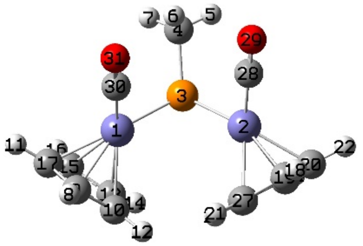

7Q -3521.197111 6.93 C1
WBI 0.06

Distance matrix (angstroms):

		1	2	3	4	5
1	Fe	0.000000				
2	Fe	3.590735	0.000000			
3	P	2.259350	2.248782	0.000000		
4	C	3.402985	3.437246	1.861091	0.000000	
5	H	4.362728	3.584180	2.446220	1.092488	0.000000
6	H	3.599558	3.684127	2.522954	1.091253	1.769153
7	H	3.566652	4.380251	2.438023	1.092446	1.764062
8	H	2.934740	5.539265	5.052125	6.160446	7.141803
9	C	2.195201	5.092608	4.345259	5.579638	6.544799

Charge and spin density

		1	2
1	Fe	0.337987	2.209588
2	Fe	0.350031	2.178855
3	P	-0.304269	-0.079075
4	C	-0.028308	0.001817
9	C	-0.026135	-0.027949
10	C	-0.020161	0.040997
13	C	0.056203	-0.023738
15	C	-0.037880	0.054949
17	C	-0.047730	-0.033231
19	C	-0.003851	-0.027610
20	C	-0.045302	-0.030740
23	C	-0.040615	0.050332
25	C	0.079113	-0.033181
27	C	-0.078445	0.052192
28	C	0.015148	-0.136977
29	O	-0.113359	-0.039169
30	C	0.019060	-0.127621
31	O	-0.111488	-0.029439

10	C	2.242770	4.351690	3.870300	5.451059	6.273474
11	H	2.894065	6.464072	5.003944	5.688166	6.745285
12	H	2.985495	4.139333	4.231583	5.914981	6.639151
13	C	2.194609	4.469201	3.449654	5.033088	5.801396
14	H	2.929599	4.392592	3.493781	5.182722	5.786496
15	C	2.179730	5.314511	3.797459	4.974410	5.867764
16	H	2.891708	5.887788	4.095847	5.049304	5.886008
17	C	2.165201	5.641409	4.314620	5.305275	6.316175
18	H	5.722136	2.942832	5.094607	6.084190	6.192694
19	C	5.124913	2.206252	4.377986	5.581506	5.688622
20	C	5.559240	2.170268	4.282042	5.426588	5.294189
21	H	4.023639	2.974699	4.323551	5.831523	6.314256
22	H	6.446592	2.899927	4.947428	5.825568	5.506955
23	C	4.996616	2.167433	3.737051	5.176184	5.115068
24	H	5.482718	2.881346	3.996088	5.350524	5.150358
25	C	4.064140	2.180525	3.453157	5.157249	5.391615
26	H	3.787338	2.917942	3.510482	5.344274	5.683234
27	C	4.205368	2.243877	3.933137	5.454751	5.774174
28	C	4.205792	1.768331	2.859495	3.034957	3.083188
29	O	4.916427	2.911211	3.688016	3.312956	3.287148
30	C	1.774365	3.927782	2.870880	3.133834	4.162244
31	O	2.916673	4.518360	3.691095	3.473714	4.429109

		6	7	8	9	10
6	H	0.000000				
7	H	1.770138	0.000000			
8	H	6.046985	6.351643	0.000000		
9	C	5.641974	5.699723	1.079607	0.000000	
10	C	5.708472	5.689752	2.223350	1.407305	0.000000
11	H	5.741867	5.398811	2.690250	2.238975	3.336627
12	H	6.141032	6.315539	2.679847	2.220243	1.079298
13	C	5.528105	5.088277	3.328536	2.283560	1.415238
14	H	5.837577	5.267451	4.340692	3.328044	2.232191
15	C	5.414140	4.757866	3.334690	2.293248	2.295436
16	H	5.607146	4.623550	4.348409	3.339840	3.337066
17	C	5.468607	5.150223	2.238415	1.429733	2.295817
18	H	5.963508	7.099527	6.448011	6.426341	5.748249
19	C	5.642028	6.551832	6.114767	5.927813	5.083550
20	C	5.693543	6.386608	7.049863	6.678191	5.695477
21	H	5.868962	6.596686	4.170616	4.089188	3.288919
22	H	6.066387	6.821071	8.049745	7.691282	6.744464
23	C	5.671476	5.984661	6.664806	6.112489	4.969297
24	H	6.005868	6.062745	7.389987	6.725277	5.534524
25	C	5.581962	5.880963	5.366467	4.834100	3.644353
26	H	5.873580	5.897665	5.032634	4.360088	3.035583
27	C	5.607825	6.285864	4.977040	4.723222	3.769471
28	C	2.816549	4.115380	6.133962	5.868686	5.496554

29	O	2.777136	4.355368	6.738845	6.578315	6.389824
30	C	2.766588	3.408170	3.365960	3.149299	3.664293
31	O	2.759014	3.779567	4.045537	4.062095	4.705485
		11	12	13	14	15
11	H	0.000000				
12	H	4.346944	0.000000			
13	C	3.334074	2.227586	0.000000		
14	H	4.340632	2.690407	1.079369	0.000000	
15	C	2.227051	3.338466	1.419597	2.226085	0.000000
16	H	2.691141	4.345683	2.228665	2.673888	1.079848
17	C	1.079484	3.340071	2.288148	3.328351	1.409150
18	H	8.262319	5.059804	6.439612	6.468014	7.459533
19	C	7.760146	4.412107	5.625691	5.540505	6.720608
20	C	8.350009	5.128777	5.910700	5.592048	7.024421
21	H	6.172360	2.455763	4.220921	4.409369	5.329315
22	H	9.287330	6.203126	6.906577	6.553417	7.973634
23	C	7.717023	4.484823	4.940621	4.443978	6.120314
24	H	8.162852	5.160737	5.244396	4.568230	6.382147
25	C	6.603719	3.076461	3.814111	3.463592	5.080778
26	H	6.071015	2.569156	3.005638	2.518241	4.356652
27	C	6.672146	3.032698	4.390279	4.354646	5.563242
28	C	6.941345	5.409825	5.703138	5.823007	6.245651
29	O	7.465068	6.356057	6.635097	6.837354	7.027786
30	C	3.449876	4.223712	3.944191	4.691101	3.715117
31	O	4.180589	5.185679	5.078919	5.829474	4.790958
		16	17	18	19	20
16	H	0.000000				
17	C	2.226058	0.000000			
18	H	8.264909	7.447956	0.000000		
19	C	7.480960	6.874882	1.079727	0.000000	
20	C	7.628451	7.431767	2.236671	1.428010	0.000000
21	H	6.267499	5.265067	2.671098	2.215631	3.338353
22	H	8.520181	8.399331	2.687080	2.236684	1.079523
23	C	6.646147	6.737966	3.334021	2.292392	1.409893
24	H	6.755171	7.184257	4.347836	3.339047	2.227382
25	C	5.754593	5.588980	3.327019	2.282307	2.289062
26	H	5.002356	5.013756	4.337588	3.325515	3.329002
27	C	6.389611	5.727014	2.220510	1.405600	2.295704
28	C	6.769562	6.327577	3.289547	3.102221	3.232104
29	O	7.517350	6.981359	3.927142	3.991988	4.174737
30	C	4.304669	3.195721	5.717301	5.410330	6.006195
31	O	5.330189	4.132634	5.998917	5.877728	6.539253
		21	22	23	24	25
21	H	0.000000				
22	H	4.343583	0.000000			
23	C	3.342243	2.228569	0.000000		

		1	2	3	4	5
24	H	4.350080	2.694202	1.079622	0.000000	
25	C	2.232281	3.335548	1.421424	2.230348	0.000000
26	H	2.695208	4.342392	2.228108	2.677145	1.078471
27	C	1.079597	3.335897	2.297622	3.339127	1.416626
28	C	4.080541	3.528034	3.744559	4.374749	3.906039
29	O	4.992762	4.281242	4.833236	5.430250	5.034411
30	C	4.574734	6.752786	5.830810	6.437026	5.072476
31	O	5.224236	7.181469	6.570643	7.220199	5.916294

		26	27	28	29	30
26	H	0.000000				
27	C	2.231626	0.000000			
28	C	4.652576	3.578835	0.000000		
29	O	5.784123	4.591865	1.143000	0.000000	
30	C	5.111362	4.822781	3.760901	4.084668	0.000000
31	O	6.086750	5.493333	3.902039	3.902823	1.142323

		31
31	O	0.000000

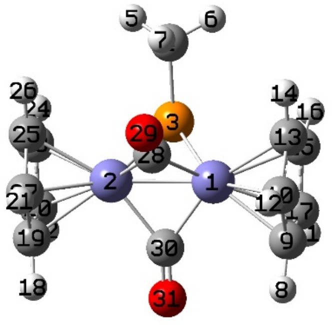

8T -3521.197037 6.98 C1
WBI 0.42

Charge and spin density

		1	2
1	Fe	0.011333	1.140128
2	Fe	0.010954	1.145874
3	P	-0.007694	-0.025867
4	C	-0.034721	-0.011397
9	C	0.013721	-0.023772
10	C	0.038468	-0.013852
13	C	-0.001596	0.015080
15	C	0.052070	-0.037841
17	C	-0.002220	0.021603
19	C	0.013878	-0.023896
20	C	-0.002259	0.021810
23	C	0.051821	-0.037760
25	C	-0.001961	0.014892
27	C	0.038059	-0.013607

		1	2	3	4	5
1	Fe	0.000000				
2	Fe	2.260080	0.000000			
3	P	2.247878	2.248750	0.000000		
4	C	3.313579	3.316240	1.855636	0.000000	
5	H	4.234312	3.738358	2.433848	1.094869	0.000000
6	H	3.737688	4.237369	2.433617	1.094870	1.756373
7	H	3.303163	3.307701	2.521150	1.090352	1.778331
8	H	2.870066	4.551257	4.919362	6.129730	7.082131
9	C	2.132681	4.164913	4.178067	5.231336	6.231534
10	C	2.132753	4.162342	4.217530	4.784748	5.838130
11	H	2.859901	4.611228	3.789293	5.171224	6.035185
12	H	2.870503	4.547252	4.983107	5.391804	6.413405
13	C	2.120912	4.190175	3.572110	3.788739	4.881633
14	H	2.863100	4.608450	3.926297	3.609210	4.676954
15	C	2.121529	4.219430	3.061758	3.690904	4.725755
16	H	2.884974	4.688600	3.032986	3.442416	4.394161
17	C	2.120477	4.193782	3.495906	4.649394	5.615957
18	H	4.550663	2.869640	4.918262	6.132640	6.363029
19	C	4.164400	2.132214	4.177537	5.235064	5.377544
20	C	4.192658	2.120106	3.494499	4.651947	4.624071
21	H	4.547892	2.870046	4.984737	5.398410	5.615335
22	H	4.609621	2.859637	3.786592	5.171963	5.123977
23	C	4.218718	2.121268	3.062055	3.695500	3.467462
24	H	4.687435	2.884667	3.032912	3.446090	2.951946
25	C	4.190037	2.120356	3.573798	3.795693	3.705387
26	H	4.608585	2.862606	3.929023	3.617914	3.435078
27	C	4.162438	2.132179	4.218626	4.790732	4.920016
28	C	1.896901	1.894348	2.937235	3.256556	4.003592

28	C	0.062963	-0.071489
29	O	-0.183131	-0.018228
30	C	0.123103	-0.065007
31	O	-0.182790	-0.016671

29	O	2.915225	2.914434	3.925156	3.782895	4.479764
30	C	1.903027	1.903367	2.904091	4.477144	5.113520
31	O	2.921808	2.922105	3.888453	5.571601	6.165713
		6	7	8	9	10
6	H	0.000000				
7	H	1.778403	0.000000			
8	H	6.361370	6.075490	0.000000		
9	C	5.375386	5.186476	1.079395	0.000000	
10	C	4.916869	4.483246	2.228703	1.414440	0.000000
11	H	5.122951	5.576022	2.683295	2.231167	3.336202
12	H	5.612103	4.897630	2.688713	2.229173	1.079438
13	C	3.701486	3.572422	3.336979	2.293292	1.418433
14	H	3.430276	3.196369	4.345651	3.335572	2.229865
15	C	3.464265	3.887357	3.336516	2.294384	2.294656
16	H	2.948773	3.858091	4.344676	3.336520	3.338367
17	C	4.622214	4.878693	2.227423	1.418054	2.293441
18	H	7.084356	6.082116	5.519801	5.693750	6.015268
19	C	6.234621	5.194186	5.693845	5.662204	5.832463
20	C	5.616859	4.885092	6.077363	5.899380	6.164354
21	H	6.421353	4.908670	6.131867	6.011074	5.682607
22	H	6.033438	5.580344	6.257520	6.143969	6.621644
23	C	4.729018	3.895982	6.604709	6.211950	6.207385
24	H	4.395955	3.865336	7.227482	6.733978	6.730032
25	C	4.888652	3.584269	6.559452	6.162428	5.888643
26	H	4.686576	3.210534	7.122337	6.617863	6.130935
27	C	5.844546	4.493740	6.013839	5.831772	5.653818
28	C	4.007630	2.721919	4.136551	3.572621	2.958895
29	O	4.484160	2.954383	4.827157	4.276661	3.347077
30	C	5.113541	4.611283	3.036392	2.936295	3.534329
31	O	6.165138	5.764267	3.053269	3.309791	4.214995
		11	12	13	14	15
11	H	0.000000				
12	H	4.346758	0.000000			
13	C	3.339285	2.227699	0.000000		
14	H	4.350091	2.680789	1.079276	0.000000	
15	C	2.231817	3.336742	1.419585	2.233648	0.000000
16	H	2.685821	4.347159	2.233692	2.694490	1.079005
17	C	1.079074	3.337264	2.296396	3.340279	1.419735
18	H	6.254874	6.134495	6.560254	7.123832	6.603719
19	C	6.142937	6.012236	6.162879	6.618676	6.211500
20	C	5.888464	6.558701	6.223240	6.675659	6.007156
21	H	7.123507	5.505089	6.065300	6.243288	6.598622
22	H	5.786405	7.124147	6.676516	7.231684	6.246492
23	C	6.248806	6.596936	6.001166	6.239955	5.884861
24	H	6.495851	7.220792	6.315711	6.488642	6.059503
25	C	6.678249	6.063035	5.778512	5.874607	6.002316

26	H	7.234181	6.239623	5.874700	5.770815	6.241961
27	C	6.621538	5.682243	5.889775	6.132773	6.208333
28	C	4.752113	3.081353	3.020186	3.206300	3.665041
29	O	5.718326	3.135895	3.424901	3.305689	4.385504
30	C	3.264640	4.071754	3.952998	4.752313	3.706137
31	O	3.418998	4.719587	4.818873	5.714996	4.450972
		16	17	18	19	20
16	H	0.000000				
17	C	2.230057	0.000000			
18	H	7.226127	6.075841	0.000000		
19	C	6.733505	5.898777	1.079395	0.000000	
20	C	6.320663	5.790707	2.227487	1.418119	0.000000
21	H	7.223527	6.558571	2.688622	2.229108	3.337277
22	H	6.492570	5.888084	2.683348	2.231216	1.079073
23	C	6.059963	6.008349	3.336536	2.294409	1.419696
24	H	6.041964	6.322168	4.344697	3.336549	2.230013
25	C	6.318027	6.224246	3.336988	2.293293	2.296347
26	H	6.492460	6.677150	4.345638	3.335555	3.340231
27	C	6.731778	6.164389	2.228612	1.414362	2.293448
28	C	4.319673	3.952973	4.135644	3.570718	3.950065
29	O	5.036127	4.819933	4.829246	4.277786	4.819466
30	C	4.380847	3.056134	3.034637	2.935136	3.056077
31	O	5.136296	3.486912	3.050478	3.308140	3.487229
		21	22	23	24	25
21	H	0.000000				
22	H	4.346752	0.000000			
23	C	3.336798	2.231790	0.000000		
24	H	4.347224	2.685783	1.079001	0.000000	
25	C	2.227822	3.339235	1.419543	2.233649	0.000000
26	H	2.680902	4.350043	2.233624	2.694467	1.079272
27	C	1.079432	3.336192	2.294720	3.338440	1.418558
28	C	3.080063	4.749460	3.661119	4.315259	3.016152
29	O	3.138327	5.717759	4.383729	5.033231	3.423485
30	C	4.070162	3.264902	3.706531	4.381548	3.952602
31	O	4.716903	3.420149	4.451695	5.137676	4.818297
		26	27	28	29	30
26	H	0.000000				
27	C	2.229960	0.000000			
28	C	3.201983	2.956469	0.000000		
29	O	3.303228	3.348058	1.164968	0.000000	
30	C	4.751977	3.533095	2.787354	3.884707	0.000000
31	O	5.714433	4.213089	3.877593	4.918040	1.163205
		31				
31	O	0.000000				

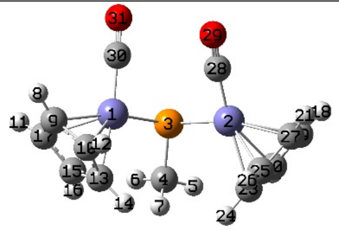

9Q -3521.195601 7.88 C1
WBI 0.06

Charge and spin density

		1	2
1	Fe	0.314704	2.214436
2	Fe	0.306398	2.239740
3	P	-0.229606	-0.098219
4	C	-0.080078	-0.001477
9	C	0.004537	-0.032928
10	C	-0.029669	0.057872
13	C	0.010207	-0.030955
15	C	-0.015960	-0.005148
17	C	-0.024994	-0.004543
19	C	-0.035145	0.007680
20	C	-0.030251	-0.000081
23	C	0.047111	-0.040955
25	C	-0.047185	0.047564
27	C	0.010123	-0.030400
28	C	-0.008630	-0.122835
29	O	-0.104804	-0.047778
30	C	0.009126	-0.113824
31	O	-0.095886	-0.038148

		1	2	3	4	5
1	Fe	0.000000				
2	Fe	3.551545	0.000000			
3	P	2.263903	2.249285	0.000000		
4	C	3.366561	3.366466	1.863634	0.000000	
5	H	4.335779	3.494866	2.456047	1.092682	0.000000
6	H	3.589832	4.329269	2.444266	1.093122	1.761294
7	H	3.482364	3.595894	2.517269	1.092091	1.772964
8	H	2.957981	6.186290	5.216181	6.136074	7.180609
9	C	2.215292	5.526258	4.448681	5.176455	6.237889
10	C	2.234963	4.824280	4.186213	4.783583	5.788248
11	H	2.933504	6.406107	4.616733	4.982295	6.036205
12	H	2.965558	4.923314	4.763842	5.483877	6.411409
13	C	2.154289	4.416153	3.526142	3.639391	4.649104
14	H	2.884205	4.183040	3.653720	3.458315	4.332835
15	C	2.139762	5.001989	3.460039	3.391791	4.478838
16	H	2.865001	5.280907	3.534678	2.948040	3.982207
17	C	2.199999	5.659291	4.084981	4.474307	5.560822
18	H	6.430450	2.941863	4.693796	5.375313	4.891784
19	C	5.747679	2.213014	4.122278	4.695444	4.307710
20	C	5.258543	2.143566	3.456169	3.537739	3.049053
21	H	6.103753	2.969220	5.216426	6.130551	6.049116
22	H	5.609323	2.869220	3.559597	3.300043	2.525986
23	C	4.725398	2.142525	3.425210	3.368615	3.223300
24	H	4.643644	2.874964	3.512576	2.955152	2.907489
25	C	4.969514	2.231741	4.099500	4.504541	4.516707
26	H	5.051832	2.964910	4.650195	5.051311	5.231134
27	C	5.549286	2.225978	4.436798	5.158456	5.023407
28	C	3.524932	1.789634	3.019554	4.670257	4.984892
29	O	4.030118	2.929352	3.930074	5.684259	6.050662
30	C	1.793014	3.942347	2.909234	4.528480	5.318152
31	O	2.931582	4.592998	3.741133	5.447685	6.140061

		6	7	8	9	10
6	H	0.000000				
7	H	1.770552	0.000000			
8	H	6.143774	6.025166	0.000000		
9	C	5.198086	4.995010	1.080040	0.000000	
10	C	5.057175	4.380182	2.223653	1.412974	0.000000
11	H	4.536222	5.045760	2.682499	2.230772	3.340986
12	H	5.904308	5.027523	2.674898	2.224149	1.080010
13	C	3.904048	3.094224	3.323370	2.280640	1.409403
14	H	3.922398	2.667682	4.335036	3.325699	2.225094
15	C	3.244882	3.101644	3.326946	2.284063	2.297216
16	H	2.554986	2.683533	4.340292	3.329901	3.336853
17	C	4.235958	4.384353	2.230396	1.420235	2.300126
18	H	6.329249	5.730974	9.069908	8.457862	7.739955

19	C	5.720898	4.898035	8.371389	7.701603	6.881411
20	C	4.577493	3.686487	8.033529	7.231690	6.407909
21	H	7.174570	6.117856	8.165711	7.672392	6.721502
22	H	4.222703	3.624619	8.499515	7.656613	6.947189
23	C	4.458306	3.108675	7.284015	6.435809	5.429118
24	H	3.982202	2.405124	7.128706	6.189507	5.156326
25	C	5.581224	4.217020	7.206245	6.488356	5.379876
26	H	6.082881	4.588715	6.918960	6.238099	5.004450
27	C	6.222818	5.136613	7.856411	7.246612	6.283469
28	C	5.442251	4.986127	5.645515	5.277619	4.809537
29	O	6.369021	6.021814	5.656858	5.504592	5.195247
30	C	4.702453	4.943685	3.299261	3.116361	3.550001
31	O	5.583927	5.970076	3.933066	4.000640	4.562889
		11	12	13	14	15
11	H	0.000000				
12	H	4.348059	0.000000			
13	C	3.343984	2.225668	0.000000		
14	H	4.352617	2.687024	1.079620	0.000000	
15	C	2.228639	3.343177	1.430367	2.239661	0.000000
16	H	2.693986	4.349435	2.237475	2.689159	1.078810
17	C	1.079340	3.342295	2.297399	3.338719	1.410289
18	H	9.211904	7.733961	7.276644	6.874338	7.821293
19	C	8.549259	6.860888	6.369750	5.893623	7.008378
20	C	7.861424	6.554338	5.643408	5.087372	6.166758
21	H	9.027454	6.358241	6.602544	6.161457	7.556050
22	H	7.992005	7.232152	6.048966	5.528121	6.354133
23	C	7.299475	5.497658	4.645960	3.940223	5.399481
24	H	6.928465	5.335237	4.168430	3.338431	4.877126
25	C	7.726899	5.176715	4.925106	4.268908	5.921936
26	H	7.710540	4.630677	4.695105	3.999410	5.876851
27	C	8.438151	6.061210	5.963082	5.456042	6.847178
28	C	6.373942	4.756989	4.901779	4.984418	5.472313
29	O	6.696156	5.040693	5.555497	5.774302	6.103898
30	C	3.645344	4.019550	3.891459	4.619827	3.768841
31	O	4.388390	4.932910	5.016982	5.749524	4.848456
		16	17	18	19	20
16	H	0.000000				
17	C	2.227137	0.000000			
18	H	7.921154	8.540765	0.000000		
19	C	7.128763	7.806341	1.079696	0.000000	
20	C	6.125093	7.132770	2.226343	1.408751	0.000000
21	H	7.960383	8.169409	2.678023	2.228075	3.326618
22	H	6.139328	7.363485	2.690009	2.225290	1.079185
23	C	5.455654	6.446815	3.341979	2.295238	1.431522
24	H	4.816522	6.069493	4.348750	3.334914	2.238051
25	C	6.209852	6.799854	3.339892	2.299075	2.300457

26	H	6.284091	6.713029	4.346350	3.340916	3.346625
27	C	7.147025	7.586830	2.228616	1.418721	2.284510
28	C	5.999626	5.701820	3.747953	3.369269	3.807968
29	O	6.737321	6.092604	4.515338	4.295682	4.897367
30	C	4.426495	3.315989	6.496384	6.058933	5.900480
31	O	5.473076	4.245514	6.831831	6.552859	6.567600

		21	22	23	24	25
21	H	0.000000				
22	H	4.338960	0.000000			
23	C	3.324098	2.240891	0.000000		
24	H	4.336422	2.690146	1.078843	0.000000	
25	C	2.224176	3.341318	1.411937	2.228136	0.000000
26	H	2.675784	4.354662	2.229062	2.693336	1.079683
27	C	1.080127	3.330029	2.280634	3.325592	1.412649
28	C	3.227729	4.508134	3.830430	4.552289	3.430299
29	O	3.775024	5.592617	4.932495	5.659123	4.378945
30	C	6.280895	6.250745	5.694659	5.892357	5.755861
31	O	6.701234	6.898814	6.527332	6.837840	6.509566

		26	27	28	29	30
26	H	0.000000				
27	C	2.223763	0.000000			
28	C	3.848226	3.063544	0.000000		
29	O	4.661676	3.892056	1.142012	0.000000	
30	C	5.947060	5.930152	3.158818	3.217329	0.000000
31	O	6.748042	6.473727	3.483011	3.215194	1.139620

		31
31	O	0.000000

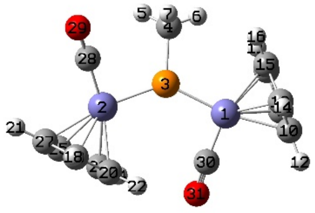

10T -3521.194435 8.61 C1
WBI 0.05

Charge and spin density

		1	2
1	Fe	0.202375	0.570453
2	Fe	0.287374	1.987346
3	P	-0.019218	-0.358282
4	C	-0.020788	-0.007253
9	C	-0.019441	-0.023966
10	C	-0.002965	-0.029650
13	C	-0.048562	0.074942

		1	2	3	4	5
1	Fe	0.000000				
2	Fe	3.975595	0.000000			
3	P	2.148529	2.189975	0.000000		
4	C	3.351780	3.374257	1.852989	0.000000	
5	H	4.230212	3.413742	2.465434	1.091069	0.000000
6	H	3.295290	4.307221	2.454134	1.089570	1.769906
7	H	3.842955	3.710979	2.459306	1.093407	1.767307
8	H	2.870576	6.386002	4.572450	5.321336	6.008051
9	C	2.119353	5.929440	3.974589	4.651742	5.469571
10	C	2.116377	6.050157	4.240450	5.118829	6.071032
11	H	2.865326	5.687313	3.566061	3.339643	3.987779
12	H	2.865962	6.595256	5.002034	6.076110	7.012994
13	C	2.124124	5.762260	3.885607	4.474837	5.527172
14	H	2.859254	6.060420	4.400225	5.005112	6.085704
15	C	2.110831	5.420819	3.312715	3.394057	4.424716
16	H	2.868203	5.476838	3.442256	3.029411	4.068005
17	C	2.118984	5.546445	3.393317	3.553676	4.395476
18	H	5.718015	2.908843	4.588000	5.823178	6.036708

15	C	0.057741	-0.034784
17	C	-0.065922	-0.015460
19	C	-0.032845	-0.003472
20	C	-0.022895	-0.006194
23	C	0.029052	-0.027209
25	C	-0.038016	0.051493
27	C	0.010941	-0.037104
28	C	-0.024153	-0.116866
29	O	-0.096100	-0.040954
30	C	-0.061259	0.005697
31	O	-0.135320	0.011263

19	C	5.252399	2.173260	4.035270	5.395121	5.529554
20	C	4.262465	2.150011	3.460865	5.132132	5.401996
21	H	6.816396	2.915007	5.096139	5.963354	5.683665
22	H	3.846600	2.885366	3.595330	5.364850	5.826014
23	C	4.443702	2.156450	3.468780	5.065546	5.126569
24	H	4.223126	2.900365	3.619229	5.254282	5.347376
25	C	5.506301	2.205693	4.069014	5.318607	5.116468
26	H	6.130074	2.934304	4.617029	5.657410	5.271174
27	C	5.905164	2.175236	4.344516	5.473278	5.324885
28	C	4.895680	1.766714	2.842620	3.011481	2.822392
29	O	5.711760	2.909967	3.685985	3.316493	3.006514
30	C	1.748684	3.889297	2.884339	4.553587	5.204343
31	O	2.894337	4.321531	3.793552	5.533919	6.070674
		6	7	8	9	10
6	H	0.000000				
7	H	1.769852	0.000000			
8	H	4.758161	6.046027	0.000000		
9	C	4.100890	5.246788	1.079167	0.000000	
10	C	4.740907	5.483981	2.239469	1.428989	0.000000
11	H	2.445650	4.040635	2.677701	2.219921	3.337738
12	H	5.771533	6.430375	2.695310	2.240015	1.079422
13	C	4.147621	4.588063	3.338269	2.296731	1.410041
14	H	4.818789	4.907147	4.348422	3.340754	2.222008
15	C	2.867870	3.574109	3.338470	2.293905	2.290434
16	H	2.521446	2.938048	4.348256	3.336456	3.332061
17	C	2.840261	4.110861	2.224902	1.409553	2.294272
18	H	6.780349	5.744421	8.344403	7.801313	7.428305
19	C	6.327018	5.513545	7.724297	7.281781	7.070046
20	C	5.903365	5.443767	6.523507	6.172593	5.982017
21	H	6.987684	6.103304	9.155191	8.779470	8.823744
22	H	6.021320	5.631334	6.086815	5.698117	5.305849
23	C	5.773303	5.620443	6.292822	6.130920	6.255993
24	H	5.789683	5.957499	5.620046	5.621472	5.876933
25	C	6.156201	5.817050	7.424908	7.241628	7.456809
26	H	6.444252	6.255488	7.790862	7.706311	8.090710
27	C	6.443183	5.711928	8.195589	7.848736	7.873549
28	C	4.097677	2.961850	7.403761	6.807321	6.948574
29	O	4.372626	2.987806	8.208806	7.551448	7.695072
30	C	4.687323	5.156817	3.194759	2.959648	2.912434
31	O	5.720338	6.174811	3.777138	3.803029	3.754165
		11	12	13	14	15
11	H	0.000000				
12	H	4.346108	0.000000			
13	C	3.340367	2.223897	0.000000		
14	H	4.351245	2.677954	1.079949	0.000000	
15	C	2.237067	3.334079	1.417916	2.230073	0.000000

16	H	2.697896	4.341934	2.229204	2.684752	1.078849
17	C	1.079337	3.336448	2.296610	3.340535	1.423645
18	H	8.078108	7.674608	7.043995	6.922948	7.172392
19	C	7.520718	7.342949	6.833683	6.875853	6.879731
20	C	6.672490	6.178608	5.965138	6.119061	6.120862
21	H	8.556064	9.234162	8.543224	8.691350	8.293087
22	H	6.513708	5.375639	5.339408	5.409545	5.723225
23	C	6.438265	6.519941	6.421545	6.792718	6.380726
24	H	6.064737	6.092174	6.263328	6.760197	6.242842
25	C	7.206493	7.815382	7.487118	7.844131	7.270487
26	H	7.487191	8.493265	8.181390	8.632020	7.840650
27	C	7.794032	8.239395	7.685664	7.867609	7.516300
28	C	6.094285	7.646489	6.349678	6.560406	5.758622
29	O	6.615091	8.455920	6.947443	7.100053	6.245411
30	C	4.160876	3.111103	3.484520	4.057478	3.815762
31	O	5.127904	3.683016	4.506311	5.015959	4.937582
		16	17	18	19	20
16	H	0.000000				
17	C	2.237364	0.000000			
18	H	7.212532	7.655165	0.000000		
19	C	7.007194	7.172131	1.079304	0.000000	
20	C	6.446136	6.259461	2.228303	1.409494	0.000000
21	H	8.266084	8.459334	2.685467	2.234303	3.330228
22	H	6.146957	5.944411	2.693845	2.226290	1.078439
23	C	6.761251	6.214366	3.342488	2.295966	1.428251
24	H	6.762383	5.864447	4.349005	3.335390	2.235337
25	C	7.492255	7.129144	3.340146	2.299584	2.297014
26	H	8.053823	7.551149	4.348399	3.342646	3.342233
27	C	7.597262	7.634415	2.233692	1.424405	2.287730
28	C	5.475520	6.089154	3.543178	3.227327	3.732145
29	O	5.776301	6.680475	4.286193	4.161891	4.817476
30	C	4.615115	3.539183	5.324234	4.701817	3.416145
31	O	5.760865	4.563704	5.463998	4.768998	3.372346
		21	22	23	24	25
21	H	0.000000				
22	H	4.343427	0.000000			
23	C	3.326659	2.234505	0.000000		
24	H	4.337487	2.683072	1.078224	0.000000	
25	C	2.224269	3.336374	1.411644	2.227182	0.000000
26	H	2.677130	4.347782	2.226276	2.688149	1.080083
27	C	1.079916	3.333422	2.283269	3.327031	1.411778
28	C	3.195275	4.400572	3.868930	4.623140	3.499461
29	O	3.818083	5.453823	4.997224	5.755565	4.511758
30	C	6.397598	2.906502	3.455490	2.999587	4.758761
31	O	6.464382	2.852395	3.269503	2.621889	4.657371
		26	27	28	29	30

26	H	0.000000				
27	C	2.223437	0.000000			
28	C	3.979542	3.025950	0.000000		
29	O	4.888104	3.910233	1.143459	0.000000	
30	C	5.402812	5.363715	5.314581	6.331755	0.000000
31	O	5.268498	5.391349	5.935172	7.020381	1.148158
		31				
31	O	0.000000				

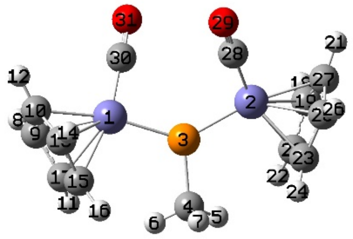

11T -3521.193433 9.24 C2
WBI 0.10

Charge and spin density

		1	2
1	Fe	0.250059	2.006909
2	Fe	0.238910	0.539470
3	P	-0.024092	-0.365489
4	C	-0.058772	-0.010438
9	C	-0.019888	-0.022236
10	C	-0.000880	-0.039629
13	C	-0.028972	0.062926
15	C	0.071663	-0.039445
17	C	-0.076052	0.016006
19	C	-0.039822	0.079559
20	C	0.009550	-0.044445
23	C	-0.016534	0.006927
25	C	-0.027595	-0.047291
27	C	0.006631	-0.017923
28	C	-0.051263	0.004102
29	O	-0.131486	0.014124
30	C	-0.007780	-0.106137
31	O	-0.093676	-0.036990

		1	2	3	4	5
1	Fe	0.000000				
2	Fe	3.989211	0.000000			
3	P	2.178429	2.148492	0.000000		
4	C	3.338351	3.358128	1.852249	0.000000	
5	H	4.238998	3.342288	2.459671	1.090848	0.000000
6	H	3.292416	4.264493	2.467530	1.090247	1.773568
7	H	3.786114	3.776956	2.446242	1.093818	1.768413
8	H	2.900434	6.464604	4.685199	5.339031	6.099678
9	C	2.160844	6.000950	4.078880	4.673140	5.552178
10	C	2.165204	6.112849	4.314123	5.140944	6.129146
11	H	2.896779	5.766445	3.696635	3.377264	4.114372
12	H	2.905378	6.657287	5.065031	6.094163	7.062666
13	C	2.203838	5.847108	3.971202	4.528865	5.598905
14	H	2.928714	6.141327	4.462056	5.061310	6.141554
15	C	2.159508	5.484771	3.399337	3.448129	4.507700
16	H	2.903206	5.530928	3.509749	3.094582	4.146676
17	C	2.161411	5.619574	3.510337	3.591918	4.498116
18	H	6.242937	2.863133	4.518293	5.298517	4.759978
19	C	5.871468	2.128669	3.958393	4.656311	4.137218
20	C	5.479465	2.113211	3.363845	3.545026	2.868752
21	H	6.637628	2.868255	5.008145	6.091183	5.827234
22	H	5.569835	2.868140	3.528887	3.321594	2.440894
23	C	5.488170	2.119722	3.345297	3.434114	2.963155
24	H	5.568405	2.866834	3.477803	3.075013	2.630792
25	C	5.866032	2.125249	3.915210	4.504287	4.230262
26	H	6.260598	2.875861	4.470346	5.063235	4.926671
27	C	6.081976	2.119891	4.246372	5.134770	4.798672
28	C	3.825380	1.756646	2.829618	4.466911	4.779239
29	O	4.189387	2.900159	3.704276	5.389674	5.817351
30	C	1.776131	3.789741	2.845796	4.508454	5.171945
31	O	2.916705	4.119314	3.702183	5.445766	5.985861
		6	7	8	9	10
6	H	0.000000				
7	H	1.769670	0.000000			
8	H	4.742892	5.981823	0.000000		
9	C	4.116015	5.177767	1.079244	0.000000	
10	C	4.789559	5.421746	2.238694	1.429619	0.000000

11	H	2.458902	3.960751	2.687242	2.222843	3.337315
12	H	5.812211	6.375206	2.691072	2.238986	1.079643
13	C	4.259357	4.546848	3.339294	2.298901	1.408759
14	H	4.948484	4.879656	4.349656	3.343291	2.222234
15	C	2.992716	3.509535	3.338064	2.291796	2.282669
16	H	2.694702	2.871778	4.347362	3.333619	3.325338
17	C	2.888122	4.037036	2.226230	1.407968	2.291527
18	H	6.039488	5.966157	8.046821	7.901714	8.330502
19	C	5.521610	5.183090	7.974286	7.686263	8.013924
20	C	4.436049	4.032359	7.616756	7.230935	7.639435
21	H	7.053320	6.376223	9.014495	8.655825	8.644185
22	H	4.034723	3.965254	7.405169	7.084407	7.678439
23	C	4.486725	3.527767	7.971763	7.410336	7.600253
24	H	4.122475	2.896893	8.064867	7.409507	7.586588
25	C	5.571125	4.534979	8.497851	7.938602	7.934653
26	H	6.145200	4.885534	9.030315	8.387742	8.214444
27	C	6.114760	5.428066	8.489141	8.089894	8.173293
28	C	5.270193	4.719355	6.412048	5.916846	5.661492
29	O	6.132574	5.548812	6.700785	6.198516	5.705168
30	C	4.658844	5.086724	3.406746	3.146512	3.062918
31	O	5.657467	6.067143	4.081886	4.045647	3.950755
		11	12	13	14	15
11	H	0.000000				
12	H	4.346026	0.000000			
13	C	3.339651	2.222761	0.000000		
14	H	4.349464	2.679046	1.080032	0.000000	
15	C	2.236167	3.326670	1.413356	2.225443	0.000000
16	H	2.691267	4.335804	2.225563	2.680353	1.079177
17	C	1.079489	3.333335	2.297777	3.341492	1.425427
18	H	7.473192	8.821548	8.342551	8.800075	7.860143
19	C	7.187618	8.558144	7.855519	8.230160	7.351496
20	C	6.388890	8.332115	7.319488	7.726581	6.611565
21	H	8.598410	8.991404	8.453206	8.590029	8.273448
22	H	5.982130	8.445196	7.402678	7.927470	6.537215
23	C	6.623472	8.300083	7.050128	7.277300	6.404298
24	H	6.429995	8.367852	6.871636	7.058970	6.115553
25	C	7.504701	8.489236	7.428222	7.521633	7.030195
26	H	8.065541	8.723666	7.594646	7.533992	7.309995
27	C	7.812074	8.636313	7.904730	8.099628	7.575214
28	C	6.297637	5.960308	5.473107	5.565873	5.549066
29	O	6.912936	5.815654	5.563309	5.497452	5.914446
30	C	4.302887	3.258117	3.597732	4.133269	3.903165
31	O	5.303122	3.900741	4.633048	5.096394	5.032197
		16	17	18	19	20
16	H	0.000000				
17	C	2.236463	0.000000			

18	H	7.969848	7.605255	0.000000		
19	C	7.352439	7.272553	1.079963	0.000000	
20	C	6.459904	6.582466	2.230158	1.417360	0.000000
21	H	8.312188	8.434794	2.677284	2.223699	3.333005
22	H	6.374330	6.345075	2.684306	2.228372	1.079216
23	C	6.098629	6.681896	3.340772	2.296575	1.423988
24	H	5.642137	6.520957	4.350042	3.339187	2.235000
25	C	6.817963	7.398828	3.341375	2.297661	2.295048
26	H	7.039332	7.858534	4.348742	3.339067	3.339727
27	C	7.539451	7.727965	2.222465	1.410582	2.289572
28	C	5.764491	5.855249	4.039313	3.484684	3.828927
29	O	6.207488	6.322028	4.998933	4.512388	4.953970
30	C	4.668326	3.680273	5.646273	5.483471	5.531339
31	O	5.808995	4.733181	5.555653	5.554817	5.878276
		21	22	23	24	25
21	H	0.000000				
22	H	4.340952	0.000000			
23	C	3.335200	2.238574	0.000000		
24	H	4.346118	2.695692	1.079179	0.000000	
25	C	2.238576	3.338193	1.410374	2.222731	0.000000
26	H	2.693707	4.350297	2.226174	2.682730	1.079102
27	C	1.079470	3.331477	2.292835	3.337084	1.427696
28	C	3.120182	4.619382	3.577740	4.210532	3.010455
29	O	3.713526	5.763866	4.612041	5.188966	3.872375
30	C	5.947515	5.775673	5.738744	6.131699	5.800710
31	O	5.796545	6.192995	6.201379	6.749966	6.080878
		26	27	28	29	30
26	H	0.000000				
27	C	2.238321	0.000000			
28	C	3.260951	2.930615	0.000000		
29	O	3.873923	3.788517	1.145385	0.000000	
30	C	6.243002	5.634008	3.203141	3.377094	0.000000
31	O	6.538925	5.668039	3.332727	3.340143	1.141438
		31				
31	O	0.000000				

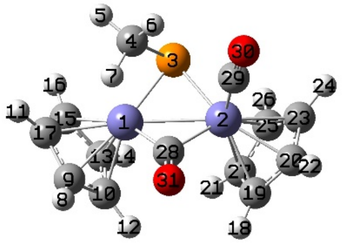

12S -3521.192739 9.68 C1
WBI 0.31

		1	2	3	4	5
1	Fe	0.000000				
2	Fe	2.568750	0.000000			
3	P	2.179835	2.230165	0.000000		
4	C	2.223394	3.162193	1.847652	0.000000	
5	H	2.872263	4.176123	2.473956	1.089128	0.000000
6	H	3.037698	3.338291	2.510725	1.090224	1.788429
7	H	1.731474	3.261581	2.546256	1.167609	1.801554
8	H	2.841312	4.767427	5.016107	4.521923	4.958506
9	C	2.089113	4.299705	4.237724	3.963791	4.340855
10	C	2.063425	3.762695	3.899811	4.267262	4.763121

Charge and spin density

		1
1	Fe	0.028444
2	Fe	-0.039755
3	P	-0.170873
4	C	0.101904
9	C	0.014034
10	C	-0.014661
13	C	0.010176
15	C	0.053517
17	C	0.003566
19	C	0.011836
20	C	0.005726
23	C	0.010448
25	C	0.046453
27	C	0.019295
28	C	0.272322
29	C	-0.011725
30	O	-0.129165
31	O	-0.211543

11	H	2.831606	5.348573	4.417007	3.484626	3.298209
12	H	2.813179	3.825947	4.479692	5.025677	5.651638
13	C	2.065989	3.842855	3.271246	3.940262	4.239513
14	H	2.819094	3.980351	3.395148	4.491151	4.794172
15	C	2.078871	4.402562	3.248132	3.356146	3.332234
16	H	2.837960	4.957401	3.356083	3.482988	3.165441
17	C	2.086004	4.648447	3.873341	3.373308	3.415297
18	H	4.070193	2.857077	4.810392	5.620606	6.600163
19	C	3.849498	2.110531	4.102113	5.143563	6.129890
20	C	4.470265	2.087218	4.210700	5.185640	6.226470
21	H	3.497350	2.883857	3.693813	5.121038	5.846149
22	H	5.145967	2.834204	4.993455	5.705923	6.781433
23	C	4.552773	2.068532	3.615991	4.904589	5.850546
24	H	5.291736	2.817722	4.029638	5.229483	6.130610
25	C	4.028275	2.111459	3.089190	4.711488	5.533556
26	H	4.388306	2.858902	3.059075	4.851722	5.514323
27	C	3.529405	2.121211	3.426559	4.849385	5.700335
28	C	1.861326	1.973567	3.124816	3.088524	4.056780
29	C	3.420620	1.742097	2.818940	2.941710	3.987240
30	O	4.333608	2.886204	3.669516	3.398016	4.337971
31	O	2.880816	2.898871	4.278665	4.031786	4.973417

		6	7	8	9	10
6	H	0.000000				
7	H	1.719527	0.000000			
8	H	5.102362	3.454616	0.000000		
9	C	4.720332	3.047912	1.078952	0.000000	
10	C	5.085691	3.625449	2.237472	1.424778	0.000000
11	H	4.284337	2.711247	2.680672	2.221041	3.329877
12	H	5.736741	4.390491	2.688269	2.232332	1.079431
13	C	4.920079	3.637218	3.345493	2.303619	1.416271
14	H	5.457326	4.409988	4.356681	3.347784	2.232703
15	C	4.414048	3.058061	3.344199	2.300797	2.293299
16	H	4.568467	3.482316	4.353148	3.343070	3.336294
17	C	4.277248	2.629916	2.227654	1.410856	2.285574
18	H	5.868559	5.240114	4.745017	4.613897	3.779917
19	C	5.374458	4.982697	5.168543	4.857884	3.957413
20	C	5.156362	5.212726	6.077061	5.806109	5.085069
21	H	5.746038	5.073848	5.083053	4.397212	3.097188
22	H	5.498285	5.662716	6.485658	6.373312	5.808032
23	C	4.911962	5.241310	6.699658	6.229390	5.441817
24	H	5.072678	5.725824	7.565316	7.097742	6.397044
25	C	5.025246	5.060885	6.304019	5.657720	4.673316
26	H	5.250189	5.381636	6.871760	6.103609	5.101614
27	C	5.293687	4.880286	5.328346	4.742640	3.624028
28	C	3.330289	2.458759	3.003561	2.847008	2.820307
29	C	2.514581	3.178904	5.453671	5.175281	5.051919

30	O	2.620256	3.686880	6.187885	6.012953	6.060731
31	O	4.104218	3.238819	2.957560	3.207619	3.381001
		11	12	13	14	15
11	H	0.000000				
12	H	4.337401	0.000000			
13	C	3.336180	2.229829	0.000000		
14	H	4.344007	2.693052	1.078781	0.000000	
15	C	2.229508	3.337413	1.420858	2.229476	0.000000
16	H	2.688758	4.347365	2.233034	2.684583	1.079083
17	C	1.080445	3.327317	2.293075	3.335102	1.420093
18	H	6.519224	3.053693	4.543828	4.633149	5.616317
19	C	6.542192	3.432160	4.405913	4.355217	5.441294
20	C	7.227607	4.710780	5.412664	5.366467	6.259993
21	H	5.977946	2.735022	2.988215	2.502312	4.266376
22	H	7.784904	5.399554	6.284028	6.338282	7.060225
23	C	7.365293	5.257932	5.395758	5.173808	6.154533
24	H	8.037130	6.286941	6.264271	6.034036	6.892963
25	C	6.809340	4.542772	4.388918	3.974601	5.265788
26	H	7.021136	5.112110	4.517019	3.937308	5.294003
27	C	6.253397	3.283289	3.628893	3.301294	4.740766
28	C	4.125805	2.962924	3.494028	4.110348	3.865087
29	C	5.771799	5.241552	5.180911	5.480263	5.367292
30	O	6.373010	6.285913	6.209221	6.559729	6.243261
31	O	4.638643	3.306255	4.357573	5.032680	4.742128
		16	17	18	19	20
16	H	0.000000				
17	C	2.235438	0.000000			
18	H	6.484945	5.640785	0.000000		
19	C	6.183804	5.663748	1.079132	0.000000	
20	C	6.891900	6.464987	2.217607	1.407760	0.000000
21	H	4.878073	4.952628	2.691399	2.232770	3.332766
22	H	7.729946	7.100688	2.672933	2.221920	1.079181
23	C	6.584019	6.614853	3.334454	2.291264	1.429239
24	H	7.215664	7.367849	4.343249	3.333664	2.240634
25	C	5.640673	5.958323	3.339260	2.296740	2.302496
26	H	5.471423	6.192145	4.349701	3.340508	3.346088
27	C	5.338231	5.303666	2.236318	1.423002	2.292479
28	C	4.696152	3.514054	2.879806	2.857120	3.314471
29	C	5.810368	5.358690	4.095075	3.474903	2.838762
30	O	6.621434	6.119566	5.055186	4.491599	3.668553
31	O	5.650774	4.129185	2.906397	3.230223	3.661718
		21	22	23	24	25
21	H	0.000000				
22	H	4.343065	0.000000			
23	C	3.328818	2.242117	0.000000		
24	H	4.338509	2.700097	1.079286	0.000000	

		1	2	3	4	5
25	C	2.224084	3.345253	1.415969	2.228145	0.000000
26	H	2.681973	4.355630	2.228147	2.683689	1.079293
27	C	1.078125	3.336516	2.286566	3.329670	1.412731
28	C	3.633789	3.708725	3.900876	4.673174	3.892352
29	C	4.624427	3.038724	2.801574	2.985797	3.455221
30	O	5.765274	3.593531	3.627589	3.526963	4.465236
31	O	4.386740	3.769255	4.567960	5.328000	4.755277

		26	27	28	29	30
26	H	0.000000				
27	C	2.226718	0.000000			
28	C	4.640762	3.271565	0.000000		
29	C	4.062254	3.801926	2.579304	0.000000	
30	O	5.008949	4.915433	3.462259	1.146059	0.000000
31	O	5.614161	4.010794	1.170202	3.208337	3.877890

		31
31	O	0.000000

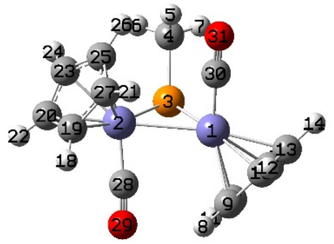

13T -3521.192070 10.10 C1
WBI 0.33

Charge and spin density

		1	2
1	Fe	0.259466	1.889173
2	Fe	0.215517	0.515503
3	P	-0.150416	-0.114024
4	C	-0.043496	0.000313
9	C	-0.064075	0.030370
10	C	0.013567	-0.034609
13	C	-0.049229	-0.030825
15	C	0.049234	0.030056
17	C	0.058767	-0.033383
19	C	0.024755	0.015543
20	C	-0.043329	-0.005284
23	C	0.014019	-0.008968
25	C	0.020794	0.011832
27	C	-0.045506	-0.079140
28	C	0.011758	-0.031186
29	O	-0.125434	-0.010453
30	C	-0.030430	-0.106356

		1	2	3	4	5
1	Fe	0.000000				
2	Fe	2.650633	0.000000			
3	P	2.286590	2.195833	0.000000		
4	C	3.525150	3.353641	1.859562	0.000000	
5	H	3.615851	3.484177	2.534710	1.089965	0.000000
6	H	4.416553	3.646532	2.431287	1.093673	1.773282
7	H	3.919061	4.291266	2.437260	1.093232	1.769102
8	H	2.903988	3.945886	4.594633	6.222296	6.372785
9	C	2.172174	3.888697	4.035732	5.594994	5.775040
10	C	2.138546	4.440765	4.368726	5.576453	5.563533
11	H	2.930275	3.990693	3.487376	5.266074	5.848624
12	H	2.875092	4.958500	5.161664	6.211573	6.026907
13	C	2.149824	4.783844	4.017351	5.024102	5.118821
14	H	2.882554	5.517109	4.584211	5.239155	5.220576
15	C	2.206578	4.510663	3.419933	4.699061	5.089343
16	H	2.931135	5.021403	3.494978	4.577764	5.115490
17	C	2.186769	3.906463	3.393405	5.044547	5.469622
18	H	4.677456	2.872866	5.051380	6.074388	5.923387
19	C	4.232813	2.124254	4.296617	5.123310	4.939958
20	C	4.693803	2.091227	4.013712	4.696891	4.691869
21	H	3.680536	2.928821	4.506144	4.969644	4.409376
22	H	5.451281	2.833935	4.599265	5.389307	5.531944
23	C	4.481963	2.103410	3.445103	3.605602	3.497615
24	H	5.085112	2.835889	3.594280	3.417302	3.451199
25	C	3.839051	2.134362	3.394256	3.397261	2.928230
26	H	3.983905	2.891157	3.521599	3.005720	2.251509
27	C	3.681905	2.166990	3.967943	4.455615	4.023749
28	C	2.958950	1.753990	2.951435	4.623902	4.981914
29	O	3.715634	2.896818	3.872234	5.638312	6.071072

31 O -0.115962 -0.038562	30 C	1.744863	3.169112	2.872959	3.136229	2.707109
	31 O	2.890890	3.939542	3.692808	3.396131	2.637309
		6	7	8	9	10
	6 H	0.000000				
	7 H	1.759896	0.000000			
	8 H	6.930564	6.697074	0.000000		
	9 C	6.398450	5.936838	1.079338	0.000000	
	10 C	6.525124	5.804260	2.227369	1.411185	0.000000
	11 H	5.859454	5.484748	2.679648	2.229999	3.332244
	12 H	7.174211	6.489154	2.695572	2.230744	1.079357
	13 C	6.036685	4.996013	3.339602	2.293250	1.430504
	14 H	6.307556	5.057629	4.348799	3.335095	2.239156
	15 C	5.594730	4.626073	3.334707	2.293888	2.297822
	16 H	5.425586	4.285536	4.340940	3.335210	3.341994
	17 C	5.799600	5.234168	2.225954	1.418030	2.290400
	18 H	6.276616	7.065361	4.381092	4.947616	5.572958
	19 C	5.278891	6.150068	4.624695	4.972249	5.487670
	20 C	4.569899	5.761557	5.441182	5.677916	6.302192
	21 H	5.466649	5.858111	4.588972	4.688354	4.604484
	22 H	5.124621	6.422692	5.898881	6.225891	7.019125
	23 C	3.397340	4.696842	5.963821	5.954544	6.349444
	24 H	2.893436	4.460703	6.779114	6.684349	7.086428
	25 C	3.562188	4.443626	5.565874	5.465652	5.573620
	26 H	3.263163	3.958715	6.116952	5.855033	5.733435
	27 C	4.783736	5.444672	4.731873	4.840985	4.995459
	28 C	4.931983	5.375231	2.947749	3.153450	4.201734
	29 O	5.928108	6.306720	2.818663	3.243635	4.524002
	30 C	4.114518	3.551832	4.321544	3.678571	3.100234
	31 O	4.324040	3.761997	5.359979	4.753833	4.017275
		11	12	13	14	15
	11 H	0.000000				
	12 H	4.346027	0.000000			
	13 C	3.325893	2.239090	0.000000		
	14 H	4.337091	2.690300	1.079566	0.000000	
	15 C	2.224939	3.338294	1.408856	2.224689	0.000000
	16 H	2.675602	4.348950	2.222911	2.683801	1.079021
	17 C	1.078529	3.336515	2.284689	3.329392	1.414665
	18 H	5.703584	5.659384	6.511793	7.297911	6.544148
	19 C	5.585695	5.655223	6.233513	6.949887	6.237882
	20 C	5.766127	6.650242	6.817537	7.536577	6.579094
	21 H	5.971753	4.454988	5.354215	5.811587	5.838478
	22 H	6.044146	7.430038	7.542597	8.330524	7.155698
	23 C	6.008286	6.723804	6.618707	7.184660	6.424217
	24 H	6.456259	7.544819	7.181893	7.701472	6.863221
	25 C	5.972903	5.796185	5.873406	6.316820	5.963473
	26 H	6.411163	5.922667	5.844557	6.110854	6.044115

	27	C	5.734338	5.059016	5.626983	6.171218	5.861023
	28	C	2.877767	4.854807	4.721799	5.677540	4.183872
	29	O	2.692958	5.200610	5.129700	6.164662	4.472045
	30	C	4.658534	3.371370	2.982064	3.151993	3.531552
	31	O	5.798344	4.075761	3.855661	3.757606	4.545651
			16	17	18	19	20
	16	H	0.000000				
	17	C	2.224026	0.000000			
	18	H	7.325000	5.610241	0.000000		
	19	C	6.923795	5.463696	1.079676	0.000000	
	20	C	7.083263	5.838810	2.235821	1.426549	0.000000
	21	H	6.608280	5.446160	2.682664	2.225378	3.343493
	22	H	7.616468	6.292710	2.690185	2.237545	1.079131
	23	C	6.796071	5.972031	3.335713	2.293143	1.415438
	24	H	7.079816	6.506909	4.347675	3.338199	2.228927
	25	C	6.432264	5.683019	3.330395	2.285996	2.291919
	26	H	6.424639	6.014433	4.337970	3.327160	3.333954
	27	C	6.536683	5.378749	2.224679	1.411126	2.299507
	28	C	4.781080	3.112341	3.051188	2.885407	2.991979
	29	O	5.079979	3.180637	3.578379	3.702170	3.839365
	30	C	4.056076	3.887311	5.048992	4.403843	4.908570
	31	O	4.978875	5.016241	5.609382	4.894289	5.393052
			21	22	23	24	25
	21	H	0.000000				
	22	H	4.351126	0.000000			
	23	C	3.338876	2.232507	0.000000		
	24	H	4.346974	2.692694	1.079508	0.000000	
	25	C	2.230499	3.336701	1.416056	2.227049	0.000000
	26	H	2.682554	4.346055	2.227843	2.681812	1.078301
	27	C	1.079200	3.340791	2.296302	3.338536	1.417905
	28	C	4.023131	3.250399	3.596346	4.242551	3.831925
	29	O	4.937672	3.853609	4.632197	5.239805	4.945736
	30	C	3.143721	5.865112	4.319817	4.895165	3.259722
	31	O	3.310842	6.415818	4.618163	5.132450	3.385587
			26	27	28	29	30
	26	H	0.000000				
	27	C	2.226967	0.000000			
	28	C	4.628078	3.458413	0.000000		
	29	O	5.767934	4.451565	1.146825	0.000000	
	30	C	2.974922	3.346514	4.203480	5.155342	0.000000
	31	O	2.781776	3.630543	5.196530	6.200369	1.146340
			31				
	31	O	0.000000				

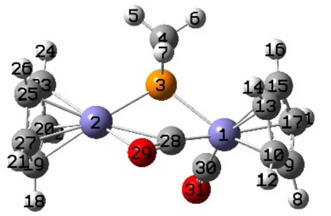

14T -3521.185229 14.39 C1
WBI 0.07

Charge and spin density

		1	2
1	Fe	0.225033	-0.032865
2	Fe	0.315614	2.151178
3	P	-0.169350	-0.076200
4	C	-0.026198	0.015503
9	C	-0.020517	0.002390
10	C	0.026842	0.001186
13	C	0.031416	-0.002084
15	C	-0.009895	0.002916
17	C	0.035281	-0.003230
19	C	-0.024166	0.080594
20	C	-0.014798	-0.024796
23	C	-0.002608	-0.051732
25	C	-0.076585	0.047372
27	C	0.010122	-0.057950
28	C	0.129952	-0.050341
29	O	-0.279942	-0.006339
30	C	-0.025353	0.002714
31	O	-0.124849	0.001683

		1	2	3	4	5
1	Fe	0.000000				
2	Fe	3.734666	0.000000			
3	P	2.346789	2.348534	0.000000		
4	C	3.369183	3.112152	1.870868	0.000000	
5	H	4.355758	3.262218	2.450945	1.092695	0.000000
6	H	3.523221	4.141859	2.460925	1.092475	1.767035
7	H	3.498163	3.123914	2.519830	1.092618	1.771968
8	H	2.834857	6.269211	5.127722	6.118361	7.148769
9	C	2.082333	5.722214	4.387678	5.179599	6.234399
10	C	2.086715	5.282867	4.233040	4.620842	5.704229
11	H	2.872885	6.359307	4.393158	5.283135	6.199458
12	H	2.832300	5.475194	4.864152	5.190782	6.242578
13	C	2.087525	5.021704	3.632143	3.569795	4.658175
14	H	2.845322	5.007067	3.852624	3.277934	4.309902
15	C	2.108948	5.355772	3.436876	3.622316	4.647500
16	H	2.859368	5.600516	3.471985	3.364000	4.266690
17	C	2.111917	5.777418	3.944741	4.668940	5.672139
18	H	5.776348	2.984652	4.927141	6.076818	6.139070
19	C	5.530562	2.240901	4.307877	5.280923	5.219171
20	C	5.278970	2.164574	3.636507	4.716786	4.531087
21	H	6.305521	2.931224	5.264911	5.580888	5.488940
22	H	5.336520	2.893002	3.777730	5.132891	5.000778
23	C	5.462366	2.099617	3.445470	3.971611	3.519460
24	H	5.714241	2.831307	3.469107	3.813261	3.160443
25	C	5.833063	2.162431	4.073785	4.207490	3.787078
26	H	6.349534	2.900979	4.565647	4.242090	3.680809
27	C	5.815868	2.191361	4.503304	4.971112	4.810796
28	C	1.749482	2.246815	2.062145	2.930943	3.745752
29	O	2.928476	1.956437	2.899781	3.409768	4.035363
30	C	1.753470	3.913090	2.866007	4.505141	5.288218
31	O	2.896324	4.448472	3.670267	5.446393	6.119591

		6	7	8	9	10
6	H	0.000000				
7	H	1.771135	0.000000			
8	H	6.060043	6.123801	0.000000		
9	C	5.049556	5.184444	1.079424	0.000000	
10	C	4.565554	4.354686	2.223404	1.410793	0.000000
11	H	4.878748	5.709675	2.699428	2.244386	3.345442
12	H	5.274292	4.731480	2.679960	2.224281	1.078963
13	C	3.316042	3.359963	3.332085	2.287851	1.421744
14	H	3.039544	2.818447	4.341684	3.330598	2.233516
15	C	3.121736	3.832180	3.333535	2.291977	2.301482
16	H	2.603263	3.773334	4.343645	3.335905	3.344281
17	C	4.319781	4.929827	2.241812	1.432436	2.303980
18	H	7.074393	6.075549	7.576835	7.421096	7.221822

19	C	6.311939	5.320536	7.664895	7.360262	7.078766
20	C	5.676073	5.034262	7.614647	7.229200	7.090069
21	H	6.663595	5.213240	8.451554	8.049934	7.388687
22	H	5.976713	5.609072	7.509013	7.200335	7.271610
23	C	4.959939	4.295934	8.085161	7.524373	7.212574
24	H	4.674027	4.348287	8.420727	7.788817	7.537210
25	C	5.279346	4.176077	8.424393	7.843382	7.296554
26	H	5.273496	4.089634	9.007764	8.341233	7.661387
27	C	6.059012	4.799408	8.122554	7.689446	7.155861
28	C	3.585161	2.757464	4.177215	3.565513	3.044083
29	O	4.245779	2.948663	5.074180	4.544238	3.810280
30	C	4.799583	4.823506	3.004145	2.825621	3.421597
31	O	5.783897	5.829066	3.571787	3.674815	4.436901
		11	12	13	14	15
11	H	0.000000				
12	H	4.355835	0.000000			
13	C	3.337136	2.234987	0.000000		
14	H	4.344909	2.692294	1.079026	0.000000	
15	C	2.221686	3.344840	1.422635	2.234331	0.000000
16	H	2.674027	4.354423	2.234398	2.690799	1.079253
17	C	1.079043	3.347822	2.292974	3.334297	1.406403
18	H	8.202499	7.188381	7.427073	7.592224	7.764982
19	C	8.014983	7.130046	7.077857	7.147126	7.383892
20	C	7.492923	7.348802	6.905276	7.026742	6.959619
21	H	9.092184	7.200855	7.335080	7.113869	7.995463
22	H	7.226466	7.625519	7.137266	7.399884	7.003787
23	C	7.721101	7.513381	6.764715	6.697102	6.845921
24	H	7.705023	7.961914	6.925297	6.847312	6.835601
25	C	8.379902	7.419986	6.879798	6.641976	7.236081
26	H	8.897956	7.764544	7.108799	6.713451	7.535967
27	C	8.496181	7.123316	7.013172	6.867829	7.499940
28	C	4.611684	3.289560	2.992666	3.216024	3.520655
29	O	5.801091	3.742210	3.829601	3.796234	4.595169
30	C	3.155408	4.002843	3.787354	4.596508	3.516612
31	O	3.772175	4.940673	4.909900	5.737779	4.558328
		16	17	18	19	20
16	H	0.000000				
17	C	2.216890	0.000000			
18	H	8.183717	7.768397	0.000000		
19	C	7.686577	7.558092	1.080157	0.000000	
20	C	7.101958	7.163946	2.217457	1.401191	0.000000
21	H	8.330686	8.414873	2.678600	2.227976	3.321685
22	H	7.128889	7.047606	2.679764	2.219048	1.079481
23	C	6.826015	7.321383	3.340124	2.293751	1.434665
24	H	6.649062	7.387908	4.345410	3.332750	2.240441
25	C	7.299488	7.820862	3.345035	2.303750	2.305791

26	H	7.521041	8.281308	4.352681	3.346226	3.352690
27	C	7.757114	7.903490	2.230107	1.419229	2.280298
28	C	4.085847	3.841592	4.440484	4.113144	4.122567
29	O	5.140708	4.986915	3.923068	3.599983	3.965104
30	C	4.152513	2.913202	5.132420	5.106914	4.798398
31	O	5.142761	3.780569	5.039059	5.178148	4.853270

		21	22	23	24	25
21	H	0.000000				
22	H	4.333904	0.000000			
23	C	3.324542	2.242276	0.000000		
24	H	4.339854	2.689604	1.079754	0.000000	
25	C	2.226453	3.346508	1.415908	2.233943	0.000000
26	H	2.682647	4.360548	2.234506	2.702263	1.079747
27	C	1.080642	3.326304	2.280032	3.327115	1.415178
28	C	4.591208	4.483536	4.240260	4.724025	4.323025
29	O	3.586347	4.568568	4.036367	4.718004	3.743293
30	C	6.402600	4.557653	5.401033	5.718526	6.017372
31	O	6.745659	4.405831	5.698818	6.041443	6.433483

		26	27	28	29	30
26	H	0.000000				
27	C	2.229172	0.000000			
28	C	4.839437	4.186612	0.000000		
29	O	4.206625	3.395432	1.216318	0.000000	
30	C	6.771665	5.806497	2.563080	3.585016	0.000000
31	O	7.298131	6.111841	3.502778	4.370391	1.143285

		31
31	O	0.000000

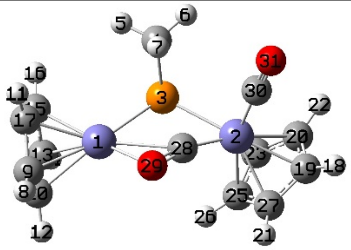

15T -3521.185001 14.53 C1
WBI 0.08

Charge and spin density

		1	2
1	Fe	0.332948	2.139628
2	Fe	0.259442	-0.024495
3	P	-0.187076	-0.071567
4	C	-0.010219	0.012711
9	C	0.019909	-0.046731
10	C	-0.031737	0.068821

		1	2	3	4	5
1	Fe	0.000000				
2	Fe	3.768967	0.000000			
3	P	2.348210	2.345080	0.000000		
4	C	3.163558	3.419848	1.868481	0.000000	
5	H	3.286672	4.391614	2.441889	1.092444	0.000000
6	H	4.188904	3.579692	2.460503	1.092202	1.771127
7	H	3.211736	3.576759	2.520862	1.091913	1.767971
8	H	2.935312	6.314447	5.271639	5.691695	5.599890
9	C	2.195572	5.850074	4.513210	5.026214	4.846950
10	C	2.243152	5.623293	4.300972	5.282652	5.153036
11	H	2.903020	6.327959	4.608487	4.264139	3.759643
12	H	2.990385	5.894652	4.910581	6.101833	6.084094
13	C	2.161777	5.389764	3.635069	4.624086	4.345039
14	H	2.892360	5.491716	3.769570	4.995827	4.741514
15	C	2.099446	5.524348	3.474893	3.861136	3.344206
16	H	2.834567	5.780260	3.519039	3.625393	2.878705
17	C	2.164444	5.848706	4.104562	4.205663	3.787916
18	H	6.439605	2.835658	5.155313	5.913094	6.975955

13	C	-0.031175	-0.019928
15	C	-0.021246	-0.053591
17	C	-0.087709	0.046856
19	C	-0.011039	0.002465
20	C	0.027359	-0.004686
23	C	0.038797	0.004769
25	C	0.029245	-0.006348
27	C	0.007050	0.001817
28	C	0.134708	-0.033263
29	O	-0.269732	-0.021950
30	C	-0.078130	0.003697
31	O	-0.121395	0.001795

19	C	5.703339	2.084052	4.362809	5.380371	6.393274
20	C	5.690343	2.119187	3.896219	4.960809	5.897234
21	H	5.165567	2.826278	4.773386	6.041437	6.950506
22	H	6.413273	2.880736	4.390385	5.170713	6.092101
23	C	4.941903	2.120584	3.282703	4.763265	5.548746
24	H	5.064619	2.858377	3.260236	4.763702	5.392153
25	C	4.425547	2.087066	3.438735	5.075331	5.856603
26	H	4.114339	2.842069	3.595307	5.381372	6.020223
27	C	4.979725	2.082466	4.132910	5.460468	6.384224
28	C	2.314642	1.742324	2.080673	3.035361	3.835548
29	O	1.970233	2.922535	2.898723	3.598150	4.203881
30	C	4.549640	1.750168	3.065127	3.102687	4.195096
31	O	5.339150	2.893997	3.925760	3.435294	4.483113
		6	7	8	9	10
6	H	0.000000				
7	H	1.769803	0.000000			
8	H	6.774861	5.368742	0.000000		
9	C	6.111828	4.886300	1.080697	0.000000	
10	C	6.306568	5.387868	2.227062	1.418035	0.000000
11	H	5.303203	4.026647	2.681492	2.229143	3.345963
12	H	7.090509	6.196433	2.677611	2.229045	1.080203
13	C	5.576253	4.986931	3.320987	2.279265	1.402213
14	H	5.824738	5.536893	4.333588	3.325473	2.220177
15	C	4.853198	4.167158	3.324417	2.280181	2.294962
16	H	4.492529	4.115094	4.339532	3.327135	3.333870
17	C	5.280524	4.139612	2.225912	1.415326	2.303799
18	H	5.761766	5.868509	8.604781	8.345809	8.136176
19	C	5.308893	5.479433	8.005444	7.655385	7.319426
20	C	4.731153	5.329756	8.313012	7.788113	7.343829
21	H	6.354560	5.962102	6.829546	6.679846	6.305166
22	H	4.714041	5.597570	9.133204	8.569441	8.173333
23	C	4.748900	5.282039	7.590844	6.972940	6.336549
24	H	4.708685	5.472401	7.817822	7.079156	6.344223
25	C	5.326607	5.400264	6.742973	6.249942	5.583813
26	H	5.797268	5.731424	6.222379	5.708030	4.869077
27	C	5.658931	5.538142	7.051978	6.744872	6.301426
28	C	3.685062	2.897977	4.617220	4.240634	4.237893
29	O	4.436154	3.225893	3.572945	3.394234	3.638654
30	C	2.948301	2.961122	6.952432	6.576640	6.680601
31	O	3.080147	3.108600	7.585743	7.268391	7.534630
		11	12	13	14	15
11	H	0.000000				
12	H	4.352256	0.000000			
13	C	3.351965	2.219224	0.000000		
14	H	4.359742	2.682320	1.079549	0.000000	
15	C	2.234552	3.341650	1.434418	2.241612	0.000000

16	H	2.702269	4.347000	2.240458	2.689059	1.079693
17	C	1.079681	3.345099	2.305010	3.345672	1.415905
18	H	8.930782	8.198223	8.094763	8.150061	8.322545
19	C	8.337634	7.369739	7.206629	7.184862	7.522448
20	C	8.313366	7.492747	6.960941	6.788576	7.236772
21	H	7.850133	6.102171	6.590628	6.682757	7.154438
22	H	8.882820	8.401977	7.674721	7.478815	7.825925
23	C	7.693830	6.434723	5.885695	5.586553	6.340557
24	H	7.711666	6.502626	5.681550	5.229681	6.136612
25	C	7.305528	5.512506	5.425884	5.232741	6.062069
26	H	7.011414	4.669623	4.780919	4.521290	5.626053
27	C	7.749637	6.211106	6.353233	6.341434	6.865879
28	C	4.812442	4.595371	4.255123	4.652756	4.310118
29	O	4.156803	3.985830	4.000225	4.623618	4.037767
30	C	6.536700	7.119810	6.430491	6.692922	6.197962
31	O	6.945345	8.044523	7.282921	7.614935	6.875612
		16	17	18	19	20
16	H	0.000000				
17	C	2.233780	0.000000			
18	H	8.612942	8.515143	0.000000		
19	C	7.810572	7.831408	1.079447	0.000000	
20	C	7.351972	7.780162	2.243154	1.433222	0.000000
21	H	7.730036	7.239369	2.678861	2.223643	3.347564
22	H	7.810563	8.421162	2.700160	2.244457	1.079021
23	C	6.491647	7.025375	3.334183	2.292555	1.405363
24	H	6.154850	7.011659	4.344905	3.336607	2.217913
25	C	6.446031	6.568309	3.332996	2.288545	2.291131
26	H	6.108347	6.166348	4.343561	3.332003	3.332278
27	C	7.313306	7.129284	2.222383	1.409986	2.303527
28	C	4.786670	4.343955	4.178283	3.561365	3.838400
29	O	4.718289	3.718679	5.078773	4.553830	4.994254
30	C	6.313931	6.337350	2.998643	2.823664	2.931161
31	O	6.914097	6.911670	3.567211	3.674833	3.802820
		21	22	23	24	25
21	H	0.000000				
22	H	4.355209	0.000000			
23	C	3.345482	2.220857	0.000000		
24	H	4.352637	2.676889	1.078836	0.000000	
25	C	2.237941	3.335207	1.420798	2.228900	0.000000
26	H	2.697739	4.342510	2.231769	2.681988	1.079370
27	C	1.078946	3.344667	2.302175	3.343079	1.424435
28	C	3.271869	4.613232	3.514085	4.063351	2.971250
29	O	3.766965	5.802422	4.615341	5.143597	3.855255
30	C	3.979883	3.183097	3.534998	4.164856	3.784072
31	O	4.915656	3.807905	4.579901	5.160645	4.908207
		26	27	28	29	30

26	H	0.000000				
27	C	2.237447	0.000000			
28	C	3.186661	3.027117	0.000000		
29	O	3.828178	3.830197	1.210187	0.000000	
30	C	4.589052	3.407202	2.561728	3.537642	0.000000
31	O	5.731168	4.423037	3.503445	4.308897	1.144179

		31
31	O	0.000000

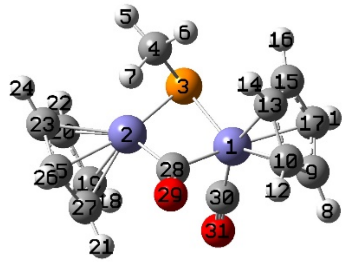

16S -3521.184988 14.54 C1
WBI 0.30

Charge and spin density

		1
1	Fe	0.016641
2	Fe	-0.089769
3	P	-0.148937
4	C	0.087032
9	C	-0.014705
10	C	0.090630
13	C	-0.027646
15	C	0.036875
17	C	0.020625
19	C	0.046763
20	C	0.061717
23	C	0.012387
25	C	0.023164
27	C	-0.006027
28	C	0.243297
29	O	-0.218267
30	C	0.012475
31	O	-0.146259

		1	2	3	4	5
1	Fe	0.000000				
2	Fe	2.584856	0.000000			
3	P	2.248325	2.169684	0.000000		
4	C	3.061363	2.220382	1.855229	0.000000	
5	H	4.100461	2.883238	2.489484	1.090117	0.000000
6	H	3.151640	3.023093	2.507687	1.090054	1.792195
7	H	3.168020	1.736422	2.552855	1.165553	1.798650
8	H	2.829423	5.261109	4.842017	5.813031	6.857030
9	C	2.091613	4.637828	4.104217	4.895326	5.944805
10	C	2.110764	4.267303	4.194703	4.446806	5.528900
11	H	2.829911	5.136173	3.645166	4.864986	5.701306
12	H	2.850339	4.617901	4.980267	5.074229	6.152340
13	C	2.127819	3.938370	3.568240	3.438238	4.465025
14	H	2.899814	4.070263	3.986684	3.315790	4.253953
15	C	2.115296	4.138901	2.995920	3.337546	4.265444
16	H	2.883387	4.434465	2.955843	3.113431	3.843543
17	C	2.081640	4.553374	3.377348	4.308507	5.254456
18	H	4.003102	2.824992	3.504893	4.633544	5.043953
19	C	3.882272	2.068581	3.313059	4.023862	4.394480
20	C	4.381839	2.083133	3.156135	3.433142	3.471466
21	H	4.021105	2.813619	4.600415	5.011040	5.657682
22	H	4.884808	2.846355	3.220223	3.624214	3.419716
23	C	4.665775	2.081061	3.745641	3.319695	3.331640
24	H	5.334147	2.821087	4.221516	3.377735	3.102826
25	C	4.407116	2.085989	4.205101	3.874304	4.217456
26	H	4.918816	2.845323	4.991468	4.381592	4.746218
27	C	3.899101	2.065267	3.963102	4.260031	4.775508
28	C	1.970161	1.865157	3.170938	3.141461	4.107870
29	O	2.910079	2.869454	4.331084	4.095938	5.027364
30	C	1.743013	3.025976	2.875384	4.243277	5.132671
31	O	2.889269	3.785909	3.712189	5.212809	6.013421

		6	7	8	9	10
6	H	0.000000				
7	H	1.716758	0.000000			
8	H	5.655843	5.913318	0.000000		
9	C	4.642485	5.051711	1.079625	0.000000	
10	C	4.052962	4.373488	2.220209	1.407589	0.000000

11	H	4.693673	5.487490	2.694764	2.239115	3.337713
12	H	4.709697	4.768055	2.677804	2.222209	1.078658
13	C	2.820274	3.547873	3.335609	2.291498	1.425160
14	H	2.522013	3.266229	4.342047	3.332149	2.233345
15	C	2.811776	3.869711	3.337560	2.294466	2.294730
16	H	2.502924	3.901406	4.345861	3.336721	3.340102
17	C	4.033399	4.785024	2.233842	1.424012	2.295152
18	H	5.542878	4.495275	5.850228	5.680089	5.903511
19	C	4.966822	3.696345	6.069081	5.755438	5.722816
20	C	4.490822	3.185612	6.927269	6.428393	6.270884
21	H	5.674461	4.301212	5.605780	5.469577	5.187049
22	H	4.712707	3.682216	7.441604	6.915130	6.879537
23	C	4.265400	2.663375	7.298188	6.711512	6.252357
24	H	4.256858	2.749279	8.063875	7.381964	6.821102
25	C	4.641832	2.960614	6.759855	6.279072	5.710825
26	H	4.977144	3.304973	7.134306	6.640695	5.875828
27	C	5.049155	3.575792	5.953025	5.653848	5.350463
28	C	3.376274	2.504988	4.097955	3.513623	2.813458
29	O	4.184004	3.288579	4.418377	3.948912	3.000790
30	C	4.642561	4.297656	2.900633	2.780609	3.332587
31	O	5.712140	5.251107	3.431951	3.616195	4.324148
		11	12	13	14	15
11	H	0.000000				
12	H	4.348045	0.000000			
13	C	3.336720	2.234421	0.000000		
14	H	4.348899	2.684914	1.079365	0.000000	
15	C	2.232272	3.335605	1.412628	2.229907	0.000000
16	H	2.681420	4.347993	2.228538	2.693077	1.079448
17	C	1.078743	3.338430	2.294741	3.339777	1.419958
18	H	5.807445	6.248984	6.025492	6.501416	5.876574
19	C	6.073758	6.001741	5.712999	6.023313	5.734304
20	C	6.516595	6.638241	5.936504	6.071846	5.889749
21	H	6.561356	5.061834	5.590338	5.850464	6.072369
22	H	6.656498	7.369943	6.423550	6.588157	6.162669
23	C	7.125268	6.467432	5.841819	5.739803	6.083046
24	H	7.719987	7.053481	6.224599	5.963809	6.474666
25	C	7.114034	5.713063	5.569853	5.484840	6.068805
26	H	7.718055	5.709409	5.760730	5.522056	6.474543
27	C	6.473214	5.385097	5.480837	5.665636	5.847576
28	C	4.784823	2.884658	2.898276	3.074052	3.621543
29	O	5.595458	2.636928	3.337473	3.341560	4.362627
30	C	3.255450	3.845598	3.797548	4.611965	3.609896
31	O	3.920289	4.735188	4.917433	5.748217	4.674677
		16	17	18	19	20
16	H	0.000000				
17	C	2.227281	0.000000			

18	H	6.237882	5.643512	0.000000		
19	C	6.063348	5.745689	1.078730	0.000000	
20	C	5.985667	6.185932	2.235421	1.422105	0.000000
21	H	6.705686	5.994432	2.680292	2.225479	3.335433
22	H	6.095770	6.466959	2.694695	2.234875	1.078813
23	C	6.200119	6.602933	3.335291	2.291547	1.417118
24	H	6.452119	7.176538	4.344622	3.334401	2.225742
25	C	6.416270	6.473219	3.348106	2.306093	2.303997
26	H	6.867023	6.969228	4.356228	3.348031	3.347389
27	C	6.327578	5.937342	2.228694	1.416075	2.292710
28	C	4.295089	3.938547	3.901890	3.354194	3.846363
29	O	5.120861	4.666229	4.736827	4.153009	4.711338
30	C	4.316774	2.967606	2.968976	3.278127	4.222039
31	O	5.349979	3.856384	2.760804	3.392765	4.537774

		21	22	23	24	25
21	H	0.000000				
22	H	4.345266	0.000000			
23	C	3.326753	2.231643	0.000000		
24	H	4.337651	2.682720	1.080609	0.000000	
25	C	2.233188	3.345720	1.414720	2.224089	0.000000
26	H	2.692085	4.355765	2.232909	2.686598	1.078774
27	C	1.079481	3.335979	2.283325	3.327748	1.423713
28	C	2.776543	4.685266	3.599441	4.277616	2.900768
29	O	2.950635	5.641714	4.233420	4.855983	3.236150
30	C	3.451203	4.715690	4.873819	5.769250	4.534966
31	O	3.566574	4.983293	5.366408	6.344498	5.004104

		26	27	28	29	30
26	H	0.000000				
27	C	2.237717	0.000000			
28	C	3.135818	2.704838	0.000000		
29	O	3.099360	3.166905	1.169412	0.000000	
30	C	5.227720	3.525510	2.776940	3.621362	0.000000
31	O	5.757893	3.774621	3.701103	4.433942	1.146840

		31
31	O	0.000000

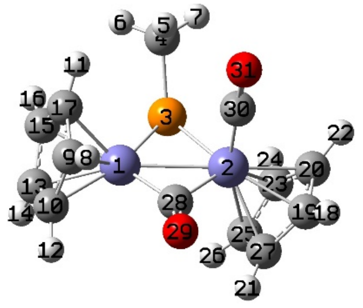

17S -3521.182587 16.05 C1
WBI 0.33

		1	2	3	4	5
1	Fe	0.000000				
2	Fe	2.524899	0.000000			
3	P	2.172887	2.235788	0.000000		
4	C	3.334457	3.508027	1.856211	0.000000	
5	H	3.412173	3.613661	2.515700	1.090301	0.000000
6	H	3.686683	4.400361	2.432876	1.093393	1.778446
7	H	4.258903	3.882170	2.437175	1.092984	1.772960
8	H	2.802895	4.399507	4.683000	5.086151	4.553684
9	C	2.057407	4.154208	4.016117	4.530734	4.187619
10	C	2.086910	4.374898	4.190258	5.141747	5.070048

Charge and spin density

		1
1	Fe	0.172718
2	Fe	-0.045407
3	P	-0.213749
4	C	-0.020984
9	C	0.026523
10	C	0.009813
13	C	0.018645
15	C	0.017412
17	C	0.013816
19	C	-0.028353
20	C	0.011567
23	C	0.055605
25	C	0.017817
27	C	0.064377
28	C	0.192587
29	O	-0.194749
30	C	0.016106
31	O	-0.113744

11	H	2.785890	4.462196	3.575027	3.113834	2.567433
12	H	2.843046	4.786566	4.961197	6.084724	6.014315
13	C	2.062483	4.511877	3.659733	4.616397	4.801460
14	H	2.797308	5.006043	4.074920	5.219571	5.577935
15	C	2.062159	4.426056	3.107044	3.535816	3.677882
16	H	2.829335	4.904947	3.145404	3.302395	3.656221
17	C	2.042086	4.186240	3.356377	3.457393	3.168722
18	H	5.044089	2.831713	5.024792	6.014117	5.881513
19	C	4.438147	2.090361	4.205977	5.382840	5.425788
20	C	4.559581	2.069262	3.626919	4.633779	4.844059
21	H	4.110364	2.859800	4.702077	6.282027	6.395348
22	H	5.263695	2.817653	4.100683	4.708703	4.848638
23	C	4.152047	2.116468	2.997055	4.356800	4.851612
24	H	4.561554	2.869073	2.964542	4.157625	4.842730
25	C	3.697903	2.131628	3.265905	4.956013	5.399286
26	H	3.756471	2.895376	3.468814	5.285362	5.845602
27	C	3.900744	2.113243	4.005231	5.542680	5.725707
28	C	1.857772	1.943443	3.224895	4.428044	4.280010
29	O	2.871647	2.891776	4.394512	5.517531	5.248532
30	C	3.140762	1.742459	2.857998	3.148794	2.756901
31	O	3.937730	2.887442	3.678324	3.406270	2.700372

		6	7	8	9	10
6	H	0.000000				
7	H	1.759195	0.000000			
8	H	5.387673	6.073237	0.000000		
9	C	4.699394	5.574024	1.079163	0.000000	
10	C	5.230369	6.165418	2.230520	1.420526	0.000000
11	H	3.090758	4.150239	2.679272	2.227266	3.342418
12	H	6.242656	7.070123	2.687815	2.233699	1.078807
13	C	4.485752	5.624143	3.326226	2.283615	1.413770
14	H	5.022388	6.148036	4.335235	3.326859	2.223360
15	C	3.252688	4.590473	3.340535	2.298385	2.306119
16	H	2.724652	4.272851	4.352315	3.342848	3.346687
17	C	3.430353	4.543705	2.228728	1.417881	2.301026
18	H	7.027810	6.138610	5.952404	6.144322	6.415354
19	C	6.357935	5.498578	5.833586	5.834213	5.970997
20	C	5.626689	4.514317	6.362268	6.208456	6.406905
21	H	7.052331	6.689585	5.407765	5.333951	5.004431
22	H	5.748639	4.382004	6.921678	6.823363	7.185049
23	C	5.185852	4.291234	6.446771	6.065538	6.015869
24	H	4.880503	3.916916	7.049597	6.555988	6.495491
25	C	5.680000	5.166222	5.952622	5.553220	5.241931
26	H	5.837935	5.576056	6.177705	5.637417	5.074540
27	C	6.373010	5.827666	5.551718	5.401812	5.215272
28	C	5.097587	5.152222	2.830520	2.739945	2.887867
29	O	6.190550	6.237232	2.849281	3.103561	3.230147

	30	C	4.159855	3.485603	4.115070	4.136977	4.882403
	31	O	4.382856	3.668234	4.331532	4.519165	5.507687
			11	12	13	14	15
	11	H	0.000000				
	12	H	4.350045	0.000000			
	13	C	3.333477	2.230135	0.000000		
	14	H	4.342452	2.682852	1.080474	0.000000	
	15	C	2.235690	3.348798	1.420264	2.227913	0.000000
	16	H	2.702428	4.355439	2.232900	2.682744	1.078559
	17	C	1.079628	3.343163	2.288131	3.330561	1.421198
	18	H	6.753363	6.471528	6.952099	7.433167	7.063199
	19	C	6.394133	6.089955	6.322591	6.711675	6.443424
	20	C	6.320547	6.723445	6.479360	6.836115	6.368294
	21	H	6.590782	4.775839	5.498221	5.693335	6.100801
	22	H	6.644233	7.581684	7.238169	7.660877	6.953548
	23	C	6.221991	6.356757	5.831758	6.002271	5.804466
	24	H	6.444180	6.940919	6.088074	6.181102	5.925168
	25	C	6.198074	5.391330	5.169720	5.234663	5.476707
	26	H	6.407999	5.171499	4.830901	4.682945	5.309251
	27	C	6.307184	5.203527	5.519315	5.747269	5.904898
	28	C	3.931524	3.097439	3.554271	4.198582	3.837766
	29	O	4.637763	3.106893	4.227055	4.862575	4.684426
	30	C	3.879587	5.445227	5.171257	5.919917	4.712417
	31	O	3.900042	6.124756	5.857946	6.706732	5.222854
			16	17	18	19	20
	16	H	0.000000				
	17	C	2.238647	0.000000			
	18	H	7.669411	6.565234	0.000000		
	19	C	6.970225	6.134155	1.078974	0.000000	
	20	C	6.686904	6.182615	2.241904	1.427159	0.000000
	21	H	6.791473	5.994015	2.672903	2.217364	3.329577
	22	H	7.196563	6.672720	2.701433	2.239091	1.079379
	23	C	6.006534	5.936534	3.346515	2.303143	1.417460
	24	H	5.931955	6.214051	4.356426	3.345807	2.229709
	25	C	5.844055	5.690120	3.342537	2.299513	2.291108
	26	H	5.624289	5.768463	4.350322	3.341083	3.334139
	27	C	6.474040	5.820993	2.223572	1.409062	2.286640
	28	C	4.675161	3.380873	3.535548	3.172777	3.814252
	29	O	5.620244	4.086957	3.563209	3.496478	4.465024
	30	C	5.184180	4.012302	3.191224	2.953454	2.837248
	31	O	5.661454	4.304424	3.863259	3.858300	3.713133
			21	22	23	24	25
	21	H	0.000000				
	22	H	4.339624	0.000000			
	23	C	3.333174	2.228514	0.000000		
	24	H	4.342123	2.684597	1.078176	0.000000	

	25 C 2.232782 3.333468 1.413787 2.224177 0.000000<br>26 H 2.691647 4.342772 2.226623 2.680157 1.078906<br>27 C 1.079013 3.330022 2.290979 3.333474 1.422446<br>28 C 2.731290 4.595958 3.853138 4.642271 3.221153<br>29 O 2.710922 5.236975 4.693919 5.590672 3.934531<br>30 C 4.204521 3.006057 3.441348 4.012121 3.826389<br>31 O 5.222424 3.605015 4.464945 4.948881 4.956613<br>26 27 28 29 30<br>26 H 0.000000<br>27 C 2.236131 0.000000<br>28 C 3.621828 2.727307 0.000000<br>29 O 4.344307 3.072986 1.171239 0.000000<br>30 C 4.630835 3.562907 2.612971 3.341645 0.000000<br>31 O 5.772695 4.628489 3.492721 4.050579 1.145050<br>31<br>31 O 0.000000

Table S4. Distance matrix for the lowest energy $Cp_2Fe_2PMe(CO)_3$ structures as optimized at the PBE0/def2TZVP level. Included are the ranking order, spin multiplicity (S, T or Q), total energy (in a.u.), relative energy (in kcal/mol), symmetry point group Wiberg bond indices for the Fe-Fe bonds, Mulliken charges and the spin density (Cp moieties are omitted for clarity).

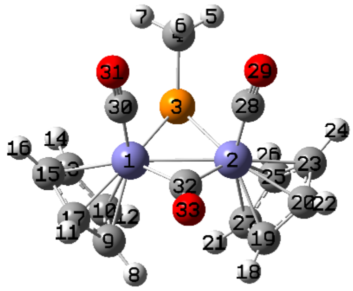

1S -3634.471791 0.00 Cs
WBI 0.30

Charge and spin density

		1
1	Fe	-0.025282
2	Fe	0.012789
3	P	-0.109438
4	C	-0.013926
9	C	-0.006400
10	C	0.022181
13	C	0.063971
15	C	-0.000824
17	C	0.063682
19	C	0.013543
20	C	-0.008519
23	C	0.010520
25	C	0.068739
27	C	0.032354
28	C	-0.004229
29	O	-0.094523
30	C	-0.048497
31	O	-0.096131
32	C	0.302849
33	O	-0.182857

		1	2	3	4	5
1	Fe	0.000000				
2	Fe	2.580775	0.000000			
3	P	2.243715	2.248135	0.000000		
4	C	3.471449	3.467627	1.858866	0.000000	
5	H	4.370626	3.813074	2.418743	1.094226	0.000000
6	H	3.624459	3.618597	2.562309	1.089639	1.772576
7	H	3.819354	4.369096	2.417760	1.094200	1.756150
8	H	2.861567	3.777127	4.468718	6.117563	6.831927
9	C	2.114551	3.714883	3.879896	5.449114	6.250658
10	C	2.126807	3.718729	3.170987	4.814344	5.544916
11	H	2.834145	4.835163	5.075241	6.141235	7.118977
12	H	2.875284	3.784123	3.229970	4.996786	5.547662
13	C	2.105974	4.314206	3.153958	4.382318	5.253702
14	H	2.855522	4.843826	3.216938	4.198667	5.003437
15	C	2.076622	4.637531	3.857829	4.825036	5.831261
16	H	2.819396	5.386997	4.442354	5.038067	6.099562
17	C	2.096110	4.314890	4.258494	5.466264	6.420788
18	H	4.077262	2.862227	4.780623	6.273874	6.655269
19	C	3.880737	2.114437	4.075231	5.539953	5.797977
20	C	4.469784	2.086220	4.223655	5.312206	5.390415
21	H	3.674070	2.907413	3.625405	5.434951	5.712336
22	H	5.098611	2.826415	5.019421	5.889417	5.969751
23	C	4.598554	2.069756	3.636742	4.599512	4.442689
24	H	5.324951	2.815337	4.073603	4.635567	4.260617
25	C	4.144009	2.121245	3.066051	4.429285	4.346597
26	H	4.540466	2.869814	3.030738	4.284757	4.046173
27	C	3.650197	2.137311	3.377731	5.039962	5.231523
28	C	3.358920	1.740527	2.813762	2.998815	3.271155
29	O	4.237233	2.882901	3.647120	3.250154	3.411278
30	C	1.741890	3.352805	2.807751	3.001726	4.040619
31	O	2.884536	4.225018	3.637800	3.247445	4.260465
32	C	1.906823	1.908416	3.195930	4.270990	4.977042
33	O	2.876597	2.880077	4.357424	5.304451	6.012412

		6	7	8	9	10
6	H	0.000000				
7	H	1.772612	0.000000			
8	H	6.354752	6.526510	0.000000		
9	C	5.718826	5.724434	1.079441	0.000000	
10	C	5.326111	4.945608	2.228138	1.415193	0.000000

11	H	6.050685	6.345649	2.680210	2.230844	3.340655
12	H	5.661990	5.168310	2.694653	2.233794	1.078140
13	C	4.858046	4.240844	3.333513	2.290325	1.415211
14	H	4.819078	3.826645	4.342128	3.332514	2.226979
15	C	4.999955	4.725076	3.326819	2.284284	2.288186
16	H	5.090266	4.783110	4.337172	3.328358	3.331266
17	C	5.544467	5.619610	2.223561	1.416280	2.298189
18	H	6.383285	7.076753	2.902526	3.654834	4.333669
19	C	5.731202	6.392623	3.350677	3.878274	4.229360
20	C	5.376795	6.302251	4.609874	4.998360	5.255973
21	H	5.991659	6.010221	2.905466	3.197066	2.923781
22	H	5.771692	6.921925	5.241899	5.679432	6.104484
23	C	4.852805	5.588834	5.234340	5.417280	5.298908
24	H	4.832303	5.662675	6.260497	6.374429	6.184810
25	C	4.954125	5.248968	4.608023	4.714811	4.335038
26	H	4.998727	4.982991	5.233222	5.193887	4.526992
27	C	5.492729	5.777069	3.346346	3.650327	3.524966
28	C	2.678051	4.038382	5.197616	5.009668	5.059343
29	O	2.618125	4.263166	6.238351	6.012945	6.081567
30	C	2.683303	3.276295	4.414927	3.681977	3.754338
31	O	2.616396	3.412105	5.490071	4.764232	4.855077
32	C	4.118878	4.978307	2.913862	2.832218	3.467143
33	O	5.000683	6.012719	3.196753	3.345312	4.300962
		11	12	13	14	15
11	H	0.000000				
12	H	4.351765	0.000000			
13	C	3.346290	2.219168	0.000000		
14	H	4.356134	2.670575	1.078448	0.000000	
15	C	2.235526	3.327699	1.418856	2.233589	0.000000
16	H	2.691733	4.335922	2.229149	2.688444	1.079813
17	C	1.078838	3.341947	2.303083	3.345784	1.419720
18	H	4.815143	4.334703	5.470829	6.315743	5.597039
19	C	5.248535	4.084359	5.314875	6.026871	5.634345
20	C	6.126056	5.128788	6.150071	6.758856	6.436105
21	H	5.257936	2.303142	4.198331	4.743679	4.987814
22	H	6.496560	6.085432	6.936517	7.592687	7.052457
23	C	6.772396	5.000338	6.081958	6.479571	6.612532
24	H	7.625949	5.888241	6.835324	7.131221	7.368876
25	C	6.419780	3.836682	5.211590	5.536421	5.978534
26	H	7.005199	3.882438	5.278560	5.397672	6.236245
27	C	5.458851	3.086181	4.653793	5.200154	5.312806
28	C	5.549728	5.285566	5.276835	5.686012	5.357353
29	O	6.283418	6.359931	6.142328	6.482735	6.109342
30	C	3.436339	4.523555	3.212652	3.662522	2.727419
31	O	4.163921	5.632860	4.158704	4.468626	3.540196
32	C	3.186259	4.011596	3.917955	4.737266	3.655502

33	O	3.128053	4.911079	4.773230	5.679347	4.249925
		16	17	18	19	20
16	H	0.000000				
17	C	2.230493	0.000000			
18	H	6.529278	4.597260	0.000000		
19	C	6.560971	4.863290	1.079344	0.000000	
20	C	7.253643	5.794422	2.217022	1.409430	0.000000
21	H	6.025460	4.513055	2.691607	2.231756	3.335526
22	H	7.800604	6.326315	2.673058	2.224658	1.079070
23	C	7.406119	6.253751	3.330567	2.287740	1.427420
24	H	8.082489	7.117781	4.339808	3.330762	2.238492
25	C	6.855359	5.726777	3.336383	2.293170	2.303415
26	H	7.078253	6.201196	4.345525	3.335601	3.346159
27	C	6.289991	4.811673	2.234702	1.420790	2.295275
28	C	5.833783	5.206122	4.189685	3.535188	2.888088
29	O	6.430070	6.038514	5.182605	4.571243	3.740024
30	C	2.824281	3.083296	5.287073	5.056370	5.256419
31	O	3.311552	3.993185	6.241703	6.008590	6.036068
32	C	4.328770	2.981724	2.817118	2.780562	3.172162
33	O	4.835416	3.307836	2.878762	3.190570	3.550224
		21	22	23	24	25
21	H	0.000000				
22	H	4.345713	0.000000			
23	C	3.328683	2.241233	0.000000		
24	H	4.338169	2.698743	1.079490	0.000000	
25	C	2.223230	3.346307	1.416625	2.228336	0.000000
26	H	2.678696	4.356045	2.229203	2.684997	1.078262
27	C	1.077746	3.339040	2.286794	3.329819	1.412481
28	C	4.638579	3.115768	2.767979	2.918002	3.406256
29	O	5.775573	3.715501	3.592104	3.443686	4.402844
30	C	5.268013	5.650706	5.337171	5.808926	5.220992
31	O	6.372558	6.298092	6.093905	6.420193	6.132098
32	C	3.702738	3.523595	3.795364	4.553521	3.860290
33	O	4.469266	3.600726	4.489407	5.229617	4.744194
		26	27	28	29	30
26	H	0.000000				
27	C	2.224478	0.000000			
28	C	3.979722	3.811008	0.000000		
29	O	4.896027	4.923962	1.143617	0.000000	
30	C	5.580248	5.033687	3.188973	3.631598	0.000000
31	O	6.463350	6.068539	3.622017	3.714637	1.143635
32	C	4.637946	3.273256	2.560098	3.451710	2.553203
33	O	5.624372	4.035269	3.269978	3.962278	3.260771
		31	32	33		
31	O	0.000000				
32	C	3.441942	0.000000			

33	O	3.950108	1.169150	0.000000

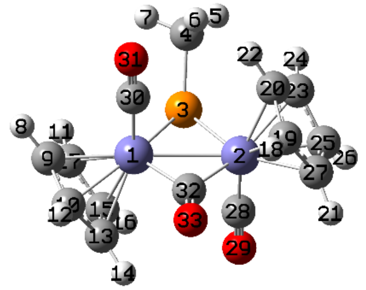

2S -3634.466521 3.31 C1
WBI 0.30

Charge and spin density

		1
1	Fe	-0.064183
2	Fe	-0.046928
3	P	-0.083738
4	C	-0.047289
9	C	-0.004257
10	C	0.022487
13	C	0.060172
15	C	0.084479
17	C	0.067734
19	C	0.081506
20	C	0.009854
23	C	0.064810
25	C	0.036151
27	C	-0.035220
28	C	0.070977
29	O	-0.108324
30	C	-0.004340
31	O	-0.114058
32	C	0.194488
33	O	-0.184322

		1	2	3	4	5
1	Fe	0.000000				
2	Fe	2.591601	0.000000			
3	P	2.246134	2.261323	0.000000		
4	C	3.429940	3.403973	1.861310	0.000000	
5	H	4.368729	3.867857	2.437377	1.093690	0.000000
6	H	3.605045	3.458671	2.558518	1.089985	1.779151
7	H	3.679158	4.282945	2.413361	1.094618	1.751971
8	H	2.814268	5.363603	4.359439	4.837189	5.875032
9	C	2.074400	4.660224	3.783801	4.658721	5.658334
10	C	2.095435	4.409658	4.231704	5.377204	6.362792
11	H	2.854449	4.830712	3.071822	3.957801	4.693323
12	H	2.836380	4.934845	5.071884	6.080355	7.102670
13	C	2.126536	3.891259	3.871023	5.401376	6.244527
14	H	2.874858	4.017046	4.490944	6.118363	6.898932
15	C	2.133077	3.841741	3.110777	4.715618	5.453136
16	H	2.884835	3.929865	3.178677	4.925630	5.479507
17	C	2.102472	4.341977	3.042885	4.194960	5.039846
18	H	4.264874	2.880101	4.793772	5.205302	5.749502
19	C	4.051191	2.131950	4.093043	4.520454	4.930730
20	C	3.877498	2.130418	3.371659	3.325381	3.659013
21	H	5.089166	2.840891	5.053420	6.010374	6.250585
22	H	3.981108	2.898608	3.605413	3.081977	3.535101
23	C	4.273302	2.096696	3.054597	3.155446	3.115732
24	H	4.703865	2.862598	3.039721	2.735823	2.358221
25	C	4.639295	2.067204	3.661957	4.315988	4.285344
26	H	5.302630	2.807955	4.100353	4.877256	4.675355
27	C	4.515739	2.094710	4.248427	5.017316	5.241974
28	C	3.145315	1.749589	2.810949	4.472637	4.803309
29	O	3.939327	2.892502	3.627839	5.379477	5.633686
30	C	1.740305	3.187603	2.876573	3.040342	4.100673
31	O	2.885465	3.986891	3.714071	3.315433	4.333670
32	C	1.904929	1.909267	3.243152	4.420738	5.204583
33	O	2.872706	2.880871	4.412201	5.528777	6.320743

		6	7	8	9	10
6	H	0.000000				
7	H	1.767943	0.000000			
8	H	4.970483	4.417088	0.000000		
9	C	4.904915	4.411265	1.079751	0.000000	
10	C	5.510321	5.379879	2.232425	1.421200	0.000000
11	H	4.658756	3.508387	2.687073	2.231923	3.344513
12	H	6.046508	6.116624	2.694923	2.237379	1.078779
13	C	5.708084	5.556118	3.330451	2.286211	1.417163
14	H	6.380413	6.407284	4.340133	3.329257	2.225443
15	C	5.265971	4.766489	3.334984	2.291686	2.298179

16	H	5.612111	5.056068	4.343078	3.333686	3.340914
17	C	4.737714	3.953394	2.227589	1.416972	2.301457
18	H	4.627892	6.067157	6.508892	6.064629	5.488522
19	C	4.053971	5.477862	6.530500	6.022549	5.596671
20	C	2.778686	4.323886	6.200311	5.812787	5.728162
21	H	5.846790	6.996636	7.814245	7.066579	6.383399
22	H	2.241637	3.950550	5.899812	5.698521	5.771926
23	C	3.004080	4.247189	6.763732	6.262928	6.276432
24	H	2.748443	3.803549	6.993499	6.558953	6.784522
25	C	4.303142	5.376050	7.368327	6.701037	6.465275
26	H	5.044690	5.905831	8.050047	7.314217	7.094356
27	C	4.812169	6.031146	7.237530	6.563778	6.065761
28	C	4.854973	5.207781	5.855200	4.930991	4.544673
29	O	5.879566	6.031868	6.457051	5.454967	5.014056
30	C	2.726095	3.233133	2.873751	2.766400	3.066691
31	O	2.680906	3.431294	3.408318	3.609853	3.987292
32	C	4.318505	5.022970	4.397877	3.675479	2.976841
33	O	5.318057	6.118880	4.957065	4.278438	3.267677
		11	12	13	14	15
11	H	0.000000				
12	H	4.355373	0.000000			
13	C	3.330916	2.231271	0.000000		
14	H	4.339927	2.682186	1.079484	0.000000	
15	C	2.230119	3.339963	1.411891	2.222652	0.000000
16	H	2.682755	4.348964	2.227294	2.681449	1.078858
17	C	1.078327	3.344860	2.289387	3.332316	1.419795
18	H	7.029357	5.443927	5.455787	5.369206	6.008923
19	C	6.644975	5.756696	5.391726	5.362637	5.706747
20	C	6.120314	6.038385	5.626775	5.846401	5.649204
21	H	7.532541	6.595036	5.728716	5.325180	6.084262
22	H	6.095193	6.023751	5.919278	6.280675	5.937847
23	C	6.094534	6.776220	5.907867	6.109494	5.654972
24	H	6.064669	7.379364	6.434582	6.754320	5.966108
25	C	6.597325	6.945928	5.845162	5.804829	5.704798
26	H	6.968785	7.656356	6.305303	6.205793	6.033021
27	C	6.921211	6.349169	5.527193	5.326938	5.737872
28	C	4.797305	5.219087	3.522491	3.402721	3.314772
29	O	5.149833	5.730480	3.773775	3.488182	3.484752
30	C	3.751847	3.396504	3.676442	4.390152	3.785864
31	O	4.602861	4.124269	4.762443	5.458167	4.901795
32	C	4.691065	3.235157	2.720143	2.762038	3.333135
33	O	5.621507	3.171257	3.116259	2.853382	4.082182
		16	17	18	19	20
16	H	0.000000				
17	C	2.229570	0.000000			
18	H	6.395699	6.363711	0.000000		

19	C	5.952813	6.088114	1.079028	0.000000	
20	C	5.889788	5.766827	2.224506	1.413959	0.000000
21	H	6.033168	6.904460	2.676050	2.226315	3.337539
22	H	6.315114	5.804245	2.674854	2.223180	1.078441
23	C	5.640260	5.888497	3.333882	2.291440	1.420032
24	H	5.884299	6.050159	4.343839	3.334187	2.234292
25	C	5.538349	6.262969	3.330118	2.287426	2.291060
26	H	5.674418	6.703440	4.340421	3.331095	3.334212
27	C	5.739212	6.381789	2.219459	1.411479	2.295007
28	C	2.987947	4.287471	4.244877	3.579506	3.814436
29	O	2.870236	4.666146	5.254064	4.627875	4.931907
30	C	4.562433	3.272151	3.932822	3.874578	3.411578
31	O	5.686123	4.249254	4.144600	4.197691	3.595130
32	C	3.833799	3.862768	2.746500	2.721614	3.207278
33	O	4.622911	4.693087	2.692681	3.049873	3.895968
		21	22	23	24	25
21	H	0.000000				
22	H	4.342615	0.000000			
23	C	3.344739	2.235186	0.000000		
24	H	4.352583	2.698430	1.077759	0.000000	
25	C	2.240262	3.335060	1.415633	2.225510	0.000000
26	H	2.699360	4.345447	2.226266	2.678188	1.079470
27	C	1.078921	3.334733	2.301764	3.342904	1.425774
28	C	3.175771	4.636289	3.376534	3.951202	2.769720
29	O	3.807473	5.773834	4.376746	4.862137	3.606514
30	C	5.564681	3.046974	4.213646	4.584517	4.980379
31	O	6.155674	2.907149	4.581024	4.884584	5.528270
32	C	3.487189	3.629601	3.812743	4.620691	3.766561
33	O	3.542277	4.306390	4.655502	5.566289	4.437888
		26	27	28	29	30
26	H	0.000000				
27	C	2.237826	0.000000			
28	C	2.902982	2.926774	0.000000		
29	O	3.441963	3.798232	1.143503	0.000000	
30	C	5.850000	4.808592	4.406290	5.386621	0.000000
31	O	6.471022	5.338188	5.398656	6.431321	1.145425
32	C	4.535549	3.125841	2.638628	3.542665	2.630915
33	O	5.208772	3.466510	3.425465	4.170521	3.411774
		31	32	33		
31	O	0.000000				
32	C	3.530931	0.000000			
33	O	4.152353	1.169217	0.000000		

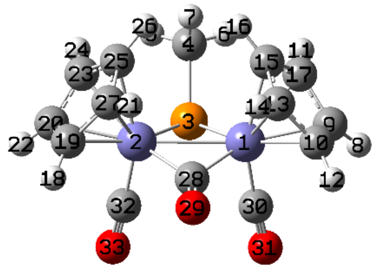

3S -3634.464104 4.82 Cs
WBI 0.31

Charge and spin density

		1
1	Fe	-0.003843
2	Fe	-0.004200
3	P	-0.062236
4	C	-0.086123
9	C	0.045303
10	C	-0.003021
13	C	0.044370
15	C	-0.065797
17	C	0.077727
19	C	-0.001301
20	C	0.045107
23	C	0.074372
25	C	-0.064383
27	C	0.043578
28	C	0.262667
29	O	-0.183349
30	C	0.027035
31	O	-0.086245
32	C	0.026684
33	O	-0.086343

		1	2	3	4	5
1	Fe	0.000000				
2	Fe	2.587862	0.000000			
3	P	2.264145	2.263856	0.000000		
4	C	3.382006	3.381401	1.862468	0.000000	
5	H	4.307689	3.743718	2.429187	1.093860	0.000000
6	H	3.750445	4.308815	2.428804	1.093878	1.746799
7	H	3.458113	3.461524	2.554243	1.091152	1.777278
8	H	2.813997	5.357405	4.193631	4.866971	5.893572
9	C	2.069678	4.631452	3.746513	4.326758	5.395789
10	C	2.091789	4.440154	4.282267	5.037338	6.078961
11	H	2.868599	4.689016	3.203589	2.789866	3.836887
12	H	2.833857	5.027208	5.060576	6.014975	7.031203
13	C	2.119478	3.864856	4.114346	4.570016	5.566430
14	H	2.868315	4.019929	4.787486	5.261800	6.175729
15	C	2.126251	3.710104	3.444306	3.404494	4.428510
16	H	2.894203	3.761328	3.674473	3.193931	4.093937
17	C	2.104477	4.226229	3.181835	3.206867	4.300627
18	H	5.020148	2.834182	5.062949	6.016358	6.149772
19	C	4.435960	2.092276	4.284219	5.038477	5.157509
20	C	4.632714	2.069921	3.753028	4.335431	4.192098
21	H	4.011044	2.868524	4.780889	5.250060	5.714538
22	H	5.359633	2.814118	4.203906	4.881172	4.562285
23	C	4.230800	2.104208	3.184861	3.212787	3.069660
24	H	4.697161	2.868131	3.208575	2.802374	2.333676
25	C	3.711428	2.126252	3.440404	3.397904	3.645682
26	H	3.764148	2.894075	3.666797	3.180040	3.568168
27	C	3.859889	2.119752	4.110684	4.563126	4.884231
28	C	1.904271	1.904677	3.188030	4.508898	5.189032
29	O	2.874314	2.874276	4.342824	5.666862	6.339547
30	C	1.753244	3.348099	2.731302	4.347078	5.121464
31	O	2.893050	4.215696	3.533418	5.219565	5.902709
32	C	3.348063	1.753223	2.730722	4.346216	4.550831
33	O	4.215433	2.893058	3.532802	5.218719	5.335176

		6	7	8	9	10
6	H	0.000000				
7	H	1.777314	0.000000			
8	H	4.552148	5.050130	0.000000		
9	C	4.190177	4.329977	1.079611	0.000000	
10	C	5.162757	4.863964	2.236623	1.425159	0.000000
11	H	2.334103	2.766879	2.679343	2.226776	3.344604
12	H	6.153110	5.891691	2.697776	2.239745	1.078901
13	C	4.899144	4.135470	3.330165	2.286590	1.411467
14	H	5.733243	4.722859	4.339497	3.329483	2.219372
15	C	3.663748	2.872825	3.330134	2.287032	2.294688
16	H	3.594452	2.371181	4.338653	3.328954	3.334565

17	C	3.075277	3.049025	2.226477	1.416139	2.303377
18	H	7.033506	5.896123	7.788759	7.006090	6.363420
19	C	6.081254	4.869518	7.248651	6.425197	5.919860
20	C	5.403593	4.345735	7.380890	6.635653	6.429822
21	H	6.168159	4.711806	6.580130	5.592674	4.794280
22	H	5.905509	5.070914	8.035548	7.382127	7.252541
23	C	4.306350	3.066047	6.835227	6.042540	6.011527
24	H	3.846897	2.793903	7.072999	6.354038	6.544960
25	C	4.425821	2.873429	6.307651	5.379619	5.145289
26	H	4.086201	2.362276	6.062579	5.089928	4.941225
27	C	5.562901	4.131997	6.589713	5.650347	5.084640
28	C	5.193576	4.481207	4.476978	3.743944	3.095645
29	O	6.344620	5.576084	5.094427	4.398933	3.441164
30	C	4.554938	4.777988	2.852941	2.733541	2.932888
31	O	5.337812	5.776003	3.354473	3.546980	3.794510
32	C	5.119693	4.780760	5.778142	5.322290	5.218949
33	O	5.899641	5.778649	6.358155	6.056270	5.994282
		11	12	13	14	15
11	H	0.000000				
12	H	4.354045	0.000000			
13	C	3.336207	2.226305	0.000000		
14	H	4.346456	2.675644	1.079314	0.000000	
15	C	2.231125	3.337693	1.416697	2.229535	0.000000
16	H	2.688397	4.343700	2.227004	2.684064	1.077457
17	C	1.077783	3.346164	2.293224	3.336209	1.416301
18	H	7.368986	6.648635	5.629140	5.234592	5.900437
19	C	6.531591	6.368113	5.082044	4.795095	5.135330
20	C	6.348357	7.013053	5.656194	5.602303	5.380604
21	H	5.916457	5.243253	3.664610	3.051330	3.972775
22	H	7.072194	7.793627	6.595145	6.587840	6.311038
23	C	5.370553	6.770573	5.125803	5.205748	4.540930
24	H	5.331668	7.383080	5.720456	5.942646	4.910589
25	C	4.894130	5.915117	4.056961	3.996020	3.573940
26	H	4.364684	5.816643	3.746614	3.761071	3.026183
27	C	5.700317	5.638589	4.028490	3.672176	4.043500
28	C	4.684752	3.398613	2.784837	2.832200	3.326304
29	O	5.654072	3.412299	3.212356	2.945075	4.105553
30	C	3.894322	3.193381	3.587731	4.272187	3.800324
31	O	4.776100	3.821419	4.633152	5.286890	4.907758
32	C	5.646028	5.580811	5.055828	5.281078	5.066751
33	O	6.490150	6.229598	6.010708	6.252014	6.087836
		16	17	18	19	20
16	H	0.000000				
17	C	2.227045	0.000000			
18	H	5.797616	6.758285	0.000000		
19	C	4.927158	6.001456	1.078896	0.000000	

20	C	5.089990	6.040282	2.239606	1.424953	0.000000
21	H	3.727895	5.184795	2.675803	2.219480	3.329277
22	H	6.066270	6.836227	2.697552	2.236373	1.079622
23	C	4.075511	5.198150	3.346259	2.303466	1.416234
24	H	4.385251	5.376802	4.354101	3.344664	2.226874
25	C	3.022416	4.532930	3.337737	2.294757	2.286972
26	H	2.234407	4.066718	4.343738	3.334625	3.328867
27	C	3.726086	5.111242	2.226394	1.411546	2.286364
28	C	3.800742	3.872584	3.389063	3.089971	3.740457
29	O	4.605752	4.744144	3.397689	3.432785	4.391974
30	C	4.623674	3.339405	5.577109	5.217039	5.323108
31	O	5.753207	4.318148	6.228002	5.993750	6.057092
32	C	5.328110	5.241044	3.201189	2.937857	2.732626
33	O	6.418717	6.123782	3.832275	3.800885	3.545929
		21	22	23	24	25
21	H	0.000000				
22	H	4.339311	0.000000			
23	C	3.336119	2.226522	0.000000		
24	H	4.346415	2.679371	1.077797	0.000000	
25	C	2.229338	3.330082	1.416330	2.231271	0.000000
26	H	2.683868	4.338574	2.226988	2.688494	1.077427
27	C	1.079321	3.329955	2.293162	3.336195	1.416582
28	C	2.832231	4.471818	3.874150	4.687623	3.330555
29	O	2.949315	5.084148	4.744259	5.655278	4.110900
30	C	5.277267	5.779468	5.243137	5.649769	5.067425
31	O	6.250478	6.359227	6.124932	6.491966	6.088403
32	C	4.278107	2.850471	3.335138	3.886911	3.799548
33	O	5.294846	3.351172	4.312923	4.766418	4.906877
		26	27	28	29	30
26	H	0.000000				
27	C	2.226926	0.000000			
28	C	3.808317	2.784553	0.000000		
29	O	4.616090	3.213489	1.169875	0.000000	
30	C	5.329358	5.053774	2.557796	3.260784	0.000000
31	O	6.419484	6.010064	3.446692	3.949239	1.140951
32	C	4.622082	3.591715	2.558347	3.261029	3.143838
33	O	5.751212	4.638311	3.446991	3.949292	3.566717
		31	32	33		
31	O	0.000000				
32	C	3.567081	0.000000			
33	O	3.632814	1.140968	0.000000		

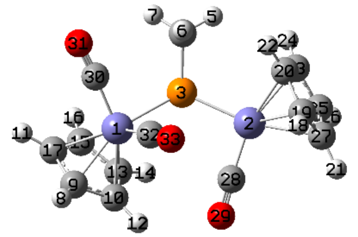

4T -3634.461201 6.65 C1
WBI 0.03

Charge and spin density

		1	2
1	Fe	0.082436	0.076529
2	Fe	0.310780	2.175958
3	P	-0.115456	-0.060170
4	C	-0.053191	0.003569
9	C	0.042259	-0.005877
10	C	0.020636	-0.004317
13	C	0.068078	0.008519
15	C	0.063331	0.002264
17	C	-0.017317	0.003277
19	C	-0.044966	0.038132
20	C	0.031434	-0.037374
23	C	-0.041920	-0.002342
25	C	-0.031667	0.007692
27	C	-0.002980	-0.026401
28	C	-0.010925	-0.134225
29	O	-0.126675	-0.052623
30	C	-0.015199	0.003296
31	O	-0.090778	0.000902
32	C	0.033824	-0.001173
33	O	-0.101704	0.004363

		1	2	3	4	5
1	Fe	0.000000				
2	Fe	3.941693	0.000000			
3	P	2.336426	2.230307	0.000000		
4	C	3.414950	3.321927	1.860115	0.000000	
5	H	4.387385	3.335890	2.438701	1.091440	0.000000
6	H	3.587217	3.630773	2.510556	1.093128	1.770749
7	H	3.565071	4.286025	2.458485	1.092418	1.763249
8	H	2.850696	6.184401	5.104827	6.176957	7.181085
9	C	2.101536	5.538915	4.306378	5.514104	6.484694
10	C	2.108102	4.565461	3.702585	5.264398	6.090445
11	H	2.844629	6.734946	4.893340	5.586036	6.607828
12	H	2.864332	4.434602	4.116136	5.755510	6.500356
13	C	2.119649	4.323492	3.118974	4.745014	5.479529
14	H	2.859656	3.919017	3.033632	4.792886	5.340574
15	C	2.113330	5.197980	3.459954	4.673651	5.515068
16	H	2.865221	5.596842	3.689948	4.683516	5.434766
17	C	2.099725	5.867940	4.176071	5.173582	6.156998
18	H	5.769563	2.955476	4.644755	4.951454	4.902168
19	C	5.600475	2.229666	4.097623	4.438430	4.229627
20	C	5.275140	2.145428	3.433958	3.327959	2.930513
21	H	6.601965	2.962834	5.190723	6.082200	5.850914
22	H	5.225798	2.880161	3.540381	2.906366	2.557317
23	C	5.629085	2.148706	3.451636	3.555600	2.902790
24	H	5.867861	2.876084	3.560041	3.367098	2.490169
25	C	6.102372	2.207647	4.095238	4.695558	4.184921
26	H	6.684956	2.940172	4.662055	5.397955	4.843012
27	C	6.052442	2.222003	4.417796	5.118279	4.818194
28	C	3.769293	1.770238	2.944755	4.577620	4.838184
29	O	4.135654	2.916034	3.825436	5.568333	5.907630
30	C	1.747629	4.871096	2.935032	2.970932	4.056045
31	O	2.888807	5.730816	3.775712	3.263313	4.293266
32	C	1.751528	3.517315	2.756485	3.542824	4.436450
33	O	2.894550	3.725879	3.533388	4.080436	4.854615

		6	7	8	9	10
6	H	0.000000				
7	H	1.769652	0.000000			
8	H	6.110180	6.274799	0.000000		
9	C	5.617265	5.579196	1.079045	0.000000	
10	C	5.510719	5.517063	2.239041	1.425114	0.000000
11	H	5.768624	5.216534	2.678904	2.222342	3.333251
12	H	5.930339	6.175188	2.700268	2.238401	1.078450
13	C	5.255395	4.869067	3.339733	2.296586	1.413392
14	H	5.441531	5.009356	4.348903	3.338976	2.224351
15	C	5.193770	4.483457	3.344602	2.301302	2.293962
16	H	5.352214	4.299793	4.353286	3.343302	3.336169

17	C	5.426572	4.974294	2.225846	1.411505	2.288938
18	H	4.588297	6.031363	7.417966	7.157416	6.426182
19	C	4.286688	5.529409	7.559961	7.148575	6.313923
20	C	3.262528	4.399956	7.616025	7.101648	6.380319
21	H	6.179576	7.119523	8.252797	7.791506	6.658666
22	H	2.577202	3.932761	7.596230	7.139417	6.618722
23	C	3.902181	4.511595	8.142195	7.471607	6.597666
24	H	3.901398	4.154876	8.550468	7.805493	6.995721
25	C	5.058418	5.653555	8.368520	7.703005	6.634473
26	H	5.903979	6.263297	8.930836	8.195596	7.032809
27	C	5.228519	6.165867	7.989217	7.475612	6.424835
28	C	4.907321	5.375443	5.389593	4.752211	3.511277
29	O	5.898573	6.279974	5.194941	4.603255	3.242461
30	C	2.947868	2.727659	3.769715	3.272355	3.766011
31	O	3.104123	2.757203	4.601237	4.227675	4.870476
32	C	3.259509	4.070771	3.249901	2.995571	2.909167
33	O	3.555105	4.752269	3.905862	3.888700	3.788216
		11	12	13	14	15
11	H	0.000000				
12	H	4.343708	0.000000			
13	C	3.332164	2.223167	0.000000		
14	H	4.340994	2.675985	1.078583	0.000000	
15	C	2.234512	3.334544	1.414391	2.222992	0.000000
16	H	2.694691	4.342486	2.228239	2.679763	1.078537
17	C	1.079965	3.332214	2.288775	3.331256	1.424234
18	H	8.557817	6.024608	6.705627	6.547782	7.548862
19	C	8.439562	5.995721	6.368709	6.075235	7.212185
20	C	8.046725	6.298282	6.202306	5.919950	6.825385
21	H	9.379564	6.095833	6.704044	6.167551	7.839013
22	H	7.876784	6.650500	6.470505	6.334994	6.901083
23	C	8.331563	6.563568	6.148852	5.648236	6.804760
24	H	8.402967	7.105258	6.360015	5.835505	6.848745
25	C	8.845034	6.409094	6.254842	5.613787	7.151526
26	H	9.323866	6.802386	6.524281	5.740931	7.463934
27	C	8.874326	6.018816	6.353076	5.847121	7.359381
28	C	6.347653	3.106206	3.474669	3.006617	4.693688
29	O	6.407255	2.602373	3.386668	2.920357	4.756337
30	C	2.867320	4.564588	3.634209	4.335532	3.017909
31	O	3.356628	5.683604	4.705019	5.391412	3.910816
32	C	4.205433	3.104752	3.460395	4.011729	3.823868
33	O	5.204669	3.721617	4.493902	4.967469	4.954373
		16	17	18	19	20
16	H	0.000000				
17	C	2.238799	0.000000			
18	H	8.118696	7.800465	0.000000		
19	C	7.673343	7.650841	1.079763	0.000000	

20	C	7.121592	7.349089	2.227913	1.413114	0.000000
21	H	8.338522	8.436599	2.676014	2.222540	3.324621
22	H	7.166249	7.299909	2.688259	2.227397	1.078945
23	C	6.948662	7.581529	3.345153	2.299996	1.430337
24	H	6.825139	7.721318	4.353830	3.341672	2.240865
25	C	7.385428	7.978696	3.341251	2.298605	2.294958
26	H	7.622454	8.422613	4.346991	3.339331	3.341613
27	C	7.783040	7.984675	2.222940	1.410517	2.281049
28	C	5.292906	5.338804	3.868217	3.446142	3.828897
29	O	5.451389	5.346776	4.715622	4.423324	4.946671
30	C	3.340330	2.752421	6.398948	6.248312	5.619588
31	O	4.032199	3.561739	7.061874	6.929127	6.164142
32	C	4.608089	3.570292	4.453666	4.556370	4.480918
33	O	5.750560	4.624169	3.824167	4.178955	4.320567
		21	22	23	24	25
21	H	0.000000				
22	H	4.335487	0.000000			
23	C	3.327990	2.239213	0.000000		
24	H	4.340196	2.693886	1.079449	0.000000	
25	C	2.230226	3.335686	1.408868	2.224470	0.000000
26	H	2.681018	4.349743	2.226618	2.688643	1.079604
27	C	1.080077	3.324978	2.285934	3.331434	1.421018
28	C	3.249490	4.550288	3.793423	4.484137	3.354606
29	O	3.841175	5.667784	4.894210	5.571286	4.301948
30	C	7.669633	5.241606	6.084703	6.131020	6.889406
31	O	8.530353	5.614993	6.672638	6.609654	7.627243
32	C	5.779807	4.374221	5.230146	5.720321	5.673865
33	O	5.517488	4.172794	5.314930	5.946586	5.713464
		26	27	28	29	30
26	H	0.000000				
27	C	2.231087	0.000000			
28	C	3.731188	3.076007	0.000000		
29	O	4.516025	3.937905	1.146610	0.000000	
30	C	7.562216	6.950253	5.205514	5.758434	0.000000
31	O	8.331639	7.741042	6.255399	6.867869	1.142282
32	C	6.456103	5.271394	3.531032	4.026348	2.544527
33	O	6.596653	5.069265	3.852376	4.369876	3.463854
		31	32	33		
31	O	0.000000				
32	C	3.477646	0.000000			
33	O	4.220945	1.143434	0.000000		

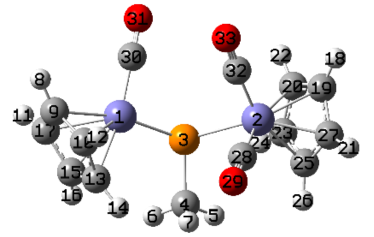

5T -3634.461201 7.77 C1
WBI 0.03

Charge and spin density

		1	2
1	Fe	0.282571	2.124559
2	Fe	0.114735	0.117362
3	P	-0.132128	-0.077361
4	C	-0.053199	0.003156
9	C	-0.002941	-0.030795
10	C	-0.049667	0.054788
13	C	0.035252	-0.040967
15	C	-0.054690	0.002291
17	C	-0.034800	-0.008614
19	C	-0.007350	0.002859
20	C	0.034094	-0.000238
23	C	0.092480	0.012465
25	C	0.019188	-0.004471
27	C	0.029992	-0.004377
28	C	0.037652	-0.004312
29	O	-0.105487	0.005832
30	C	0.003409	-0.110384
31	O	-0.090362	-0.039951
32	C	-0.035209	-0.000423
33	O	-0.083543	-0.001420

		1	2	3	4	5
1	Fe	0.000000				
2	Fe	3.976395	0.000000			
3	P	2.223470	2.338343	0.000000		
4	C	3.349362	3.331265	1.863557	0.000000	
5	H	4.312958	3.392585	2.456498	1.093640	0.000000
6	H	3.427392	4.318955	2.442117	1.092268	1.761956
7	H	3.622240	3.552467	2.522524	1.092667	1.769545
8	H	2.952943	6.609170	5.170792	6.118994	7.159158
9	C	2.210127	6.022801	4.401773	5.151293	6.212743
10	C	2.232235	5.458919	4.076682	4.538141	5.618354
11	H	2.922658	6.778689	4.678172	5.318528	6.257598
12	H	2.961266	5.568610	4.609734	5.095670	6.135878
13	C	2.145109	5.104575	3.425019	3.403452	4.494159
14	H	2.878049	4.955151	3.520415	3.026024	4.056404
15	C	2.141468	5.577745	3.469029	3.522590	4.557688
16	H	2.870313	5.833452	3.596430	3.268862	4.183835
17	C	2.185314	6.118841	4.095787	4.654246	5.670847
18	H	6.420832	2.843098	5.021267	6.104372	5.935029
19	C	5.904899	2.096363	4.247061	5.180223	4.921400
20	C	5.379290	2.113137	3.577102	4.647441	4.349224
21	H	6.666382	2.842299	4.992136	5.244584	4.898955
22	H	5.480104	2.866834	3.912419	5.229732	5.017532
23	C	5.203871	2.123247	3.077948	3.643563	3.124048
24	H	5.114039	2.860790	2.966219	3.374155	2.775441
25	C	5.622513	2.102890	3.534918	3.632735	3.044742
26	H	5.936120	2.860816	3.849436	3.392291	2.636160
27	C	6.047275	2.096384	4.229920	4.659271	4.280343
28	C	3.852366	1.747962	2.734480	3.069772	3.400923
29	O	4.212664	2.891534	3.495802	3.424345	3.866677
30	C	1.785960	4.049132	2.899123	4.579867	5.332107
31	O	2.926818	4.519251	3.743414	5.531058	6.173021
32	C	3.659279	1.754045	2.990509	4.474385	4.839557
33	O	3.972764	2.893049	3.864203	5.441394	5.903971

		6	7	8	9	10
6	H	0.000000				
7	H	1.771329	0.000000			
8	H	5.992394	6.159167	0.000000		
9	C	4.975756	5.172375	1.079992	0.000000	
10	C	4.522721	4.291496	2.220256	1.407829	0.000000
11	H	4.779919	5.701851	2.685470	2.234729	3.339108
12	H	5.253224	4.684936	2.674118	2.220785	1.079982
13	C	3.245289	3.155874	3.324220	2.280607	1.413654
14	H	2.992810	2.474671	4.334265	3.324075	2.227457
15	C	3.001898	3.671361	3.329886	2.287835	2.299800
16	H	2.464430	3.568139	4.342931	3.333799	3.340830

17	C	4.221052	4.885226	2.234196	1.425107	2.298927
18	H	7.087819	6.318390	8.673938	8.328285	7.875325
19	C	6.164508	5.453422	8.399104	7.937016	7.454529
20	C	5.492300	5.191793	8.015586	7.521446	7.229295
21	H	6.329017	5.123837	9.152755	8.562508	7.740079
22	H	5.950527	5.885356	7.960668	7.565803	7.468788
23	C	4.477064	4.250041	8.063889	7.405506	7.015442
24	H	3.999774	4.192383	8.018308	7.311250	7.038483
25	C	4.625756	3.898417	8.452741	7.736792	7.096143
26	H	4.345704	3.528643	8.787620	7.978821	7.235754
27	C	5.714483	4.749188	8.663141	8.067920	7.380347
28	C	4.082582	2.725885	6.240627	5.561486	4.639359
29	O	4.338022	2.722600	6.287319	5.575042	4.437020
30	C	4.793170	5.022303	3.300397	3.112000	3.563425
31	O	5.779862	6.036229	3.945731	4.004351	4.587543
32	C	5.312297	4.630492	5.733214	5.404791	5.005758
33	O	6.199085	5.559138	5.484642	5.370493	5.102204
		11	12	13	14	15
11	H	0.000000				
12	H	4.347726	0.000000			
13	C	3.341432	2.227199	0.000000		
14	H	4.350325	2.685754	1.079106	0.000000	
15	C	2.227108	3.344542	1.429637	2.239516	0.000000
16	H	2.690629	4.352148	2.238980	2.692744	1.079217
17	C	1.079414	3.342291	2.294595	3.335922	1.408521
18	H	9.156473	7.793096	7.787300	7.688717	8.274020
19	C	8.625651	7.477508	7.191864	7.037921	7.615926
20	C	7.858471	7.453305	6.859639	6.823098	7.020590
21	H	9.515443	7.604461	7.345740	6.882123	8.055536
22	H	7.743395	7.750112	7.198566	7.312965	7.210602
23	C	7.595006	7.340486	6.383785	6.212495	6.493827
24	H	7.193459	7.511596	6.281939	6.166081	6.170925
25	C	8.209909	7.277396	6.422265	6.033944	6.793386
26	H	8.416657	7.439740	6.401433	5.866065	6.797589
27	C	8.828741	7.375191	6.943927	6.584669	7.492621
28	C	6.703039	4.561290	4.257886	3.851490	5.115043
29	O	6.926447	4.204762	4.060828	3.459602	5.150466
30	C	3.591263	4.048286	3.884083	4.618879	3.746828
31	O	4.323881	4.980149	5.014696	5.754016	4.823501
32	C	6.450334	4.961401	5.118173	5.237266	5.650657
33	O	6.579400	4.937888	5.510916	5.768973	6.061301
		16	17	18	19	20
16	H	0.000000				
17	C	2.224703	0.000000			
18	H	8.589219	8.594481	0.000000		
19	C	7.829257	8.061463	1.079618	0.000000	

20	C	7.120505	7.429214	2.238960	1.428211	0.000000
21	H	8.239856	8.767171	2.678040	2.223228	3.342811
22	H	7.330976	7.441129	2.699017	2.241852	1.078773
23	C	6.419343	7.134269	3.334717	2.291980	1.411270
24	H	5.946676	6.837461	4.342464	3.333767	2.218424
25	C	6.752166	7.593944	3.336285	2.291953	2.292884
26	H	6.648926	7.761339	4.344921	3.334008	3.335455
27	C	7.630141	8.159685	2.221848	1.409400	2.298704
28	C	5.471103	5.837940	4.076048	3.486862	3.825247
29	O	5.554062	5.962100	5.039575	4.522436	4.955513
30	C	4.397414	3.274226	5.857258	5.546866	4.942045
31	O	5.434718	4.202177	5.809432	5.650194	5.013768
32	C	6.179660	5.814515	2.955017	2.807772	2.964993
33	O	6.725911	5.984127	3.463585	3.622350	3.845419
		21	22	23	24	25
21	H	0.000000				
22	H	4.351777	0.000000			
23	C	3.339364	2.224742	0.000000		
24	H	4.349615	2.672815	1.078702	0.000000	
25	C	2.238618	3.335202	1.414920	2.227123	0.000000
26	H	2.695260	4.344070	2.229198	2.686601	1.079019
27	C	1.079316	3.340832	2.296291	3.339555	1.426236
28	C	3.128835	4.613642	3.542575	4.143289	2.955695
29	O	3.747356	5.757214	4.591581	5.133617	3.843593
30	C	6.821404	4.727235	5.203038	5.198127	5.900747
31	O	7.198191	4.578358	5.531614	5.565535	6.366804
32	C	3.917226	3.235150	3.585505	4.240231	3.794708
33	O	4.783718	3.895424	4.645623	5.274210	4.903844
		26	27	28	29	30
26	H	0.000000				
27	C	2.237367	0.000000			
28	C	3.192420	2.927309	0.000000		
29	O	3.834706	3.805161	1.143823	0.000000	
30	C	6.493508	6.100010	4.507758	5.141762	0.000000
31	O	7.088879	6.437394	5.249315	5.981655	1.141930
32	C	4.600799	3.371284	2.522089	3.445099	3.187298
33	O	5.728074	4.341963	3.440090	4.181968	3.158952
		31	32	33		
31	O	0.000000				
32	C	3.432492	0.000000			
33	O	3.160713	1.141338	0.000000		

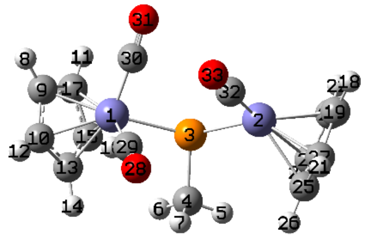

6T -3634.45838 8.42 C1
WBI 0.03

Charge and spin density

		1	2
1	Fe	0.104929	0.045815
2	Fe	0.301893	2.077223
3	P	-0.062574	0.052491
4	C	-0.049997	0.007734
9	C	-0.012867	0.001324
10	C	0.038695	-0.001347
13	C	0.031436	-0.001753
15	C	0.052854	0.008451
17	C	0.036928	-0.000703
19	C	-0.018607	-0.040500
20	C	-0.062053	0.058515
23	C	0.021926	-0.028844
25	C	-0.021233	0.014393
27	C	-0.037817	-0.009548
28	O	-0.091197	0.003156
29	C	0.008636	-0.005340
30	C	-0.025618	0.003851
31	O	-0.080743	-0.000524
32	C	-0.033111	-0.134204
33	O	-0.101477	-0.050189

		1	2	3	4	5
1	Fe	0.000000				
2	Fe	4.093016	0.000000			
3	P	2.322898	2.225664	0.000000		
4	C	3.310412	3.310661	1.860611	0.000000	
5	H	4.303969	3.320441	2.449801	1.091623	0.000000
6	H	3.377631	4.279133	2.467312	1.092536	1.765562
7	H	3.507822	3.612939	2.493314	1.092774	1.769162
8	H	2.849348	6.686138	5.047685	6.029365	7.033630
9	C	2.100644	6.112061	4.268159	5.071304	6.082272
10	C	2.100103	6.111245	4.210068	4.544286	5.619732
11	H	2.868154	5.825600	3.999787	5.073922	5.837640
12	H	2.843704	6.678529	4.949319	5.165476	6.256029
13	C	2.099963	5.612300	3.510007	3.435566	4.469803
14	H	2.858274	5.814802	3.799264	3.176840	4.167476
15	C	2.118560	5.307656	3.106211	3.408103	4.298488
16	H	2.860012	5.202540	3.018525	3.086521	3.776814
17	C	2.113081	5.625269	3.631999	4.483452	5.370608
18	H	6.788102	2.890919	5.083442	6.062691	5.791472
19	C	6.231258	2.157201	4.325944	5.113015	4.763158
20	C	6.048948	2.191891	3.901676	4.625332	4.124771
21	H	6.411083	2.930536	4.820830	5.118983	4.886523
22	H	6.422535	2.908488	4.342624	5.238899	4.751492
23	C	5.654053	2.189287	3.364793	3.551302	2.870877
24	H	5.737048	2.927995	3.422718	3.318835	2.466727
25	C	5.674262	2.221672	3.594601	3.512409	2.957575
26	H	5.744438	2.955140	3.811943	3.211319	2.630895
27	C	6.027584	2.196482	4.173720	4.539491	4.194865
28	O	2.893086	4.150426	3.520181	3.793954	4.658720
29	C	1.751734	3.844857	2.743850	3.321830	4.290126
30	C	1.755272	3.893426	2.895917	4.489742	5.301137
31	O	2.893384	4.238461	3.721155	5.453104	6.166019
32	C	3.786788	1.760830	2.932031	4.275498	4.637638
33	O	4.064458	2.904817	3.812141	5.148096	5.631321

		6	7	8	9	10
6	H	0.000000				
7	H	1.768877	0.000000			
8	H	5.807622	6.214868	0.000000		
9	C	4.771434	5.291881	1.079384	0.000000	
10	C	4.198585	4.531279	2.221281	1.407964	0.000000
11	H	4.727361	5.714463	2.701132	2.242901	3.338356
12	H	4.892876	4.937514	2.676375	2.220961	1.079465
13	C	2.891848	3.552226	3.337549	2.293466	1.425592
14	H	2.550783	3.093088	4.345360	3.334856	2.236725
15	C	2.817819	3.929108	3.336080	2.293687	2.295573
16	H	2.362487	3.833309	4.343707	3.335534	3.339904

17	C	4.092021	4.984359	2.241396	1.429978	2.296076
18	H	7.064928	6.273326	9.097761	8.705134	8.840014
19	C	6.122587	5.347675	8.726404	8.221953	8.256787
20	C	5.514656	5.120683	8.563167	7.968470	8.068423
21	H	6.211405	4.882313	9.013405	8.484429	8.187846
22	H	6.009869	5.878647	8.748954	8.197333	8.459467
23	C	4.441629	4.035662	8.358175	7.624605	7.521417
24	H	4.015725	3.994229	8.402198	7.589728	7.480146
25	C	4.548419	3.594098	8.473837	7.750514	7.446134
26	H	4.209718	3.064520	8.588764	7.797239	7.305218
27	C	5.617032	4.519322	8.692468	8.110759	7.903376
28	O	4.333836	3.158699	4.941103	4.450623	3.727574
29	C	3.733265	2.987214	4.007665	3.439128	2.883182
30	C	4.790242	4.761572	3.005470	2.822594	3.437359
31	O	5.820869	5.755922	3.536719	3.636558	4.439781
32	C	5.170978	4.303579	6.013049	5.670279	5.733493
33	O	5.983881	5.042662	5.890176	5.726778	5.818464
		11	12	13	14	15
11	H	0.000000				
12	H	4.349561	0.000000			
13	C	3.334957	2.236969	0.000000		
14	H	4.344013	2.693021	1.078779	0.000000	
15	C	2.222313	3.338707	1.417030	2.231997	0.000000
16	H	2.668809	4.350247	2.230625	2.692378	1.078991
17	C	1.079018	3.340458	2.292296	3.334894	1.409032
18	H	8.268059	9.334944	8.472233	8.689563	8.115844
19	C	7.786328	8.782223	7.753776	7.891209	7.412804
20	C	7.218482	8.733669	7.396901	7.547675	6.858216
21	H	8.614159	8.468855	7.745706	7.667617	7.811785
22	H	7.173023	9.205889	7.801896	8.052965	7.085924
23	C	7.012699	8.173355	6.671936	6.655548	6.242396
24	H	6.811288	8.208974	6.481380	6.424379	5.946210
25	C	7.545958	7.932238	6.672579	6.525627	6.540748
26	H	7.789061	7.717240	6.452390	6.137332	6.496494
27	C	7.994372	8.314757	7.349883	7.316813	7.247753
28	O	5.755126	3.621835	3.830852	3.842700	4.612261
29	C	4.614995	3.055223	2.963922	3.219684	3.569989
30	C	3.078561	4.037845	3.791172	4.611689	3.489802
31	O	3.620684	4.978585	4.897573	5.746353	4.495258
32	C	5.814802	6.118731	5.649328	5.991809	5.554691
33	O	6.155459	6.050918	6.001902	6.402680	6.045210
		16	17	18	19	20
16	H	0.000000				
17	C	2.215925	0.000000			
18	H	7.976961	8.253953	0.000000		
19	C	7.201470	7.704733	1.079572	0.000000	

20	C	6.470464	7.228079	2.228716	1.412985	0.000000
21	H	7.754407	8.260923	2.691155	2.239210	3.340704
22	H	6.640563	7.341266	2.689991	2.228280	1.080031
23	C	5.771490	6.868375	3.328890	2.284007	1.412896
24	H	5.315491	6.691971	4.337576	3.326142	2.222255
25	C	6.228095	7.224285	3.332247	2.291205	2.296506
26	H	6.188645	7.338655	4.344034	3.336487	3.338440
27	C	7.080512	7.720198	2.239213	1.430244	2.299520
28	O	5.198406	4.940837	6.444795	5.923238	6.291928
29	C	4.202906	3.820289	6.391324	5.844311	6.002343
30	C	4.108948	2.862046	6.106560	5.805141	5.770818
31	O	5.057907	3.677956	5.980090	5.873376	5.926473
32	C	5.783210	5.558263	3.336148	3.098809	3.668010
33	O	6.444233	5.871067	4.001435	3.990539	4.724648
		21	22	23	24	25
21	H	0.000000				
22	H	4.353030	0.000000			
23	C	3.333577	2.224827	0.000000		
24	H	4.343814	2.674380	1.079494	0.000000	
25	C	2.221880	3.339919	1.422340	2.236089	0.000000
26	H	2.680297	4.347520	2.233215	2.691665	1.079628
27	C	1.079337	3.345203	2.287905	3.330741	1.404464
28	O	5.081059	7.045688	5.794511	6.214355	5.101616
29	C	5.433680	6.626332	5.537726	5.842639	5.120277
30	C	6.290206	5.996028	5.797502	6.079482	5.922839
31	O	6.552808	6.029689	6.214581	6.588871	6.408578
32	C	3.295835	4.261414	3.915958	4.684834	3.620930
33	O	3.900802	5.274243	5.041572	5.827683	4.636506
		26	27	28	29	30
26	H	0.000000				
27	C	2.218644	0.000000			
28	O	4.902904	5.182130	0.000000		
29	C	5.040212	5.316967	1.142544	0.000000	
30	C	6.276597	5.921132	3.579639	2.618200	0.000000
31	O	6.906648	6.201988	4.410405	3.589738	1.140307
32	C	4.190994	3.088530	3.406300	3.228507	3.128300
33	O	5.131183	3.949481	3.360602	3.330557	3.172366
		31	32	33		
31	O	0.000000				
32	C	3.266622	0.000000			
33	O	3.118861	1.145542	0.000000		

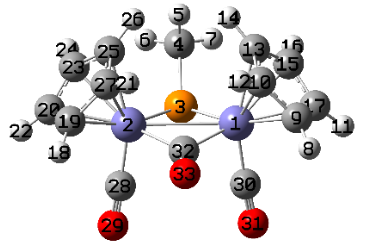

7T -3634.446048 16.15 Cs
WBI 0.15

Charge and spin density

		1	2
1	Fe	0.075334	0.715227
2	Fe	0.075599	0.714545
3	P	-0.107883	0.631362
4	C	-0.031409	0.022163
9	C	0.006423	0.002505
10	C	0.019878	-0.009659
13	C	-0.037266	-0.009439
15	C	0.027152	0.023449
17	C	0.007446	-0.016172
19	C	0.006037	0.002651
20	C	0.007543	-0.016056
23	C	0.028500	0.023260
25	C	-0.038284	-0.009122
27	C	0.019995	-0.009989
28	C	-0.001370	-0.030870
29	O	-0.088439	-0.028357
30	C	-0.001292	-0.030904
31	O	-0.088405	-0.028361
32	C	0.309742	0.021544
33	O	-0.189298	0.032223

		1	2	3	4	5
1	Fe	0.000000				
2	Fe	2.896655	0.000000			
3	P	2.184831	2.184923	0.000000		
4	C	3.478657	3.478826	1.843244	0.000000	
5	H	3.628538	3.624849	2.468653	1.092472	0.000000
6	H	4.394123	3.768238	2.439820	1.092216	1.773692
7	H	3.763775	4.393698	2.440031	1.092200	1.773782
8	H	2.866427	5.254065	5.050457	6.186795	6.115728
9	C	2.126306	4.705059	4.266411	5.220414	5.101013
10	C	2.142994	4.073419	3.949608	4.676872	4.312265
11	H	2.898083	5.778121	4.501583	5.230966	5.411384
12	H	2.879082	4.120709	4.525674	5.294301	4.828079
13	C	2.133766	3.977170	3.306251	3.547010	3.094459
14	H	2.891511	3.976938	3.444315	3.249350	2.507890
15	C	2.150301	4.612663	3.307139	3.527687	3.406295
16	H	2.892621	5.084457	3.412614	3.181418	3.161712
17	C	2.157297	5.026620	3.935719	4.639231	4.663649
18	H	5.255901	2.866431	5.050505	6.187029	6.111393
19	C	4.706168	2.126316	4.266527	5.220724	5.096027
20	C	5.026544	2.157357	3.934795	4.637712	4.654825
21	H	4.122515	2.879041	4.527686	5.297888	4.829492
22	H	5.777999	2.898189	4.499921	5.228099	5.400997
23	C	4.611520	2.150331	3.306625	3.526700	3.396481
24	H	5.082474	2.892698	3.411497	3.178802	3.148508
25	C	3.976661	2.133888	3.307529	3.549255	3.090417
26	H	3.975881	2.891671	3.446479	3.253581	2.507100
27	C	4.074470	2.143048	3.950973	4.679218	4.310434
28	C	3.578827	1.758292	2.854428	4.478847	4.958434
29	O	4.392350	2.899108	3.715300	5.354590	5.953568
30	C	1.758352	3.578667	2.854711	4.479181	4.961945
31	O	2.899148	4.392189	3.715379	5.354698	5.956683
32	C	1.949690	1.949565	2.908641	4.456389	4.516797
33	O	2.870174	2.870150	4.075079	5.619041	5.617909

		6	7	8	9	10
6	H	0.000000				
7	H	1.768831	0.000000			
8	H	7.181209	6.299722	0.000000		
9	C	6.246382	5.312227	1.079113	0.000000	
10	C	5.666635	4.973019	2.225516	1.413842	0.000000
11	H	6.254716	4.883782	2.696503	2.237026	3.338275
12	H	6.197536	5.747746	2.674667	2.222308	1.078953
13	C	4.581836	3.751333	3.330645	2.288235	1.413516
14	H	4.187069	3.596120	4.340118	3.331209	2.226882
15	C	4.618488	3.337252	3.333426	2.290876	2.295185
16	H	4.234504	2.690904	4.344149	3.334824	3.337525

17	C	5.699972	4.458607	2.239704	1.426077	2.295762
18	H	6.305135	7.180698	6.696838	6.474018	5.720451
19	C	5.318533	6.245814	6.472679	6.083430	5.216872
20	C	4.463139	5.699360	7.256545	6.731677	5.895242
21	H	5.757911	6.197114	5.219893	4.817149	3.690008
22	H	4.885557	6.254150	8.064154	7.581234	6.847385
23	C	3.344519	4.617778	7.040899	6.339721	5.391179
24	H	2.696987	4.233725	7.696723	6.913278	6.008899
25	C	3.762185	4.581278	6.091320	5.373321	4.253873
26	H	3.609562	4.186713	6.002123	5.169202	3.948958
27	C	4.982369	5.666127	5.717420	5.215809	4.144258
28	C	4.624228	5.226702	5.747359	5.419367	5.193080
29	O	5.422220	6.005322	6.353950	6.151500	6.112223
30	C	5.225354	4.623162	3.252301	2.994768	3.615305
31	O	6.002708	5.422153	3.900290	3.870565	4.667637
32	C	5.105078	5.102198	3.476582	3.170836	2.829415
33	O	6.260296	6.257089	3.405381	3.438456	3.209781
		11	12	13	14	15
11	H	0.000000				
12	H	4.346311	0.000000			
13	C	3.335132	2.228879	0.000000		
14	H	4.342509	2.688372	1.078690	0.000000	
15	C	2.219518	3.339969	1.424733	2.235293	0.000000
16	H	2.674577	4.349505	2.236080	2.691434	1.079156
17	C	1.079557	3.337916	2.290578	3.331442	1.405367
18	H	8.065169	5.223116	6.095793	6.008088	7.044476
19	C	7.581948	4.817598	5.376429	5.173730	6.342624
20	C	7.908803	5.700817	5.737252	5.422705	6.548106
21	H	6.693541	3.003017	4.082913	3.871484	5.333236
22	H	8.626084	6.692205	6.682888	6.406556	7.382668
23	C	7.382392	5.327751	4.936223	4.446465	5.747156
24	H	7.693174	6.082037	5.332259	4.768255	5.985372
25	C	6.683152	4.076537	3.888154	3.341995	4.938127
26	H	6.406731	3.861885	3.340631	2.572122	4.448283
27	C	6.848282	3.688179	4.257653	3.955044	5.395017
28	C	6.181507	5.320348	5.262298	5.487219	5.571020
29	O	6.728450	6.264689	6.247920	6.553027	6.412577
30	C	3.045812	4.270713	3.827251	4.634406	3.428498
31	O	3.584593	5.288369	4.941635	5.767941	4.417474
32	C	4.608492	2.846964	3.355309	3.810214	3.950792
33	O	5.135021	2.934160	4.102573	4.609561	4.763073
		16	17	18	19	20
16	H	0.000000				
17	C	2.218390	0.000000			
18	H	7.700754	7.258374	0.000000		
19	C	6.917047	6.732813	1.079121	0.000000	

20	C	6.918181	7.115956	2.239725	1.426085	0.000000
21	H	6.089049	5.702856	2.674510	2.222225	3.337906
22	H	7.693920	7.908690	2.696565	2.237056	1.079554
23	C	5.987003	6.547174	3.333416	2.290849	1.405384
24	H	6.032525	6.916348	4.344181	3.334823	2.218449
25	C	5.336447	5.737025	3.330623	2.288217	2.290621
26	H	4.773312	5.421988	4.340040	3.331159	3.331528
27	C	6.014286	5.896432	2.225469	1.413813	2.295775
28	C	6.004265	5.675635	3.250771	2.993829	2.888963
29	O	6.814440	6.368527	3.897786	3.869157	3.716908
30	C	3.975968	2.888625	5.747769	5.419523	5.675648
31	O	4.873568	3.716405	6.353934	6.151412	6.368643
32	C	4.747186	3.866488	3.478597	3.171924	3.867191
33	O	5.666814	4.437512	3.408328	3.439941	4.438915
		21	22	23	24	25
21	H	0.000000				
22	H	4.346292	0.000000			
23	C	3.339969	2.219539	0.000000		
24	H	4.349488	2.674672	1.079158	0.000000	
25	C	2.228916	3.335172	1.424740	2.236034	0.000000
26	H	2.688304	4.342609	2.235404	2.691514	1.078696
27	C	1.078955	3.338290	2.295162	3.337483	1.413505
28	C	4.269304	3.046593	3.429345	3.977526	3.827439
29	O	5.286218	3.585802	4.418788	4.876040	4.941912
30	C	5.320223	6.181709	5.570643	6.003787	5.261941
31	O	6.264046	6.728861	6.412490	6.814496	6.247640
32	C	2.846213	4.609766	3.950116	4.746266	3.353876
33	O	2.931839	5.137449	4.762644	5.666243	4.100658
		26	27	28	29	30
26	H	0.000000				
27	C	2.226791	0.000000			
28	C	4.634785	3.614467	0.000000		
29	O	5.768502	4.666382	1.141599	0.000000	
30	C	5.486688	5.193015	3.354121	3.729569	0.000000
31	O	6.552648	6.111881	3.729574	3.758503	1.141582
32	C	3.807890	2.829004	2.551195	3.425119	2.550812
33	O	4.606333	3.208675	3.187328	3.856635	3.186668
		31	32	33		
31	O	0.000000				
32	C	3.424923	0.000000			
33	O	3.856240	1.173257	0.000000		

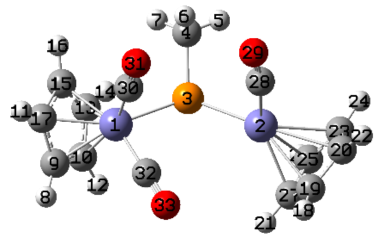

8Q -3634.443791 17.57 C1
WBI 0.02

Charge and spin density

		1	2
1	Fe	0.093773	0.100331
2	Fe	0.370707	3.426726
3	P	-0.129599	0.316571
4	C	-0.017600	0.017489
9	C	-0.001456	0.000504
10	C	0.039327	-0.001868
13	C	0.081860	0.009552
15	C	0.035835	-0.002606
17	C	0.016787	-0.003184
19	C	-0.057094	0.009904
20	C	-0.034066	0.114908
23	C	-0.063178	0.012965
25	C	-0.071751	0.032225
27	C	-0.088823	0.038148
28	C	0.019795	-0.020189
29	O	-0.041855	-0.042704
30	C	0.022722	-0.008151
31	O	-0.087763	0.000029
32	C	0.003698	0.002437
33	O	-0.091319	-0.003087

		1	2	3	4	5
1	Fe	0.000000				
2	Fe	4.107567	0.000000			
3	P	2.310119	2.336531	0.000000		
4	C	3.390862	3.464730	1.856262	0.000000	
5	H	4.359025	3.458628	2.434632	1.093020	0.000000
6	H	3.625713	3.769010	2.493915	1.093605	1.769877
7	H	3.490613	4.428927	2.470167	1.092735	1.765759
8	H	2.842781	6.406004	4.948038	6.172782	7.116057
9	C	2.095459	5.943554	4.201168	5.264313	6.220582
10	C	2.108370	5.380229	3.519773	4.655498	5.473947
11	H	2.843654	6.851205	5.020509	5.471701	6.554750
12	H	2.862849	5.387159	3.814872	5.165514	5.842007
13	C	2.117692	5.313613	3.095899	3.700197	4.539444
14	H	2.859406	5.231872	3.010798	3.375621	4.025162
15	C	2.101028	5.824990	3.599546	3.824947	4.830517
16	H	2.859251	6.210888	3.961951	3.661766	4.652967
17	C	2.095288	6.198967	4.246106	4.839172	5.887268
18	H	6.032842	3.073204	5.092708	6.450037	6.513664
19	C	5.834285	2.351639	4.530262	5.815639	5.747861
20	C	6.462111	2.457701	4.785455	5.673852	5.381249
21	H	5.156970	2.893891	4.148004	5.813416	5.874114
22	H	7.110876	3.195979	5.484311	6.165581	5.851570
23	C	6.388509	2.356005	4.398105	5.146824	4.662689
24	H	7.022652	3.082164	4.871478	5.264482	4.582510
25	C	5.741691	2.223133	3.886644	5.005127	4.664468
26	H	5.879009	2.897162	3.975095	5.013281	4.601032
27	C	5.357564	2.215644	3.975043	5.437085	5.360907
28	C	4.518175	1.969786	3.108381	3.431995	3.518693
29	O	4.947153	3.092464	3.806986	3.697043	3.845514
30	C	1.752310	4.131411	2.746201	3.171805	4.181725
31	O	2.894006	4.556124	3.517987	3.529133	4.457787
32	C	1.753367	3.574890	2.823601	4.398598	5.182436
33	O	2.895083	3.682122	3.615290	5.281696	5.953371

		6	7	8	9	10
6	H	0.000000				
7	H	1.772603	0.000000			
8	H	6.435984	6.075587	0.000000		
9	C	5.575735	5.072216	1.079651	0.000000	
10	C	5.226522	4.411619	2.238097	1.427171	0.000000
11	H	5.397103	5.191006	2.677681	2.223472	3.342516
12	H	5.855516	5.005211	2.698758	2.241315	1.078783
13	C	4.305798	3.220694	3.335593	2.292670	1.412942
14	H	4.180528	2.779127	4.343823	3.334789	2.220853
15	C	4.090603	3.299171	3.336698	2.292407	2.293115
16	H	3.783734	2.974933	4.345645	3.334742	3.335804

17	C	4.961532	4.526164	2.221496	1.409486	2.298479
18	H	6.568477	7.400811	7.459227	7.429964	7.167250
19	C	6.045607	6.770241	7.517579	7.350923	6.901075
20	C	5.844441	6.695911	8.497167	8.194234	7.667711
21	H	6.353291	6.519021	6.330810	6.216626	5.554911
22	H	6.167979	7.232628	9.216102	8.920373	8.493005
23	C	5.526707	6.090974	8.553944	8.121208	7.376674
24	H	5.637135	6.182641	9.346129	8.826852	8.020764
25	C	5.589978	5.815447	7.650260	7.250900	6.395873
26	H	5.767484	5.672378	7.726422	7.257910	6.232602
27	C	5.910084	6.260669	6.936440	6.716179	6.052568
28	C	3.132170	4.502455	7.044423	6.553131	6.304976
29	O	3.066673	4.709174	7.503673	6.990564	6.891263
30	C	2.845047	3.499348	4.106782	3.502179	3.821250
31	O	2.850373	3.942733	5.083338	4.541515	4.947676
32	C	4.583658	4.824841	2.999491	2.841568	2.979937
33	O	5.440721	5.823312	3.573058	3.698052	3.870062
		11	12	13	14	15
11	H	0.000000				
12	H	4.351694	0.000000			
13	C	3.339974	2.225758	0.000000		
14	H	4.350308	2.674947	1.078759	0.000000	
15	C	2.239744	3.335187	1.414285	2.226415	0.000000
16	H	2.697160	4.344040	2.228171	2.684789	1.079156
17	C	1.079316	3.340781	2.296981	3.340363	1.427271
18	H	8.483155	6.962458	7.599492	7.756591	8.078637
19	C	8.470861	6.668576	7.201265	7.217970	7.784201
20	C	9.138924	7.515634	7.731624	7.610514	8.270078
21	H	7.817446	5.041966	6.106138	6.107605	6.985980
22	H	9.673391	8.422822	8.500967	8.409840	8.912230
23	C	9.178675	7.170436	7.301812	6.995581	7.988433
24	H	9.786377	7.856768	7.783777	7.363512	8.453061
25	C	8.580733	6.043488	6.468454	6.160846	7.332449
26	H	8.707056	5.796216	6.243651	5.792764	7.250933
27	C	8.110894	5.671160	6.393502	6.314044	7.189880
28	C	6.830328	6.605361	6.025191	6.063633	6.093538
29	O	6.907181	7.330242	6.494051	6.582368	6.328375
30	C	3.138455	4.614593	3.512111	4.106149	2.923908
31	O	3.759341	5.756477	4.545942	5.073953	3.795111
32	C	3.943922	3.248972	3.586352	4.235729	3.801582
33	O	4.864514	3.908904	4.644222	5.250556	4.925439
		16	17	18	19	20
16	H	0.000000				
17	C	2.238776	0.000000			
18	H	8.657120	7.986404	0.000000		
19	C	8.317504	7.877078	1.080496	0.000000	

	20 C 8.650504 8.549483 2.223796 1.408682 0.000000<br>21 H 7.715041 7.050710 2.687891 2.234209 3.333901<br>22 H 9.216607 9.171027 2.678244 2.221191 1.080385<br>23 C 8.328716 8.472380 3.327215 2.282761 1.409675<br>24 H 8.681761 9.074414 4.337281 3.326662 2.223514<br>25 C 7.810343 7.779674 3.337075 2.293284 2.291492<br>26 H 7.727407 7.820622 4.347910 3.337518 3.334907<br>27 C 7.798179 7.375879 2.230932 1.420257 2.290960<br>28 C 6.228508 6.431997 3.837360 3.456832 3.160130<br>29 O 6.306449 6.658247 4.736818 4.480282 4.094173<br>30 C 3.144588 2.926629 6.043561 5.927824 6.335272<br>31 O 3.757923 3.801723 6.351738 6.290740 6.543119<br>32 C 4.601132 3.393884 4.651562 4.669213 5.574010<br>33 O 5.739050 4.404620 3.932903 4.157049 5.255490<br>21 22 23 24 25<br>21 H 0.000000<br>22 H 4.344106 0.000000<br>23 C 3.336702 2.222209 0.000000<br>24 H 4.347974 2.677532 1.080569 0.000000<br>25 C 2.233782 3.335074 1.419002 2.230505 0.000000<br>26 H 2.691575 4.345127 2.234384 2.690026 1.079721<br>27 C 1.080133 3.334697 2.291686 3.335987 1.419403<br>28 C 4.681448 3.266372 3.485123 3.887707 3.976652<br>29 O 5.792099 3.966463 4.476352 4.731614 5.084167<br>30 C 5.907253 6.740864 6.456914 7.001599 6.183424<br>31 O 6.630231 6.772317 6.779622 7.247821 6.732758<br>32 C 4.004564 6.235140 5.783024 6.617838 5.098684<br>33 O 3.535979 5.901443 5.669463 6.613359 4.988435<br>26 27 28 29 30<br>26 H 0.000000<br>27 C 2.232376 0.000000<br>28 C 4.705068 3.957574 0.000000<br>29 O 5.787377 5.082784 1.137182 0.000000<br>30 C 6.526885 5.839897 3.725660 3.779661 0.000000<br>31 O 7.174315 6.426321 3.627168 3.338864 1.142042<br>32 C 5.454348 4.326525 4.207755 4.824955 2.548839<br>33 O 5.478754 3.951284 4.410801 5.104127 3.479479<br>31 32 33<br>31 O 0.000000<br>32 C 3.484058 0.000000<br>33 O 4.244542 1.141888 0.000000

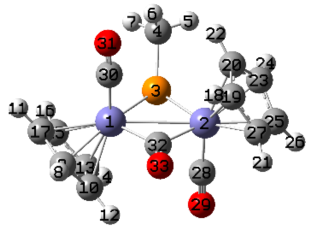

9T -3634.443496 17.76 C1
WBI 0.15

Charge and spin density

		1	2
1	Fe	0.049308	0.616269
2	Fe	0.076107	0.765673
3	P	-0.144112	0.659807
4	C	-0.012214	0.019419
9	C	0.033826	-0.020740
10	C	0.024538	0.007703
13	C	0.071569	0.001283
15	C	0.005175	0.040296
17	C	-0.013066	-0.008358
19	C	0.047119	0.009951
20	C	0.078033	-0.042751
23	C	-0.044181	0.039842
25	C	0.003843	-0.020741
27	C	0.018526	-0.013164
28	C	0.032679	-0.038820
29	O	-0.104485	-0.024791
30	C	-0.007707	-0.054023
31	O	-0.121115	-0.022826
32	C	0.189075	0.040015
33	O	-0.182918	0.045956

		1	2	3	4	5
1	Fe	0.000000				
2	Fe	2.902293	0.000000			
3	P	2.186473	2.175431	0.000000		
4	C	3.527006	3.495032	1.840205	0.000000	
5	H	4.418611	3.699455	2.449415	1.091811	0.000000
6	H	3.623656	3.755084	2.462988	1.092752	1.780352
7	H	3.885384	4.399658	2.424945	1.092653	1.771660
8	H	2.857038	5.108170	5.031274	6.295647	7.228925
9	C	2.121628	4.640675	4.248285	5.572662	6.512959
10	C	2.133561	4.059924	3.819508	5.443385	6.249067
11	H	2.900728	5.782238	4.654063	5.320127	6.397878
12	H	2.868848	4.058160	4.327383	6.073297	6.768944
13	C	2.139076	4.111241	3.210370	4.742626	5.536839
14	H	2.885132	4.168756	3.267196	4.840764	5.477454
15	C	2.162767	4.749926	3.368780	4.447888	5.401990
16	H	2.904216	5.280816	3.535209	4.256883	5.194500
17	C	2.163105	5.049824	4.023331	5.017133	6.046068
18	H	4.195870	2.875065	4.344398	4.946126	5.215865
19	C	4.159324	2.138452	3.821039	4.461249	4.564887
20	C	4.180583	2.126324	3.177573	3.366988	3.281493
21	H	5.153581	2.865989	5.032576	6.159235	6.197145
22	H	4.281746	2.888244	3.260680	2.905870	2.874753
23	C	4.769717	2.153137	3.319994	3.660811	3.258049
24	H	5.279507	2.893271	3.471141	3.476522	2.790471
25	C	5.056311	2.159059	3.987489	4.799867	4.520612
26	H	5.762587	2.899772	4.616263	5.514508	5.144309
27	C	4.687107	2.125904	4.238373	5.194517	5.170025
28	C	3.380918	1.762988	2.904626	4.587428	4.778256
29	O	4.110669	2.905898	3.774979	5.493104	5.665615
30	C	1.742014	3.477950	2.798054	3.268056	4.219063
31	O	2.888407	4.233652	3.623850	3.574306	4.464627
32	C	1.935369	1.978325	2.938373	4.390404	4.992805
33	O	2.874537	2.879281	4.109355	5.506792	6.091798

		6	7	8	9	10
6	H	0.000000				
7	H	1.767185	0.000000			
8	H	6.171969	6.567276	0.000000		
9	C	5.593500	5.758226	1.078837	0.000000	
10	C	5.708853	5.674771	2.231122	1.419251	0.000000
11	H	5.179463	5.177227	2.692821	2.232052	3.336280
12	H	6.363450	6.425126	2.684185	2.229280	1.079207
13	C	5.208143	4.774162	3.333512	2.291843	1.410060
14	H	5.498108	4.846994	4.343333	3.335328	2.223976
15	C	4.770050	4.258865	3.334656	2.291960	2.293123
16	H	4.664630	3.812145	4.344951	3.335400	3.334842

17	C	5.045581	4.961991	2.237371	1.421968	2.292980
18	H	4.515313	5.942941	5.389863	5.456271	5.517009
19	C	4.219910	5.511613	5.800485	5.684605	5.504374
20	C	3.179856	4.456949	6.368962	6.054797	5.842543
21	H	6.171737	7.156901	6.266887	6.232184	5.712790
22	H	2.442554	3.960362	6.532648	6.208424	6.184824
23	C	3.863814	4.699546	7.129695	6.688210	6.182760
24	H	3.855858	4.419551	7.845103	7.295782	6.738498
25	C	5.039134	5.806360	7.065684	6.710838	6.051710
26	H	5.896611	6.452768	7.732233	7.332971	6.505423
27	C	5.208475	6.223366	6.242310	6.084665	5.601702
28	C	5.118976	5.263806	5.230345	4.649131	3.596999
29	O	6.121474	6.047824	5.640282	5.028726	3.768535
30	C	2.826127	3.710320	3.468702	3.127634	3.702227
31	O	2.805271	4.011000	4.233701	4.070432	4.801642
32	C	4.383835	5.110747	3.243201	3.008530	2.756458
33	O	5.396861	6.243052	3.114501	3.249653	3.148074
		11	12	13	14	15
11	H	0.000000				
12	H	4.345781	0.000000			
13	C	3.335185	2.222996	0.000000		
14	H	4.343481	2.680313	1.079068	0.000000	
15	C	2.218985	3.337349	1.426229	2.237698	0.000000
16	H	2.675410	4.345664	2.236842	2.693273	1.078977
17	C	1.079881	3.336090	2.290671	3.332492	1.405970
18	H	6.412204	5.496542	6.059601	6.503837	6.356916
19	C	6.690858	5.452726	5.867462	6.142874	6.283474
20	C	6.715976	5.974013	5.872124	6.042475	6.140542
21	H	7.806260	5.238390	6.304502	6.411073	7.130690
22	H	6.506133	6.482543	6.115237	6.369013	6.134892
23	C	7.513491	6.199125	6.057002	5.972676	6.537813
24	H	7.946760	6.830428	6.398567	6.192193	6.819572
25	C	7.918324	5.823592	6.138559	6.007433	6.874536
26	H	8.660495	6.166740	6.541710	6.252387	7.420243
27	C	7.426072	5.309657	5.995134	6.084191	6.701098
28	C	6.167276	3.301219	3.602718	3.325469	4.685283
29	O	6.687997	3.287484	3.741218	3.237452	5.017940
30	C	3.022366	4.405526	3.790884	4.554908	3.326072
31	O	3.581316	5.489756	4.907513	5.673133	4.298884
32	C	4.482000	2.778130	3.390149	3.898500	3.950995
33	O	4.958040	2.900930	4.133325	4.703750	4.737089
		16	17	18	19	20
16	H	0.000000				
17	C	2.220247	0.000000			
18	H	7.002253	6.023739	0.000000		
19	C	6.858056	6.190875	1.078819	0.000000	

20	C	6.506456	6.261271	2.226086	1.413351	0.000000
21	H	7.863829	7.099105	2.678819	2.226154	3.331323
22	H	6.382915	6.206279	2.680789	2.224546	1.078755
23	C	6.843747	6.916382	3.340751	2.296908	1.427410
24	H	6.969024	7.363031	4.350322	3.339535	2.239485
25	C	7.343031	7.204609	3.337691	2.294646	2.289930
26	H	7.874171	7.869121	4.346663	3.337320	3.334683
27	C	7.328332	6.759628	2.225005	1.414497	2.289119
28	C	5.279952	5.225030	4.348902	3.670806	3.819564
29	O	5.613315	5.668352	5.397602	4.745730	4.938004
30	C	3.790671	2.887153	3.675075	3.901430	3.813153
31	O	4.630363	3.739614	3.781651	4.163572	4.004539
32	C	4.780375	3.764597	2.788095	2.785094	3.383938
33	O	5.667858	4.299677	2.807965	3.091406	4.062008
		21	22	23	24	25
21	H	0.000000				
22	H	4.339479	0.000000			
23	C	3.334570	2.239905	0.000000		
24	H	4.344849	2.699144	1.079222	0.000000	
25	C	2.239899	3.331672	1.404137	2.217522	0.000000
26	H	2.696042	4.343419	2.217499	2.672108	1.079807
27	C	1.079023	3.331183	2.292235	3.335910	1.425587
28	C	3.366707	4.616421	3.394100	3.899402	2.899574
29	O	4.070985	5.746306	4.380374	4.777077	3.743298
30	C	5.494173	3.536202	4.827497	5.362037	5.397676
31	O	6.011070	3.473062	5.206101	5.720985	5.913718
32	C	3.339162	3.878704	3.977750	4.798496	3.828937
33	O	3.171401	4.614998	4.715997	5.647415	4.323193
		26	27	28	29	30
26	H	0.000000				
27	C	2.236011	0.000000			
28	C	3.043279	3.071172	0.000000		
29	O	3.597836	3.979776	1.143215	0.000000	
30	C	6.311181	4.897270	4.606394	5.537029	0.000000
31	O	6.906116	5.369059	5.574071	6.566975	1.146574
32	C	4.560719	3.082044	2.615369	3.487679	2.637097
33	O	5.012469	3.265830	3.320806	4.028919	3.380170
		31	32	33		
31	O	0.000000				
32	C	3.532399	0.000000			
33	O	4.118991	1.171802	0.000000		

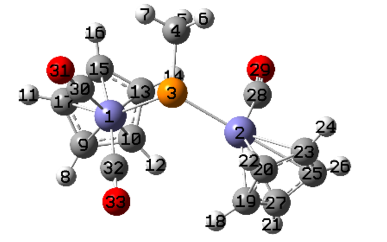

10Q -3634.442862 18.15 C1
WBI 0.03

Charge and spin density

		1	2
1	Fe	0.066291	0.115743
2	Fe	0.376355	3.367570
3	P	-0.084625	0.423400
4	C	-0.005786	0.016701
9	C	0.021606	-0.002705
10	C	0.003289	0.006636
13	C	0.063083	-0.003802
15	C	0.053491	-0.000005
17	C	0.020776	-0.003723
19	C	-0.076846	0.036565
20	C	-0.052753	0.032585
23	C	-0.078684	0.007363
25	C	-0.026110	0.116977
27	C	-0.083602	0.005305
28	C	0.015687	-0.048467
29	O	-0.084303	-0.054534
30	C	0.022095	0.000378
31	O	-0.085866	-0.002388
32	C	0.024278	-0.006397
33	O	-0.088375	-0.007202

		1	2	3	4	5
1	Fe	0.000000				
2	Fe	4.022162	0.000000			
3	P	2.305068	2.329430	0.000000		
4	C	3.368507	3.555541	1.849366	0.000000	
5	H	3.564967	3.887384	2.480376	1.094423	0.000000
6	H	4.345089	3.580155	2.434425	1.092483	1.770190
7	H	3.486907	4.492804	2.463088	1.092210	1.773508
8	H	2.847460	6.042629	5.003468	6.118791	6.064242
9	C	2.098066	5.438497	4.244097	5.180849	5.043771
10	C	2.109713	4.391057	3.606779	4.562712	4.298342
11	H	2.843772	6.809523	4.973215	5.353214	5.261534
12	H	2.869335	4.138228	3.954224	5.108773	4.861633
13	C	2.116801	4.271023	3.134461	3.539207	3.071501
14	H	2.873126	3.882201	3.087151	3.211432	2.538909
15	C	2.099048	5.261220	3.565328	3.642159	3.266597
16	H	2.858317	5.751000	3.886630	3.437003	2.976118
17	C	2.095840	5.896586	4.225862	4.714249	4.545279
18	H	4.869590	2.891695	4.088833	5.830452	6.365829
19	C	5.125710	2.211538	3.937483	5.510080	5.991793
20	C	5.607164	2.220833	3.899483	5.171498	5.777604
21	H	5.758094	3.070634	5.029898	6.476948	6.602009
22	H	5.781726	2.899613	4.011847	5.202232	5.971586
23	C	6.295944	2.354503	4.427419	5.367044	5.791007
24	H	6.987258	3.082517	4.928673	5.547228	5.978522
25	C	6.311854	2.451239	4.777606	5.828005	6.040191
26	H	6.978152	3.189716	5.478396	6.327042	6.382424
27	C	5.600926	2.344121	4.484868	5.887523	6.134664
28	C	4.413736	1.935970	3.005604	3.412876	3.179253
29	O	4.827183	3.065054	3.681808	3.626397	3.053015
30	C	1.755187	4.902126	2.717694	3.317382	3.847872
31	O	2.896056	5.713573	3.482896	3.754400	4.406892
32	C	1.758235	3.879543	2.819718	4.449635	4.890305
33	O	2.898207	4.209914	3.616570	5.362015	5.882998

		6	7	8	9	10
6	H	0.000000				
7	H	1.766273	0.000000			
8	H	7.114937	6.231634	0.000000		
9	C	6.200376	5.317186	1.079516	0.000000	
10	C	5.491120	4.976198	2.238925	1.427565	0.000000
11	H	6.437940	5.043285	2.677450	2.222383	3.340435
12	H	5.916557	5.678262	2.697635	2.240311	1.079063
13	C	4.502939	3.945281	3.337535	2.294794	1.412849
14	H	4.024994	3.839469	4.346958	3.337647	2.223140
15	C	4.717748	3.640676	3.337618	2.293391	2.292325
16	H	4.480683	3.235027	4.346674	3.335755	3.334892

17	C	5.798475	4.607169	2.221844	1.408995	2.296214
18	H	5.994430	6.481190	6.039704	5.927996	5.300436
19	C	5.528907	6.287914	6.559100	6.295865	5.460858
20	C	4.926720	5.943304	7.498783	7.103550	6.253561
21	H	6.619946	7.389613	6.511655	6.295658	5.224608
22	H	4.892063	5.825052	7.837572	7.453977	6.753700
23	C	4.965620	6.283198	8.214747	7.713983	6.664419
24	H	4.946513	6.448392	9.090499	8.518666	7.453115
25	C	5.605129	6.822861	7.848701	7.393486	6.227048
26	H	6.067485	7.375985	8.422457	7.924571	6.657548
27	C	5.900913	6.804777	6.803035	6.491994	5.433212
28	C	3.477220	4.486045	6.298837	5.499008	4.205755
29	O	3.738203	4.657881	6.584148	5.697784	4.372911
30	C	4.221852	2.918679	4.045939	3.460773	3.820753
31	O	4.527092	3.071133	4.998668	4.486155	4.944737
32	C	5.208305	4.653298	3.033988	2.859786	2.950779
33	O	6.000177	5.594837	3.612009	3.714421	3.828165
		11	12	13	14	15
11	H	0.000000				
12	H	4.349329	0.000000			
13	C	3.339666	2.226284	0.000000		
14	H	4.349056	2.679489	1.078396	0.000000	
15	C	2.238366	3.335134	1.415190	2.225773	0.000000
16	H	2.696535	4.344007	2.228385	2.682480	1.079128
17	C	1.079443	3.338406	2.296677	3.339388	1.426385
18	H	7.514233	4.823382	5.865586	5.927103	6.719335
19	C	7.889647	4.956771	5.834254	5.697707	6.799051
20	C	8.445198	5.906678	6.341306	6.071517	7.208636
21	H	8.263241	4.376449	5.785528	5.548789	7.030005
22	H	8.575983	6.538589	6.783403	6.586430	7.477776
23	C	9.120826	6.250261	6.600092	6.109755	7.603161
24	H	9.789314	7.112229	7.224722	6.640579	8.172304
25	C	9.050226	5.626922	6.312975	5.797043	7.491201
26	H	9.625017	6.007912	6.674893	6.036582	7.929085
27	C	8.285836	4.743668	5.803174	5.504114	6.973833
28	C	6.825575	3.998893	3.701260	2.893233	4.856619
29	O	6.898617	4.260233	3.636835	2.632451	4.768974
30	C	3.073960	4.622948	3.544406	4.178786	2.938274
31	O	3.668750	5.763504	4.585539	5.164080	3.812318
32	C	4.001745	3.203764	3.556985	4.205966	3.807476
33	O	4.936330	3.839933	4.603615	5.203512	4.929449
		16	17	18	19	20
16	H	0.000000				
17	C	2.238720	0.000000			
18	H	7.459053	6.757499	0.000000		
19	C	7.463813	7.054142	1.080573	0.000000	

20	C	7.701389	7.642561	2.232956	1.419402	0.000000
21	H	7.816956	7.297889	2.692407	2.232656	3.338003
22	H	7.896200	7.870693	2.689075	2.231869	1.079799
23	C	8.030870	8.232838	3.337134	2.292154	1.419690
24	H	8.481187	8.917395	4.348227	3.336519	2.231172
25	C	8.034900	8.084303	3.336162	2.292242	2.292198
26	H	8.441975	8.615530	4.346621	3.335964	3.335776
27	C	7.665454	7.343430	2.236565	1.420929	2.293600
28	C	5.213345	5.820841	4.653863	3.947684	3.977362
29	O	5.000841	5.890724	5.752899	5.073171	5.104294
30	C	3.178874	2.887449	5.649499	5.952772	6.133933
31	O	3.804184	3.749949	6.395536	6.711308	6.731968
32	C	4.612238	3.429822	3.610004	4.173433	4.868177
33	O	5.750491	4.444867	3.077574	3.881390	4.707155
		21	22	23	24	25
21	H	0.000000				
22	H	4.348756	0.000000			
23	C	3.326974	2.235645	0.000000		
24	H	4.336966	2.691625	1.080701	0.000000	
25	C	2.223780	3.335724	1.409109	2.222824	0.000000
26	H	2.677919	4.346033	2.221811	2.676790	1.080350
27	C	1.080799	3.337715	2.282659	3.326694	1.409486
28	C	3.858505	4.696164	3.525708	3.940570	3.214377
29	O	4.745880	5.799391	4.552002	4.836665	4.172097
30	C	7.062357	5.998651	6.964810	7.512072	7.302339
31	O	8.021884	6.429820	7.611655	8.065586	8.107089
32	C	5.050294	4.988132	5.844215	6.679166	5.876960
33	O	4.880180	4.782382	5.837881	6.739527	5.873608
		26	27	28	29	30
26	H	0.000000				
27	C	2.222006	0.000000			
28	C	3.346867	3.474998	0.000000		
29	O	4.082116	4.498820	1.143042	0.000000	
30	C	8.063705	6.710784	5.505682	5.941210	0.000000
31	O	8.902629	7.585647	6.383795	6.813315	1.141250
32	C	6.707943	4.907627	4.923438	5.640878	2.564758
33	O	6.780728	4.764567	5.539827	6.378448	3.497482
		31	32	33		
31	O	0.000000				
32	C	3.502077	0.000000			
33	O	4.268303	1.140251	0.000000		

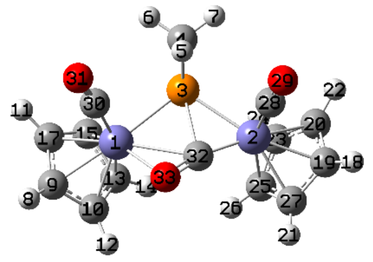

11S -3634.439079 20.53 C1
WBI 0.07

Charge and spin density

		1
1	Fe	0.132939
2	Fe	0.241327
3	P	-0.117124
4	C	-0.023199
9	C	-0.018846
10	C	0.040735
13	C	0.011487
15	C	0.023880
17	C	-0.005420
19	C	-0.030445
20	C	0.033418
23	C	0.047466
25	C	0.008393
27	C	-0.002217
28	C	-0.070666
29	O	-0.098769
30	C	-0.006018
31	O	-0.093801
32	C	0.120142
33	O	-0.193283

		1	2	3	4	5
1	Fe	0.000000				
2	Fe	3.751210	0.000000			
3	P	2.362865	2.360568	0.000000		
4	C	3.417115	3.383585	1.861041	0.000000	
5	H	3.556283	3.544660	2.525575	1.091402	0.000000
6	H	3.571820	4.366772	2.434894	1.093049	1.766821
7	H	4.396890	3.516969	2.443398	1.092184	1.770523
8	H	2.863241	6.367197	5.219159	6.031623	5.952901
9	C	2.100846	5.634731	4.385909	5.409579	5.493403
10	C	2.114911	4.865606	4.071793	5.467602	5.619602
11	H	2.821217	6.405024	4.433638	5.011767	5.312320
12	H	2.840734	4.967762	4.670831	6.082844	6.116673
13	C	2.107440	4.352644	3.324069	4.948814	5.361438
14	H	2.873099	4.040852	3.429444	5.231280	5.722227
15	C	2.082145	4.917032	3.200061	4.520904	5.054490
16	H	2.817028	5.093422	3.166883	4.405592	5.134056
17	C	2.073513	5.658135	3.903583	4.830979	5.138247
18	H	6.496806	2.833153	5.120171	5.731677	5.705565
19	C	5.730137	2.084856	4.348205	5.240351	5.361478
20	C	5.599855	2.118165	3.804547	4.741505	5.137981
21	H	5.353495	2.835519	4.919715	6.115482	6.063378
22	H	6.277340	2.879321	4.225597	4.845033	5.304895
23	C	4.817419	2.123107	3.260168	4.662932	5.203134
24	H	4.849919	2.858259	3.198919	4.650957	5.379939
25	C	4.426659	2.094659	3.551260	5.116801	5.457535
26	H	4.145786	2.854979	3.791140	5.529908	5.889644
27	C	5.072009	2.088511	4.227318	5.466480	5.566577
28	C	4.559224	1.747843	3.051973	3.018995	2.813243
29	O	5.368610	2.891145	3.911073	3.344331	2.908543
30	C	1.757713	4.608987	3.004728	2.997920	2.822260
31	O	2.898200	5.461043	3.851093	3.302346	2.945161
32	C	2.368531	1.736037	2.178271	3.203335	3.098626
33	O	2.039238	2.910946	2.954855	3.804949	3.511506

		6	7	8	9	10
6	H	0.000000				
7	H	1.769755	0.000000			
8	H	5.933155	7.090938	0.000000		
9	C	5.340781	6.425714	1.078762	0.000000	
10	C	5.634150	6.374951	2.227411	1.410404	0.000000
11	H	4.502513	5.991465	2.704251	2.241048	3.328668
12	H	6.387584	6.956810	2.675763	2.216946	1.080472
13	C	5.104170	5.728851	3.342805	2.300176	1.414343
14	H	5.526403	5.854506	4.351508	3.342243	2.231464
15	C	4.379099	5.343116	3.346258	2.303722	2.289209
16	H	4.156574	5.085483	4.355733	3.346249	3.331511

17	C	4.545478	5.806404	2.245764	1.430368	2.284806
18	H	6.804560	5.481608	8.892395	8.247217	7.320548
19	C	6.269065	5.075131	8.167604	7.448133	6.469107
20	C	5.681071	4.426595	8.248786	7.408702	6.492418
21	H	7.069922	6.325026	7.181281	6.603020	5.465339
22	H	5.750048	4.295136	9.030308	8.177639	7.356682
23	C	5.461267	4.582409	7.363197	6.445047	5.452374
24	H	5.281654	4.551218	7.400756	6.408312	5.495772
25	C	5.934497	5.285523	6.656052	5.815455	4.660331
26	H	6.223938	5.860100	6.054859	5.197916	3.929731
27	C	6.424451	5.570103	7.228854	6.530435	5.420414
28	C	4.110628	2.914428	7.147819	6.567848	6.073235
29	O	4.379593	3.082568	7.860687	7.371363	7.010459
30	C	2.861754	4.089854	3.264574	2.963131	3.602639
31	O	2.953799	4.339397	3.898797	3.821840	4.650861
32	C	4.004107	3.833216	4.767216	4.165692	3.567141
33	O	4.418000	4.624432	3.842556	3.438649	3.004577
		11	12	13	14	15
11	H	0.000000				
12	H	4.338347	0.000000			
13	C	3.335525	2.223608	0.000000		
14	H	4.342673	2.685543	1.078302	0.000000	
15	C	2.221473	3.332066	1.423353	2.233523	0.000000
16	H	2.675838	4.340387	2.234189	2.689157	1.078368
17	C	1.079786	3.328031	2.292051	3.333188	1.410490
18	H	9.219865	7.135919	6.908304	6.379837	7.663293
19	C	8.401029	6.342247	5.951511	5.365061	6.725547
20	C	8.030769	6.582553	5.695437	5.057872	6.265447
21	H	8.084469	5.000314	5.340432	4.801106	6.453160
22	H	8.573441	7.532260	6.485881	5.884829	6.886629
23	C	7.092560	5.608591	4.518128	3.776177	5.149555
24	H	6.781119	5.823338	4.354063	3.598895	4.781549
25	C	6.890785	4.585151	3.987239	3.225558	4.950563
26	H	6.429312	3.809535	3.275677	2.418358	4.414952
27	C	7.763597	5.156766	5.033110	4.416924	6.022488
28	C	7.110387	6.201300	5.722696	5.608724	6.043872
29	O	7.791454	7.142499	6.736330	6.698013	6.951750
30	C	2.853362	4.271026	3.785829	4.601043	3.302723
31	O	3.328040	5.297590	4.888929	5.726768	4.265313
32	C	5.173124	3.669364	3.461303	3.545685	4.027594
33	O	4.745842	3.001660	3.369199	3.725343	3.958325
		16	17	18	19	20
16	H	0.000000				
17	C	2.223068	0.000000			
18	H	7.816911	8.435707	0.000000		
19	C	6.856251	7.584238	1.079678	0.000000	

20	C	6.178873	7.280646	2.241054	1.431619	0.000000
21	H	6.899572	7.133540	2.678666	2.224719	3.349315
22	H	6.665553	7.913244	2.698644	2.243669	1.078804
23	C	5.059815	6.281437	3.332251	2.290213	1.407019
24	H	4.492366	6.033337	4.342354	3.333793	2.218103
25	C	5.148654	5.957100	3.329920	2.285415	2.291150
26	H	4.723100	5.428644	4.338500	3.327368	3.332631
27	C	6.319462	6.846951	2.221723	1.410366	2.305561
28	C	6.181844	6.548400	2.919630	2.776282	2.957896
29	O	7.069432	7.335049	3.446741	3.606067	3.833994
30	C	3.809473	2.735078	7.260907	6.666758	6.546593
31	O	4.667277	3.530988	7.997429	7.497235	7.377540
32	C	4.511422	4.416107	4.226571	3.595152	3.828322
33	O	4.671893	3.993246	5.186609	4.625311	4.987189
		21	22	23	24	25
21	H	0.000000				
22	H	4.356771	0.000000			
23	C	3.345627	2.222923	0.000000		
24	H	4.353095	2.677626	1.078761	0.000000	
25	C	2.238270	3.335136	1.419108	2.228447	0.000000
26	H	2.693156	4.343294	2.231491	2.684805	1.078718
27	C	1.078861	3.346546	2.302259	3.343265	1.423628
28	C	3.891502	3.240669	3.575885	4.234543	3.776341
29	O	4.784898	3.886007	4.630768	5.255821	4.893451
30	C	6.521807	7.050961	6.032212	6.072759	5.823650
31	O	7.458607	7.784368	6.986034	7.019174	6.858478
32	C	3.350348	4.595535	3.480643	4.002502	2.970853
33	O	3.915517	5.783145	4.558534	5.033843	3.846981
		26	27	28	29	30
26	H	0.000000				
27	C	2.232852	0.000000			
28	C	4.587174	3.346802	0.000000		
29	O	5.722708	4.337172	1.143973	0.000000	
30	C	5.735276	6.253933	4.787320	5.268658	0.000000
31	O	6.837389	7.201181	5.330721	5.595894	1.142754
32	C	3.186190	3.072664	2.547252	3.490786	3.218571
33	O	3.802736	3.913360	3.516643	4.289209	2.780063
		31	32	33		
31	O	0.000000				
32	C	4.144066	0.000000			
33	O	3.697594	1.193178	0.000000		

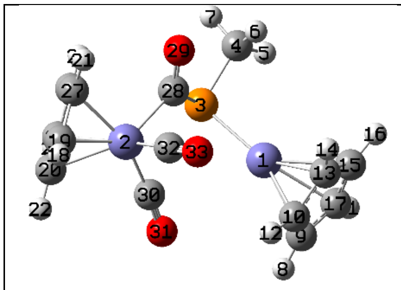

12Q -3634.438548 20.86 C1
WBI 0.01

Charge and spin density

		1	2
1	Fe	0.471134	3.806779
2	Fe	0.154800	0.021779
3	P	-0.214614	-0.043417
4	C	-0.013823	0.014414
9	C	-0.052568	0.032215
10	C	-0.041396	0.015468
13	C	-0.064292	0.045335
15	C	-0.025269	0.063531
17	C	-0.017694	0.038370
19	C	0.022069	-0.000547
20	C	0.002386	-0.000096
23	C	0.044031	-0.001580
25	C	0.044188	0.001419
27	C	0.041841	-0.000237
28	C	0.239744	-0.003042
29	O	-0.279866	0.007127
30	C	-0.095662	-0.002656
31	O	-0.102883	0.004311
32	C	-0.010282	0.001385
33	O	-0.101844	-0.000559

		1	2	3	4	5
1	Fe	0.000000				
2	Fe	4.027829	0.000000			
3	P	2.361677	3.285109	0.000000		
4	C	3.319977	4.772050	1.858429	0.000000	
5	H	3.438153	5.626812	2.446975	1.092221	0.000000
6	H	3.423210	4.890794	2.481331	1.090850	1.785402
7	H	4.323601	5.081572	2.449456	1.092194	1.767957
8	H	2.936924	5.693399	4.883315	6.088705	5.960310
9	C	2.229362	5.481840	4.456095	5.452300	5.335496
10	C	2.209810	4.891445	4.452126	5.419551	5.534962
11	H	3.016605	6.929698	4.817162	5.217258	4.664039
12	H	2.920782	4.581002	4.892313	6.045273	6.306000
13	C	2.261894	5.338945	4.420599	4.902171	4.976331
14	H	2.975395	5.418943	4.810298	5.114685	5.329306
15	C	2.313658	6.116437	4.407173	4.597537	4.362766
16	H	3.040888	6.792650	4.773832	4.530680	4.171252
17	C	2.292511	6.193069	4.427087	4.961926	4.614022
18	H	6.643846	2.857650	6.116778	7.440014	8.378234
19	C	6.107223	2.113005	5.287538	6.631925	7.571192
20	C	5.895830	2.122909	4.989751	6.554057	7.386701
21	H	6.270003	2.843138	5.114139	5.874423	6.940142
22	H	6.270939	2.872410	5.620443	7.306916	8.058254
23	C	5.530429	2.114365	4.122055	5.589222	6.413535
24	H	5.615961	2.866069	4.088584	5.596951	6.296699
25	C	5.557633	2.123278	3.931179	5.037921	6.000972
26	H	5.642716	2.862829	3.691687	4.474020	5.449743
27	C	5.895929	2.103459	4.693204	5.743155	6.760527
28	C	3.138014	2.003720	1.845839	2.869722	3.818994
29	O	3.926992	2.807573	2.689305	2.973522	4.029489
30	C	3.254754	1.751482	3.152375	4.947684	5.528695
31	O	3.305859	2.893162	3.660826	5.478469	5.869681
32	C	3.860600	1.749717	3.984720	5.202780	6.021589
33	O	4.168685	2.892225	4.747878	5.765785	6.529907

		6	7	8	9	10
6	H	0.000000				
7	H	1.769252	0.000000			
8	H	6.323012	7.046551	0.000000		
9	C	5.546132	6.473155	1.080361	0.000000	
10	C	5.277910	6.439386	2.233886	1.421342	0.000000
11	H	5.427506	6.224337	2.688897	2.230219	3.339547
12	H	5.882378	7.000913	2.695468	2.237395	1.080199
13	C	4.537687	5.961328	3.339972	2.295620	1.420075
14	H	4.539021	6.119456	4.350918	3.339944	2.232157
15	C	4.364548	5.689255	3.335630	2.291248	2.293222
16	H	4.188633	5.590983	4.346086	3.335238	3.337195

17	C	5.028800	6.021393	2.231516	1.416659	2.294620
18	H	7.388189	7.621844	7.847021	7.723937	6.885035
19	C	6.686700	6.743231	7.495353	7.377232	6.669337
20	C	6.825148	6.664808	6.989207	7.044041	6.587859
21	H	5.704718	5.807695	8.368285	7.982075	7.152247
22	H	7.624799	7.488382	6.903153	7.109264	6.736045
23	C	6.003586	5.552080	6.979361	6.980609	6.684298
24	H	6.188680	5.517801	6.893946	6.999630	6.922219
25	C	5.292744	4.879482	7.505260	7.305489	6.847414
26	H	4.780587	4.121228	7.851170	7.579809	7.195458
27	C	5.750890	5.710925	7.789190	7.522282	6.816846
28	C	2.906963	3.222512	5.661075	5.170638	4.659409
29	O	2.671056	3.153750	6.627432	6.012747	5.354719
30	C	5.251845	5.469515	4.195493	4.219036	3.945835
31	O	5.867271	6.085757	3.418179	3.701570	3.728799
32	C	4.984332	5.733822	5.462311	5.057093	4.071406
33	O	5.368423	6.392048	5.609077	5.096153	3.885099
		11	12	13	14	15
11	H	0.000000				
12	H	4.352023	0.000000			
13	C	3.334495	2.231875	0.000000		
14	H	4.344986	2.686495	1.080480	0.000000	
15	C	2.226368	3.336345	1.414596	2.229503	0.000000
16	H	2.681211	4.346464	2.226824	2.684357	1.080529
17	C	1.080756	3.339769	2.290663	3.335026	1.413335
18	H	9.489948	6.209070	7.418699	7.258207	8.479914
19	C	8.986494	6.128274	7.174700	7.102696	8.103219
20	C	8.589484	6.107205	7.271210	7.406962	8.054038
21	H	9.284453	6.815450	7.219207	6.920049	8.075558
22	H	8.763652	6.171781	7.596848	7.816876	8.391441
23	C	8.205715	6.425866	7.222216	7.419487	7.796556
24	H	8.048463	6.764006	7.516682	7.847261	7.927686
25	C	8.416473	6.655434	7.120798	7.148780	7.714448
26	H	8.427838	7.151394	7.306827	7.331603	7.749286
27	C	8.873987	6.460023	7.066528	6.921613	7.882633
28	C	6.079144	4.763012	4.616067	4.652168	5.106489
29	O	6.802418	5.469074	5.041739	4.842743	5.557369
30	C	5.776478	3.677606	4.754071	5.158324	5.397572
31	O	5.309298	3.492958	4.775583	5.372484	5.297348
32	C	6.733466	3.592231	4.394363	4.228658	5.471748
33	O	6.850921	3.306122	4.079519	3.704260	5.330209
		16	17	18	19	20
16	H	0.000000				
17	C	2.226592	0.000000			
18	H	9.195663	8.645961	0.000000		
19	C	8.787243	8.213997	1.079819	0.000000	

20	C	8.802248	7.928223	2.220918	1.407761	0.000000
21	H	8.518116	8.505369	2.699434	2.242107	3.338886
22	H	9.223801	8.123811	2.678640	2.222734	1.079467
23	C	8.440718	7.658854	3.337167	2.292186	1.428916
24	H	8.570280	7.629468	4.346956	3.334989	2.241710
25	C	8.223666	7.818889	3.337795	2.294949	2.297537
26	H	8.135543	7.907610	4.346680	3.337519	3.340586
27	C	8.419255	8.137366	2.237136	1.426670	2.296237
28	C	5.527718	5.422468	4.578939	3.825689	3.949094
29	O	5.802778	6.121815	4.864600	4.200889	4.642183
30	C	6.238127	5.115881	4.038722	3.442143	2.849255
31	O	6.226541	4.738456	4.934557	4.422192	3.653907
32	C	6.161295	5.813314	3.065035	2.896023	3.438707
33	O	5.998832	5.844397	3.670408	3.772689	4.459147
		21	22	23	24	25
21	H	0.000000				
22	H	4.350708	0.000000			
23	C	3.331657	2.240710	0.000000		
24	H	4.339530	2.699942	1.079288	0.000000	
25	C	2.225558	3.339966	1.411677	2.223709	0.000000
26	H	2.676741	4.349568	2.223069	2.677376	1.078517
27	C	1.078631	3.340813	2.290747	3.333141	1.415077
28	C	3.360381	4.773982	3.323571	3.773833	2.733739
29	O	3.048227	5.575360	4.043667	4.615457	3.070543
30	C	4.593030	3.037268	2.881013	3.102172	3.507190
31	O	5.729822	3.543925	3.734037	3.707594	4.549851
32	C	3.252601	3.993482	3.818480	4.604955	3.600874
33	O	3.939774	4.923533	4.947765	5.743510	4.669210
		26	27	28	29	30
26	H	0.000000				
27	C	2.224643	0.000000			
28	C	2.707944	3.086851	0.000000		
29	O	2.836632	3.170239	1.203568	0.000000	
30	C	4.131823	3.793437	2.820913	3.941680	0.000000
31	O	5.137819	4.904812	3.781493	4.940161	1.145385
32	C	4.242239	3.000996	2.566198	3.003077	2.550606
33	O	5.265288	3.911822	3.405445	3.619904	3.471081
		31	32	33		
31	O	0.000000				
32	C	3.475093	0.000000			
33	O	4.217611	1.142522	0.000000		

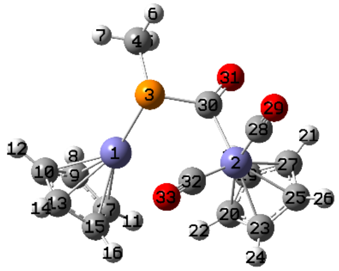

13Q -3634.438139 21.12 C1
WBI 0.02

Charge and spin density

		1	2
1	Fe	0.470658	3.795501
2	Fe	0.100009	0.038445
3	P	-0.175690	-0.032374
4	C	-0.012056	0.012003
9	C	-0.047882	0.033101
10	C	-0.033446	0.032754
13	C	-0.013209	0.063694
15	C	-0.060917	0.034011
17	C	-0.063281	0.017749
19	C	0.003414	0.007651
20	C	0.014329	0.001913
23	C	0.007300	-0.000989
25	C	0.017618	0.001446
27	C	0.080182	-0.000965
28	C	-0.001715	-0.002751
29	O	-0.083740	-0.001356
30	C	0.214429	0.000372
31	O	-0.284971	0.005824
32	C	-0.040094	-0.004018
33	O	-0.090938	-0.002011

		1	2	3	4	5
1	Fe	0.000000				
2	Fe	3.855190	0.000000			
3	P	2.339554	3.283062	0.000000		
4	C	3.411323	4.781840	1.852832	0.000000	
5	H	3.555923	4.909408	2.480768	1.090315	0.000000
6	H	4.389283	5.101413	2.440399	1.091560	1.768439
7	H	3.549540	5.625430	2.439875	1.091875	1.782512
8	H	2.939451	6.295876	4.830079	4.927255	4.549345
9	C	2.227754	5.736510	4.403158	4.875510	4.696524
10	C	2.286376	6.065277	4.302864	4.874103	4.992862
11	H	2.947021	4.922236	5.008696	5.877808	5.562253
12	H	3.006166	6.838058	4.630632	4.897576	5.087712
13	C	2.321413	5.570964	4.341010	5.391894	5.657404
14	H	3.043099	5.970382	4.678517	5.820340	6.255214
15	C	2.280541	4.851275	4.457432	5.687151	5.788446
16	H	3.003698	4.659528	4.907440	6.353082	6.500100
17	C	2.226040	4.969441	4.499646	5.396322	5.234256
18	H	3.800657	2.858725	4.078309	4.800461	4.299059
19	C	4.066689	2.121276	4.231758	5.278180	4.960067
20	C	3.875452	2.118826	4.480365	5.864290	5.723830
21	H	5.813043	2.836239	5.125334	5.880485	5.561094
22	H	3.426364	2.878084	4.586392	5.981227	5.851842
23	C	4.957090	2.127812	5.178984	6.702281	6.689815
24	H	5.448236	2.878829	5.798313	7.448286	7.532317
25	C	5.634142	2.112168	5.344078	6.676166	6.610359
26	H	6.583160	2.855973	6.077078	7.404811	7.399593
27	C	5.173410	2.102696	4.793929	5.825047	5.582026
28	C	5.067376	1.752818	3.703557	5.031507	5.355445
29	O	6.011499	2.892969	4.365357	5.512233	5.926274
30	C	3.212534	2.001611	1.835372	2.882109	2.978637
31	O	4.110733	2.808641	2.677237	2.995150	2.800645
32	C	3.407240	1.760249	3.106304	4.929629	5.348738
33	O	3.686985	2.897602	3.627756	5.475934	6.024672

		6	7	8	9	10
6	H	0.000000				
7	H	1.769385	0.000000			
8	H	6.009815	4.759965	0.000000		
9	C	5.961227	4.732387	1.080151	0.000000	
10	C	5.920821	4.512776	2.232312	1.418361	0.000000
11	H	6.870910	6.115646	2.689418	2.232514	3.338214
12	H	5.906171	4.309184	2.688750	2.230959	1.080199
13	C	6.364056	5.176195	3.335181	2.291510	1.412011
14	H	6.706208	5.531199	4.345625	3.335395	2.225404
15	C	6.656245	5.724562	3.338797	2.294175	2.287997
16	H	7.247923	6.493954	4.352256	3.340025	3.330271

17	C	6.426638	5.487786	2.234144	1.420720	2.294679
18	H	5.385462	5.622051	4.883444	4.752135	5.694508
19	C	5.785416	6.130807	5.583295	5.270064	6.070827
20	C	6.463587	6.585084	5.595650	5.051077	5.708363
21	H	6.000371	6.916163	7.456591	7.253292	7.985509
22	H	6.719911	6.553303	4.917951	4.287901	4.949894
23	C	7.143705	7.480758	6.961849	6.344843	6.843780
24	H	7.900742	8.156622	7.519039	6.795061	7.163453
25	C	6.936250	7.589100	7.657821	7.166164	7.719724
26	H	7.541918	8.346076	8.713927	8.204440	8.695033
27	C	6.094019	6.792525	6.926291	6.607655	7.309709
28	C	4.982307	5.930609	7.687670	7.151051	7.297463
29	O	5.245859	6.401658	8.673012	8.151350	8.212572
30	C	3.176270	3.837945	5.492318	5.154055	5.466910
31	O	3.066475	4.068632	5.951491	5.812622	6.277392
32	C	5.341571	5.514173	6.203802	5.415371	5.357606
33	O	5.914744	5.881214	6.498014	5.592741	5.276956
		11	12	13	14	15
11	H	0.000000				
12	H	4.349010	0.000000			
13	C	3.337615	2.225096	0.000000		
14	H	4.347720	2.680626	1.080157	0.000000	
15	C	2.236422	3.331461	1.412599	2.224164	0.000000
16	H	2.699635	4.339044	2.222702	2.673771	1.080334
17	C	1.080325	3.338928	2.292933	3.336170	1.419805
18	H	3.603497	6.506277	5.722071	6.540676	4.806000
19	C	3.940849	6.942752	5.847310	6.556425	4.842246
20	C	3.510851	6.676905	5.190819	5.809619	4.044117
21	H	6.124933	8.743709	7.814924	8.431341	6.944035
22	H	2.620474	5.974669	4.377100	5.047478	3.117462
23	C	4.841313	7.795634	6.141343	6.580711	5.043702
24	H	5.278992	8.132570	6.277451	6.587684	5.179526
25	C	5.774888	8.603656	7.165090	7.625274	6.159103
26	H	6.837325	9.558744	8.082631	8.463177	7.113909
27	C	5.340410	8.139061	7.012681	7.612985	6.061613
28	C	6.607440	7.923882	6.810113	7.033733	6.317871
29	O	7.723808	8.760154	7.733590	7.869782	7.346282
30	C	5.005189	6.016625	5.402369	5.887027	5.038151
31	O	5.640871	6.765112	6.395323	6.954286	6.014389
32	C	4.938784	6.092123	4.569678	4.701462	4.071939
33	O	5.306572	5.962509	4.280057	4.162414	3.969546
		16	17	18	19	20
16	H	0.000000				
17	C	2.235607	0.000000			
18	H	4.973252	4.098227	0.000000		
19	C	4.781953	4.410956	1.078671	0.000000	

20	C	3.723773	3.929751	2.228095	1.413171	0.000000
21	H	6.863043	6.555120	2.671127	2.223928	3.330311
22	H	2.776114	3.035065	2.686363	2.226045	1.079025
23	C	4.501372	5.194387	3.343754	2.299651	1.427271
24	H	4.444657	5.565963	4.353412	3.342157	2.240067
25	C	5.748705	6.157869	3.335113	2.293397	2.288594
26	H	6.639139	7.197917	4.343496	3.336025	3.333409
27	C	5.891437	5.771575	2.222057	1.414574	2.289742
28	C	6.131890	6.548318	4.267457	3.613651	3.815373
29	O	7.162310	7.622659	5.295673	4.679925	4.937137
30	C	5.268348	4.876976	2.801475	2.803822	3.422366
31	O	6.305325	5.638847	2.858038	3.073080	4.074862
32	C	3.765153	4.670884	4.136799	3.519059	2.908463
33	O	3.557816	4.877713	5.145082	4.558684	3.753814
		21	22	23	24	25
21	H	0.000000				
22	H	4.339031	0.000000			
23	C	3.340078	2.237220	0.000000		
24	H	4.351528	2.695435	1.079227	0.000000	
25	C	2.241462	3.330502	1.408070	2.223423	0.000000
26	H	2.698374	4.341960	2.220222	2.678265	1.079771
27	C	1.078366	3.332599	2.297622	3.341818	1.425282
28	C	3.260619	4.612833	3.408830	3.951763	2.863354
29	O	3.943970	5.746355	4.411887	4.858106	3.720872
30	C	3.300916	3.919700	3.990638	4.817461	3.807096
31	O	2.941800	4.685829	4.638039	5.578135	4.147928
32	C	4.595703	3.139522	2.882080	3.077045	3.463193
33	O	5.728025	3.741563	3.671441	3.567522	4.430416
		26	27	28	29	30
26	H	0.000000				
27	C	2.235268	0.000000			
28	C	3.015405	3.004459	0.000000		
29	O	3.590029	3.907101	1.140258	0.000000	
30	C	4.526824	3.074777	2.507609	3.336904	0.000000
31	O	4.789367	3.111080	3.023604	3.648961	1.204509
32	C	4.058056	3.806806	2.534080	3.451866	2.841407
33	O	4.939906	4.911474	3.451026	4.188513	3.821182
		31	32	33		
31	O	0.000000				
32	C	3.976048	0.000000			
33	O	4.995544	1.143608	0.000000		

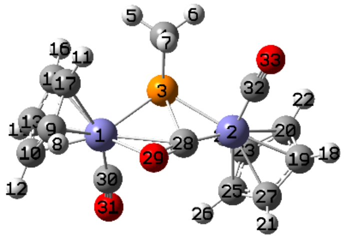

14S -3634.437195 21.71 C1
WBI 0.06

Charge and spin density

		1
1	Fe	0.169774
2	Fe	0.238883
3	P	-0.095819
4	C	-0.046981
9	C	0.061535
10	C	-0.043004
13	C	0.019985
15	C	-0.026596
17	C	0.018750
19	C	-0.018363
20	C	0.026642
23	C	0.055060
25	C	0.048074
27	C	-0.001784
28	C	0.055426
29	O	-0.178455
30	C	0.007192
31	O	-0.120983
32	C	-0.070882
33	O	-0.098455

		1	2	3	4	5
1	Fe	0.000000				
2	Fe	3.764219	0.000000			
3	P	2.365277	2.366263	0.000000		
4	C	3.323239	3.367477	1.864996	0.000000	
5	H	3.491657	4.359916	2.447657	1.092497	0.000000
6	H	4.321895	3.497661	2.449135	1.092473	1.763576
7	H	3.412347	3.512273	2.524025	1.092221	1.769248
8	H	2.844357	5.801675	4.857838	4.957203	4.973360
9	C	2.117159	5.519326	4.200284	4.402443	4.285788
10	C	2.106886	5.828321	4.383495	5.036766	4.866136
11	H	2.873725	5.235452	3.683019	2.923859	2.616457
12	H	2.864783	6.395600	5.187667	6.014283	5.909810
13	C	2.075020	5.687778	3.807869	4.505214	4.153850
14	H	2.827141	6.161182	4.251008	5.162053	4.777403
15	C	2.084335	5.294304	3.196345	3.371474	2.858437
16	H	2.828254	5.440134	3.137157	3.109367	2.363366
17	C	2.108980	5.174552	3.480189	3.275754	2.967415
18	H	6.514034	2.834057	5.127935	5.749117	6.821056
19	C	5.735870	2.084239	4.349508	5.255528	6.285689
20	C	5.594057	2.116571	3.798962	4.797596	5.732582
21	H	5.354876	2.834775	4.918171	6.065746	7.033303
22	H	6.273622	2.876885	4.220675	4.934744	5.828157
23	C	4.793218	2.121591	3.243964	4.701181	5.502543
24	H	4.811663	2.855891	3.173522	4.708265	5.341946
25	C	4.402543	2.095727	3.538312	5.105405	5.934258
26	H	4.109596	2.858507	3.774210	5.499195	6.208033
27	C	5.069407	2.088725	4.224302	5.444060	6.412098
28	C	2.417199	1.730083	2.206233	3.112986	3.944992
29	O	2.060974	2.907279	2.948699	3.652526	4.291079
30	C	1.753713	3.867658	2.954730	4.534356	4.802002
31	O	2.895910	4.414451	3.781695	5.504726	5.784578
32	C	4.605663	1.748821	3.063801	3.002181	4.093528
33	O	5.429037	2.891965	3.926352	3.334261	4.361834

		6	7	8	9	10
6	H	0.000000				
7	H	1.770645	0.000000			
8	H	6.013150	4.447093	0.000000		
9	C	5.491381	4.106857	1.080243	0.000000	
10	C	6.107700	5.002982	2.214830	1.406286	0.000000
11	H	3.927819	2.499035	2.681930	2.231111	3.338868
12	H	7.072470	5.958644	2.672344	2.222617	1.079002
13	C	5.512151	4.763967	3.331206	2.287707	1.432709
14	H	6.084217	5.581560	4.341544	3.331202	2.245293
15	C	4.382060	3.636007	3.330566	2.288233	2.300560
16	H	3.969274	3.628185	4.343353	3.333854	3.342006

17	C	4.355351	3.088315	2.226333	1.418321	2.298902
18	H	5.492913	5.726734	8.178565	8.108472	8.501480
19	C	5.099850	5.367371	7.646233	7.477224	7.734280
20	C	4.514597	5.192237	7.876884	7.515437	7.655888
21	H	6.285920	5.965151	6.828741	6.855728	7.018437
22	H	4.421793	5.411575	8.577685	8.171109	8.362752
23	C	4.669302	5.215565	7.308515	6.843220	6.755805
24	H	4.673435	5.409845	7.523103	6.918080	6.691234
25	C	5.313308	5.400594	6.670587	6.343918	6.225100
26	H	5.875825	5.799035	6.367732	6.015244	5.694947
27	C	5.565189	5.506786	6.917666	6.789347	6.910456
28	C	3.734616	2.947926	4.097470	3.907875	4.338811
29	O	4.470978	3.280770	3.034151	3.024739	3.567700
30	C	5.352432	4.826414	4.152102	3.517643	2.868339
31	O	6.229355	5.875900	5.143884	4.544125	3.696827
32	C	2.820630	2.846785	6.121427	5.958543	6.611867
33	O	2.955317	2.991094	6.612601	6.526890	7.342463
		11	12	13	14	15
11	H	0.000000				
12	H	4.347665	0.000000			
13	C	3.337630	2.246472	0.000000		
14	H	4.347170	2.707715	1.079379	0.000000	
15	C	2.235907	3.343353	1.411337	2.223317	0.000000
16	H	2.701399	4.350744	2.217360	2.667838	1.079135
17	C	1.078138	3.341884	2.294940	3.337759	1.420516
18	H	7.757594	8.954455	8.491927	8.947035	8.105457
19	C	7.264981	8.168271	7.654374	8.034666	7.358237
20	C	7.151098	8.176660	7.312187	7.568731	6.961035
21	H	7.255343	7.193119	7.301305	7.716399	7.325396
22	H	7.552349	8.964894	7.899252	8.141383	7.411076
23	C	6.785497	7.200287	6.352137	6.478816	6.209221
24	H	6.835628	7.166253	6.079925	6.059706	5.962918
25	C	6.668772	6.508601	6.101859	6.294266	6.163842
26	H	6.678883	5.837705	5.631890	5.730813	5.933484
27	C	6.990951	7.196972	6.981798	7.335822	6.918582
28	C	3.955628	4.897047	4.483578	5.145891	4.175164
29	O	3.526596	4.045879	4.055097	4.843401	3.896892
30	C	4.619438	3.110565	2.743551	2.898828	3.369336
31	O	5.752532	3.683855	3.533445	3.375805	4.344182
32	C	5.085816	7.323216	6.497413	7.135332	5.766697
33	O	5.354706	8.109239	7.247615	7.954065	6.366718
		16	17	18	19	20
16	H	0.000000				
17	C	2.236558	0.000000			
18	H	8.224247	7.849710	0.000000		
19	C	7.475206	7.236501	1.079651	0.000000	

20	C	6.894544	7.085925	2.241924	1.432274	0.000000
21	H	7.743808	7.047770	2.678802	2.224272	3.348634
22	H	7.201494	7.582879	2.699676	2.244253	1.078846
23	C	6.190814	6.518202	3.332518	2.290440	1.406646
24	H	5.814315	6.498759	4.343103	3.334324	2.218346
25	C	6.388752	6.308189	3.331911	2.287478	2.292614
26	H	6.262158	6.158754	4.342310	3.330693	3.332922
27	C	7.204360	6.792459	2.222310	1.410394	2.304814
28	C	4.614979	3.792729	4.213317	3.581540	3.820617
29	O	4.580965	3.260672	5.194178	4.625645	4.980119
30	C	3.940056	3.789334	6.429412	5.491048	5.290592
31	O	4.830434	4.893064	6.725540	5.723873	5.481560
32	C	5.812854	5.383822	2.925041	2.776324	2.942089
33	O	6.357699	5.852816	3.451248	3.604545	3.814223
		21	22	23	24	25
21	H	0.000000				
22	H	4.356295	0.000000			
23	C	3.344113	2.222666	0.000000		
24	H	4.351079	2.678223	1.078834	0.000000	
25	C	2.237433	3.336536	1.419738	2.227680	0.000000
26	H	2.697572	4.342963	2.229805	2.679749	1.078772
27	C	1.078938	3.345964	2.300710	3.341431	1.423589
28	C	3.330661	4.589299	3.475617	4.000744	2.961811
29	O	3.917134	5.775975	4.543356	5.012231	3.833447
30	C	4.882282	6.100689	4.169271	4.098490	3.594975
31	O	5.005066	6.330035	4.207518	4.053506	3.566074
32	C	3.908687	3.215306	3.563390	4.216550	3.779113
33	O	4.805635	3.852757	4.615419	5.233634	4.895850
		26	27	28	29	30
26	H	0.000000				
27	C	2.236500	0.000000			
28	C	3.183661	3.057410	0.000000		
29	O	3.789237	3.911707	1.189740	0.000000	
30	C	2.950194	4.579079	2.956129	2.866300	0.000000
31	O	2.687077	4.707473	3.800952	3.811110	1.145283
32	C	4.594938	3.357015	2.559257	3.545201	5.238000
33	O	5.731094	4.348153	3.506789	4.329292	6.258728
		31	32	33		
31	O	0.000000				
32	C	5.970670	0.000000			
33	O	7.053390	1.143910	0.000000		

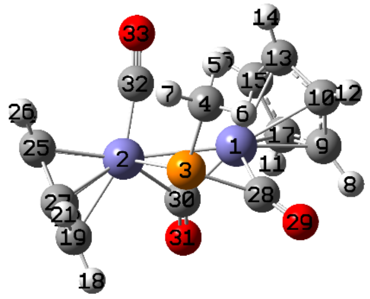

15T -3634.432302 24.78 C1
WBI 0.23

Charge and spin density

		1	2
1	Fe	0.167648	2.373174
2	Fe	-0.056848	0.045163
3	P	0.020894	-0.098737
4	C	0.002977	-0.004757
9	C	-0.022944	-0.015240
10	C	-0.040622	0.036464
13	C	-0.028299	0.019336
15	C	-0.046603	0.050306
17	C	0.012396	0.006786
19	C	0.001495	-0.007603
20	C	0.047669	-0.000990
23	C	0.004266	-0.002251
25	C	0.007638	-0.000490
27	C	0.102318	0.009507
28	C	0.094583	-0.132050
29	O	-0.182039	-0.103186
30	C	0.170400	-0.085000
31	O	-0.172517	-0.081407
32	C	0.030919	-0.006057
33	O	-0.113333	-0.002967

		1	2	3	4	5
1	Fe	0.000000				
2	Fe	2.601300	0.000000			
3	P	2.413516	2.294770	0.000000		
4	C	3.258742	3.563160	1.856411	0.000000	
5	H	3.093348	3.690826	2.515657	1.089991	0.000000
6	H	3.742494	4.474761	2.439422	1.092187	1.779355
7	H	4.170806	3.858924	2.418705	1.093258	1.771962
8	H	2.874564	5.328835	4.740581	5.403378	5.249702
9	C	2.183343	4.714654	4.344537	4.868343	4.548388
10	C	2.243392	4.743676	4.074434	4.044573	3.533422
11	H	2.937624	4.584054	5.232670	6.172425	5.835012
12	H	2.963778	5.380472	4.268584	3.920207	3.477757
13	C	2.287920	4.301670	4.185193	4.039725	3.271803
14	H	3.017848	4.604530	4.450712	3.889212	2.926444
15	C	2.292436	4.004515	4.541902	4.881967	4.235145
16	H	3.028027	4.074815	5.074514	5.421385	4.721138
17	C	2.230893	4.284895	4.636486	5.331099	4.911578
18	H	4.503367	2.864289	3.714793	5.486490	6.027775
19	C	4.254043	2.110214	3.444924	5.059842	5.498086
20	C	4.298709	2.094396	4.150001	5.591691	5.769983
21	H	4.886206	2.868773	2.981909	4.128172	4.839674
22	H	4.563778	2.830033	4.876507	6.374711	6.476511
23	C	4.539590	2.091194	4.233566	5.280438	5.329396
24	H	5.004945	2.838276	5.024534	5.862550	5.729444
25	C	4.638174	2.099719	3.598910	4.472949	4.709392
26	H	5.183692	2.858247	3.992155	4.428298	4.604780
27	C	4.485572	2.125489	3.063180	4.341186	4.853687
28	C	1.842976	3.424615	1.935151	2.950038	3.313387
29	O	2.902475	4.543817	2.862943	3.656368	4.113577
30	C	1.911834	1.971509	2.961810	4.466803	4.537033
31	O	2.893229	2.935507	3.969768	5.574697	5.684417
32	C	2.691333	1.748801	3.027983	3.405532	3.018552
33	O	3.417737	2.888555	3.912314	3.800156	3.132167

		6	7	8	9	10
6	H	0.000000				
7	H	1.767163	0.000000			
8	H	5.359613	6.470370	0.000000		
9	C	4.982926	5.918708	1.079563	0.000000	
10	C	4.111752	5.096256	2.231407	1.421597	0.000000
11	H	6.586346	7.079548	2.688141	2.229648	3.341627
12	H	3.715095	4.980117	2.684912	2.232762	1.079461
13	C	4.421626	4.949255	3.326385	2.283750	1.409784
14	H	4.321021	4.667105	4.338632	3.329430	2.223147
15	C	5.412713	5.733243	3.332923	2.289089	2.295197
16	H	6.094787	6.139604	4.344509	3.333501	3.336889

17	C	5.708429	6.277927	2.233965	1.419501	2.299363
18	H	6.105544	5.678682	6.394173	6.219315	6.700197
19	C	5.830724	5.160050	6.526069	6.173839	6.494000
20	C	6.481344	5.743350	6.626165	6.126563	6.483581
21	H	4.830584	3.828140	7.344744	6.977721	6.908980
22	H	7.225421	6.642702	6.565138	6.109961	6.661709
23	C	6.291927	5.258288	7.180801	6.514448	6.592030
24	H	6.914309	5.840991	7.610939	6.848452	6.879866
25	C	5.473417	4.225235	7.415849	6.785068	6.663263
26	H	5.454636	3.963954	8.035371	7.338771	7.020831
27	C	5.177256	4.178451	7.047911	6.601196	6.622859
28	C	2.978901	3.895706	2.992186	2.891102	2.941423
29	O	3.340407	4.583755	3.008908	3.277870	3.412395
30	C	5.092929	5.127555	3.849265	3.427002	4.013353
31	O	6.132694	6.233399	4.078535	3.868294	4.758104
32	C	4.415785	3.713151	5.479493	4.602885	4.253972
33	O	4.790105	4.034937	6.014849	5.035758	4.463164
		11	12	13	14	15
11	H	0.000000				
12	H	4.350123	0.000000			
13	C	3.331266	2.228609	0.000000		
14	H	4.340016	2.686985	1.080699	0.000000	
15	C	2.226316	3.340163	1.417368	2.226559	0.000000
16	H	2.685557	4.348478	2.229791	2.679772	1.079458
17	C	1.079294	3.341942	2.286464	3.328944	1.410828
18	H	5.824633	7.257720	6.680049	7.210873	6.220610
19	C	5.751127	7.092283	6.265393	6.676492	5.814729
20	C	5.291401	7.242108	6.006109	6.399445	5.314878
21	H	7.183767	7.225437	6.743771	6.904503	6.743622
22	H	4.913745	7.512850	6.196036	6.703438	5.291183
23	C	5.846988	7.315823	5.881839	6.040873	5.331682
24	H	6.008516	7.666137	5.983892	6.056209	5.341452
25	C	6.567024	7.207519	6.064349	6.101556	5.835489
26	H	7.287404	7.482837	6.331708	6.184090	6.268931
27	C	6.521692	7.090137	6.314323	6.516031	6.125718
28	C	4.212746	3.088730	3.640832	4.267896	4.004211
29	O	4.833842	3.284690	4.418902	5.070765	4.871837
30	C	2.955281	4.804393	3.917875	4.644341	3.282341
31	O	2.898377	5.581829	4.771714	5.599514	3.931737
32	C	4.600327	4.920330	3.367216	3.350519	3.228480
33	O	5.093730	5.091120	3.323612	2.985863	3.333042
		16	17	18	19	20
16	H	0.000000				
17	C	2.225159	0.000000			
18	H	6.392713	5.921225	0.000000		
19	C	5.862430	5.756935	1.078757	0.000000	

20	C	5.122645	5.401650	2.237426	1.426173	0.000000
21	H	6.911888	6.892418	2.679061	2.220599	3.333958
22	H	5.035555	5.231066	2.689149	2.234124	1.078656
23	C	4.992660	5.770504	3.334930	2.293773	1.406548
24	H	4.798146	5.950395	4.345501	3.338048	2.220100
25	C	5.673541	6.309450	3.336138	2.293464	2.295714
26	H	6.071953	6.916763	4.345797	3.336469	3.337742
27	C	6.174467	6.312934	2.228226	1.412535	2.291651
28	C	4.846342	3.609371	4.440987	4.505403	5.086709
29	O	5.803021	4.281293	5.131129	5.377694	6.086412
30	C	3.599120	2.926008	3.011696	2.865780	2.778690
31	O	4.190478	3.263448	3.018579	3.154871	2.992094
32	C	3.092244	4.064841	4.603843	3.777835	3.361341
33	O	3.016856	4.467927	5.733501	4.867765	4.336216
		21	22	23	24	25
21	H	0.000000				
22	H	4.340523	0.000000			
23	C	3.336572	2.220871	0.000000		
24	H	4.346584	2.677601	1.079571	0.000000	
25	C	2.222292	3.339893	1.430398	2.241626	0.000000
26	H	2.681848	4.349025	2.243009	2.700735	1.079055
27	C	1.079008	3.332621	2.292717	3.335798	1.411737
28	C	4.651430	5.482425	5.503119	6.215687	5.236007
29	O	5.414006	6.437639	6.600462	7.353097	6.282186
30	C	4.276327	2.833359	3.502261	4.096586	3.949072
31	O	4.926402	2.667499	4.037518	4.603499	4.681603
32	C	4.163937	3.935715	2.734946	2.885229	2.831372
33	O	5.098420	4.851413	3.497368	3.338661	3.598485
		26	27	28	29	30
26	H	0.000000				
27	C	2.227381	0.000000			
28	C	5.773112	4.632029	0.000000		
29	O	6.815298	5.537745	1.173693	0.000000	
30	C	4.809696	3.626696	2.835862	3.802561	0.000000
31	O	5.643392	4.244721	3.594088	4.349800	1.166424
32	C	3.068701	3.507853	4.031420	5.198518	2.973696
33	O	3.549392	4.487802	4.841872	5.974653	3.994301
		31	32	33		
31	O	0.000000				
32	C	4.025713	0.000000			
33	O	5.016234	1.147378	0.000000		

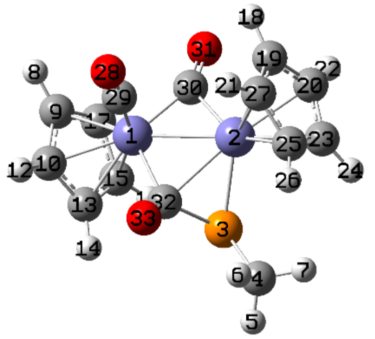

16T -3634.428006 27.48 C1
WBI 0.27

Charge and spin density

		1	2
1	Fe	-0.020535	-0.019417
2	Fe	0.002633	2.275829
3	P	-0.095817	-0.147771
4	C	-0.039402	0.002347
9	C	-0.007070	0.003927
10	C	0.027074	-0.003514
13	C	0.046522	0.000732
15	C	0.059140	0.012797
17	C	0.082229	-0.010472
19	C	-0.027344	-0.047808
20	C	0.001595	0.013629
23	C	-0.034281	-0.032009
25	C	0.001994	-0.018924
27	C	0.024164	0.062375
28	O	-0.087549	-0.003686
29	C	0.085366	-0.004002
30	C	0.095230	-0.033601
31	O	-0.151502	0.006999
32	C	0.324656	-0.051947
33	O	-0.287103	-0.005485

		1	2	3	4	5
1	Fe	0.000000				
2	Fe	2.543323	0.000000			
3	P	3.076205	2.291663	0.000000		
4	C	4.666900	3.410215	1.853164	0.000000	
5	H	5.309250	4.375455	2.437949	1.092611	0.000000
6	H	4.714177	3.569958	2.453196	1.091562	1.773934
7	H	5.305633	3.566920	2.468655	1.091643	1.767835
8	H	2.846113	5.194998	5.845818	7.452805	7.993318
9	C	2.105274	4.594384	4.925239	6.564062	7.036627
10	C	2.093259	4.550525	4.411254	5.834456	6.199967
11	H	2.862202	4.575247	4.974527	6.817416	7.286991
12	H	2.833744	5.126782	5.013542	6.204159	6.557117
13	C	2.133878	4.194398	3.435792	4.907085	5.127544
14	H	2.885227	4.507598	3.254011	4.422735	4.464537
15	C	2.128723	3.958113	3.395203	5.144951	5.421194
16	H	2.876432	4.079645	3.168722	4.904890	5.076380
17	C	2.125091	4.242845	4.398311	6.194964	6.624309
18	H	4.215078	2.938125	5.224861	6.174570	7.212296
19	C	4.043548	2.191372	4.439645	5.210906	6.267354
20	C	4.430516	2.161057	4.025251	4.746605	5.744785
21	H	4.340961	2.988428	4.816192	5.122822	6.180357
22	H	4.875398	2.880184	4.529197	5.409173	6.323969
23	C	4.662596	2.120746	3.388112	3.617042	4.630572
24	H	5.298010	2.842837	3.418605	3.370229	4.253688
25	C	4.497591	2.184320	3.534774	3.496226	4.584418
26	H	4.993180	2.909506	3.656500	3.095489	4.133725
27	C	4.146865	2.255271	4.215613	4.588467	5.674989
28	O	2.898204	3.737889	5.146823	6.125963	6.971129
29	C	1.758958	2.970927	4.196606	5.389966	6.188247
30	C	1.838634	2.035341	3.480809	5.099586	5.892892
31	O	2.884631	2.929413	4.446515	6.063050	6.852426
32	C	2.037976	2.278540	1.815086	2.864469	3.517132
33	O	2.877809	3.158145	2.758320	3.157625	3.739728

		6	7	8	9	10
6	H	0.000000				
7	H	1.785318	0.000000			
8	H	7.423795	8.145072	0.000000		
9	C	6.607365	7.294331	1.079326	0.000000	
10	C	5.764064	6.703462	2.241482	1.429322	0.000000
11	H	7.177422	7.328857	2.676012	2.218282	3.334273
12	H	5.953621	7.125012	2.701895	2.242599	1.079268
13	C	5.033146	5.799276	3.341491	2.298903	1.413156
14	H	4.520188	5.410693	4.350819	3.341595	2.223854
15	C	5.508750	5.860055	3.338550	2.294384	2.289692
16	H	5.467803	5.529880	4.347403	3.336447	3.331191

17	C	6.467633	6.829481	2.222684	1.407266	2.290544
18	H	6.153493	6.094902	5.701935	5.638531	6.127885
19	C	5.206367	5.062576	6.020698	5.774209	6.051512
20	C	5.010113	4.395664	6.680988	6.264249	6.519122
21	H	4.652774	5.173860	6.248948	6.056499	5.942775
22	H	5.826492	5.014519	6.935186	6.532489	6.954890
23	C	3.871576	3.119148	7.253953	6.694315	6.653088
24	H	3.843907	2.627092	7.983088	7.327876	7.220679
25	C	3.326395	3.221030	7.053932	6.555632	6.334430
26	H	2.693166	2.832376	7.611333	7.058627	6.627822
27	C	4.324548	4.501239	6.330905	6.025798	5.987103
28	O	5.695166	6.646093	3.601702	3.691447	3.682423
29	C	5.113328	5.959143	3.041885	2.857590	2.843856
30	C	5.337617	5.392646	3.686210	3.235495	3.794157
31	O	6.377629	6.233854	4.043554	3.786687	4.640206
32	C	2.736027	3.666574	4.690549	3.881797	3.150973
33	O	2.612850	4.029190	5.159328	4.457338	3.483905
		11	12	13	14	15
11	H	0.000000				
12	H	4.344361	0.000000			
13	C	3.335055	2.224624	0.000000		
14	H	4.344379	2.677349	1.078269	0.000000	
15	C	2.232680	3.332012	1.414783	2.226186	0.000000
16	H	2.692599	4.339169	2.225782	2.680163	1.078728
17	C	1.079181	3.333706	2.292920	3.335734	1.422498
18	H	5.457505	6.593564	6.327623	6.935328	5.946349
19	C	5.634041	6.521270	6.056277	6.522672	5.738218
20	C	5.736327	7.132315	6.223489	6.606120	5.712333
21	H	6.689443	6.046317	6.161914	6.441357	6.354746
22	H	5.634688	7.677062	6.605508	7.061874	5.876804
23	C	6.398267	7.187283	6.182893	6.334765	5.870644
24	H	6.886810	7.801085	6.561742	6.599759	6.196578
25	C	6.733614	6.667175	6.045001	6.129369	6.046052
26	H	7.437058	6.846340	6.281561	6.189421	6.474062
27	C	6.325378	6.267884	5.999656	6.283188	5.997814
28	O	5.047941	3.591667	4.515622	5.078296	4.936230
29	C	4.097065	3.024541	3.498815	4.119958	3.824161
30	C	2.773680	4.587919	3.727568	4.477918	3.071151
31	O	2.752268	5.475778	4.636427	5.465008	3.753494
32	C	4.685474	3.510070	2.645921	2.594887	3.163977
33	O	5.649292	3.470752	3.236427	2.975516	4.099752
		16	17	18	19	20
16	H	0.000000				
17	C	2.236900	0.000000			
18	H	6.261344	5.520535	0.000000		
19	C	5.950307	5.575652	1.079845	0.000000	

20	C	5.661076	5.760729	2.229088	1.420200	0.000000
21	H	6.784457	6.308499	2.684437	2.230135	3.345859
22	H	5.710071	5.847181	2.680538	2.230531	1.079135
23	C	5.741066	6.227554	3.324217	2.281280	1.411485
24	H	5.889725	6.725684	4.338092	3.327697	2.227552
25	C	6.128092	6.384986	3.322073	2.279997	2.302571
26	H	6.551188	6.968386	4.333144	3.324703	3.343710
27	C	6.277015	6.032493	2.230276	1.418765	2.303673
28	O	5.771144	4.502798	3.755346	3.908647	5.055879
29	C	4.634116	3.490587	3.652045	3.667098	4.573964
30	C	3.403396	2.725416	2.928769	2.895631	3.109915
31	O	3.977406	3.128806	2.904695	3.155489	3.271143
32	C	3.523812	3.884138	4.840847	4.236837	4.421457
33	O	4.564258	4.764221	5.422958	4.820650	5.195068
		21	22	23	24	25
21	H	0.000000				
22	H	4.352235	0.000000			
23	C	3.337299	2.231797	0.000000		
24	H	4.342945	2.697019	1.079497	0.000000	
25	C	2.213867	3.349693	1.433363	2.240837	0.000000
26	H	2.671096	4.358510	2.243072	2.693987	1.079437
27	C	1.080114	3.344807	2.292098	3.331688	1.399761
28	O	3.059841	5.778783	5.389216	6.344379	4.602262
29	C	3.294761	5.224821	4.880230	5.749614	4.298324
30	C	4.103826	3.299071	3.805980	4.501210	4.063839
31	O	4.760806	3.120450	4.285429	4.953603	4.761300
32	C	3.980194	5.125838	4.011081	4.460679	3.574674
33	O	3.971087	6.025438	4.653256	5.133717	3.875393
		26	27	28	29	30
26	H	0.000000				
27	C	2.215329	0.000000			
28	O	5.027259	3.598462	0.000000		
29	C	4.773287	3.514229	1.140297	0.000000	
30	C	4.885008	3.597682	3.512352	2.596087	0.000000
31	O	5.695021	4.200833	4.248998	3.486232	1.161220
32	C	3.664025	3.766412	3.600241	2.720005	3.178700
33	O	3.693186	4.034464	3.559735	2.975045	4.173885
		31	32	33		
31	O	0.000000				
32	C	4.332598	0.000000			
33	O	5.332016	1.203162	0.000000		

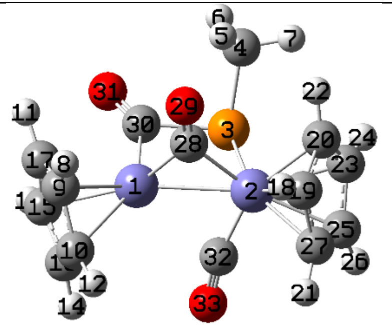

17T -3634.426458 28.45 C1
WBI 0.26

Charge and spin density

		1	2
1	Fe	0.107856	2.587163
2	Fe	-0.139311	-0.033391
3	P	0.089881	-0.063904
4	C	-0.003462	-0.005230
9	C	0.032325	0.027518
10	C	-0.046304	0.053822
13	C	0.000254	0.033465
15	C	-0.057555	-0.013528
17	C	-0.031614	-0.015381
19	C	0.066800	0.004204
20	C	0.029376	0.011394
23	C	0.057413	-0.014304
25	C	0.020079	0.003928
27	C	0.005837	0.003567
28	C	0.156775	-0.139144
29	O	-0.171172	-0.110590
30	C	0.140052	-0.175215
31	O	-0.211819	-0.131473
32	C	0.076126	-0.032647
33	O	-0.121536	0.009746

		1	2	3	4	5
1	Fe	0.000000				
2	Fe	2.525494	0.000000			
3	P	2.730110	2.321723	0.000000		
4	C	3.846035	3.364885	1.854694	0.000000	
5	H	3.714365	3.403907	2.504493	1.090964	0.000000
6	H	4.369730	4.323651	2.424740	1.092162	1.779782
7	H	4.689316	3.634296	2.430396	1.092312	1.776505
8	H	2.988715	4.585500	5.347022	5.707250	5.015161
9	C	2.267851	4.288267	4.866114	5.518492	5.006973
10	C	2.319241	4.119325	5.017759	6.067376	5.732846
11	H	2.860849	5.151815	4.674291	4.969124	4.535592
12	H	3.060573	4.277121	5.604098	6.678718	6.317523
13	C	2.261937	4.352377	4.746756	6.047437	5.936973
14	H	2.979033	4.696308	5.137826	6.645132	6.666298
15	C	2.161615	4.637354	4.390111	5.472570	5.370934
16	H	2.866105	5.227564	4.543150	5.659898	5.715467
17	C	2.161571	4.595718	4.466827	5.107216	4.737968
18	H	4.451525	2.835148	4.904894	5.154741	4.677449
19	C	4.202020	2.094095	4.179310	4.579946	4.313343
20	C	4.323578	2.088188	3.456310	3.492497	3.347865
21	H	4.670713	2.848221	5.065449	6.102354	6.059155
22	H	4.675933	2.833620	3.703475	3.175301	2.827863
23	C	4.525945	2.102608	3.075836	3.535690	3.817085
24	H	5.026898	2.854273	2.996323	3.260847	3.789723
25	C	4.513950	2.100247	3.640306	4.609365	4.882112
26	H	5.013740	2.856712	4.030651	5.222983	5.654827
27	C	4.315074	2.094557	4.270881	5.149606	5.132058
28	C	1.943284	1.915026	2.877866	3.238785	2.722270
29	O	2.929877	2.899950	3.732916	3.536222	2.701038
30	C	1.921139	3.311048	1.882375	2.746889	2.950013
31	O	2.920216	4.477184	2.784291	3.209107	3.427542
32	C	2.357339	1.754884	2.944774	4.554143	4.754091
33	O	3.075775	2.889265	3.798793	5.539238	5.819657

		6	7	8	9	10
6	H	0.000000				
7	H	1.771485	0.000000			
8	H	6.214506	6.549949	0.000000		
9	C	5.969244	6.417697	1.079383	0.000000	
10	C	6.605407	6.883871	2.227834	1.414064	0.000000
11	H	5.008140	6.031574	2.686867	2.231409	3.334291
12	H	7.332880	7.390749	2.683666	2.226710	1.080129
13	C	6.437384	6.910152	3.331488	2.288571	1.410732
14	H	7.044245	7.438131	4.340717	3.331774	2.223668
15	C	5.653160	6.452912	3.339149	2.295605	2.288360
16	H	5.661627	6.654329	4.346317	3.336642	3.333264

17	C	5.318720	6.122620	2.222667	1.412681	2.289214
18	H	6.211554	5.202242	4.737035	5.024744	5.007660
19	C	5.660695	4.529270	5.163868	5.257128	5.144662
20	C	4.582391	3.234815	5.656367	5.709627	5.807063
21	H	7.114569	6.124699	5.907178	5.741843	5.063846
22	H	4.223520	2.855543	5.709370	5.892533	6.234016
23	C	4.543971	3.137802	6.479032	6.313442	6.212606
24	H	4.139781	2.636404	7.181873	6.966226	6.938697
25	C	5.585087	4.396123	6.531116	6.261008	5.849391
26	H	6.100854	4.997689	7.273307	6.880227	6.316645
27	C	6.187606	5.095236	5.754594	5.620531	5.164151
28	C	4.077448	3.861231	2.968476	2.935491	3.356668
29	O	4.335176	4.099876	2.886537	3.245566	3.981991
30	C	2.856750	3.742187	4.321043	3.689458	4.080566
31	O	2.899522	4.246564	4.873850	4.252727	4.812661
32	C	5.302888	4.971983	4.807108	4.180367	3.506950
33	O	6.193214	5.962420	5.462853	4.708690	3.763146
		11	12	13	14	15
11	H	0.000000				
12	H	4.344576	0.000000			
13	C	3.336562	2.222788	0.000000		
14	H	4.347209	2.676771	1.079999	0.000000	
15	C	2.237786	3.331061	1.413395	2.224643	0.000000
16	H	2.688007	4.343073	2.232368	2.689645	1.079077
17	C	1.078833	3.332157	2.295648	3.340052	1.430255
18	H	6.445177	4.697081	5.911456	6.387819	6.419347
19	C	6.496767	4.938861	5.817848	6.197893	6.287525
20	C	6.480673	5.836777	6.293591	6.727406	6.484537
21	H	7.329033	4.576244	5.561953	5.568382	6.440119
22	H	6.426091	6.355954	6.777310	7.336784	6.789175
23	C	6.961634	6.286817	6.415452	6.663853	6.624357
24	H	7.319410	7.126996	6.992187	7.227138	7.041331
25	C	7.236231	5.756857	6.013891	6.071926	6.499122
26	H	7.814377	6.223559	6.286651	6.167811	6.824801
27	C	6.962063	4.870624	5.623845	5.757989	6.288338
28	C	3.726066	3.716530	3.942018	4.682654	3.937305
29	O	3.917731	4.289725	4.775151	5.620271	4.666027
30	C	2.962128	4.942366	3.649673	4.255779	2.851986
31	O	2.869772	5.777852	4.260037	4.885855	3.143105
32	C	5.205620	3.641107	3.340391	3.332929	3.942293
33	O	5.770299	3.823557	3.315061	2.935479	4.115833
		16	17	18	19	20
16	H	0.000000				
17	C	2.238222	0.000000			
18	H	7.284986	5.925078	0.000000		
19	C	7.047096	5.967077	1.079056	0.000000	

20	C	7.100410	6.133247	2.230922	1.422623	0.000000
21	H	7.174586	6.534699	2.682141	2.222926	3.336414
22	H	7.392416	6.247787	2.681936	2.231975	1.078733
23	C	7.084656	6.558479	3.336607	2.295078	1.418193
24	H	7.363577	7.020821	4.347929	3.339890	2.232255
25	C	6.999321	6.639293	3.341723	2.296961	2.293186
26	H	7.216912	7.169781	4.351134	3.339207	3.336519
27	C	6.976924	6.282725	2.223577	1.408672	2.292211
28	C	4.702118	3.339060	2.893733	2.800634	2.848719
29	O	5.463715	3.752099	2.905288	3.123953	3.117495
30	C	2.917637	2.880122	5.631352	5.191687	4.768216
31	O	2.894121	3.134276	6.710427	6.310508	5.797906
32	C	4.448181	4.411243	4.051628	3.426702	3.761776
33	O	4.500991	4.888841	4.999301	4.401673	4.828992
		21	22	23	24	25
21	H	0.000000				
22	H	4.342987	0.000000			
23	C	3.335874	2.234370	0.000000		
24	H	4.345007	2.695619	1.079770	0.000000	
25	C	2.240668	3.336204	1.409851	2.218283	0.000000
26	H	2.699226	4.346441	2.224207	2.673138	1.079169
27	C	1.079366	3.332717	2.293781	3.337037	1.430330
28	C	4.100090	2.991549	3.579191	4.227539	3.913905
29	O	4.783065	2.902822	4.170830	4.797168	4.696719
30	C	5.991891	4.885309	4.739688	4.830820	5.123634
31	O	7.174662	5.784098	5.773782	5.736194	6.248117
32	C	2.967165	4.583836	3.414464	4.035578	2.762169
33	O	3.419002	5.700012	4.331922	4.883958	3.454258
		26	27	28	29	30
26	H	0.000000				
27	C	2.242901	0.000000			
28	C	4.760002	3.495634	0.000000		
29	O	5.645099	4.158294	1.169199	0.000000	
30	C	5.533534	5.390106	2.900240	3.666717	0.000000
31	O	6.621310	6.563992	3.899728	4.475363	1.183078
32	C	2.950520	2.772965	2.997828	4.140031	3.457199
33	O	3.312028	3.510670	4.070999	5.212238	4.138902
		31	32	33		
31	O	0.000000				
32	C	4.587670	0.000000			
33	O	5.163084	1.151215	0.000000		

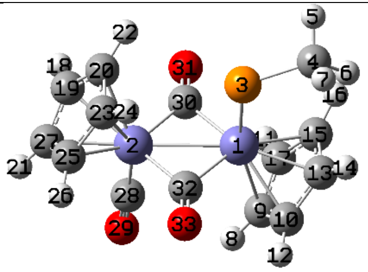

18S -3634.424434 29.72 Cs
WBI 0.28

Charge and spin density

		1
1	Fe	-0.210893
2	Fe	0.020124
3	P	-0.012577
4	C	-0.036804
9	C	0.045790
10	C	0.062874
13	C	-0.017747
15	C	-0.075132
17	C	0.061021
19	C	0.054089
20	C	0.060485
23	C	0.068571
25	C	0.061061
27	C	0.023563
28	C	-0.025303
29	O	-0.106410
30	C	0.186340
31	O	-0.178905
32	C	0.202266
33	O	-0.182414

		1	2	3	4	5
1	Fe	0.000000				
2	Fe	2.552419	0.000000			
3	P	2.084650	3.362676	0.000000		
4	C	3.311764	5.162251	1.847779	0.000000	
5	H	4.120121	5.704701	2.421255	1.094140	0.000000
6	H	3.367588	5.578453	2.541497	1.090135	1.780745
7	H	3.885244	5.654482	2.407888	1.097096	1.750130
8	H	2.928732	3.781450	5.008373	6.029511	6.906368
9	C	2.164149	3.730963	4.202078	5.040462	5.932152
10	C	2.111532	4.103461	3.768329	4.358610	5.378589
11	H	2.876224	4.237193	4.508940	5.201078	5.783567
12	H	2.848098	4.444469	4.296058	4.913867	5.979509
13	C	2.068317	4.561443	3.120760	3.202556	4.207810
14	H	2.812441	5.248358	3.205776	2.795683	3.844980
15	C	2.076838	4.492071	3.212904	3.344739	4.113981
16	H	2.816773	5.123890	3.363290	3.087858	3.637032
17	C	2.128914	3.982745	3.895247	4.530244	5.263886
18	H	4.871870	2.844085	5.158087	6.847856	6.990394
19	C	4.364593	2.104397	4.556016	6.338675	6.564631
20	C	3.855438	2.122908	3.467289	5.215295	5.379815
21	H	5.331252	2.822862	6.055696	7.895987	8.330607
22	H	3.962950	2.864053	3.154818	4.706891	4.680799
23	C	3.840877	2.123702	3.456499	5.228280	5.569942
24	H	3.936343	2.865344	3.132264	4.733744	5.082120
25	C	4.345451	2.107436	4.543949	6.357366	6.819227
26	H	4.837513	2.846985	5.136435	6.878978	7.435509
27	C	4.632731	2.083292	5.100203	6.946585	7.338790
28	C	3.200464	1.750807	4.725531	6.367730	7.027503
29	O	4.034848	2.893762	5.754948	7.307847	8.009220
30	C	1.941566	1.891383	2.867848	4.360927	4.792550
31	O	2.907864	2.880127	3.594216	4.844290	5.082244
32	C	1.921713	1.908531	2.882190	4.428688	5.246802
33	O	2.902434	2.881914	3.637940	4.987729	5.870554

		6	7	8	9	10
6	H	0.000000				
7	H	1.776559	0.000000			
8	H	5.761968	6.512649	0.000000		
9	C	4.708550	5.544953	1.078821	0.000000	
10	C	4.049382	4.625639	2.226562	1.414533	0.000000
11	H	4.759421	6.046601	2.684332	2.226500	3.329311
12	H	4.713916	4.962388	2.683280	2.227498	1.079058
13	C	2.708584	3.556954	3.336572	2.294356	1.417028
14	H	2.280477	2.880169	4.342119	3.334346	2.225228
15	C	2.732825	4.066856	3.336876	2.294179	2.292855
16	H	2.329354	3.955060	4.342995	3.334683	3.338104

	17	C	4.077576	5.261191	2.225828	1.412543	2.285790
	18	H	7.327020	7.541090	5.934584	5.986059	6.675656
	19	C	6.900943	6.914304	5.624418	5.640042	6.146440
	20	C	5.915350	5.718538	5.799594	5.572310	5.787972
	21	H	8.382052	8.334013	5.583204	5.977885	6.557840
	22	H	5.439363	5.299970	6.238041	5.854837	6.026829
	23	C	5.993108	5.480236	5.720836	5.524325	5.481304
	24	H	5.597256	4.802238	6.099837	5.768806	5.460420
	25	C	7.010888	6.593515	5.493329	5.564552	5.671755
	26	H	7.520014	6.975352	5.695767	5.848923	5.825343
	27	C	7.501938	7.373159	5.425015	5.628858	6.071959
	28	C	6.546200	6.885883	2.798551	3.237816	4.029594
	29	O	7.375173	7.830060	2.688100	3.453503	4.427608
	30	C	4.548613	5.154545	3.586178	3.214723	3.824355
	31	O	4.942930	5.773377	4.260407	3.911246	4.689208
	32	C	4.783837	4.639276	3.325490	3.057187	2.789565
	33	O	5.366655	4.951709	3.860061	3.688349	3.118313
			11	12	13	14	15
	11	H	0.000000				
	12	H	4.338880	0.000000			
	13	C	3.334254	2.226222	0.000000		
	14	H	4.346145	2.675231	1.078181	0.000000	
	15	C	2.225473	3.334846	1.420843	2.237390	0.000000
	16	H	2.674172	4.347484	2.238340	2.706046	1.078874
	17	C	1.079031	3.328644	2.292650	3.337234	1.417738
	18	H	5.517865	7.187452	6.913642	7.629257	6.387105
	19	C	5.550688	6.544756	6.432649	7.076232	6.114284
	20	C	5.603010	6.177267	5.845554	6.314160	5.647842
	21	H	6.382226	6.696753	7.271949	7.995372	7.174757
	22	H	5.607737	6.531746	5.829806	6.220846	5.500614
	23	C	6.092903	5.630984	5.691705	6.048951	5.840534
	24	H	6.499239	5.524259	5.535783	5.703011	5.867455
	25	C	6.338003	5.687084	6.205591	6.689778	6.404330
	26	H	6.936562	5.639479	6.507445	6.936918	6.901572
	27	C	6.014652	6.256735	6.630874	7.279366	6.552752
	28	C	3.895090	4.323190	4.883874	5.769061	4.761470
	29	O	4.127203	4.663319	5.447799	6.397861	5.302161
	30	C	2.794771	4.575758	3.847970	4.637110	3.237269
	31	O	2.757126	5.547836	4.556473	5.355746	3.625428
	32	C	4.414451	2.834077	3.380359	3.890209	3.892268
	33	O	5.379084	2.735527	3.858042	4.176667	4.677690
			16	17	18	19	20
	16	H	0.000000				
	17	C	2.226623	0.000000			
	18	H	6.687891	5.774855	0.000000		
	19	C	6.505754	5.603974	1.078795	0.000000	

20	C	5.949702	5.468093	2.231668	1.417085	0.000000
21	H	7.817519	6.386958	2.688790	2.229393	3.329498
22	H	5.608340	5.511737	2.693356	2.231852	1.079044
23	C	6.302129	5.732410	3.337864	2.295079	1.414202
24	H	6.291731	5.996413	4.342554	3.334621	2.220406
25	C	7.027483	6.020944	3.347403	2.304824	2.295348
26	H	7.618830	6.518172	4.356778	3.347485	3.337983
27	C	7.133579	5.938129	2.234545	1.419424	2.285671
28	C	5.564705	3.789299	3.527598	3.136585	3.721392
29	O	6.156286	4.134850	4.285554	4.059162	4.810827
30	C	3.632133	2.773254	3.151934	2.947939	2.809662
31	O	3.755914	3.124097	3.109111	3.288559	3.322481
32	C	4.706876	3.714337	4.732694	3.912620	3.469692
33	O	5.540741	4.583024	5.662961	4.755896	4.294680
		21	22	23	24	25
21	H	0.000000				
22	H	4.341944	0.000000			
23	C	3.329241	2.220712	0.000000		
24	H	4.341791	2.663354	1.079067	0.000000	
25	C	2.228665	3.335030	1.417359	2.232451	0.000000
26	H	2.688133	4.342829	2.231679	2.693786	1.078773
27	C	1.080109	3.330704	2.285340	3.330434	1.418553
28	C	2.798346	4.473468	3.728535	4.484956	3.152034
29	O	3.277065	5.565182	4.821099	5.582237	4.080480
30	C	4.297290	2.892609	3.447288	3.985184	3.899229
31	O	4.828038	3.168316	4.281477	4.875699	4.765585
32	C	4.289074	4.016060	2.818984	2.902258	2.941246
33	O	4.777562	4.907162	3.324487	3.187449	3.253098
		26	27	28	29	30
26	H	0.000000				
27	C	2.233789	0.000000			
28	C	3.552120	2.715958	0.000000		
29	O	4.322344	3.524775	1.143854	0.000000	
30	C	4.715334	3.627514	2.643149	3.556927	0.000000
31	O	5.669275	4.241135	3.410755	4.152664	1.167957
32	C	3.130587	3.625656	2.660815	3.576148	2.857451
33	O	3.050407	4.205045	3.411973	4.158351	4.024780
		31	32	33		
31	O	0.000000				
32	C	4.025213	0.000000			
33	O	5.192458	1.167798	0.000000		

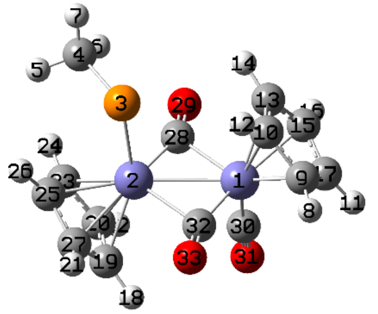

19T -3634.421678 31.45 C1
WBI 0.29

Charge and spin density

		1	2
1	Fe	0.068022	-0.205335
2	Fe	-0.047559	1.422316
3	P	-0.140306	0.887259
4	C	-0.008333	0.018783
9	C	0.058523	-0.002959
10	C	0.089244	-0.009998
13	C	0.042737	0.003916
15	C	0.042139	0.005599
17	C	0.005654	0.008767
19	C	0.005710	-0.012631
20	C	0.031236	-0.007197
23	C	0.015064	-0.002673
25	C	0.043746	-0.045321
27	C	0.021165	0.060140
28	C	0.142374	-0.065246
29	O	-0.186516	-0.044653
30	C	0.000727	0.005092
31	O	-0.093992	0.001552
32	C	0.055069	-0.024712
33	O	-0.144705	0.007303

		1	2	3	4	5
1	Fe	0.000000				
2	Fe	2.590603	0.000000			
3	P	3.612970	2.227735	0.000000		
4	C	4.967379	3.335375	1.846178	0.000000	
5	H	5.606724	3.552282	2.471205	1.093850	0.000000
6	H	4.825766	3.458138	2.458618	1.093619	1.773835
7	H	5.689759	4.299940	2.432630	1.092344	1.767795
8	H	2.835393	4.880103	5.372352	7.063967	7.761225
9	C	2.095622	4.375596	4.770796	6.337889	7.107640
10	C	2.110526	3.834101	3.634259	5.153904	6.003639
11	H	2.824109	5.378718	6.292306	7.586722	8.330871
12	H	2.858658	3.926535	3.282409	4.911330	5.728875
13	C	2.129348	3.850615	3.624155	4.734224	5.674015
14	H	2.872484	3.937850	3.245122	4.020946	5.039180
15	C	2.102769	4.368342	4.731290	5.753199	6.623123
16	H	2.851460	4.877726	5.314770	6.052973	6.913040
17	C	2.081840	4.667336	5.325919	6.644073	7.434585
18	H	3.946810	2.933292	5.082209	6.110010	6.030533
19	C	3.871692	2.182079	4.271723	5.142836	4.988749
20	C	3.999621	2.167157	4.129240	4.541200	4.366176
21	H	4.762964	2.906167	4.112953	5.256644	5.000462
22	H	4.177802	2.908112	4.848194	5.122348	5.028505
23	C	4.486593	2.104969	3.373639	3.419225	3.051551
24	H	5.041534	2.829138	3.580548	3.055837	2.607054
25	C	4.679068	2.108010	3.039365	3.487396	3.036401
26	H	5.385668	2.854093	2.993902	3.222737	2.590505
27	C	4.341045	2.174481	3.691448	4.626681	4.351977
28	C	2.117921	1.789006	2.664346	3.329158	3.903792
29	O	2.957617	2.868971	3.359936	3.480261	4.114876
30	C	1.757288	3.183576	4.886346	5.973565	6.431079
31	O	2.899176	3.981168	5.867692	6.816997	7.176857
32	C	1.781606	2.202628	3.523740	5.148636	5.543742
33	O	2.882791	2.988698	4.167940	5.870239	6.156332

		6	7	8	9	10
6	H	0.000000				
7	H	1.776082	0.000000			
8	H	7.173747	7.541733	0.000000		
9	C	6.340256	6.811502	1.079218	0.000000	
10	C	5.226014	5.510111	2.236963	1.424262	0.000000
11	H	7.300438	8.186348	2.679876	2.223055	3.334469
12	H	5.199834	5.146791	2.700869	2.240323	1.079288
13	C	4.530605	5.100289	3.339140	2.296350	1.414160
14	H	3.757549	4.264528	4.345340	3.336927	2.220705
15	C	5.372670	6.246217	3.344463	2.300474	2.291988
16	H	5.488447	6.559269	4.353816	3.342890	3.334415

17	C	6.411552	7.189071	2.224915	1.410843	2.290128
18	H	6.164583	7.128224	5.618538	5.518995	5.703729
19	C	5.235129	6.175778	5.852210	5.618431	5.530084
20	C	4.395223	5.629137	6.462316	6.003622	5.751059
21	H	5.778911	6.089483	5.979048	5.984654	5.725934
22	H	4.757060	6.206007	6.763975	6.236153	6.098569
23	C	3.416438	4.494179	6.949035	6.426991	5.865622
24	H	2.858893	4.095426	7.636638	7.003757	6.315365
25	C	3.902177	4.443287	6.713140	6.352953	5.745945
26	H	3.880332	4.014018	7.252347	6.901782	6.133896
27	C	5.005925	5.566714	6.049121	5.876793	5.555249
28	C	2.939263	4.205428	4.863816	4.040263	3.431567
29	O	2.746285	4.266285	5.670017	4.735847	4.094300
30	C	5.657246	6.854214	3.873813	3.353458	3.802194
31	O	6.426217	7.753580	4.761117	4.344712	4.920408
32	C	5.327854	5.919447	2.957384	2.807775	2.875299
33	O	6.201565	6.597513	3.155248	3.379815	3.583236
		11	12	13	14	15
11	H	0.000000				
12	H	4.346304	0.000000			
13	C	3.334668	2.222646	0.000000		
14	H	4.346250	2.667126	1.078673	0.000000	
15	C	2.239562	3.332153	1.411426	2.224508	0.000000
16	H	2.700380	4.339634	2.225096	2.682496	1.078899
17	C	1.079736	3.334539	2.291692	3.336071	1.428681
18	H	5.877573	5.962207	5.964551	6.408553	5.924355
19	C	6.195191	5.694570	5.689133	5.964430	5.841827
20	C	6.382527	6.014191	5.561515	5.648488	5.674672
21	H	7.196528	5.483221	6.292583	6.534056	6.820312
22	H	6.243494	6.524188	5.726876	5.835547	5.604186
23	C	7.139617	5.936623	5.640399	5.489627	6.057823
24	H	7.654716	6.400483	5.885352	5.554370	6.333143
25	C	7.433443	5.584777	5.840227	5.747305	6.459740
26	H	8.189007	5.802459	6.272269	6.057036	7.070142
27	C	6.901390	5.447570	5.885651	6.048700	6.348839
28	C	4.618726	3.878922	2.807623	2.755585	3.129613
29	O	4.997594	4.627185	3.102293	2.800773	3.260453
30	C	2.919311	4.594600	3.620790	4.296599	2.989294
31	O	3.463417	5.726998	4.687667	5.333565	3.887040
32	C	4.009184	3.083126	3.531801	4.162878	3.815436
33	O	4.702466	3.561368	4.498049	5.148452	4.823360
		16	17	18	19	20
16	H	0.000000				
17	C	2.242801	0.000000			
18	H	6.360457	5.653040	0.000000		
19	C	6.262131	5.802431	1.080197	0.000000	

20	C	5.883515	5.951970	2.228560	1.417891	0.000000
21	H	7.492402	6.647418	2.675130	2.224429	3.340343
22	H	5.630791	5.936966	2.685589	2.231860	1.079416
23	C	6.301362	6.529795	3.327597	2.284677	1.410550
24	H	6.445031	7.008091	4.337625	3.328438	2.223106
25	C	6.913984	6.756075	3.325640	2.282641	2.296667
26	H	7.563643	7.427425	4.334963	3.325745	3.338806
27	C	6.902839	6.346016	2.222902	1.412663	2.298759
28	C	3.371926	3.880342	4.072746	3.507946	2.936247
29	O	3.144354	4.313847	4.993196	4.421478	3.560128
30	C	3.267127	2.792414	3.287670	3.520089	3.556375
31	O	3.947074	3.635475	3.341020	3.770092	3.746373
32	C	4.629958	3.421467	2.953720	2.957004	3.676557
33	O	5.694911	4.217527	3.073511	3.240493	4.288117
		21	22	23	24	25
21	H	0.000000				
22	H	4.348225	0.000000			
23	C	3.343934	2.224182	0.000000		
24	H	4.353960	2.679682	1.079126	0.000000	
25	C	2.228014	3.341401	1.430000	2.242561	0.000000
26	H	2.684959	4.350749	2.242176	2.702179	1.079017
27	C	1.079250	3.341087	2.299136	3.341765	1.413014
28	C	4.681913	3.095161	2.992758	3.208859	3.611405
29	O	5.751855	3.446751	3.536483	3.412732	4.416822
30	C	5.066732	3.360144	4.524993	5.122890	5.024106
31	O	5.579864	3.291959	4.932818	5.519391	5.571390
32	C	3.247643	4.211875	4.152906	4.991000	3.861955
33	O	2.899604	4.935724	4.748997	5.687888	4.163908
		26	27	28	29	30
26	H	0.000000				
27	C	2.224939	0.000000			
28	C	4.266728	3.899345	0.000000		
29	O	5.008111	4.893696	1.168824	0.000000	
30	C	5.953165	4.507080	2.725173	3.329606	0.000000
31	O	6.579395	4.981728	3.576910	4.010479	1.142255
32	C	4.548742	3.126608	2.998793	4.122229	2.586175
33	O	4.760851	3.163734	4.099141	5.244781	3.515908
		31	32	33		
31	O	0.000000				
32	C	3.508415	0.000000			
33	O	4.279517	1.158606	0.000000		

Table S5. Distance matrix for the lowest energy $Cp_2Fe_2PMe(CO)_4$ structures as optimized at the PBE0/def2TZVP level. Included are the ranking order, spin multiplicity (S, T or Q), total energy (in a.u.), relative energy (in kcal/mol), symmetry point group Wiberg bond indices for the Fe-Fe bonds, Mulliken charges and the spin density (Cp moieties are omitted for clarity).

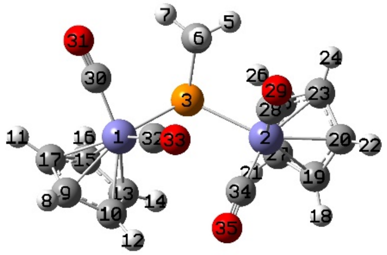

1S -3747.724626 0.00 C1
WBI 0.02

Charge and spin density

		1
1	Fe	0.096099
2	Fe	0.130138
3	P	-0.043753
4	C	-0.019618
9	C	0.025148
10	C	0.023548
13	C	0.025502
15	C	0.080149
17	C	-0.010161
19	C	-0.003001
20	C	0.008418
23	C	0.021031
25	C	0.087333
27	C	0.024275
28	C	-0.013053
29	O	-0.088399
30	C	-0.006946
31	O	-0.097269
32	C	-0.006363
33	O	-0.093684
34	C	-0.017192
35	O	-0.122202

		1	2	3	4	5
1	Fe	0.000000				
2	Fe	4.074022	0.000000			
3	P	2.345354	2.374990	0.000000		
4	C	3.403674	3.339243	1.865502	0.000000	
5	H	4.378669	3.288447	2.440263	1.093442	0.000000
6	H	3.556852	3.646926	2.541015	1.092665	1.766051
7	H	3.584719	4.319952	2.446964	1.092504	1.763729
8	H	2.859391	6.496719	5.175609	6.144976	7.169671
9	C	2.108704	5.734526	4.339012	5.505548	6.486479
10	C	2.108482	4.726392	3.809214	5.289808	6.136585
11	H	2.847510	6.745761	4.727379	5.561402	6.553284
12	H	2.856529	4.710886	4.311080	5.771974	6.557383
13	C	2.112365	4.208638	3.100189	4.802037	5.532980
14	H	2.852424	3.652906	3.036618	4.883461	5.431244
15	C	2.113015	5.029679	3.290611	4.719848	5.529829
16	H	2.853164	5.265710	3.397527	4.731143	5.427532
17	C	2.103828	5.884578	4.062101	5.171561	6.135985
18	H	6.218016	2.843816	4.897849	6.138482	5.962164
19	C	5.801848	2.098993	4.165649	5.264432	4.989058
20	C	6.172045	2.100595	4.312231	4.913906	4.440196
21	H	5.129528	2.872030	3.617304	5.117645	5.016865
22	H	6.861740	2.847419	5.132002	5.557788	5.069269
23	C	5.847817	2.107764	3.674637	3.966929	3.302283
24	H	6.309671	2.862511	4.094625	3.878137	3.012091
25	C	5.247025	2.124040	3.043434	3.781139	3.288105
26	H	5.172501	2.860618	2.912337	3.472299	2.953289
27	C	5.202045	2.113818	3.399340	4.649328	4.404297
28	C	4.257239	1.746163	2.894532	2.966374	2.970830
29	O	4.788260	2.887347	3.700160	3.275741	3.295372
30	C	1.746141	5.029449	2.900044	2.989573	4.066411
31	O	2.887949	5.897932	3.728653	3.303007	4.319188
32	C	1.745288	3.827137	2.868750	3.415619	4.337212
33	O	2.888270	4.111574	3.671136	3.900871	4.705898
34	C	3.623736	1.742297	2.998600	4.321896	4.629358
35	O	3.844067	2.885399	3.858233	5.236382	5.664530

		6	7	8	9	10
6	H	0.000000				
7	H	1.769954	0.000000			
8	H	6.053410	6.216376	0.000000		

9	C	5.578453	5.591287	1.078900	0.000000	
10	C	5.433001	5.611340	2.236484	1.422083	0.000000
11	H	5.835533	5.214629	2.682537	2.224671	3.331040
12	H	5.793983	6.248315	2.695610	2.234300	1.078508
13	C	5.222556	5.068826	3.340140	2.297040	1.414454
14	H	5.400094	5.283943	4.349510	3.339634	2.226603
15	C	5.238702	4.671605	3.344755	2.301871	2.294228
16	H	5.430446	4.540051	4.353522	3.343941	3.336070
17	C	5.458805	5.026448	2.229131	1.414297	2.286904
18	H	6.487430	7.057918	8.092230	7.307048	5.978235
19	C	5.685489	6.175133	7.978879	7.151754	5.921730
20	C	5.207363	5.900726	8.527318	7.767682	6.653977
21	H	5.853656	5.727973	7.311841	6.320380	5.189118
22	H	5.673786	6.594917	9.093860	8.417627	7.296857
23	C	4.429759	4.861474	8.476286	7.668827	6.725848
24	H	4.252190	4.733517	9.011117	8.252180	7.427626
25	C	4.515222	4.502098	7.902021	6.989492	6.061728
26	H	4.384526	3.968003	7.921655	6.977469	6.214781
27	C	5.306255	5.396265	7.562434	6.628632	5.506268
28	C	2.739965	4.052340	6.575645	6.029649	5.262624
29	O	2.684470	4.301462	6.930886	6.531229	5.922904
30	C	3.059264	2.698201	3.672331	3.218215	3.743238
31	O	3.303599	2.696784	4.462187	4.154066	4.840346
32	C	3.058234	3.919168	3.355052	3.054361	2.875208
33	O	3.264961	4.525967	4.047821	3.962344	3.742207
34	C	4.419651	5.224120	5.428363	4.762468	3.567632
35	O	5.237541	6.076664	5.021236	4.473534	3.195389
		11	12	13	14	15
11	H	0.000000				
12	H	4.341394	0.000000			
13	C	3.330650	2.224126	0.000000		
14	H	4.339457	2.678623	1.078831	0.000000	
15	C	2.229968	3.335143	1.416264	2.225489	0.000000
16	H	2.689892	4.342746	2.229023	2.681164	1.078429
17	C	1.080013	3.330234	2.287288	3.329894	1.420159
18	H	8.613012	5.657329	5.440636	4.510823	6.581573
19	C	8.251782	5.758713	5.258417	4.365963	6.245029
20	C	8.797675	6.499332	6.084640	5.329603	6.960607
21	H	7.019771	5.364670	4.102536	3.104980	4.843818
22	H	9.583452	7.019817	6.884626	6.171637	7.826020
23	C	8.358057	6.788893	5.999931	5.335904	6.611092
24	H	8.815758	7.527133	6.749354	6.187916	7.237318
25	C	7.493053	6.282675	5.111051	4.389339	5.613380
26	H	7.153926	6.607974	5.145493	4.534842	5.373206
27	C	7.405034	5.600194	4.564207	3.653232	5.332708
28	C	7.074051	5.175271	5.106681	4.856806	5.802243

29	O	7.558637	5.799137	5.944960	5.827779	6.559175
30	C	2.862273	4.515993	3.677495	4.401834	3.093401
31	O	3.347270	5.620378	4.763419	5.483689	4.008790
32	C	4.290200	3.035701	3.382873	3.891802	3.804832
33	O	5.322531	3.624143	4.395931	4.806602	4.932181
34	C	6.260102	3.252641	3.438934	2.969982	4.599620
35	O	6.274371	2.571776	3.423769	3.074075	4.734480
		16	17	18	19	20
16	H	0.000000				
17	C	2.235337	0.000000			
18	H	6.808134	7.612750	0.000000		
19	C	6.370693	7.312060	1.079847	0.000000	
20	C	7.076457	7.924917	2.221080	1.410577	0.000000
21	H	4.715653	6.141139	2.697296	2.239910	3.342687
22	H	8.021380	8.699395	2.678309	2.225558	1.079045
23	C	6.565399	7.600534	3.333364	2.289039	1.426964
24	H	7.159143	8.141287	4.342883	3.331962	2.237905
25	C	5.432149	6.747642	3.333751	2.290375	2.297798
26	H	5.014449	6.525608	4.341678	3.332259	3.341263
27	C	5.271239	6.532956	2.235414	1.424546	2.300222
28	C	6.161814	6.322061	4.190149	3.545588	2.947150
29	O	6.965002	6.891355	5.185168	4.586646	3.809412
30	C	3.447623	2.756673	7.536464	6.946036	7.062520
31	O	4.187366	3.565179	8.517267	7.855376	7.851361
32	C	4.569419	3.622883	6.093247	5.726616	5.827903
33	O	5.706136	4.693826	6.301596	5.986574	5.909089
34	C	5.147512	5.272977	2.915456	2.790409	3.285804
35	O	5.449653	5.240976	3.414246	3.605205	4.238156
		21	22	23	24	25
21	H	0.000000				
22	H	4.353372	0.000000			
23	C	3.333461	2.240578	0.000000		
24	H	4.342265	2.697642	1.079463	0.000000	
25	C	2.226791	3.340539	1.411854	2.225050	0.000000
26	H	2.675371	4.350988	2.225169	2.682199	1.078917
27	C	1.078651	3.343897	2.291691	3.334519	1.414429
28	C	4.609701	3.193321	2.854764	3.036250	3.431999
29	O	5.748388	3.810403	3.691918	3.590326	4.438035
30	C	6.275247	7.738624	6.444729	6.649182	5.914859
31	O	7.186607	8.509116	7.098528	7.157725	6.616620
32	C	5.736512	6.274711	5.687293	6.033546	5.506450
33	O	6.387503	6.163995	5.899945	6.154814	5.982683
34	C	3.396448	3.773794	3.770254	4.555759	3.654109
35	O	4.114017	4.595880	4.876232	5.672839	4.735506
		26	27	28	29	30
26	H	0.000000				

27	C	2.221763	0.000000			
28	C	3.986335	3.806880	0.000000		
29	O	4.913616	4.925228	1.142277	0.000000	
30	C	5.598051	6.242805	4.847935	5.116483	0.000000
31	O	6.181930	7.113094	5.555961	5.673837	1.143037
32	C	5.667636	5.521458	3.370922	3.598288	2.529174
33	O	6.267673	6.027772	3.232767	3.149322	3.448672
34	C	4.345951	3.056476	2.537172	3.478097	5.016870
35	O	5.415574	3.964376	3.467769	4.238494	5.406413

		31	32	33	34	35
31	O	0.000000				
32	C	3.462596	0.000000			
33	O	4.204937	1.143286	0.000000		
34	C	6.056013	3.224160	3.469756	0.000000	
35	O	6.497088	3.377660	3.546334	1.145025	0.000000

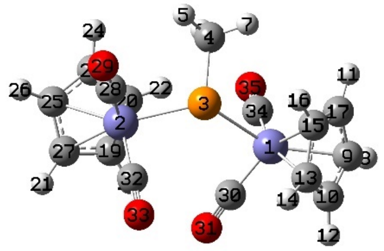

2S -3747.722848 1.12 C1
WBI 0.02

Charge and spin density

		1
1	Fe	0.140329
2	Fe	0.068425
3	P	-0.028006
4	C	-0.035473
9	C	0.008899
10	C	-0.000926
13	C	0.027995
15	C	0.114332
17	C	0.011808
19	C	0.034342
20	C	0.017875
23	C	0.042146
25	C	0.019133
27	C	0.006650
28	C	0.000494
29	O	-0.093402
30	C	-0.016729
31	O	-0.112639

		1	2	3	4	5
1	Fe	0.000000				
2	Fe	4.088512	0.000000			
3	P	2.364956	2.363872	0.000000		
4	C	3.388879	3.297421	1.862991	0.000000	
5	H	4.363121	3.397071	2.433729	1.093658	0.000000
6	H	3.666877	3.452731	2.542639	1.092430	1.770799
7	H	3.413797	4.302419	2.443409	1.094394	1.765014
8	H	2.843814	6.847276	5.097165	5.556052	6.614813
9	C	2.098251	6.185608	4.290413	4.931464	5.942271
10	C	2.096530	5.892512	4.185257	5.314361	6.239909
11	H	2.862454	6.239741	4.026817	3.857743	4.766137
12	H	2.841140	6.338134	4.929918	6.192079	7.118467
13	C	2.114001	5.321438	3.439577	4.721207	5.489563
14	H	2.865940	5.299367	3.681412	5.202138	5.831281
15	C	2.125533	5.293662	3.043995	3.836909	4.596707
16	H	2.858182	5.205409	2.906225	3.531054	4.080133
17	C	2.105946	5.829419	3.633176	3.977554	4.912604
18	H	4.329297	2.870690	4.140393	5.038602	5.580531
19	C	4.526748	2.111220	3.774853	4.477564	4.856276
20	C	4.311501	2.115797	3.311463	3.438203	3.800577
21	H	6.211680	2.850712	5.094451	6.038181	6.116966
22	H	3.891341	2.879133	3.305493	3.126410	3.715146
23	C	5.269819	2.099542	3.675582	3.534970	3.469582
24	H	5.705007	2.857176	3.969291	3.327752	3.043797
25	C	5.963577	2.098875	4.305965	4.619246	4.458337
26	H	6.867182	2.845369	5.014632	5.261872	4.896665
27	C	5.573667	2.103798	4.356058	5.095064	5.191712
28	C	4.953028	1.751832	2.691892	3.324914	2.914490
29	O	5.764678	2.893364	3.434647	3.816303	3.138253
30	C	1.745615	3.645442	2.974186	4.364877	5.220462

32 C 0.026523	31	O	2.887902	3.864725	3.827591	5.270903	6.047903
33 O -0.095142	32	C	4.031633	1.753395	2.802056	4.394826	4.599902
34 C -0.009995	33	O	4.410252	2.894827	3.568519	5.314031	5.551135
35 O -0.126639	34	C	1.737033	4.133881	2.849528	3.021181	4.091090
	35	O	2.882975	4.581223	3.641775	3.320590	4.329489
			6	7	8	9	10
	6	H	0.000000				
	7	H	1.768745	0.000000			
	8	H	5.612245	5.153986	0.000000		
	9	C	5.171349	4.535016	1.079236	0.000000	
	10	C	5.691536	5.101039	2.224722	1.410137	0.000000
	11	H	4.177639	3.057131	2.699064	2.239045	3.333192
	12	H	6.499471	6.078364	2.678104	2.221363	1.079871
	13	C	5.342159	4.515038	3.344695	2.300841	1.426652
	14	H	5.910049	5.124106	4.353846	3.343135	2.241459
	15	C	4.531056	3.384059	3.340619	2.297506	2.290317
	16	H	4.411121	3.025808	4.350983	3.340803	3.331837
	17	C	4.389182	3.381085	2.241080	1.427500	2.290201
	18	H	4.706246	5.889993	6.409815	6.116281	5.837068
	19	C	4.139820	5.439503	6.849140	6.469690	6.265951
	20	C	2.906816	4.419771	6.585365	6.223544	6.294113
	21	H	5.923073	7.067109	8.722079	8.217779	7.727982
	22	H	2.376663	3.951347	5.861719	5.611447	5.896138
	23	C	3.134973	4.610918	7.711637	7.248186	7.295434
	24	H	2.878401	4.348307	8.054932	7.592899	7.781204
	25	C	4.420317	5.712440	8.559569	8.030599	7.845566
	26	H	5.155682	6.346595	9.530652	8.949777	8.724957
	27	C	4.900679	6.142121	8.094227	7.608861	7.275988
	28	C	3.841421	4.239546	7.756117	6.952780	6.658837
	29	O	4.464453	4.591684	8.518609	7.659512	7.375460
	30	C	4.474858	4.744969	3.832745	3.320528	2.792494
	31	O	5.300592	5.771568	4.679314	4.285059	3.611029
	32	C	4.783869	5.198626	6.784629	6.017664	5.337413
	33	O	5.783711	6.003872	7.007354	6.202812	5.290584
	34	C	2.759039	3.148068	3.175422	2.941244	3.523226
	35	O	2.685853	3.483199	3.803005	3.818040	4.570006
			11	12	13	14	15
	11	H	0.000000				
	12	H	4.344421	0.000000			
	13	C	3.335260	2.237208	0.000000		
	14	H	4.343314	2.698402	1.078788	0.000000	
	15	C	2.226457	3.333464	1.412644	2.225979	0.000000
	16	H	2.683460	4.340842	2.219058	2.673118	1.078846
	17	C	1.079477	3.334712	2.292625	3.334697	1.412920
	18	H	6.877180	5.893029	5.936560	6.081225	6.276816
	19	C	6.899944	6.478612	6.163930	6.293303	6.317811

20	C	6.217684	6.711282	6.138313	6.433499	5.973643
21	H	8.739711	7.825304	7.414696	7.233229	7.748615
22	H	5.505811	6.369898	5.888669	6.356883	5.603133
23	C	6.950468	7.796297	6.911003	7.103704	6.619549
24	H	6.972681	8.390192	7.333485	7.588991	6.842101
25	C	7.972639	8.227687	7.388286	7.385633	7.306345
26	H	8.791596	9.126973	8.153907	8.068626	8.043558
27	C	7.943480	7.490192	6.967750	6.916695	7.137836
28	C	6.609890	7.251647	5.759139	5.609987	5.514453
29	O	7.105156	8.030890	6.332538	6.121087	5.974687
30	C	4.577003	2.923591	3.014649	3.318487	3.625569
31	O	5.701113	3.430512	3.911981	4.011270	4.698671
32	C	6.528644	5.600826	4.676024	4.345587	5.062768
33	O	6.973462	5.403470	4.603256	4.048217	5.255067
34	C	3.089305	4.149861	3.807317	4.599048	3.464380
35	O	3.683464	5.141121	4.936288	5.744568	4.492388
		16	17	18	19	20
16	H	0.000000				
17	C	2.225698	0.000000			
18	H	6.662516	6.372724	0.000000		
19	C	6.538285	6.491531	1.078386	0.000000	
20	C	6.094589	6.013511	2.228301	1.413650	0.000000
21	H	7.829759	8.221192	2.690882	2.236808	3.337422
22	H	5.812158	5.410920	2.685390	2.225000	1.077334
23	C	6.516711	6.822292	3.334522	2.291642	1.414105
24	H	6.623940	6.997285	4.344232	3.334552	2.227783
25	C	7.188710	7.697831	3.334726	2.294089	2.295897
26	H	7.815077	8.533798	4.345272	3.338388	3.338984
27	C	7.198259	7.517737	2.235770	1.425894	2.294903
28	C	5.082454	6.289702	4.618527	3.808269	3.539571
29	O	5.358641	6.849666	5.757806	4.928085	4.581624
30	C	4.297874	3.780997	3.060963	3.557954	3.886052
31	O	5.351974	4.890931	2.615276	3.360799	4.078304
32	C	5.063619	5.860259	3.235824	2.975894	3.566490
33	O	5.311549	6.177504	3.904952	3.875934	4.627957
34	C	4.028144	2.886430	3.962684	4.075175	3.500673
35	O	4.983041	3.750172	4.157014	4.204676	3.416522
		21	22	23	24	25
21	H	0.000000				
22	H	4.346620	0.000000			
23	C	3.338441	2.222237	0.000000		
24	H	4.347413	2.678791	1.079132	0.000000	
25	C	2.222071	3.337054	1.427079	2.239289	0.000000
26	H	2.677849	4.346600	2.239068	2.697077	1.079636
27	C	1.079534	3.337288	2.293926	3.336127	1.408428
28	C	4.006275	4.196016	2.916005	3.159752	2.842170

29	O	4.938136	5.189321	3.783079	3.778669	3.685662
30	C	5.044547	3.743786	5.001216	5.726566	5.369898
31	O	4.562713	4.120850	5.219495	6.089314	5.343825
32	C	3.062261	4.222752	3.808729	4.605389	3.437041
33	O	3.660020	5.236807	4.936157	5.744117	4.456777
34	C	6.145131	2.726495	4.593532	4.920443	5.577497
35	O	6.405295	2.420583	4.515589	4.732870	5.653074
		26	27	28	29	30
26	H	0.000000				
27	C	2.221829	0.000000			
28	C	3.012920	3.428143	0.000000		
29	O	3.574235	4.438338	1.142089	0.000000	
30	C	6.319243	4.607081	4.929566	5.918898	0.000000
31	O	6.284449	4.328993	5.312609	6.357182	1.144002
32	C	4.002426	2.885655	2.542734	3.477259	3.236817
33	O	4.935585	3.755642	3.458399	4.216869	3.476988
34	C	6.562195	5.322422	5.208162	6.095413	2.535267
35	O	6.650830	5.502369	5.704495	6.594349	3.470027
		31	32	33	34	35
31	O	0.000000				
32	C	3.235034	0.000000			
33	O	3.311527	1.141590	0.000000		
34	C	3.465685	4.705286	5.378112	0.000000	
35	O	4.226392	5.446636	6.230043	1.146438	0.000000

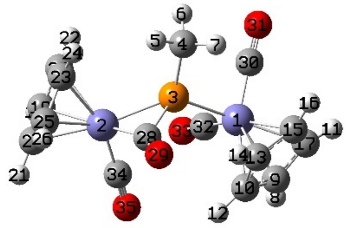

3S -3747.710595 8.80 C1
WBI 0.05

Charge and spin density

		1
1	Fe	0.005251
2	Fe	0.260628
3	P	0.094353
4	C	-0.016400
9	C	0.045807
10	C	0.063700
13	C	0.104807
15	C	0.050799
17	C	0.004937

		1	2	3	4	5
1	Fe	0.000000				
2	Fe	4.132527	0.000000			
3	P	2.286727	2.243629	0.000000		
4	C	3.434538	3.472589	1.841238	0.000000	
5	H	4.363732	3.369363	2.422538	1.090986	0.000000
6	H	3.779121	4.008487	2.458997	1.091777	1.770950
7	H	3.453125	4.317955	2.453560	1.092391	1.773025
8	H	2.836996	6.024232	4.839846	6.236304	7.092756
9	C	2.092338	5.533097	4.116885	5.385793	6.237829
10	C	2.105356	4.584445	3.397966	4.773269	5.450469
11	H	2.844448	6.959969	5.044306	5.700228	6.714440
12	H	2.862257	4.271621	3.643897	5.214425	5.742550
13	C	2.128603	4.655024	3.062633	3.957894	4.624739
14	H	2.877031	4.377562	2.994944	3.675774	4.122100
15	C	2.109427	5.599130	3.637450	4.154552	5.017442
16	H	2.869331	6.152540	4.060631	4.091738	4.940724
17	C	2.091732	6.079961	4.239881	5.059535	6.010075
18	H	6.004721	2.902672	4.567807	5.637171	5.572643
19	C	5.769510	2.139379	4.022002	4.958025	4.745068
20	C	5.494610	2.130183	3.480713	3.936408	3.619298
21	H	6.662826	2.836465	5.059076	6.230861	5.903046

19	C	0.005082
20	C	-0.004028
23	C	-0.029985
25	C	0.038354
27	C	-0.017409
28	C	0.031300
29	O	-0.268579
30	C	-0.014063
31	O	-0.081928
32	C	0.036640
33	O	-0.066430
34	C	-0.104710
35	O	-0.138124

22	H	5.469340	2.877390	3.603127	3.787009	3.610209
23	C	5.724261	2.093106	3.485948	3.686302	3.043249
24	H	5.945471	2.856326	3.661484	3.326841	2.454561
25	C	6.126663	2.080097	4.038090	4.650732	4.053079
26	H	6.628327	2.813370	4.583004	5.105492	4.408924
27	C	6.135144	2.086598	4.317270	5.317011	4.948470
28	C	3.598781	1.836470	1.887020	2.933034	2.864108
29	O	4.229378	2.928653	2.864296	3.562044	3.378703
30	C	1.753506	4.914017	2.848117	3.154181	4.234666
31	O	2.894035	5.681078	3.660781	3.486997	4.546093
32	C	1.763032	3.940354	2.841629	4.305446	5.137655
33	O	2.900254	4.242594	3.648467	5.129325	5.883229
34	C	3.866196	1.737131	2.909903	4.621028	4.789525
35	O	4.146117	2.886053	3.774392	5.573738	5.839089
		6	7	8	9	10
6	H	0.000000				
7	H	1.775213	0.000000			
8	H	6.607163	6.176289	0.000000		
9	C	5.839467	5.225554	1.079779	0.000000	
10	C	5.445495	4.656691	2.234281	1.423950	0.000000
11	H	5.898447	5.284171	2.680543	2.225493	3.342064
12	H	5.944065	5.249186	2.692553	2.237423	1.078949
13	C	4.727701	3.588434	3.337491	2.294467	1.417531
14	H	4.612046	3.278726	4.344146	3.336128	2.222214
15	C	4.693897	3.590020	3.337281	2.292401	2.291669
16	H	4.572897	3.301559	4.346764	3.335024	3.334758
17	C	5.420318	4.679507	2.222898	1.410823	2.298154
18	H	5.678614	6.657263	7.466018	7.326330	6.732652
19	C	5.119718	5.976826	7.483223	7.196572	6.443079
20	C	3.996147	5.003546	7.651390	7.208736	6.466867
21	H	6.707180	7.086606	7.870902	7.625396	6.626897
22	H	3.551010	4.878897	7.762615	7.327805	6.756518
23	C	4.039886	4.641326	7.973739	7.389118	6.436693
24	H	3.684913	4.197886	8.398019	7.707241	6.749510
25	C	5.190630	5.497175	8.010418	7.489265	6.395078
26	H	5.786766	5.810390	8.427421	7.850891	6.635570
27	C	5.737755	6.228279	7.694695	7.353791	6.378842
28	C	3.860987	3.371946	5.563396	4.826230	3.634878
29	O	4.607553	3.703072	5.961832	5.145171	3.816459
30	C	3.006575	3.142157	4.123128	3.502455	3.808854
31	O	3.009300	3.448407	5.102762	4.540470	4.931852
32	C	4.407904	4.696685	2.943248	2.816089	3.037122
33	O	5.115311	5.656651	3.502429	3.668375	3.950757
34	C	5.173600	5.282227	4.939644	4.675030	3.776218
35	O	6.119624	6.136161	4.496558	4.443583	3.656914
		11	12	13	14	15

11	H	0.000000				
12	H	4.350892	0.000000			
13	C	3.341391	2.230715	0.000000		
14	H	4.356954	2.674682	1.080693	0.000000	
15	C	2.242555	3.334036	1.410759	2.230838	0.000000
16	H	2.699914	4.343400	2.223678	2.689716	1.079355
17	C	1.079234	3.340285	2.298800	3.346424	1.429295
18	H	8.706699	6.335531	7.143680	7.102153	7.902455
19	C	8.573617	6.060298	6.682611	6.497302	7.512247
20	C	8.300356	6.285932	6.423134	6.164717	7.114626
21	H	9.390511	5.931686	7.014919	6.690566	8.141042
22	H	8.164366	6.723576	6.656014	6.496324	7.146847
23	C	8.526251	6.229738	6.190741	5.703477	7.010916
24	H	8.642230	6.668073	6.277224	5.695527	7.002399
25	C	8.931833	5.963409	6.321948	5.779653	7.359644
26	H	9.350466	6.151019	6.481955	5.801021	7.613622
27	C	8.942489	5.833879	6.603943	6.255502	7.640282
28	C	6.196388	3.456307	3.319085	2.754578	4.417156
29	O	6.512268	3.613244	3.302191	2.445338	4.511940
30	C	3.121731	4.614307	3.481034	4.079562	2.879919
31	O	3.736079	5.754396	4.505612	5.035845	3.740338
32	C	3.849041	3.345119	3.655646	4.339884	3.805232
33	O	4.725481	4.054835	4.730151	5.385236	4.921119
34	C	6.479505	3.182813	4.358143	4.337293	5.408472
35	O	6.448414	2.873421	4.565387	4.705537	5.621725
		16	17	18	19	20
16	H	0.000000				
17	C	2.240233	0.000000			
18	H	8.521463	8.009577	0.000000		
19	C	8.064455	7.805954	1.079025	0.000000	
20	C	7.497856	7.572347	2.220374	1.403982	0.000000
21	H	8.808663	8.479493	2.700430	2.242006	3.329675
22	H	7.451422	7.548491	2.674419	2.215565	1.079387
23	C	7.312757	7.703190	3.339208	2.294837	1.424042
24	H	7.147530	7.844522	4.346731	3.335811	2.236554
25	C	7.793446	8.016139	3.347936	2.306491	2.300358
26	H	8.017394	8.380904	4.358080	3.350124	3.344054
27	C	8.220687	8.059084	2.244235	1.431890	2.287520
28	C	4.889039	5.214551	4.734557	3.951325	3.667669
29	O	4.904112	5.484970	5.803320	4.973206	4.699859
30	C	3.086477	2.899456	6.452138	6.250610	5.676536
31	O	3.679870	3.768987	6.993770	6.817352	6.096637
32	C	4.600060	3.335336	4.967543	5.033635	5.053183
33	O	5.724404	4.324010	4.564372	4.871152	5.104291
34	C	6.209725	5.574607	3.261391	2.975843	3.565297
35	O	6.543846	5.564264	3.914313	3.860526	4.626463

		21	22	23	24	25
21	H	0.000000				
22	H	4.340923	0.000000			
23	C	3.330466	2.233121	0.000000		
24	H	4.340024	2.689934	1.078960	0.000000	
25	C	2.223026	3.342088	1.420538	2.232083	0.000000
26	H	2.679482	4.352191	2.234059	2.691180	1.078929
27	C	1.079594	3.332133	2.286751	3.329654	1.412176
28	C	4.154684	4.265003	3.079576	3.316235	3.034505
29	O	4.803353	5.341239	3.868460	3.926160	3.634692
30	C	7.605972	5.323666	6.048187	6.093226	6.801980
31	O	8.382171	5.561983	6.532387	6.471472	7.454536
32	C	6.073183	4.982355	5.673879	6.155532	6.013691
33	O	5.965681	5.004491	5.947707	6.564574	6.232775
34	C	2.959278	4.226625	3.772303	4.582847	3.357483
35	O	3.508230	5.249657	4.898836	5.726029	4.362404

		26	27	28	29	30
26	H	0.000000				
27	C	2.226050	0.000000			
28	C	3.200432	3.583298	0.000000		
29	O	3.439773	4.366081	1.191078	0.000000	
30	C	7.419542	6.899464	4.603839	5.323011	0.000000
31	O	8.117708	7.597799	5.477161	6.208099	1.140771
32	C	6.709352	5.635268	4.176061	5.080510	2.545539
33	O	7.021577	5.613108	4.877224	5.863801	3.459016
34	C	3.894951	2.798384	2.520276	3.400980	5.090809
35	O	4.805314	3.643253	3.420719	4.112227	5.552684

		31	32	33	34	35
31	O	0.000000				
32	C	3.469336	0.000000			
33	O	4.204646	1.137360	0.000000		
34	C	6.044869	3.350161	3.499565	0.000000	
35	O	6.570488	3.460559	3.465060	1.149427	0.000000

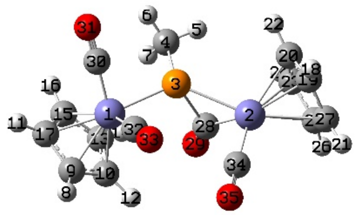

4T -3747.697911 16.76 C1
WBI 0.03

Charge and spin density
1 2

		1	2	3	4	5
1	Fe	0.000000				
2	Fe	3.941693	0.000000			
3	P	2.336426	2.230307	0.000000		
4	C	3.414950	3.321927	1.860115	0.000000	
5	H	4.387385	3.335890	2.438701	1.091440	0.000000
6	H	3.587217	3.630773	2.510556	1.093128	1.770749
7	H	3.565071	4.286025	2.458485	1.092418	1.763249
8	H	2.850696	6.184401	5.104827	6.176957	7.181085
9	C	2.101536	5.538915	4.306378	5.514104	6.484694
10	C	2.108102	4.565461	3.702585	5.264398	6.090445
11	H	2.844629	6.734946	4.893340	5.586036	6.607828
12	H	2.864332	4.434602	4.116136	5.755510	6.500356

1	Fe	0.018136	0.111408
2	Fe	0.329880	2.234842
3	P	0.082082	-0.029830
4	C	-0.026061	0.016721
9	C	0.043656	0.001138
10	C	0.025726	-0.002867
13	C	0.074417	-0.001946
15	C	0.061931	-0.003227
17	C	0.022780	-0.005882
19	C	-0.031202	-0.010300
20	C	-0.036977	0.038243
23	C	-0.023524	-0.029458
25	C	-0.023095	0.015166
27	C	-0.040280	0.053965
28	C	0.066304	-0.149621
29	O	-0.261133	-0.081995
30	C	0.006017	-0.003961
31	O	-0.082961	-0.002002
32	C	0.024019	0.002392
33	O	-0.067784	-0.002417
34	C	-0.066679	-0.076715
35	O	-0.095252	-0.073657

13	C	2.119649	4.323492	3.118974	4.745014	5.479529
14	H	2.859656	3.919017	3.033632	4.792886	5.340574
15	C	2.113330	5.197980	3.459954	4.673651	5.515068
16	H	2.865221	5.596842	3.689948	4.683516	5.434766
17	C	2.099725	5.867940	4.176071	5.173582	6.156998
18	H	5.769563	2.955476	4.644755	4.951454	4.902168
19	C	5.600475	2.229666	4.097623	4.438430	4.229627
20	C	5.275140	2.145428	3.433958	3.327959	2.930513
21	H	6.601965	2.962834	5.190723	6.082200	5.850914
22	H	5.225798	2.880161	3.540381	2.906366	2.557317
23	C	5.629085	2.148706	3.451636	3.555600	2.902790
24	H	5.867861	2.876084	3.560041	3.367098	2.490169
25	C	6.102372	2.207647	4.095238	4.695558	4.184921
26	H	6.684956	2.940172	4.662055	5.397955	4.843012
27	C	6.052442	2.222003	4.417796	5.118279	4.818194
28	C	3.769293	1.770238	2.944755	4.577620	4.838184
29	O	4.135654	2.916034	3.825436	5.568333	5.907630
30	C	1.747629	4.871096	2.935032	2.970932	4.056045
31	O	2.888807	5.730816	3.775712	3.263313	4.293266
32	C	1.751528	3.517315	2.756485	3.542824	4.436450
33	O	2.894550	3.725879	3.533388	4.080436	4.854615

		6	7	8	9	10
6	H	0.000000				
7	H	1.769652	0.000000			
8	H	6.110180	6.274799	0.000000		
9	C	5.617265	5.579196	1.079045	0.000000	
10	C	5.510719	5.517063	2.239041	1.425114	0.000000
11	H	5.768624	5.216534	2.678904	2.222342	3.333251
12	H	5.930339	6.175188	2.700268	2.238401	1.078450
13	C	5.255395	4.869067	3.339733	2.296586	1.413392
14	H	5.441531	5.009356	4.348903	3.338976	2.224351
15	C	5.193770	4.483457	3.344602	2.301302	2.293962
16	H	5.352214	4.299793	4.353286	3.343302	3.336169
17	C	5.426572	4.974294	2.225846	1.411505	2.288938
18	H	4.588297	6.031363	7.417966	7.157416	6.426182
19	C	4.286688	5.529409	7.559961	7.148575	6.313923
20	C	3.262528	4.399956	7.616025	7.101648	6.380319
21	H	6.179576	7.119523	8.252797	7.791506	6.658666
22	H	2.577202	3.932761	7.596230	7.139417	6.618722
23	C	3.902181	4.511595	8.142195	7.471607	6.597666
24	H	3.901398	4.154876	8.550468	7.805493	6.995721
25	C	5.058418	5.653555	8.368520	7.703005	6.634473
26	H	5.903979	6.263297	8.930836	8.195596	7.032809
27	C	5.228519	6.165867	7.989217	7.475612	6.424835
28	C	4.907321	5.375443	5.389593	4.752211	3.511277
29	O	5.898573	6.279974	5.194941	4.603255	3.242461

30	C	2.947868	2.727659	3.769715	3.272355	3.766011
31	O	3.104123	2.757203	4.601237	4.227675	4.870476
32	C	3.259509	4.070771	3.249901	2.995571	2.909167
33	O	3.555105	4.752269	3.905862	3.888700	3.788216
		11	12	13	14	15
11	H	0.000000				
12	H	4.343708	0.000000			
13	C	3.332164	2.223167	0.000000		
14	H	4.340994	2.675985	1.078583	0.000000	
15	C	2.234512	3.334544	1.414391	2.222992	0.000000
16	H	2.694691	4.342486	2.228239	2.679763	1.078537
17	C	1.079965	3.332214	2.288775	3.331256	1.424234
18	H	8.557817	6.024608	6.705627	6.547782	7.548862
19	C	8.439562	5.995721	6.368709	6.075235	7.212185
20	C	8.046725	6.298282	6.202306	5.919950	6.825385
21	H	9.379564	6.095833	6.704044	6.167551	7.839013
22	H	7.876784	6.650500	6.470505	6.334994	6.901083
23	C	8.331563	6.563568	6.148852	5.648236	6.804760
24	H	8.402967	7.105258	6.360015	5.835505	6.848745
25	C	8.845034	6.409094	6.254842	5.613787	7.151526
26	H	9.323866	6.802386	6.524281	5.740931	7.463934
27	C	8.874326	6.018816	6.353076	5.847121	7.359381
28	C	6.347653	3.106206	3.474669	3.006617	4.693688
29	O	6.407255	2.602373	3.386668	2.920357	4.756337
30	C	2.867320	4.564588	3.634209	4.335532	3.017909
31	O	3.356628	5.683604	4.705019	5.391412	3.910816
32	C	4.205433	3.104752	3.460395	4.011729	3.823868
33	O	5.204669	3.721617	4.493902	4.967469	4.954373
		16	17	18	19	20
16	H	0.000000				
17	C	2.238799	0.000000			
18	H	8.118696	7.800465	0.000000		
19	C	7.673343	7.650841	1.079763	0.000000	
20	C	7.121592	7.349089	2.227913	1.413114	0.000000
21	H	8.338522	8.436599	2.676014	2.222540	3.324621
22	H	7.166249	7.299909	2.688259	2.227397	1.078945
23	C	6.948662	7.581529	3.345153	2.299996	1.430337
24	H	6.825139	7.721318	4.353830	3.341672	2.240865
25	C	7.385428	7.978696	3.341251	2.298605	2.294958
26	H	7.622454	8.422613	4.346991	3.339331	3.341613
27	C	7.783040	7.984675	2.222940	1.410517	2.281049
28	C	5.292906	5.338804	3.868217	3.446142	3.828897
29	O	5.451389	5.346776	4.715622	4.423324	4.946671
30	C	3.340330	2.752421	6.398948	6.248312	5.619588
31	O	4.032199	3.561739	7.061874	6.929127	6.164142
32	C	4.608089	3.570292	4.453666	4.556370	4.480918

33	O	5.750560	4.624169	3.824167	4.178955	4.320567

		21	22	23	24	25
21	H	0.000000				
22	H	4.335487	0.000000			
23	C	3.327990	2.239213	0.000000		
24	H	4.340196	2.693886	1.079449	0.000000	
25	C	2.230226	3.335686	1.408868	2.224470	0.000000
26	H	2.681018	4.349743	2.226618	2.688643	1.079604
27	C	1.080077	3.324978	2.285934	3.331434	1.421018
28	C	3.249490	4.550288	3.793423	4.484137	3.354606
29	O	3.841175	5.667784	4.894210	5.571286	4.301948
30	C	7.669633	5.241606	6.084703	6.131020	6.889406
31	O	8.530353	5.614993	6.672638	6.609654	7.627243
32	C	5.779807	4.374221	5.230146	5.720321	5.673865
33	O	5.517488	4.172794	5.314930	5.946586	5.713464

		26	27	28	29	30
26	H	0.000000				
27	C	2.231087	0.000000			
28	C	3.731188	3.076007	0.000000		
29	O	4.516025	3.937905	1.146610	0.000000	
30	C	7.562216	6.950253	5.205514	5.758434	0.000000
31	O	8.331639	7.741042	6.255399	6.867869	1.142282
32	C	6.456103	5.271394	3.531032	4.026348	2.544527
33	O	6.596653	5.069265	3.852376	4.369876	3.463854

		31	32	33
31	O	0.000000		
32	C	3.477646	0.000000	
33	O	4.220945	1.143434	0.000000

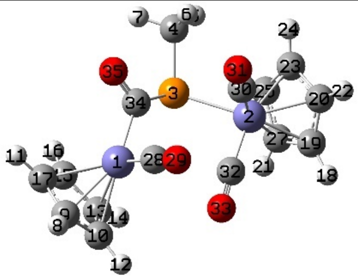

5T -3747.690898 21.16 C1
WBI 0.01

Charge and spin density

		1	2
1	Fe	0.333799	2.302286
2	Fe	0.102746	0.001458
3	P	-0.048592	-0.028705
4	C	-0.011807	-0.002692
9	C	-0.033256	-0.033520

		1	2	3	4	5
1	Fe	0.000000				
2	Fe	4.387681	0.000000			
3	P	3.397963	2.315813	0.000000		
4	C	4.709426	3.346543	1.849229	0.000000	
5	H	5.646020	3.520259	2.442615	1.091728	0.000000
6	H	4.860722	3.477976	2.493116	1.091566	1.782963
7	H	4.822207	4.321502	2.419529	1.091593	1.770689
8	H	2.951770	7.169240	6.324697	7.569386	8.545196
9	C	2.208406	6.443202	5.451294	6.776593	7.712134
10	C	2.204923	5.716222	5.006588	6.596798	7.411160
11	H	2.940517	7.098377	5.471669	6.317614	7.305280
12	H	2.939990	5.884086	5.580700	7.255552	8.019166
13	C	2.176076	5.158476	4.099900	5.721690	6.452368
14	H	2.891113	4.786461	3.909431	5.651145	6.228084
15	C	2.170363	5.629028	4.043141	5.336388	6.160191
16	H	2.900339	5.723232	3.825739	4.920340	5.672614
17	C	2.222526	6.421353	4.960674	6.084847	7.028993

10	C	-0.044477	-0.011803
13	C	-0.060132	0.038270
15	C	0.035303	-0.032929
17	C	-0.034916	0.057817
19	C	0.008036	0.000033
20	C	0.040425	-0.000014
23	C	0.043314	0.000440
25	C	0.086575	-0.000148
27	C	0.021740	-0.000353
28	C	-0.034697	-0.080556
29	O	-0.083925	-0.035889
30	C	-0.003473	0.000038
31	O	-0.067913	0.000419
32	C	-0.007608	-0.001696
33	O	-0.087197	0.003621
34	C	0.129016	-0.160034
35	O	-0.282962	-0.016045

18	H	6.448009	2.845429	5.002684	6.091171	6.008040
19	C	6.161547	2.095623	4.219319	5.147227	4.980844
20	C	6.412830	2.094981	4.196135	4.641517	4.418609
21	H	5.976343	2.867323	3.889120	5.135593	4.905630
22	H	6.892311	2.840714	4.961339	5.259234	5.102014
23	C	6.311751	2.096714	3.488003	3.562873	3.131331
24	H	6.729121	2.853809	3.798686	3.320315	2.777421
25	C	6.008707	2.114163	3.026301	3.519253	3.043245
26	H	6.155268	2.862590	2.926376	3.207487	2.566210
27	C	5.900875	2.108771	3.542249	4.561900	4.294718
28	C	1.792208	4.437756	4.207725	5.290403	6.230789
29	O	2.932791	4.865099	5.030916	5.962961	6.870539
30	C	4.138201	1.754931	2.789772	3.256248	3.747819
31	O	4.401642	2.894280	3.566273	3.669610	4.277061
32	C	3.486452	1.753496	2.948620	4.509751	4.926754
33	O	3.387134	2.892655	3.808298	5.483209	5.970678
34	C	1.933171	3.444388	1.865644	2.802921	3.793771
35	O	2.619951	4.394137	2.698503	2.883360	3.967828
		6	7	8	9	10
6	H	0.000000				
7	H	1.764631	0.000000			
8	H	7.657913	7.514849	0.000000		
9	C	6.982266	6.712091	1.079837	0.000000	
10	C	6.911075	6.693105	2.236788	1.427211	0.000000
11	H	6.553277	5.908373	2.680265	2.223997	3.343465
12	H	7.526321	7.475480	2.691394	2.238301	1.079281
13	C	6.221768	5.756354	3.330023	2.287710	1.407699
14	H	6.262366	5.788548	4.344565	3.334881	2.225029
15	C	5.841290	5.117025	3.329549	2.285660	2.295615
16	H	5.559859	4.548179	4.340753	3.329887	3.336674
17	C	6.386342	5.824702	2.223531	1.410134	2.299195
18	H	6.214974	7.086223	8.763824	8.127716	7.076423
19	C	5.309768	6.159833	8.715208	8.011362	7.044283
20	C	4.579733	5.722867	9.103434	8.445131	7.667271
21	H	5.722213	5.919173	8.469878	7.589178	6.478756
22	H	4.987051	6.348396	9.478388	8.917578	8.210015
23	C	3.640624	4.624177	9.180954	8.432370	7.750190
24	H	3.222473	4.350534	9.634280	8.909735	8.369237
25	C	3.991278	4.450448	8.861355	8.005379	7.202054
26	H	3.911317	3.968590	9.024279	8.103622	7.359388
27	C	5.007450	5.468398	8.556077	7.722846	6.729010
28	C	5.086226	5.615807	3.349533	3.140451	3.237717
29	O	5.585267	6.377789	3.981578	4.018333	4.144948
30	C	2.846774	4.181336	6.823133	6.278238	5.887116
31	O	2.956127	4.471213	6.876599	6.471078	6.303144
32	C	4.683129	5.284911	5.916523	5.237442	4.323465

33	O	5.671570	6.156918	5.342156	4.729696	3.664531
34	C	2.983896	2.939695	4.773996	4.019388	3.982066
35	O	2.866841	2.693484	5.044659	4.414588	4.727526
		11	12	13	14	15
11	H	0.000000				
12	H	4.350967	0.000000			
13	C	3.338906	2.225279	0.000000		
14	H	4.345591	2.689180	1.080162	0.000000	
15	C	2.226267	3.341048	1.425315	2.231858	0.000000
16	H	2.686577	4.348821	2.235711	2.682995	1.079374
17	C	1.080030	3.340524	2.294959	3.336219	1.414823
18	H	9.309821	6.823251	6.737293	6.120798	7.644397
19	C	8.954221	6.955864	6.543995	5.937406	7.289620
20	C	9.164374	7.687513	7.180008	6.716072	7.719147
21	H	8.425844	6.496171	5.653498	4.793596	6.428268
22	H	9.684943	8.175064	7.867574	7.491272	8.402355
23	C	8.823733	7.957981	7.054642	6.595048	7.376146
24	H	9.083097	8.663000	7.662561	7.295628	7.811819
25	C	8.403012	7.440946	6.333135	5.724467	6.708342
26	H	8.265311	7.721535	6.332194	5.713017	6.545970
27	C	8.474951	6.783880	5.972543	5.248519	6.635979
28	C	4.148266	3.511384	3.728591	4.334234	3.931660
29	O	5.088281	4.210889	4.792301	5.356028	5.058328
30	C	6.775487	6.120584	5.589665	5.549423	5.823538
31	O	6.839916	6.565889	6.161655	6.291648	6.250216
32	C	6.361316	4.295589	4.016326	3.669145	4.824910
33	O	6.199544	3.409301	3.619548	3.304473	4.673057
34	C	3.894534	4.706879	3.397633	3.720593	3.033587
35	O	3.726125	5.546409	4.227763	4.705014	3.505597
		16	17	18	19	20
16	H	0.000000				
17	C	2.230630	0.000000			
18	H	7.895179	8.456962	0.000000		
19	C	7.415896	8.165852	1.079499	0.000000	
20	C	7.785670	8.499655	2.221279	1.408965	0.000000
21	H	6.389762	7.575040	2.700961	2.242671	3.340343
22	H	8.539985	9.054826	2.676275	2.222132	1.079395
23	C	7.253588	8.249169	3.337265	2.293355	1.425909
24	H	7.615824	8.620918	4.346042	3.335573	2.238275
25	C	6.503910	7.757793	3.335284	2.292463	2.295567
26	H	6.174261	7.681226	4.344162	3.335227	3.339845
27	C	6.601189	7.690523	2.240230	1.428931	2.298007
28	C	4.676771	3.629835	6.243864	6.094519	6.167694
29	O	5.814875	4.650771	6.400807	6.351222	6.317896
30	C	6.012141	6.274600	4.041825	3.458258	2.888518
31	O	6.481065	6.475801	4.995364	4.483386	3.749672

32	C	5.208792	5.521867	2.962978	2.799543	3.393570
33	O	5.246631	5.265237	3.464114	3.601287	4.376066
34	C	3.101011	3.507983	6.072606	5.488438	5.432964
35	O	3.437580	3.685049	7.124377	6.483956	6.239776
		21	22	23	24	25
21	H	0.000000				
22	H	4.351222	0.000000			
23	C	3.336763	2.237732	0.000000		
24	H	4.345231	2.695919	1.078947	0.000000	
25	C	2.224292	3.338788	1.416521	2.230514	0.000000
26	H	2.672724	4.350131	2.229200	2.688196	1.079347
27	C	1.078863	3.342053	2.294349	3.336619	1.410906
28	C	6.530071	6.373162	6.352743	6.724333	6.411430
29	O	7.120246	6.332144	6.678950	7.013892	6.938362
30	C	4.620504	3.071548	2.939253	3.177498	3.545297
31	O	5.759778	3.665903	3.809621	3.798025	4.583315
32	C	3.150357	3.966719	3.787051	4.601229	3.530810
33	O	3.733944	4.868472	4.893671	5.734716	4.558449
34	C	5.390046	5.971845	4.986563	5.199885	4.777669
35	O	6.474993	6.692027	5.671571	5.672962	5.601832
		26	27	28	29	30
26	H	0.000000				
27	C	2.218812	0.000000			
28	C	6.806115	6.247641	0.000000		
29	O	7.455029	6.740877	1.141451	0.000000	
30	C	4.172296	3.820634	3.661130	3.840495	0.000000
31	O	5.154242	4.943091	3.595215	3.542899	1.139846
32	C	4.180416	2.901655	3.511858	3.992650	2.591706
33	O	5.168204	3.736961	3.383495	3.846882	3.550106
34	C	4.786892	5.094510	2.799144	3.742514	3.100383
35	O	5.513520	6.110582	3.291025	4.118982	3.724781
		31	32	33	34	35
31	O	0.000000				
32	C	3.538361	0.000000			
33	O	4.345154	1.141623	0.000000		
34	C	3.410063	3.394171	3.900494	0.000000	
35	O	3.716922	4.536234	5.052750	1.210201	0.000000

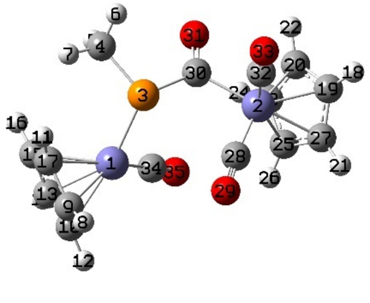

6T -3747.690427 21.46 C1

		1	2	3	4	5
1	Fe	0.000000				
2	Fe	4.448422	0.000000			
3	P	2.248783	3.328209	0.000000		
4	C	3.356918	4.767431	1.845062	0.000000	
5	H	3.555952	5.058830	2.473910	1.090455	0.000000
6	H	4.320248	4.846932	2.427847	1.091557	1.766653
7	H	3.478114	5.641727	2.433474	1.090971	1.781161
8	H	2.878471	6.305423	4.226707	5.279774	5.839607

WBI 0.01

Charge and spin density

		1	2
1	Fe	0.299291	2.168142
2	Fe	0.102641	0.010941
3	P	-0.129702	-0.017089
4	C	-0.011289	0.011072
9	C	-0.042169	0.055989
10	C	-0.019919	-0.037811
13	C	-0.015964	-0.023077
15	C	0.018806	0.041382
17	C	0.047233	-0.051249
19	C	0.023594	-0.000496
20	C	0.093239	-0.001236
23	C	0.028663	0.003368
25	C	0.026759	-0.001246
27	C	0.024628	0.000686
28	C	-0.057806	0.002697
29	O	-0.099399	0.000518
30	C	0.151590	-0.001026
31	O	-0.278888	-0.000858
32	C	0.001749	-0.000399
33	O	-0.087038	0.001127
34	C	0.034947	-0.122814
35	O	-0.110965	-0.038620

9	C	2.164930	6.186867	3.828278	4.692539	5.101308
10	C	2.148619	6.329770	4.318949	5.194390	5.334539
11	H	2.909355	6.448902	3.326673	3.401689	4.012633
12	H	2.885242	6.615377	5.079976	6.132800	6.246294
13	C	2.198988	6.531771	4.217488	4.647216	4.538259
14	H	2.939127	6.950654	4.899289	5.229078	4.908456
15	C	2.232287	6.502239	3.633364	3.652579	3.654474
16	H	2.965576	6.866890	3.875532	3.373205	3.171390
17	C	2.169026	6.243910	3.304752	3.643155	4.046451
18	H	7.304009	2.856866	6.004811	7.181614	7.450744
19	C	6.487512	2.113329	5.372613	6.605580	6.783696
20	C	6.033319	2.100706	4.845022	5.806548	5.805072
21	H	6.464323	2.881242	5.979334	7.584661	7.845145
22	H	6.512904	2.831411	5.072952	5.716694	5.663637
23	C	5.178659	2.111814	4.476728	5.551527	5.417843
24	H	4.925379	2.844108	4.382510	5.212099	4.881532
25	C	5.137323	2.113638	4.804964	6.217010	6.221277
26	H	4.867646	2.866096	5.023640	6.503636	6.464858
27	C	5.999857	2.128672	5.356498	6.837892	7.013830
28	C	3.623059	1.757929	3.133663	4.930398	5.391509
29	O	3.598854	2.896662	3.626474	5.470273	5.997382
30	C	3.505121	1.996533	1.860516	2.869218	3.083132
31	O	4.366297	2.808815	2.666442	2.918786	2.866897
32	C	5.183159	1.753768	3.490937	4.712979	5.284799
33	O	5.919449	2.894351	4.052216	5.045516	5.747722
34	C	1.773082	3.782391	2.962768	4.168581	4.053073
35	O	2.913423	3.846989	3.845867	4.977097	4.709285

		6	7	8	9	10
6	H	0.000000				
7	H	1.774818	0.000000			
8	H	6.101934	4.910931	0.000000		
9	C	5.626062	4.319911	1.079834	0.000000	
10	C	6.218881	4.980731	2.227728	1.410934	0.000000
11	H	4.199363	2.637348	2.678707	2.227090	3.327279
12	H	7.139078	6.001319	2.692665	2.228415	1.079546
13	C	5.732730	4.429798	3.345513	2.298958	1.433234
14	H	6.313661	5.116548	4.353479	3.339982	2.241431
15	C	4.721013	3.215780	3.342963	2.300411	2.294573
16	H	4.406570	2.885216	4.350623	3.342632	3.340083
17	C	4.597118	3.082928	2.228868	1.418054	2.285266
18	H	6.942839	8.122520	8.966878	8.969913	9.133263
19	C	6.501724	7.563849	8.327251	8.248593	8.294463
20	C	5.708478	6.838954	8.252903	7.996797	7.959973
21	H	7.717196	8.390553	7.796792	7.894212	7.844911
22	H	5.456143	6.786899	8.822938	8.514289	8.538257
23	C	5.718097	6.539240	7.552697	7.203750	6.962736

24	H	5.471802	6.199475	7.540112	7.045624	6.686827
25	C	6.491430	7.098251	7.155481	6.949439	6.672800
26	H	6.937759	7.295788	6.794071	6.570168	6.123713
27	C	6.945066	7.715434	7.680881	7.646971	7.568465
28	C	5.252044	5.544476	4.874601	4.946139	5.173379
29	O	5.901437	5.902585	4.193933	4.454427	4.731072
30	C	2.998214	3.842669	5.796108	5.425618	5.634815
31	O	2.799478	4.002694	6.805436	6.337209	6.496970
32	C	4.471194	5.503688	6.598183	6.630783	7.116063
33	O	4.611562	5.738857	7.045488	7.164633	7.821153
34	C	5.015919	4.656880	4.334468	3.713024	3.158983
35	O	5.740280	5.588776	5.378896	4.783483	4.062327
		11	12	13	14	15
11	H	0.000000				
12	H	4.340409	0.000000			
13	C	3.326660	2.241300	0.000000		
14	H	4.339408	2.692131	1.079582	0.000000	
15	C	2.234550	3.334479	1.402024	2.218690	0.000000
16	H	2.689343	4.346251	2.217107	2.677225	1.079911
17	C	1.079579	3.331351	2.283931	3.329540	1.421327
18	H	9.157632	9.322390	9.371694	9.732078	9.343447
19	C	8.554898	8.460537	8.484812	8.782774	8.539975
20	C	8.138622	8.230294	7.913789	8.120256	7.908676
21	H	8.719554	7.748500	8.356195	8.671313	8.681923
22	H	8.396171	8.910912	8.359866	8.561602	8.211712
23	C	7.609433	7.154261	6.882819	6.981037	7.064016
24	H	7.394220	6.932170	6.403059	6.382412	6.593190
25	C	7.705088	6.681870	6.862298	7.007644	7.224227
26	H	7.606989	5.993050	6.381358	6.453503	6.936891
27	C	8.309211	7.578795	7.907042	8.177799	8.164144
28	C	5.596536	5.349687	5.713446	6.277124	5.810880
29	O	5.423002	4.778791	5.534731	6.173644	5.741663
30	C	5.179688	6.204085	5.476377	5.908683	5.146655
31	O	5.838029	7.124038	6.115615	6.435754	5.683948
32	C	6.449034	7.541749	7.367004	7.960062	7.046997
33	O	6.730425	8.309097	8.093768	8.765851	7.627076
34	C	4.661171	3.436334	3.072396	3.260842	3.578624
35	O	5.792245	4.136650	3.913255	3.831306	4.563180
		16	17	18	19	20
16	H	0.000000				
17	C	2.231997	0.000000			
18	H	9.648432	9.050589	0.000000		
19	C	8.852453	8.350195	1.079995	0.000000	
20	C	8.077228	7.913566	2.233122	1.423071	0.000000
21	H	9.234501	8.370521	2.680291	2.225666	3.342488
22	H	8.248876	8.261741	2.695335	2.239012	1.078156

23	C	7.285834	7.214942	3.335549	2.292627	1.415991
24	H	6.708838	6.943624	4.344081	3.334867	2.226198
25	C	7.640983	7.234185	3.332117	2.287248	2.289355
26	H	7.440824	7.007658	4.342141	3.330215	3.331006
27	C	8.617063	7.967183	2.221744	1.410091	2.298617
28	C	6.420145	5.316237	4.150452	3.514438	3.795761
29	O	6.503594	5.091933	5.082970	4.506899	4.901818
30	C	5.279621	5.063999	4.455248	3.769732	3.039537
31	O	5.595107	5.783796	4.707038	4.084489	3.035302
32	C	7.356731	6.532473	2.957200	2.828506	3.051663
33	O	7.898606	6.983291	3.499994	3.669855	3.964698
34	C	4.105178	3.900236	6.504698	5.543827	5.005532
35	O	4.993871	5.016350	6.299479	5.263128	4.670975
		21	22	23	24	25
21	H	0.000000				
22	H	4.351813	0.000000			
23	C	3.342627	2.225245	0.000000		
24	H	4.351194	2.677632	1.078081	0.000000	
25	C	2.239692	3.329751	1.412484	2.224232	0.000000
26	H	2.698176	4.336770	2.222194	2.676004	1.078876
27	C	1.079108	3.340783	2.300152	3.342179	1.426073
28	C	3.139309	4.585971	3.433902	4.006919	2.834556
29	O	3.659762	5.723259	4.444785	4.964056	3.649262
30	C	4.844704	3.220269	2.848896	2.868178	3.498008
31	O	5.567468	2.822932	3.051255	2.852721	4.082571
32	C	3.841711	3.345431	3.651572	4.332443	3.791672
33	O	4.702859	4.059473	4.730063	5.388036	4.906106
34	C	5.479938	5.606168	3.881337	3.537617	3.802349
35	O	5.171405	5.332469	3.366383	2.931781	3.267116
		26	27	28	29	30
26	H	0.000000				
27	C	2.237803	0.000000			
28	C	3.017448	2.908045	0.000000		
29	O	3.555533	3.707290	1.143643	0.000000	
30	C	4.024379	4.013951	2.810869	3.777337	0.000000
31	O	4.713676	4.619040	3.959166	4.967196	1.204713
32	C	4.575297	3.338227	2.557437	3.491367	2.514460
33	O	5.699215	4.316311	3.483945	4.247357	3.354012
34	C	3.390942	4.927137	3.297925	3.532580	3.218192
35	O	2.722304	4.572128	3.659102	4.014049	3.589119
		31	32	33	34	35
31	O	0.000000				
32	C	3.151714	0.000000			
33	O	3.829513	1.140860	0.000000		
34	C	4.002435	5.049036	6.021591	0.000000	
35	O	4.218397	5.371234	6.436813	1.142841	0.000000

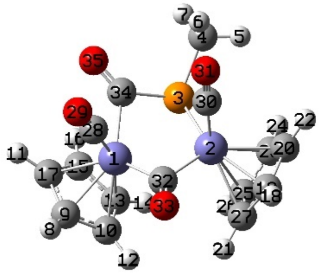

7S -3747.684202 25.36 C1
WBI 0.28

Charge and spin density

		1
1	Fe	-0.028477
2	Fe	-0.044654
3	P	-0.024327
4	C	-0.009879
9	C	0.036060
10	C	0.011576
13	C	0.019880
15	C	0.034229
17	C	0.058266
19	C	0.051500
20	C	0.027737
23	C	0.059041
25	C	0.041200
27	C	0.005051
28	C	0.023117
29	O	-0.074391
30	C	0.002900
31	O	-0.090482
32	C	0.198808
33	O	-0.173614
34	C	0.132508
35	O	-0.256049

		1	2	3	4	5
1	Fe	0.000000				
2	Fe	2.733075	0.000000			
3	P	2.864746	2.299833	0.000000		
4	C	4.414550	3.446479	1.855099	0.000000	
5	H	5.163070	3.661628	2.443151	1.092771	0.000000
6	H	4.533282	3.596669	2.504652	1.090432	1.778481
7	H	4.908702	4.391818	2.421350	1.092295	1.770330
8	H	2.834575	5.130930	5.667034	7.237113	7.995151
9	C	2.099454	4.515370	4.791929	6.443406	7.185909
10	C	2.139105	3.833842	4.315796	6.114960	6.685069
11	H	2.803761	5.508314	4.909524	6.221503	7.169713
12	H	2.889774	3.937436	4.887644	6.675422	7.123462
13	C	2.141771	3.640161	3.421060	5.254974	5.810109
14	H	2.897747	3.582106	3.289509	5.109267	5.487516
15	C	2.127820	4.272455	3.454538	5.093894	5.850363
16	H	2.880889	4.726988	3.363417	4.798338	5.575688
17	C	2.067231	4.735985	4.319412	5.846770	6.701082
18	H	4.950916	2.828475	5.086146	5.849519	5.759986
19	C	4.473478	2.089353	4.253855	5.118984	4.982578
20	C	4.813499	2.087354	3.686936	4.165334	3.809460
21	H	3.999579	2.865345	4.726382	6.181911	6.247338
22	H	5.529449	2.829910	4.168787	4.177820	3.691740
23	C	4.537073	2.114376	2.997752	3.749238	3.364369
24	H	5.065910	2.867341	2.939765	3.349173	2.753933
25	C	3.982157	2.137185	3.259925	4.568085	4.431552
26	H	4.093178	2.900130	3.428658	4.908958	4.827975
27	C	3.941339	2.118766	4.028210	5.321287	5.282868
28	C	1.751114	3.596955	3.841115	4.856455	5.731353
29	O	2.889134	4.478395	4.745344	5.467864	6.373247
30	C	3.319154	1.746213	2.825907	3.132818	3.496357
31	O	4.103374	2.888331	3.621006	3.430725	3.831232
32	C	1.899821	1.933162	3.519539	4.859460	5.348162
33	O	2.856768	2.863787	4.674511	5.928706	6.371091
34	C	1.992282	3.088302	1.794943	2.836027	3.797541
35	O	2.890840	4.206205	2.756072	3.223196	4.297295

		6	7	8	9	10
6	H	0.000000				
7	H	1.772045	0.000000			
8	H	7.278610	7.663602	0.000000		
9	C	6.607489	6.823396	1.079392	0.000000	
10	C	6.437700	6.585982	2.222333	1.407007	0.000000
11	H	6.330753	6.278124	2.700555	2.241648	3.333051
12	H	6.984891	7.260579	2.674332	2.217301	1.079124
13	C	5.766403	5.587226	3.336758	2.292222	1.423313
14	H	5.774706	5.453194	4.345550	3.333803	2.237269

15	C	5.540175	5.207437	3.345888	2.303157	2.295764
16	H	5.345629	4.696439	4.356074	3.346502	3.339021
17	C	6.059111	6.020585	2.240047	1.428056	2.289750
18	H	5.756760	6.909219	6.413044	6.170149	5.432190
19	C	5.198054	6.151564	6.235113	5.819019	4.942496
20	C	4.324540	5.207346	7.041063	6.476310	5.610294
21	H	6.453541	7.049680	4.985368	4.585987	3.461338
22	H	4.169746	5.238203	7.843876	7.314516	6.554669
23	C	4.254791	4.647785	6.890897	6.152882	5.149947
24	H	4.032596	4.121852	7.585392	6.762939	5.792414
25	C	5.106027	5.375619	5.962054	5.223199	4.052031
26	H	5.597498	5.548956	5.917162	5.077733	3.805699
27	C	5.615125	6.234584	5.506006	4.988154	3.895659
28	C	4.581072	5.337672	3.095516	2.893828	3.541745
29	O	4.994549	5.912615	3.692048	3.746767	4.570518
30	C	2.731011	4.125719	5.715769	5.271801	5.003555
31	O	2.680440	4.345464	6.360991	6.020389	5.943455
32	C	4.807928	5.669452	3.473885	3.147011	2.806317
33	O	5.778189	6.776812	3.512465	3.520986	3.292681
34	C	2.985532	3.088856	4.609898	3.836180	3.908237
35	O	3.199284	3.156872	5.115190	4.436281	4.792556
		11	12	13	14	15
11	H	0.000000				
12	H	4.343253	0.000000			
13	C	3.327878	2.236137	0.000000		
14	H	4.333014	2.697932	1.077601	0.000000	
15	C	2.228512	3.337954	1.410453	2.216454	0.000000
16	H	2.684369	4.347790	2.223831	2.670525	1.078698
17	C	1.079308	3.333139	2.285756	3.325609	1.418401
18	H	7.682209	4.981868	5.792828	5.709510	6.700776
19	C	7.261184	4.574241	5.080887	4.863078	6.029588
20	C	7.589309	5.446990	5.399138	5.036829	6.201772
21	H	6.464319	2.761118	3.904740	3.724689	5.143162
22	H	8.269504	6.440890	6.322488	5.995249	7.000149
23	C	7.171800	5.092844	4.639661	4.068833	5.477110
24	H	7.528709	5.864062	5.049791	4.383395	5.739208
25	C	6.540842	3.859072	3.668303	3.056551	4.758782
26	H	6.354108	3.678849	3.205052	2.366377	4.367408
27	C	6.606594	3.442086	4.016778	3.706188	5.158183
28	C	2.954449	4.183238	3.831310	4.645323	3.460676
29	O	3.509004	5.161637	4.944034	5.780639	4.468051
30	C	5.829513	5.236755	4.896988	5.051027	5.134385
31	O	6.330077	6.221360	5.874250	6.103783	5.936824
32	C	4.504889	2.862385	3.305499	3.745496	3.873557
33	O	5.119505	3.057597	4.141656	4.622651	4.773057
34	C	3.408368	4.725046	3.247715	3.660774	2.685533

35	O	3.378260	5.695261	4.153156	4.649980	3.251247
		16	17	18	19	20
16	H	0.000000				
17	C	2.230959	0.000000			
18	H	7.355711	6.886248	0.000000		
19	C	6.606455	6.409970	1.078756	0.000000	
20	C	6.561793	6.787887	2.239017	1.421784	0.000000
21	H	5.925095	5.471792	2.673270	2.221072	3.328383
22	H	7.287223	7.549483	2.698983	2.234024	1.079654
23	C	5.714839	6.313851	3.339535	2.295467	1.413009
24	H	5.776995	6.723140	4.350302	3.338832	2.225971
25	C	5.175777	5.572746	3.339194	2.298163	2.293728
26	H	4.723487	5.346972	4.349013	3.341407	3.334001
27	C	5.796202	5.645294	2.229142	1.416665	2.287195
28	C	4.058226	2.802617	5.395619	5.205764	5.568028
29	O	4.993497	3.641042	5.983876	5.948830	6.313457
30	C	5.483125	5.326252	3.298327	3.005669	2.780497
31	O	6.220966	5.991392	3.992091	3.908025	3.616968
32	C	4.665129	3.766475	3.196079	2.965466	3.694432
33	O	5.659252	4.434130	3.045505	3.182339	4.223684
34	C	2.669061	3.091831	5.778672	5.169216	4.951964
35	O	3.002817	3.436975	6.816376	6.270920	5.997682
		21	22	23	24	25
21	H	0.000000				
22	H	4.338939	0.000000			
23	C	3.329245	2.224877	0.000000		
24	H	4.338867	2.680348	1.079197	0.000000	
25	C	2.225272	3.336761	1.419248	2.229622	0.000000
26	H	2.690320	4.342656	2.228162	2.680685	1.078032
27	C	1.079045	3.331621	2.284811	3.327458	1.411655
28	C	5.138081	6.048699	5.649090	6.188999	5.351822
29	O	6.053053	6.660168	6.555286	7.088960	6.365738
30	C	4.327879	2.901942	3.327097	3.841262	3.806383
31	O	5.375456	3.433455	4.308513	4.710514	4.920974
32	C	2.763020	4.411972	3.903803	4.740707	3.379026
33	O	2.823836	4.892766	4.673979	5.614906	4.084361
34	C	5.348001	5.412330	4.542045	4.680830	4.520893
35	O	6.511259	6.339304	5.634316	5.671853	5.701622
		26	27	28	29	30
26	H	0.000000				
27	C	2.229374	0.000000			
28	C	5.670442	5.070711	0.000000		
29	O	6.757160	5.990815	1.138778	0.000000	
30	C	4.607050	3.635177	3.309415	3.813159	0.000000
31	O	5.734221	4.707859	3.632276	3.808109	1.142481
32	C	3.907251	2.731654	2.446125	3.307476	2.559593

		31	32	33	34	35
33	O	4.667150	3.079496	3.012272	3.615308	3.280270
34	C	4.644660	4.919648	2.477631	3.304821	3.119963
35	O	5.800284	6.098808	2.818800	3.318781	3.902135

		31	32	33	34	35
31	O	0.000000				
32	C	3.434197	0.000000			
33	O	3.979158	1.169788	0.000000		
34	C	3.652808	3.313358	4.404481	0.000000	
35	O	4.141148	4.300459	5.303975	1.195350	0.000000

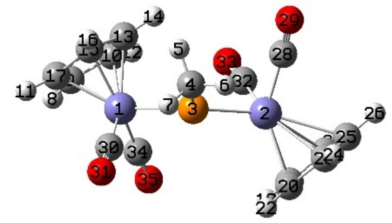

8Q -3747.682384 26.50 C1
WBI 0.03

Charge and spin density

		1	2
1	Fe	0.045332	0.144550
2	Fe	0.214123	3.154514
3	P	0.029453	0.613294
4	C	-0.006608	0.012300
9	C	0.014279	-0.001752
10	C	0.047114	0.001509
13	C	0.037143	0.000128
15	C	0.059939	-0.004070
17	C	0.021042	-0.003914
19	C	-0.084946	0.062302
20	C	-0.054602	0.020406
23	C	-0.088020	0.060039
25	C	-0.061315	0.080455
27	C	-0.053313	0.083139
28	C	0.044289	-0.033803
29	O	-0.041364	-0.065358
30	C	0.021268	-0.004931
31	O	-0.084271	-0.003955
32	C	0.056718	-0.037444
33	O	-0.056491	-0.064574
34	C	0.018064	-0.003756
35	O	-0.077834	-0.009080

		1	2	3	4	5
1	Fe	0.000000				
2	Fe	2.733075	0.000000			
3	P	2.864746	2.299833	0.000000		
4	C	4.414550	3.446479	1.855099	0.000000	
5	H	5.163070	3.661628	2.443151	1.092771	0.000000
6	H	4.533282	3.596669	2.504652	1.090432	1.778481
7	H	4.908702	4.391818	2.421350	1.092295	1.770330
8	H	2.834575	5.130930	5.667034	7.237113	7.995151
9	C	2.099454	4.515370	4.791929	6.443406	7.185909
10	C	2.139105	3.833842	4.315796	6.114960	6.685069
11	H	2.803761	5.508314	4.909524	6.221503	7.169713
12	H	2.889774	3.937436	4.887644	6.675422	7.123462
13	C	2.141771	3.640161	3.421060	5.254974	5.810109
14	H	2.897747	3.582106	3.289509	5.109267	5.487516
15	C	2.127820	4.272455	3.454538	5.093894	5.850363
16	H	2.880889	4.726988	3.363417	4.798338	5.575688
17	C	2.067231	4.735985	4.319412	5.846770	6.701082
18	H	4.950916	2.828475	5.086146	5.849519	5.759986
19	C	4.473478	2.089353	4.253855	5.118984	4.982578
20	C	4.813499	2.087354	3.686936	4.165334	3.809460
21	H	3.999579	2.865345	4.726382	6.181911	6.247338
22	H	5.529449	2.829910	4.168787	4.177820	3.691740
23	C	4.537073	2.114376	2.997752	3.749238	3.364369
24	H	5.065910	2.867341	2.939765	3.349173	2.753933
25	C	3.982157	2.137185	3.259925	4.568085	4.431552
26	H	4.093178	2.900130	3.428658	4.908958	4.827975
27	C	3.941339	2.118766	4.028210	5.321287	5.282868
28	C	1.751114	3.596955	3.841115	4.856455	5.731353
29	O	2.889134	4.478395	4.745344	5.467864	6.373247
30	C	3.319154	1.746213	2.825907	3.132818	3.496357
31	O	4.103374	2.888331	3.621006	3.430725	3.831232
32	C	1.899821	1.933162	3.519539	4.859460	5.348162
33	O	2.856768	2.863787	4.674511	5.928706	6.371091
34	C	1.992282	3.088302	1.794943	2.836027	3.797541
35	O	2.890840	4.206205	2.756072	3.223196	4.297295

		6	7	8	9	10

6	H	0.000000				
7	H	1.772045	0.000000			
8	H	7.278610	7.663602	0.000000		
9	C	6.607489	6.823396	1.079392	0.000000	
10	C	6.437700	6.585982	2.222333	1.407007	0.000000
11	H	6.330753	6.278124	2.700555	2.241648	3.333051
12	H	6.984891	7.260579	2.674332	2.217301	1.079124
13	C	5.766403	5.587226	3.336758	2.292222	1.423313
14	H	5.774706	5.453194	4.345550	3.333803	2.237269
15	C	5.540175	5.207437	3.345888	2.303157	2.295764
16	H	5.345629	4.696439	4.356074	3.346502	3.339021
17	C	6.059111	6.020585	2.240047	1.428056	2.289750
18	H	5.756760	6.909219	6.413044	6.170149	5.432190
19	C	5.198054	6.151564	6.235113	5.819019	4.942496
20	C	4.324540	5.207346	7.041063	6.476310	5.610294
21	H	6.453541	7.049680	4.985368	4.585987	3.461338
22	H	4.169746	5.238203	7.843876	7.314516	6.554669
23	C	4.254791	4.647785	6.890897	6.152882	5.149947
24	H	4.032596	4.121852	7.585392	6.762939	5.792414
25	C	5.106027	5.375619	5.962054	5.223199	4.052031
26	H	5.597498	5.548956	5.917162	5.077733	3.805699
27	C	5.615125	6.234584	5.506006	4.988154	3.895659
28	C	4.581072	5.337672	3.095516	2.893828	3.541745
29	O	4.994549	5.912615	3.692048	3.746767	4.570518
30	C	2.731011	4.125719	5.715769	5.271801	5.003555
31	O	2.680440	4.345464	6.360991	6.020389	5.943455
32	C	4.807928	5.669452	3.473885	3.147011	2.806317
33	O	5.778189	6.776812	3.512465	3.520986	3.292681
34	C	2.985532	3.088856	4.609898	3.836180	3.908237
35	O	3.199284	3.156872	5.115190	4.436281	4.792556

		11	12	13	14	15
11	H	0.000000				
12	H	4.343253	0.000000			
13	C	3.327878	2.236137	0.000000		
14	H	4.333014	2.697932	1.077601	0.000000	
15	C	2.228512	3.337954	1.410453	2.216454	0.000000
16	H	2.684369	4.347790	2.223831	2.670525	1.078698
17	C	1.079308	3.333139	2.285756	3.325609	1.418401
18	H	7.682209	4.981868	5.792828	5.709510	6.700776
19	C	7.261184	4.574241	5.080887	4.863078	6.029588
20	C	7.589309	5.446990	5.399138	5.036829	6.201772
21	H	6.464319	2.761118	3.904740	3.724689	5.143162
22	H	8.269504	6.440890	6.322488	5.995249	7.000149
23	C	7.171800	5.092844	4.639661	4.068833	5.477110
24	H	7.528709	5.864062	5.049791	4.383395	5.739208
25	C	6.540842	3.859072	3.668303	3.056551	4.758782

26	H	6.354108	3.678849	3.205052	2.366377	4.367408
27	C	6.606594	3.442086	4.016778	3.706188	5.158183
28	C	2.954449	4.183238	3.831310	4.645323	3.460676
29	O	3.509004	5.161637	4.944034	5.780639	4.468051
30	C	5.829513	5.236755	4.896988	5.051027	5.134385
31	O	6.330077	6.221360	5.874250	6.103783	5.936824
32	C	4.504889	2.862385	3.305499	3.745496	3.873557
33	O	5.119505	3.057597	4.141656	4.622651	4.773057
34	C	3.408368	4.725046	3.247715	3.660774	2.685533
35	O	3.378260	5.695261	4.153156	4.649980	3.251247
		16	17	18	19	20
16	H	0.000000				
17	C	2.230959	0.000000			
18	H	7.355711	6.886248	0.000000		
19	C	6.606455	6.409970	1.078756	0.000000	
20	C	6.561793	6.787887	2.239017	1.421784	0.000000
21	H	5.925095	5.471792	2.673270	2.221072	3.328383
22	H	7.287223	7.549483	2.698983	2.234024	1.079654
23	C	5.714839	6.313851	3.339535	2.295467	1.413009
24	H	5.776995	6.723140	4.350302	3.338832	2.225971
25	C	5.175777	5.572746	3.339194	2.298163	2.293728
26	H	4.723487	5.346972	4.349013	3.341407	3.334001
27	C	5.796202	5.645294	2.229142	1.416665	2.287195
28	C	4.058226	2.802617	5.395619	5.205764	5.568028
29	O	4.993497	3.641042	5.983876	5.948830	6.313457
30	C	5.483125	5.326252	3.298327	3.005669	2.780497
31	O	6.220966	5.991392	3.992091	3.908025	3.616968
32	C	4.665129	3.766475	3.196079	2.965466	3.694432
33	O	5.659252	4.434130	3.045505	3.182339	4.223684
34	C	2.669061	3.091831	5.778672	5.169216	4.951964
35	O	3.002817	3.436975	6.816376	6.270920	5.997682
		21	22	23	24	25
21	H	0.000000				
22	H	4.338939	0.000000			
23	C	3.329245	2.224877	0.000000		
24	H	4.338867	2.680348	1.079197	0.000000	
25	C	2.225272	3.336761	1.419248	2.229622	0.000000
26	H	2.690320	4.342656	2.228162	2.680685	1.078032
27	C	1.079045	3.331621	2.284811	3.327458	1.411655
28	C	5.138081	6.048699	5.649090	6.188999	5.351822
29	O	6.053053	6.660168	6.555286	7.088960	6.365738
30	C	4.327879	2.901942	3.327097	3.841262	3.806383
31	O	5.375456	3.433455	4.308513	4.710514	4.920974
32	C	2.763020	4.411972	3.903803	4.740707	3.379026
33	O	2.823836	4.892766	4.673979	5.614906	4.084361
34	C	5.348001	5.412330	4.542045	4.680830	4.520893

		26	27	28	29	30
35	O	6.511259	6.339304	5.634316	5.671853	5.701622

		26	27	28	29	30
26	H	0.000000				
27	C	2.229374	0.000000			
28	C	5.670442	5.070711	0.000000		
29	O	6.757160	5.990815	1.138778	0.000000	
30	C	4.607050	3.635177	3.309415	3.813159	0.000000
31	O	5.734221	4.707859	3.632276	3.808109	1.142481
32	C	3.907251	2.731654	2.446125	3.307476	2.559593
33	O	4.667150	3.079496	3.012272	3.615308	3.280270
34	C	4.644660	4.919648	2.477631	3.304821	3.119963
35	O	5.800284	6.098808	2.818800	3.318781	3.902135

		31	32	33	34	35
31	O	0.000000				
32	C	3.434197	0.000000			
33	O	3.979158	1.169788	0.000000		
34	C	3.652808	3.313358	4.404481	0.000000	
35	O	4.141148	4.300459	5.303975	1.195350	0.000000

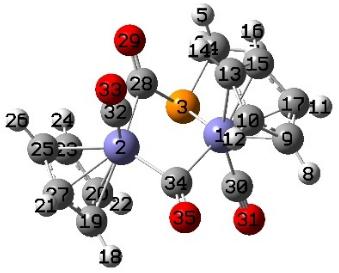

9S -3747.682032 26.72 C1
WBI 0.29

Charge and spin density

		1
1	Fe	-0.221906
2	Fe	0.005258
3	P	-0.008088
4	C	-0.055252
9	C	0.008366
10	C	0.055116
13	C	0.092230
15	C	0.089589
17	C	-0.031225
19	C	0.091917
20	C	0.077788
23	C	0.050159
25	C	0.025142
27	C	-0.019851
28	C	0.235979

		1	2	3	4	5
1	Fe	0.000000				
2	Fe	2.658764	0.000000			
3	P	2.375150	2.970893	0.000000		
4	C	3.506199	4.587942	1.855117	0.000000	
5	H	3.797626	4.679010	2.463864	1.092313	0.000000
6	H	4.443994	5.222531	2.416598	1.092723	1.772239
7	H	3.576274	5.217477	2.484038	1.091752	1.791283
8	H	2.815047	5.085839	4.982044	5.695047	6.015173
9	C	2.078729	4.471097	4.261152	4.866141	5.067629
10	C	2.114606	3.823627	4.342636	5.063939	5.021041
11	H	2.820445	5.439197	3.830202	3.714080	4.133255
12	H	2.864422	3.960590	5.114721	6.015097	5.943442
13	C	2.150726	3.643932	3.745887	4.140948	3.866662
14	H	2.914391	3.628149	4.119504	4.472527	3.975749
15	C	2.123236	4.212358	3.206422	3.144342	3.008023
16	H	2.877199	4.649145	3.147006	2.509828	2.158609
17	C	2.071713	4.675063	3.566023	3.712551	3.943860
18	H	4.312799	2.889008	4.816405	6.665676	7.095436
19	C	4.096620	2.144565	4.242479	6.073865	6.418566
20	C	3.865391	2.150038	3.243659	5.035774	5.468921
21	H	5.206712	2.844033	5.719845	7.389702	7.437652
22	H	3.909080	2.908472	3.023728	4.767949	5.395971
23	C	4.292015	2.130117	3.317562	4.870373	5.067311
24	H	4.670923	2.876039	3.166829	4.425269	4.587721
25	C	4.703025	2.082424	4.307043	5.822621	5.829672
26	H	5.380847	2.819858	4.940816	6.244896	6.076400
27	C	4.606662	2.108205	4.795303	6.498614	6.617900

29	O	-0.292876	28	C	2.584718	1.985129	1.815471	2.883762	2.770739
30	C	0.108918	29	O	3.482982	2.880135	2.745594	3.156529	2.617482
31	O	-0.096736	30	C	1.749811	3.457506	2.706917	4.012897	4.695241
32	C	0.028389	31	O	2.891074	4.322829	3.443959	4.684183	5.511288
33	O	-0.089227	32	C	3.311002	1.749466	4.127101	5.340146	5.091275
34	C	0.120629	33	O	4.130684	2.889456	5.088944	6.086239	5.674496
35	O	-0.174317	34	C	1.964031	1.868234	3.579300	5.143261	5.379423
			35	O	2.881572	2.863107	4.675711	6.231875	6.497520
					6	7	8	9	10
			6	H	0.000000				
			7	H	1.764309	0.000000			
			8	H	6.661161	5.272626	0.000000		
			9	C	5.887930	4.517507	1.079347	0.000000	
			10	C	6.122396	4.974333	2.224209	1.411234	0.000000
			11	H	4.610015	2.950343	2.700931	2.238496	3.333785
			12	H	7.052560	6.003331	2.672728	2.219425	1.079021
			13	C	5.213166	4.181218	3.340641	2.297745	1.421243
			14	H	5.487348	4.711268	4.351487	3.341430	2.239643
			15	C	4.235700	2.935302	3.340188	2.295439	2.283741
			16	H	3.561152	2.335617	4.350471	3.337619	3.323036
			17	C	4.731626	3.207892	2.240746	1.425828	2.290380
			18	H	7.133095	7.120318	5.781548	5.694252	5.440332
			19	C	6.497284	6.663238	6.033675	5.752204	5.361594
			20	C	5.300740	5.709221	6.194435	5.791252	5.551422
			21	H	7.921918	8.053129	6.956450	6.597483	5.769332
			22	H	4.925660	5.341170	6.116551	5.790316	5.809393
			23	C	5.073492	5.738013	6.905908	6.314387	5.849406
			24	H	4.445343	5.393921	7.415015	6.740923	6.332621
			25	C	6.171990	6.673227	7.146580	6.551282	5.818708
			26	H	6.577937	7.157985	7.847592	7.169751	6.293215
			27	C	6.961403	7.198831	6.649198	6.234303	5.533938
			28	C	3.536801	3.683260	5.359160	4.485839	3.974725
			29	O	3.763930	4.014911	6.101007	5.127215	4.445068
			30	C	4.701334	3.925938	3.000291	2.830889	3.471435
			31	O	5.200564	4.513979	3.573269	3.678498	4.491702
			32	C	6.116221	5.934608	5.340479	4.607205	3.495970
			33	O	6.903255	6.647719	5.829588	5.074032	3.778937
			34	C	5.951370	5.416377	3.503592	3.150890	2.697283
			35	O	7.046319	6.411667	3.478255	3.444869	3.066060
					11	12	13	14	15
			11	H	0.000000				
			12	H	4.342735	0.000000			
			13	C	3.335883	2.233477	0.000000		
			14	H	4.341053	2.700910	1.078365	0.000000	
			15	C	2.233276	3.326481	1.406697	2.214797	0.000000
			16	H	2.691445	4.331637	2.211158	2.656073	1.076615

17	C	1.079362	3.332847	2.293313	3.333196	1.418158
18	H	6.819769	5.295320	5.896085	6.154912	6.377131
19	C	6.749261	5.306723	5.559343	5.682688	6.039454
20	C	6.320501	5.762721	5.479334	5.631121	5.670033
21	H	8.016563	5.382187	5.940190	5.730094	6.831329
22	H	6.013196	6.150044	5.771159	6.080764	5.718667
23	C	6.787683	6.081045	5.469284	5.368976	5.728889
24	H	6.904733	6.699435	5.746081	5.606661	5.821791
25	C	7.415370	5.822742	5.507084	5.219485	6.092853
26	H	8.047791	6.263620	5.829411	5.348536	6.498590
27	C	7.405533	5.346198	5.579075	5.439090	6.291371
28	C	4.741269	4.532249	3.143773	3.051590	3.239799
29	O	5.324081	4.987421	3.329023	2.899399	3.494238
30	C	3.040877	4.078797	3.840538	4.662848	3.506669
31	O	3.626513	5.033552	4.957597	5.802292	4.533937
32	C	5.923292	3.385172	3.177241	2.732368	4.210554
33	O	6.520811	3.501745	3.412626	2.705324	4.630280
34	C	4.612880	2.689124	3.223523	3.629278	3.858828
35	O	5.194788	2.719615	3.964355	4.407057	4.690275
		16	17	18	19	20
16	H	0.000000				
17	C	2.229383	0.000000			
18	H	7.004909	6.260307	0.000000		
19	C	6.545496	6.146124	1.079085	0.000000	
20	C	5.979967	5.850276	2.230923	1.420264	0.000000
21	H	7.372565	7.195098	2.674442	2.223212	3.338463
22	H	5.989583	5.720009	2.691529	2.235088	1.078461
23	C	5.860659	6.234561	3.332934	2.290618	1.414128
24	H	5.754971	6.436831	4.342396	3.333588	2.225673
25	C	6.332672	6.696449	3.332160	2.288892	2.290051
26	H	6.649085	7.276164	4.342452	3.332360	3.332642
27	C	6.751361	6.658672	2.217172	1.407637	2.294641
28	C	3.237328	4.092489	4.665726	3.875215	3.190391
29	O	3.246436	4.640385	5.703024	4.848540	4.216498
30	C	4.138518	2.842048	3.946506	4.016867	3.690750
31	O	5.108550	3.695278	4.148918	4.400809	4.052129
32	C	4.678459	4.961978	4.155245	3.521970	3.830786
33	O	5.080520	5.495311	5.122977	4.543639	4.943299
34	C	4.656449	3.822333	2.753571	2.714557	3.166624
35	O	5.594761	4.433751	2.733400	3.083850	3.887523
		21	22	23	24	25
21	H	0.000000				
22	H	4.347243	0.000000			
23	C	3.342308	2.224453	0.000000		
24	H	4.352219	2.678462	1.078604	0.000000	
25	C	2.241115	3.331458	1.414722	2.226114	0.000000

	26 H 2.701473 4.339849 2.226430 2.680454 1.079411<br>27 C 1.079300 3.336658 2.299393 3.342479 1.428377<br>28 C 4.669583 3.563344 2.696048 2.673923 3.177277<br>29 O 5.252847 4.665846 3.420160 3.195150 3.663561<br>30 C 5.690977 3.311668 4.589342 5.001826 5.284682<br>31 O 6.288633 3.431708 5.148183 5.553652 5.955302<br>32 C 3.062142 4.654263 3.459220 4.060591 2.800354<br>33 O 3.639780 5.791145 4.469915 5.004337 3.636346<br>34 C 3.530191 3.569511 3.785469 4.566064 3.761018<br>35 O 3.652772 4.267971 4.665089 5.546938 4.483290<br>26 27 28 29 30<br>26 H 0.000000<br>27 C 2.241578 0.000000<br>28 C 3.548393 3.875560 0.000000<br>29 O 3.684134 4.571211 1.203142 0.000000<br>30 C 6.175471 4.995037 3.696540 4.793700 0.000000<br>31 O 6.922423 5.575996 4.660113 5.793335 1.141902<br>32 C 2.960360 2.867184 2.608713 2.858747 4.640033<br>33 O 3.513902 3.710161 3.477011 3.393548 5.597944<br>34 C 4.538110 3.146436 3.212048 4.161193 2.551954<br>35 O 5.264465 3.543374 4.373020 5.285719 3.125738<br>31 32 33 34 35<br>31 O 0.000000<br>32 C 5.651123 0.000000<br>33 O 6.644045 1.140707 0.000000<br>34 C 3.412835 2.563944 3.460486 0.000000<br>35 O 3.755486 3.313620 4.020607 1.168089 0.000000
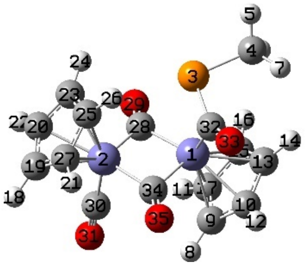<br>10S -3747.680739 27.54 C1<br>WBI 0.29<br><br>Charge and spin density<br>1<br>1 Fe -0.347827<br>2 Fe -0.017902<br>3 P 0.018615<br>4 C -0.024099<br>9 C 0.040622<br>10 C 0.136247	1 2 3 4 5<br>1 Fe 0.000000<br>2 Fe 2.561416 0.000000<br>3 P 2.374218 3.513387 0.000000<br>4 C 3.456383 5.250754 1.861862 0.000000<br>5 H 4.412940 5.907574 2.411880 1.092532 0.000000<br>6 H 3.589805 5.512538 2.475008 1.091246 1.766019<br>7 H 3.663898 5.710606 2.498590 1.089937 1.768435<br>8 H 2.871111 3.772483 5.238130 6.133710 7.169086<br>9 C 2.119095 3.710836 4.453830 5.161950 6.219767<br>10 C 2.098081 4.276284 4.029834 4.427353 5.495625<br>11 H 2.889219 3.880760 4.781529 5.460649 6.419832<br>12 H 2.815525 4.743904 4.508309 4.902697 5.917069<br>13 C 2.077326 4.622567 3.387662 3.321630 4.412470<br>14 H 2.817463 5.359226 3.409394 2.832424 3.869157<br>15 C 2.103207 4.342643 3.494743 3.584372 4.625125<br>16 H 2.843398 4.880513 3.596065 3.380284 4.308365<br>17 C 2.136202 3.773766 4.178054 4.749026 5.774575<br>18 H 5.302631 2.823706 6.161148 7.981132 8.524520

13	C	0.022740
15	C	-0.042886
17	C	0.085103
19	C	-0.011816
20	C	0.004694
23	C	0.078333
25	C	0.071241
27	C	0.094541
28	C	0.213885
29	O	-0.175144
30	C	0.001841
31	O	-0.094033
32	C	0.156782
33	O	-0.202729
34	C	0.158059
35	O	-0.166262

19	C	4.614843	2.081498	5.188308	7.031198	7.518388
20	C	4.479098	2.092794	4.694060	6.483819	6.882035
21	H	4.612370	2.854571	5.079033	6.840000	7.324568
22	H	5.075663	2.838377	5.357243	7.048342	7.436979
23	C	3.969570	2.124206	3.558514	5.353500	5.637293
24	H	4.167021	2.865613	3.276321	4.901408	5.049327
25	C	3.777847	2.129189	3.418953	5.260928	5.585379
26	H	3.839776	2.887374	3.002631	4.726757	4.953599
27	C	4.220848	2.117007	4.538672	6.376213	6.827744
28	C	1.938522	1.896657	2.735286	4.123116	4.835271
29	O	2.910549	2.875699	3.301931	4.405177	5.008010
30	C	3.220962	1.751625	4.891462	6.415254	7.209656
31	O	4.044663	2.894110	5.912894	7.322732	8.167876
32	C	1.969429	3.485247	1.774459	2.766858	3.525501
33	O	2.917592	4.333849	2.835414	3.459556	4.124221
34	C	1.946819	1.885121	3.467463	4.941010	5.755428
35	O	2.900369	2.879082	4.347941	5.696570	6.504318
		6	7	8	9	10
6	H	0.000000				
7	H	1.783378	0.000000			
8	H	6.092022	6.097974	0.000000		
9	C	5.061881	5.127067	1.079478	0.000000	
10	C	4.474467	4.136698	2.226591	1.415796	0.000000
11	H	5.013694	5.803575	2.684375	2.229699	3.338871
12	H	5.129529	4.428341	2.685195	2.230264	1.078913
13	C	3.186231	3.085759	3.326246	2.282876	1.414509
14	H	2.788158	2.320025	4.334069	3.324528	2.222361
15	C	3.116299	3.750249	3.332782	2.290543	2.301124
16	H	2.631943	3.738791	4.340598	3.332638	3.344465
17	C	4.395449	4.945740	2.228899	1.417353	2.296543
18	H	8.297312	8.435029	5.495282	5.899470	6.666714
19	C	7.393022	7.524628	5.346273	5.561898	6.186873
20	C	6.735591	7.153535	5.697687	5.699894	6.339950
21	H	7.457730	7.054024	5.364976	5.584161	5.784024
22	H	7.159682	7.797790	6.124976	6.141699	6.930570
23	C	5.727987	6.051656	5.846080	5.623652	5.971259
24	H	5.208443	5.735467	6.363011	5.981918	6.252790
25	C	5.846725	5.743137	5.596066	5.428939	5.557086
26	H	5.470551	5.121349	5.945020	5.655221	5.509378
27	C	6.916599	6.745068	5.291480	5.403430	5.715215
28	C	4.072911	4.763970	3.859179	3.355299	3.891752
29	O	4.125245	5.202061	4.671377	4.124715	4.730061
30	C	6.457927	6.815119	3.006765	3.314833	4.375698
31	O	7.260935	7.689639	3.027500	3.567313	4.829326
32	C	3.468982	2.683041	4.359720	3.688347	2.944808
33	O	4.261015	3.028952	4.768936	4.202803	3.208849

	34	C	5.336323	5.034649	2.817018	2.758404	2.918972
	35	O	6.180065	5.603827	3.037127	3.214459	3.187452
			11	12	13	14	15
	11	H	0.000000				
	12	H	4.347706	0.000000			
	13	C	3.331026	2.227868	0.000000		
	14	H	4.340389	2.678213	1.078374	0.000000	
	15	C	2.223494	3.343076	1.422059	2.233179	0.000000
	16	H	2.676255	4.353113	2.238756	2.697631	1.078900
	17	C	1.079308	3.339122	2.287095	3.329509	1.411933
	18	H	5.996951	6.934569	7.297502	8.081934	7.013297
	19	C	5.668326	6.488061	6.670401	7.377024	6.412551
	20	C	5.342565	6.829402	6.533159	7.205488	6.052860
	21	H	6.485777	5.717791	6.457590	6.988152	6.682117
	22	H	5.402677	7.525402	7.056516	7.781120	6.379275
	23	C	5.450068	6.431529	5.965260	6.465005	5.624032
	24	H	5.576556	6.804378	5.998504	6.401065	5.555123
	25	C	5.819792	5.791707	5.737025	6.159764	5.734315
	26	H	6.262295	5.639680	5.586817	5.828990	5.787059
	27	C	5.959479	5.844456	6.212224	6.776883	6.238108
	28	C	2.782302	4.693424	3.693521	4.412927	2.985077
	29	O	2.910429	5.633832	4.300888	4.969894	3.290183
	30	C	3.173304	4.931236	4.960415	5.911066	4.462654
	31	O	3.213393	5.396863	5.510873	6.534424	4.921197
	32	C	4.837827	3.071325	2.801879	2.849947	3.514965
	33	O	5.718347	2.927269	3.304613	3.157614	4.330332
	34	C	4.006596	3.076536	3.642166	4.325811	3.921268
	35	O	4.856043	2.952943	4.188277	4.789577	4.729456
			16	17	18	19	20
	16	H	0.000000				
	17	C	2.223149	0.000000			
	18	H	7.575010	6.155714	0.000000		
	19	C	6.914353	5.731948	1.079820	0.000000	
	20	C	6.344264	5.519724	2.239717	1.429677	0.000000
	21	H	7.363120	6.184323	2.679199	2.224170	3.345774
	22	H	6.563823	5.776782	2.699002	2.243032	1.078948
	23	C	5.839683	5.414385	3.334035	2.291148	1.411682
	24	H	5.574407	5.556867	4.346268	3.336106	2.225138
	25	C	6.138937	5.556517	3.330257	2.286049	2.292847
	26	H	6.173011	5.837958	4.341009	3.329801	3.333593
	27	C	6.806417	5.763298	2.223274	1.410896	2.302501
	28	C	3.226033	2.739916	4.504443	3.767146	3.122434
	29	O	3.172246	3.160445	5.151475	4.452931	3.505737
	30	C	5.091813	3.398859	2.865407	2.748778	2.954814
	31	O	5.561276	3.653887	3.388389	3.579582	3.838710
	32	C	4.072474	3.994360	6.018569	5.177415	5.165387

33	O	4.974456	4.790605	6.609615	5.833161	6.035459
34	C	4.754804	3.452538	3.979843	3.429978	3.889747
35	O	5.655208	4.235286	4.337307	3.959944	4.720106

		21	22	23	24	25
21	H	0.000000				
22	H	4.354371	0.000000			
23	C	3.339494	2.227137	0.000000		
24	H	4.345665	2.686017	1.078909	0.000000	
25	C	2.232207	3.336559	1.417196	2.225047	0.000000
26	H	2.692154	4.343242	2.226349	2.673964	1.079196
27	C	1.078979	3.344478	2.296821	3.337424	1.419067
28	C	4.650812	3.448791	2.793204	2.833892	3.309365
29	O	5.647051	3.507201	3.253915	2.982692	4.116173
30	C	3.856102	3.230595	3.606217	4.291713	3.800224
31	O	4.739740	3.889346	4.665583	5.322026	4.914502
32	C	4.452868	5.995471	4.239601	4.383913	3.563879
33	O	4.674494	6.940361	5.135780	5.374263	4.216928
34	C	2.908790	4.708855	3.647836	4.307726	2.971515
35	O	2.850016	5.589851	4.546156	5.294513	3.623466

		26	27	28	29	30
26	H	0.000000				
27	C	2.233041	0.000000			
28	C	3.756207	3.871864	0.000000		
29	O	4.585360	4.769315	1.168235	0.000000	
30	C	4.610885	3.338642	2.593353	3.300471	0.000000
31	O	5.742959	4.324178	3.487194	3.999369	1.142856
32	C	3.068246	4.274343	3.351292	4.291742	4.706679
33	O	3.580187	4.756702	4.469674	5.452934	5.484026
34	C	3.200448	2.822215	2.849163	4.012815	2.620376
35	O	3.734358	3.183746	4.012626	5.173448	3.370001

		31	32	33	34	35
31	O	0.000000				
32	C	5.673856	0.000000			
33	O	6.401895	1.181676	0.000000		
34	C	3.525770	2.500404	2.985890	0.000000	
35	O	4.094922	3.022604	3.058618	1.166403	0.000000

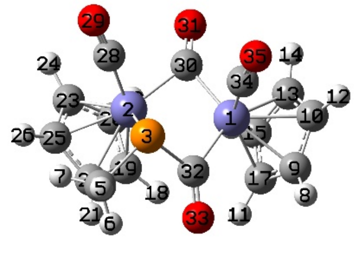

11S -3747.67865 28.85 C1
WBI 0.28

		1	2	3	4	5
1	Fe	0.000000				
2	Fe	2.661724	0.000000			
3	P	3.041293	2.376231	0.000000		
4	C	4.626019	3.468385	1.851237	0.000000	
5	H	5.318919	4.408899	2.422248	1.092297	0.000000
6	H	4.683800	3.788429	2.447466	1.092565	1.773254
7	H	5.208978	3.486753	2.483899	1.091107	1.765636
8	H	2.817148	5.117197	4.595180	5.676270	6.116452
9	C	2.092251	4.545720	4.502917	5.772991	6.350431

Charge and spin density

		1
1	Fe	-0.094314
2	Fe	-0.180667
3	P	0.029701
4	C	-0.047235
9	C	0.043034
10	C	0.023242
13	C	0.093211
15	C	0.001638
17	C	0.044711
19	C	0.048827
20	C	0.002166
23	C	0.022912
25	C	-0.021019
27	C	0.080128
28	C	0.094697
29	O	-0.088837
30	C	0.242169
31	O	-0.174070
32	C	0.280569
33	O	-0.318641
34	C	-0.004379
35	O	-0.077841

10	C	2.105181	4.736229	4.903541	6.462496	7.013855
11	H	2.846898	4.127166	4.662754	5.435176	6.253857
12	H	2.842423	5.445596	5.323833	6.951252	7.369977
13	C	2.137941	4.321383	5.166218	6.748252	7.452824
14	H	2.871809	4.722302	5.754818	7.427757	8.126155
15	C	2.135603	3.811463	4.951759	6.275445	7.110636
16	H	2.886777	3.829453	5.413048	6.624775	7.551043
17	C	2.121566	3.982302	4.550558	5.659694	6.432735
18	H	3.340893	2.902241	4.303537	4.814723	5.850611
19	C	3.438881	2.135860	3.862433	4.341209	5.417670
20	C	3.772428	2.103067	4.387653	5.115929	6.167603
21	H	4.377710	2.866119	3.237089	2.729110	3.778143
22	H	3.958975	2.852749	5.167821	6.088931	7.120273
23	C	4.486292	2.075954	4.218612	4.735139	5.737315
24	H	5.180592	2.814717	4.891608	5.471096	6.399190
25	C	4.605556	2.068569	3.516695	3.551100	4.565736
26	H	5.391374	2.819917	3.718701	3.391421	4.275901
27	C	4.019681	2.120391	3.277475	3.250740	4.342165
28	C	3.633269	1.750299	2.628481	3.818476	4.488824
29	O	4.564750	2.890180	3.356880	4.434704	4.912121
30	C	1.899857	1.937395	3.427172	5.021842	5.810224
31	O	2.871500	2.877895	4.476044	6.079264	6.842652
32	C	1.989515	2.591645	1.811001	2.894034	3.587037
33	O	2.871247	3.485617	2.721887	3.137477	3.794090
34	C	1.768157	3.423444	2.890246	4.693948	5.081209
35	O	2.900276	4.330235	3.470925	5.222178	5.404263

		6	7	8	9	10
6	H	0.000000				
7	H	1.788146	0.000000			
8	H	5.323836	6.572318	0.000000		
9	C	5.504744	6.553645	1.078396	0.000000	
10	C	6.402811	7.179451	2.237774	1.420561	0.000000
11	H	4.898394	6.012506	2.657491	2.212889	3.325486
12	H	6.979395	7.721822	2.694347	2.230827	1.080011
13	C	6.730096	7.286851	3.341010	2.298124	1.411168
14	H	7.522091	7.887827	4.350958	3.340957	2.226718
15	C	6.098051	6.738206	3.327499	2.286767	2.282159
16	H	6.448274	6.932241	4.337596	3.330761	3.323816
17	C	5.302197	6.272082	2.224918	1.415433	2.288921
18	H	4.408201	4.983192	4.600490	4.020372	4.747648
19	C	4.126372	4.323414	5.201970	4.608154	5.170773
20	C	5.155115	4.936111	6.039712	5.278336	5.486918
21	H	2.275098	2.652497	5.575854	5.384930	6.236846
22	H	6.128107	5.966596	6.243341	5.355495	5.371909
23	C	4.994801	4.297205	6.909440	6.239720	6.410145
24	H	5.867813	4.938914	7.764784	7.046050	7.047270

25	C	3.777025	3.001435	6.716494	6.245422	6.657794
26	H	3.785578	2.567233	7.453046	7.070032	7.484824
27	C	3.085092	3.049528	5.695053	5.304576	5.960545
28	C	4.545077	3.676881	6.212982	5.688184	5.577265
29	O	5.309929	4.203938	7.116543	6.620268	6.376191
30	C	5.340171	5.246599	4.705887	3.882472	3.512209
31	O	6.460850	6.215147	5.580920	4.674009	4.015474
32	C	2.768655	3.658524	2.994414	2.900442	3.647058
33	O	2.579421	3.954873	2.857547	3.029368	4.140926
34	C	5.045350	5.360275	3.452627	3.084320	2.679709
35	O	5.699948	5.902889	4.174310	3.958894	3.396456
		11	12	13	14	15
11	H	0.000000				
12	H	4.332808	0.000000			
13	C	3.342812	2.222293	0.000000		
14	H	4.352493	2.681237	1.078635	0.000000	
15	C	2.234706	3.326721	1.420439	2.233934	0.000000
16	H	2.700317	4.334319	2.227205	2.683210	1.079470
17	C	1.077689	3.332175	2.296944	3.338414	1.410803
18	H	2.272769	5.774899	4.247510	4.966871	2.963428
19	C	3.185602	6.132789	4.604396	5.187830	3.490243
20	C	4.186395	6.393459	4.633637	4.928258	3.715011
21	H	4.066540	7.070102	6.108742	6.835180	5.132424
22	H	4.376439	6.280822	4.299687	4.428211	3.437459
23	C	5.307937	7.210985	5.661799	5.880654	4.923188
24	H	6.270080	7.779207	6.209022	6.255590	5.621001
25	C	5.229303	7.440865	6.180379	6.589996	5.385716
26	H	6.151359	8.196311	7.096024	7.487252	6.382376
27	C	4.027004	6.824914	5.613633	6.221193	4.642120
28	C	5.730259	6.030308	5.220647	5.365069	5.108315
29	O	6.834236	6.691508	6.047317	6.059297	6.099474
30	C	4.145896	4.127948	2.832383	2.951436	2.838390
31	O	5.034822	4.515624	3.085441	2.779839	3.336199
32	C	2.931437	4.276242	4.016664	4.847615	3.597544
33	O	2.570483	4.828853	4.611609	5.566455	3.971519
34	C	4.453147	2.743488	3.150725	3.552792	3.734529
35	O	5.531855	3.098032	3.993251	4.224571	4.777021
		16	17	18	19	20
16	H	0.000000				
17	C	2.225177	0.000000			
18	H	2.693656	2.772731	0.000000		
19	C	3.162607	3.505143	1.076709	0.000000	
20	C	3.149267	4.220809	2.238843	1.424724	0.000000
21	H	5.120387	4.620423	2.652760	2.211490	3.331805
22	H	2.659002	4.260087	2.706157	2.241088	1.079160
23	C	4.472995	5.346515	3.334514	2.292999	1.409023

24	H	5.118646	6.208488	4.346101	3.337199	2.222221
25	C	5.129878	5.444288	3.326298	2.287706	2.292080
26	H	6.180806	6.377482	4.335367	3.331032	3.334078
27	C	4.481956	4.426033	2.211328	1.406060	2.292252
28	C	5.182607	5.419139	4.645877	3.816594	3.400824
29	O	6.182586	6.466930	5.780023	4.924495	4.392434
30	C	2.983156	3.530905	3.610162	3.238940	2.818228
31	O	3.296650	4.333262	4.450232	4.059069	3.284712
32	C	4.202024	2.878418	3.196241	3.213273	4.083016
33	O	4.558421	2.902171	3.159075	3.442578	4.606488
34	C	4.514703	3.710879	4.973981	4.876292	5.057174
35	O	5.570452	4.768546	6.101420	5.954831	6.065244
		21	22	23	24	25
21	H	0.000000				
22	H	4.341144	0.000000			
23	C	3.343070	2.217098	0.000000		
24	H	4.355031	2.669860	1.079550	0.000000	
25	C	2.234352	3.334232	1.426618	2.239787	0.000000
26	H	2.698709	4.341932	2.237366	2.696082	1.079266
27	C	1.077883	3.335759	2.298692	3.342558	1.418678
28	C	4.183080	3.981210	2.771011	2.913506	2.847611
29	O	5.170390	4.893971	3.585278	3.428842	3.690834
30	C	4.594042	2.854421	3.286855	3.684938	3.871150
31	O	5.612395	2.992321	3.695143	3.795227	4.579941
32	C	3.035410	4.714504	4.491852	5.370875	3.980906
33	O	2.907468	5.266902	5.126254	6.106887	4.463037
34	C	5.385415	5.298137	5.402465	5.907994	5.419362
35	O	6.318765	6.296540	6.271616	6.667833	6.266010
		26	27	28	29	30
26	H	0.000000				
27	C	2.233966	0.000000			
28	C	3.070839	3.531120	0.000000		
29	O	3.655107	4.560036	1.141444	0.000000	
30	C	4.650666	3.852615	2.474462	3.333371	0.000000
31	O	5.328512	4.769783	2.990978	3.576080	1.167680
32	C	4.562439	3.141855	3.693078	4.664731	3.216323
33	O	5.040086	3.332682	4.764715	5.761938	4.247293
34	C	5.958677	5.115610	3.571732	4.151834	2.593702
35	O	6.681043	6.087016	4.085452	4.389322	3.486893
		31	32	33	34	35
31	O	0.000000				
32	C	4.374470	0.000000			
33	O	5.399183	1.207755	0.000000		
34	C	3.317009	2.669908	3.727287	0.000000	
35	O	4.000980	3.606829	4.628036	1.140226	0.000000

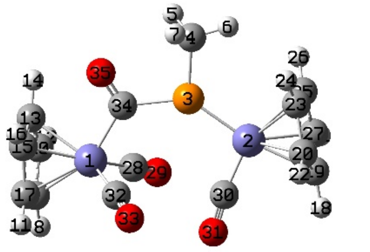

12S -3747.676879 29.96 C1
WBI 0.02

Charge and spin density

		1
1	Fe	0.144688
2	Fe	0.263376
3	P	-0.020137
4	C	-0.017865
9	C	0.013811
10	C	0.038719
13	C	0.072945
15	C	0.029137
17	C	0.046302
19	C	0.007704
20	C	-0.025289
23	C	0.030957
25	C	0.019076
27	C	-0.024262
28	C	-0.068908
29	O	-0.068063
30	C	-0.113624
31	O	-0.109882
32	C	-0.028697
33	O	-0.070062
34	C	0.168751
35	O	-0.288676

		1	2	3	4	5
1	Fe	0.000000				
2	Fe	4.614058	0.000000			
3	P	3.426386	2.090847	0.000000		
4	C	4.771642	3.420846	1.833379	0.000000	
5	H	4.843249	4.187893	2.440539	1.091084	0.000000
6	H	5.681181	3.368840	2.419646	1.089535	1.780631
7	H	4.991844	4.044463	2.440895	1.091151	1.761038
8	H	2.880714	6.882676	6.142249	7.579220	7.539238
9	C	2.129244	6.542175	5.519473	6.813884	6.733792
10	C	2.114487	6.400333	5.086931	6.135987	5.854632
11	H	2.861290	6.909489	5.961382	7.217143	7.378454
12	H	2.866737	6.635527	5.400184	6.401163	6.002331
13	C	2.113216	6.345072	4.702951	5.449292	5.144622
14	H	2.829253	6.480493	4.638293	5.034368	4.556533
15	C	2.101318	6.426625	4.904105	5.756323	5.683456
16	H	2.841073	6.673401	5.059021	5.683364	5.672719
17	C	2.116427	6.558392	5.414181	6.602748	6.637730
18	H	6.738596	2.821693	4.901112	6.152606	6.936524
19	C	6.449687	2.072527	4.145254	5.212822	6.016360
20	C	6.426629	2.069337	3.816273	4.622448	5.580825
21	H	6.681156	2.828160	4.412119	5.314032	5.776516
22	H	6.709663	2.824986	4.374382	5.187285	6.213095
23	C	6.403777	2.083460	3.263382	3.572750	4.506854
24	H	6.651500	2.834361	3.407081	3.242326	4.239418
25	C	6.393056	2.083489	3.278883	3.632282	4.313809
26	H	6.628566	2.832279	3.432104	3.363026	3.836279
27	C	6.411640	2.072232	3.840065	4.698907	5.327883
28	C	1.765174	3.959273	3.326024	4.901973	4.954029
29	O	2.898406	4.061758	3.839986	5.398483	5.414731
30	C	3.944970	1.751588	2.968647	4.706140	5.350324
31	O	4.043692	2.891478	3.953655	5.749441	6.339713
32	C	1.760720	4.100804	3.441577	4.967818	5.401974
33	O	2.896777	4.248547	3.969524	5.460796	6.063087
34	C	1.974632	3.722663	1.888423	2.845992	2.963510
35	O	2.798789	4.647035	2.617973	2.780984	2.672575

		6	7	8	9	10
6	H	0.000000				
7	H	1.780996	0.000000			
8	H	8.444308	7.863614	0.000000		
9	C	7.742885	7.024214	1.078927	0.000000	
10	C	7.084184	6.424254	2.242124	1.427851	0.000000
11	H	8.167136	7.128867	2.678059	2.219630	3.330637
12	H	7.279365	6.834930	2.698739	2.238190	1.079724
13	C	6.484907	5.547300	3.342639	2.300278	1.410106
14	H	6.075925	5.125529	4.352244	3.342740	2.224498

15	C	6.805299	5.650040	3.344218	2.300471	2.288573
16	H	6.746046	5.374516	4.353591	3.342731	3.329667
17	C	7.584019	6.597566	2.225173	1.409133	2.285689
18	H	5.861406	6.726164	8.342458	8.318338	8.477815
19	C	4.838817	5.805348	8.362164	8.206718	8.215136
20	C	4.222019	4.996627	8.648408	8.348186	8.348251
21	H	4.877127	6.213620	8.408655	8.293401	8.023779
22	H	4.877397	5.363013	8.891105	8.594015	8.732266
23	C	2.965065	4.018946	8.877300	8.445340	8.213650
24	H	2.572391	3.455425	9.293804	8.756084	8.471743
25	C	2.964706	4.398387	8.722255	8.346141	7.974597
26	H	2.569652	4.241221	9.007369	8.570153	8.022720
27	C	4.222689	5.494140	8.389820	8.186417	7.963970
28	C	5.591291	5.461795	3.172081	2.912786	2.750163
29	O	5.929818	6.114071	3.713964	3.706278	3.499279
30	C	4.923550	5.232946	5.684173	5.553805	5.734712
31	O	6.040013	6.229250	5.217314	5.283537	5.711614
32	C	5.752071	5.023193	3.738001	3.257536	3.764752
33	O	6.142198	5.403118	4.546262	4.188818	4.854235
34	C	3.826704	3.041712	4.826635	4.007427	3.505430
35	O	3.866882	2.690612	5.515678	4.564122	3.882648
		11	12	13	14	15
11	H	0.000000				
12	H	4.340466	0.000000			
13	C	3.334033	2.222306	0.000000		
14	H	4.339415	2.681144	1.077701	0.000000	
15	C	2.234278	3.331541	1.415179	2.220079	0.000000
16	H	2.697002	4.338325	2.226113	2.671389	1.078403
17	C	1.080105	3.328825	2.290753	3.330535	1.424114
18	H	8.574337	8.649442	8.707599	9.021595	8.672583
19	C	8.500139	8.387187	8.310040	8.510097	8.335324
20	C	8.364360	8.658561	8.211184	8.351721	8.097562
21	H	9.137689	7.907233	8.287232	8.357596	8.679115
22	H	8.333024	9.152210	8.539960	8.747370	8.248477
23	C	8.617543	8.466703	7.937179	7.895258	7.975273
24	H	8.790041	8.792025	8.017401	7.887281	8.001128
25	C	8.877391	8.048243	7.848827	7.756172	8.121378
26	H	9.265098	8.007318	7.846996	7.617221	8.272274
27	C	8.798364	7.984125	8.073865	8.138715	8.333099
28	C	4.229484	2.891428	3.350333	3.876455	3.779449
29	O	5.206607	3.318203	4.295452	4.740301	4.863356
30	C	5.881920	6.022768	5.959688	6.375604	5.903312
31	O	5.557358	6.017205	6.130322	6.710718	5.979204
32	C	2.851701	4.557492	3.658998	4.352483	3.033981
33	O	3.295013	5.667861	4.711699	5.396536	3.897566
34	C	4.454770	4.029824	2.883003	2.894227	3.064409

35	O	4.882621	4.428782	2.870482	2.525776	3.084678
		16	17	18	19	20
16	H	0.000000				
17	C	2.240357	0.000000			
18	H	8.998657	8.444020	0.000000		
19	C	8.599186	8.282961	1.079804	0.000000	
20	C	8.184467	8.196224	2.229002	1.419360	0.000000
21	H	9.116239	8.691029	2.686236	2.232292	3.345769
22	H	8.247438	8.296598	2.685832	2.232770	1.078672
23	C	8.012460	8.303486	3.333601	2.290148	1.420647
24	H	7.901523	8.478480	4.342491	3.332854	2.229543
25	C	8.311633	8.432451	3.332838	2.289042	2.295973
26	H	8.465041	8.713632	4.341217	3.331291	3.339211
27	C	8.659148	8.410396	2.228559	1.418378	2.303566
28	C	4.587036	3.551332	5.784021	5.579030	5.921643
29	O	5.706619	4.555789	5.527064	5.404547	6.005293
30	C	6.314957	5.663607	2.804677	2.709756	3.103898
31	O	6.483295	5.466233	3.206208	3.461259	3.988916
32	C	3.360807	2.748742	5.889774	5.707602	5.522255
33	O	4.023305	3.522644	5.675049	5.584571	5.275511
34	C	3.267944	3.773365	6.360143	5.777441	5.485015
35	O	3.002471	4.165592	7.382439	6.711522	6.265126
		21	22	23	24	25
21	H	0.000000				
22	H	4.353994	0.000000			
23	C	3.340302	2.235612	0.000000		
24	H	4.350194	2.689109	1.079325	0.000000	
25	C	2.235860	3.338999	1.413587	2.227259	0.000000
26	H	2.687822	4.349145	2.227328	2.685304	1.079312
27	C	1.078673	3.345492	2.297495	3.340631	1.420971
28	C	5.401402	6.407424	5.956327	6.455122	5.622122
29	O	4.872291	6.606645	6.075184	6.716566	5.516511
30	C	3.533677	3.505502	3.689285	4.441733	3.696754
31	O	4.261955	4.222550	4.760935	5.524107	4.771126
32	C	6.510010	5.560442	5.792527	6.041275	6.104229
33	O	6.750416	5.101204	5.752593	5.981223	6.278951
34	C	6.009282	5.860278	5.096138	5.134831	5.151273
35	O	6.919251	6.607889	5.691821	5.520686	5.809977
		26	27	28	29	30
26	H	0.000000				
27	C	2.228848	0.000000			
28	C	5.854917	5.365419	0.000000		
29	O	5.725000	5.053353	1.138870	0.000000	
30	C	4.451312	3.121074	3.139441	3.213765	0.000000
31	O	5.538646	4.011195	3.198085	3.205155	1.146247
32	C	6.595636	6.047034	2.686702	3.676466	3.209112

33	O	6.911512	6.178841	3.669173	4.538352	3.293175
34	C	5.235010	5.570531	2.715243	3.645020	3.866458
35	O	5.746565	6.441426	3.784200	4.706597	5.010954

		31	32	33	34	35
31	O	0.000000				
32	C	3.198106	0.000000			
33	O	3.182601	1.138926	0.000000		
34	C	4.482983	2.591355	3.467169	0.000000	
35	O	5.667468	3.500692	4.300586	1.206683	0.000000

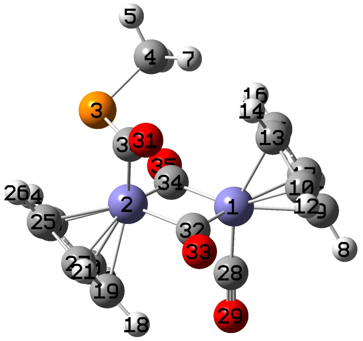

13S -3747.676108 30.4 C1
WBI 0.28

Charge and spin density

		1
1	Fe	-0.015498
2	Fe	-0.340877
3	P	0.018929
4	C	-0.005501
9	C	-0.014822
10	C	0.079072
13	C	0.065198
15	C	0.053325
17	C	0.007925
19	C	0.040806
20	C	0.099243
23	C	0.015415
25	C	0.036836
27	C	0.086631
28	C	0.005780
29	O	-0.094813
30	C	0.169145
31	O	-0.205534
32	C	0.196720
33	O	-0.168553
34	C	0.142430
35	O	-0.171857

		1	2	3	4	5
1	Fe	0.000000				
2	Fe	2.570526	0.000000			
3	P	4.005864	2.351495	0.000000		
4	C	4.076182	3.579206	1.853733	0.000000	
5	H	5.158857	4.487645	2.395219	1.092808	0.000000
6	H	4.025484	3.829434	2.448966	1.091422	1.767851
7	H	3.733827	3.762006	2.507457	1.087306	1.767606
8	H	2.827051	5.298805	6.773484	6.501816	7.534176
9	C	2.084310	4.627561	5.856383	5.464735	6.481252
10	C	2.115748	4.245514	5.267673	4.912416	5.861274
11	H	2.844669	5.102010	5.955082	5.282315	6.260101
12	H	2.849948	4.622394	5.760381	5.588933	6.486532
13	C	2.125999	3.830860	4.247679	3.593010	4.500317
14	H	2.881412	3.905549	3.880285	3.189022	3.946020
15	C	2.127472	4.029641	4.340171	3.444085	4.399498
16	H	2.877418	4.259593	4.058127	2.845495	3.711533
17	C	2.096002	4.508614	5.365566	4.718798	5.720056
18	H	3.676030	2.878441	5.193884	6.407742	7.357419
19	C	3.652911	2.125668	4.312533	5.689543	6.576621
20	C	3.811571	2.157656	3.950703	5.286953	6.144037
21	H	4.649973	2.826325	4.375043	5.912222	6.609637
22	H	3.951245	2.910575	4.603323	5.699992	6.594941
23	C	4.403529	2.120333	3.084860	4.672852	5.354346
24	H	4.983068	2.854035	3.093320	4.561655	5.115503
25	C	4.624601	2.083856	2.976941	4.762564	5.364303
26	H	5.362537	2.816644	2.903715	4.750722	5.151469
27	C	4.212939	2.102568	3.814919	5.413323	6.156548
28	C	1.750749	3.195897	5.159391	5.542205	6.630382
29	O	2.893284	4.015794	6.094025	6.587725	7.668605
30	C	3.435103	1.986787	1.774203	2.810966	3.470078
31	O	3.969796	2.960542	2.833636	3.538158	4.054467
32	C	1.874057	1.960740	3.789696	4.488920	5.459000
33	O	2.872560	2.903569	4.629166	5.387879	6.281606
34	C	1.915718	1.914942	2.898111	3.224267	4.268749
35	O	2.874516	2.905599	3.345971	3.412063	4.349989

		6	7	8	9	10

6	H	0.000000				
7	H	1.785422	0.000000			
8	H	6.349608	5.896938	0.000000		
9	C	5.342682	4.830225	1.079767	0.000000	
10	C	5.068881	4.126420	2.221929	1.409528	0.000000
11	H	4.722598	4.934875	2.699887	2.243280	3.343034
12	H	5.895546	4.765371	2.679290	2.223811	1.078856
13	C	3.788073	2.748647	3.330009	2.285945	1.418986
14	H	3.671332	2.210448	4.333962	3.324986	2.222487
15	C	3.213802	2.849216	3.333505	2.290660	2.296100
16	H	2.396902	2.429356	4.341314	3.332344	3.338823
17	C	4.360511	4.222416	2.239976	1.429644	2.300936
18	H	6.524486	6.450850	5.288023	5.177410	5.155349
19	C	5.840411	5.868138	5.760803	5.457719	5.319488
20	C	5.233071	5.684002	6.136889	5.745269	5.786001
21	H	6.443098	5.959871	6.743316	6.336629	5.702603
22	H	5.442879	6.112439	6.020896	5.719030	6.015670
23	C	4.742723	5.253577	7.033887	6.469124	6.295635
24	H	4.503549	5.324460	7.660629	7.034360	6.917008
25	C	5.140776	5.200424	7.248373	6.658381	6.203262
26	H	5.278922	5.244078	8.043381	7.375686	6.775133
27	C	5.807085	5.602739	6.526621	6.083238	5.621222
28	C	5.338376	5.378713	2.856377	2.737141	3.338392
29	O	6.321272	6.489616	3.364722	3.556825	4.320284
30	C	3.589086	2.763266	6.003503	5.149563	4.269998
31	O	4.437164	3.199458	6.188336	5.363478	4.243991
32	C	4.829092	4.170268	3.952500	3.406403	2.797727
33	O	5.851993	4.970306	4.313571	3.933682	3.144306
34	C	2.928348	3.427118	4.540854	3.805088	3.894360
35	O	2.765644	3.823903	5.173557	4.466887	4.764451

		11	12	13	14	15
11	H	0.000000				
12	H	4.353611	0.000000			
13	C	3.336083	2.230166	0.000000		
14	H	4.349050	2.672764	1.079067	0.000000	
15	C	2.225477	3.338092	1.417905	2.237972	0.000000
16	H	2.673333	4.347556	2.231506	2.701793	1.077978
17	C	1.078963	3.344582	2.292475	3.337546	1.410489
18	H	5.998480	5.215308	5.518651	5.897123	5.775537
19	C	6.086007	5.477392	5.402653	5.653544	5.615125
20	C	5.838234	6.178782	5.627807	5.923503	5.513020
21	H	7.446728	5.532844	5.728121	5.603639	6.399683
22	H	5.492384	6.518747	5.910960	6.361648	5.565519
23	C	6.504915	6.698128	5.853427	5.920949	5.787548
24	H	6.766908	7.426530	6.309471	6.351945	6.068742
25	C	7.116523	6.404555	5.797931	5.669104	6.067650

	26	H	7.856888	6.937911	6.230765	5.922390	6.581971
	27	C	6.893474	5.652688	5.532527	5.509431	5.977857
	28	C	3.200227	3.863432	3.793933	4.605981	3.589613
	29	O	3.837713	4.747956	4.905360	5.737883	4.640538
	30	C	5.915532	4.481382	3.520032	3.032030	4.158046
	31	O	6.478580	4.204591	3.624146	2.925807	4.591773
	32	C	4.700380	2.874939	2.959565	3.184676	3.645389
	33	O	5.580360	2.795417	3.589052	3.682535	4.527954
	34	C	3.510386	4.660333	3.331513	3.772110	2.841066
	35	O	3.543457	5.636420	4.098924	4.566179	3.252486
			16	17	18	19	20
	16	H	0.000000				
	17	C	2.216906	0.000000			
	18	H	6.342171	5.570879	0.000000		
	19	C	6.027188	5.640316	1.079412	0.000000	
	20	C	5.704844	5.576613	2.228212	1.415888	0.000000
	21	H	6.842074	6.740557	2.681986	2.231726	3.342326
	22	H	5.728622	5.428112	2.684542	2.228697	1.079397
	23	C	5.785467	6.168273	3.326583	2.283790	1.408127
	24	H	5.876585	6.530317	4.336919	3.327252	2.222995
	25	C	6.173327	6.582335	3.323348	2.281043	2.289810
	26	H	6.596165	7.268557	4.335502	3.326492	3.331482
	27	C	6.325470	6.293357	2.227456	1.419415	2.300839
	28	C	4.278297	2.929117	2.871704	3.213068	3.372424
	29	O	5.298075	3.799052	2.892001	3.459817	3.598375
	30	C	4.294495	5.096325	4.486203	3.803261	4.052861
	31	O	4.861318	5.543326	5.016778	4.487895	5.004064
	32	C	4.326641	3.876715	2.775099	2.716518	3.471475
	33	O	5.294613	4.702214	3.018310	3.171606	4.240130
	34	C	2.897222	3.174845	3.837987	3.356082	2.818672
	35	O	2.987079	3.524286	4.694752	4.179010	3.300240
			21	22	23	24	25
	21	H	0.000000				
	22	H	4.350556	0.000000			
	23	C	3.344305	2.221376	0.000000		
	24	H	4.350743	2.680273	1.078335	0.000000	
	25	C	2.228985	3.334245	1.424968	2.234700	0.000000
	26	H	2.688266	4.342041	2.233951	2.686243	1.079914
	27	C	1.078827	3.343296	2.299826	3.339761	1.411516
	28	C	4.817838	3.184385	4.448802	5.112818	4.907763
	29	O	5.287181	3.186385	4.876094	5.545149	5.441589
	30	C	3.285296	4.870637	3.551505	4.052889	2.897659
	31	O	3.475465	5.858216	4.589369	5.166545	3.715017
	32	C	2.964097	4.056233	3.925370	4.775334	3.593326
	33	O	2.780585	4.901740	4.696666	5.634351	4.090708
	34	C	4.671890	2.885071	3.082961	3.357451	3.734317

35	O	5.664351	3.075882	3.461525	3.394882	4.405812
		26	27	28	29	30
26	H	0.000000				
27	C	2.224950	0.000000			
28	C	5.862014	4.279820	0.000000		
29	O	6.469361	4.731413	1.143184	0.000000	
30	C	2.930595	3.101499	4.690591	5.677292	0.000000
31	O	3.624390	3.649031	5.294766	6.292851	1.182411
32	C	4.268542	2.833269	2.635884	3.552466	2.562254
33	O	4.672222	3.064391	3.417051	4.165011	3.116945
34	C	4.445445	3.888246	2.595174	3.487737	3.206355
35	O	5.064299	4.784276	3.301188	4.005084	4.118793
		31	32	33	34	35
31	O	0.000000				
32	C	2.835576	0.000000			
33	O	2.978390	1.164698	0.000000		
34	C	4.196135	2.842503	4.005463	0.000000	
35	O	5.172823	4.009176	5.171653	1.169592	0.000000

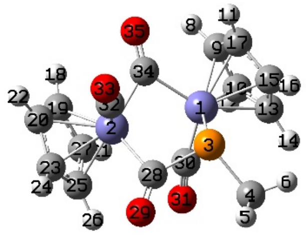

14S -3747.676051 30.48 C1
WBI 0.03

Charge and spin density

		1
1	Fe	0.109436
2	Fe	0.131039
3	P	-0.055534
4	C	-0.018366
9	C	0.022955
10	C	0.004448
13	C	-0.008769
15	C	0.023915
17	C	0.022877
19	C	0.025929
20	C	0.072336
23	C	0.045322
25	C	0.047279
27	C	0.000592
28	C	-0.033904

		1	2	3	4	5
1	Fe	0.000000				
2	Fe	2.778149	0.000000			
3	P	2.283465	2.987068	0.000000		
4	C	3.437596	4.475034	1.849225	0.000000	
5	H	4.368788	4.910164	2.423160	1.092176	0.000000
6	H	3.748561	5.279519	2.438887	1.092420	1.769392
7	H	3.507174	4.560798	2.491739	1.091614	1.774038
8	H	2.858575	4.510756	4.996399	6.212674	7.180008
9	C	2.110193	4.330676	4.213304	5.280368	6.281690
10	C	2.100256	4.620147	4.168590	4.777002	5.846019
11	H	2.873342	4.625492	3.915433	5.264703	6.081654
12	H	2.848845	5.033652	4.928803	5.384126	6.444638
13	C	2.082316	4.829087	3.445881	3.702947	4.788954
14	H	2.830581	5.400061	3.736562	3.442690	4.514810
15	C	2.116614	4.718379	3.025005	3.676836	4.661720
16	H	2.864930	5.193042	2.941476	3.367028	4.232280
17	C	2.121614	4.395033	3.555062	4.702316	5.633123
18	H	4.323728	2.905573	5.612212	7.092309	7.693774
19	C	4.082749	2.155354	5.044679	6.439799	6.951699
20	C	4.691958	2.091628	5.030090	6.474187	6.780571
21	H	3.594544	2.915174	4.924783	5.804457	6.474142
22	H	5.387007	2.829815	5.604794	7.167170	7.413095
23	C	4.720621	2.062548	4.620580	5.774863	5.941946
24	H	5.424378	2.782082	4.882688	5.920375	5.888012
25	C	4.141516	2.118868	4.379047	5.269461	5.590163
26	H	4.421865	2.847215	4.420799	4.928598	5.176395
27	C	3.685513	2.156511	4.642956	5.711397	6.253807

29	O	-0.142999
30	C	0.203334
31	O	-0.280751
32	C	0.000438
33	O	-0.080365
34	C	0.022102
35	O	-0.111312

28	C	3.055165	2.012295	1.812095	2.840147	3.018592
29	O	4.169475	2.882978	2.750666	3.193630	3.015052
30	C	1.747637	2.835177	2.795456	3.300554	4.156908
31	O	2.886943	3.538387	3.585883	3.624596	4.365320
32	C	3.669798	1.755110	3.099713	4.800976	5.007029
33	O	4.583559	2.891965	3.736610	5.402487	5.464510
34	C	1.977335	1.902636	3.159006	4.835594	5.526887
35	O	2.904363	2.852306	4.134225	5.861218	6.547656
		6	7	8	9	10
6	H	0.000000				
7	H	1.779264	0.000000			
8	H	6.298020	6.249744	0.000000		
9	C	5.283604	5.341768	1.078375	0.000000	
10	C	4.753631	4.600131	2.219865	1.405065	0.000000
11	H	5.130501	5.760399	2.688405	2.238391	3.335276
12	H	5.436740	5.004305	2.679340	2.220345	1.079611
13	C	3.473210	3.640560	3.334781	2.290389	1.427176
14	H	3.115385	3.190763	4.344142	3.332562	2.239521
15	C	3.344205	4.016593	3.330652	2.290895	2.296650
16	H	2.815708	3.935568	4.340063	3.334702	3.340822
17	C	4.564635	5.042388	2.235943	1.429176	2.295589
18	H	7.764216	7.009996	4.144186	4.645522	5.214290
19	C	7.214566	6.324184	4.610404	4.888607	5.273009
20	C	7.337472	6.488585	5.646214	5.813251	6.247453
21	H	6.533743	5.345155	4.392364	4.450535	4.233612
22	H	7.996795	7.303416	6.125303	6.369427	6.978901
23	C	6.751201	5.698901	6.296451	6.247297	6.412246
24	H	6.941438	5.918313	7.217031	7.094912	7.246501
25	C	6.233353	4.955687	5.812697	5.689335	5.584492
26	H	5.934650	4.458304	6.390408	6.127771	5.792007
27	C	6.532185	5.391166	4.729217	4.789406	4.784148
28	C	3.810422	3.041546	5.653665	5.106496	5.047500
29	O	4.260854	3.269885	6.793834	6.247159	6.069847
30	C	3.901061	2.854092	4.019562	3.413398	2.782144
31	O	4.297976	2.867198	4.970890	4.408533	3.562300
32	C	5.519582	5.245507	5.305035	5.105324	5.671095
33	O	6.045109	6.000407	6.074592	5.889027	6.570524
34	C	5.305587	5.057008	2.835391	2.753243	3.503404
35	O	6.229656	6.152699	2.654477	2.909738	4.006414
		11	12	13	14	15
11	H	0.000000				
12	H	4.346742	0.000000			
13	C	3.333697	2.238027	0.000000		
14	H	4.343015	2.695595	1.079146	0.000000	
15	C	2.222810	3.339692	1.417181	2.231640	0.000000
16	H	2.675625	4.350626	2.228841	2.687816	1.079551

17	C	1.078214	3.340735	2.290493	3.332961	1.406807
18	H	5.483019	5.282458	6.113160	6.871680	6.182758
19	C	5.673436	5.361901	5.987094	6.636159	6.096919
20	C	6.166070	6.474987	6.731488	7.342624	6.656936
21	H	5.932519	3.966952	5.000018	5.403340	5.614482
22	H	6.433262	7.286711	7.455762	8.137710	7.214836
23	C	6.661086	6.610269	6.681238	7.105544	6.722406
24	H	7.297562	7.504052	7.359354	7.718987	7.318042
25	C	6.520277	5.620769	5.900057	6.210370	6.218449
26	H	7.035143	5.767860	5.945716	6.067516	6.400505
27	C	5.903123	4.723868	5.409874	5.871029	5.794208
28	C	5.149843	5.556977	4.688792	4.938172	4.565552
29	O	6.327612	6.485505	5.652376	5.739849	5.624866
30	C	4.616277	2.954880	2.807592	3.011053	3.484484
31	O	5.746974	3.447255	3.565121	3.464640	4.449140
32	C	4.580507	6.310868	5.653678	6.288698	5.105924
33	O	4.968789	7.290754	6.464122	7.115468	5.724686
34	C	2.916553	4.125575	3.916835	4.781771	3.561437
35	O	2.738203	4.640556	4.577029	5.551780	4.055800
		16	17	18	19	20
16	H	0.000000				
17	C	2.217028	0.000000			
18	H	6.978076	5.332020	0.000000		
19	C	6.809518	5.450799	1.078772	0.000000	
20	C	7.196043	6.087746	2.222045	1.408938	0.000000
21	H	6.435091	5.316650	2.683078	2.225226	3.322529
22	H	7.698700	6.528809	2.679010	2.222898	1.079878
23	C	7.159723	6.445769	3.340565	2.297098	1.428392
24	H	7.623454	7.139235	4.350467	3.339975	2.243334
25	C	6.755966	6.084171	3.342158	2.300379	2.298577
26	H	6.870659	6.493414	4.350721	3.342553	3.340775
27	C	6.515103	5.438676	2.232482	1.417880	2.281739
28	C	4.691316	4.815875	4.888888	4.058773	3.762715
29	O	5.665133	5.982356	5.647310	4.695983	4.197970
30	C	4.125849	3.794650	4.455272	3.940089	4.585596
31	O	5.029512	4.885623	5.033743	4.415239	5.040214
32	C	5.307095	4.717936	3.934945	3.377077	2.728185
33	O	5.772182	5.309373	4.789818	4.325576	3.501758
34	C	4.209432	2.797749	2.883994	2.818443	3.302737
35	O	4.712592	2.949781	2.912491	3.214961	3.712996
		21	22	23	24	25
21	H	0.000000				
22	H	4.333944	0.000000			
23	C	3.324863	2.239016	0.000000		
24	H	4.333233	2.700747	1.078995	0.000000	
25	C	2.221986	3.341218	1.415432	2.225513	0.000000

26	H	2.682099	4.349768	2.225683	2.677697	1.078142
27	C	1.078617	3.326873	2.282688	3.325022	1.412408
28	C	4.127003	4.430091	3.011267	3.153367	2.888056
29	O	4.696150	4.856094	3.104587	2.905646	3.042336
30	C	2.690926	5.516326	4.216184	4.918723	3.209769
31	O	2.759895	6.055355	4.437935	5.078679	3.208461
32	C	4.645263	2.840201	2.866255	3.080443	3.568168
33	O	5.765088	3.288788	3.702344	3.676099	4.598748
34	C	3.472743	3.751762	3.841446	4.613108	3.767313
35	O	4.219601	3.894423	4.571976	5.343265	4.674818

		26	27	28	29	30
26	H	0.000000				
27	C	2.228158	0.000000			
28	C	2.907895	3.577020	0.000000		
29	O	2.762238	4.099025	1.194530	0.000000	
30	C	3.153866	2.973108	2.797850	3.577640	0.000000
31	O	2.815928	3.180334	3.275668	3.715890	1.148737
32	C	4.232920	3.818335	2.423955	3.192641	4.239245
33	O	5.239775	4.908373	3.259981	3.863071	5.304037
34	C	4.481332	3.145633	3.270159	4.403064	3.064962
35	O	5.496177	3.903709	4.343437	5.459801	4.148000

		31	32	33	34	35
31	O	0.000000				
32	C	5.038564	0.000000			
33	O	6.117962	1.140032	0.000000		
34	C	4.115906	2.485642	3.361432	0.000000	
35	O	5.200414	3.030678	3.641218	1.166818	0.000000

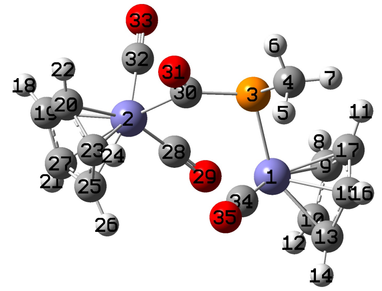

15S -3747.671553 33.30 C1
WBI 0.23

Charge and spin density

		1	2
1	Fe	0.167648	2.373174
2	Fe	-0.056848	0.045163
3	P	0.020894	-0.098737
4	C	0.002977	-0.004757
9	C	-0.022944	-0.015240

		1	2	3	4	5
1	Fe	0.000000				
2	Fe	3.983200	0.000000			
3	P	2.307217	3.313469	0.000000		
4	C	3.408729	4.753060	1.844471	0.000000	
5	H	3.512347	4.938985	2.481471	1.091981	0.000000
6	H	4.367118	4.945250	2.422054	1.091242	1.770322
7	H	3.634400	5.639704	2.438388	1.091686	1.778126
8	H	2.866477	5.591955	3.837681	5.077245	5.644782
9	C	2.115724	5.508979	3.529249	4.571672	4.976224
10	C	2.107159	5.649188	4.212523	5.227549	5.361136
11	H	2.846550	5.992100	2.955479	3.169872	3.769278
12	H	2.841788	5.828976	4.960276	6.150993	6.268423
13	C	2.098482	5.946330	4.230002	4.803556	4.688995
14	H	2.848140	6.392549	5.004773	5.466821	5.157828
15	C	2.067312	5.965178	3.526949	3.700475	3.669748
16	H	2.824141	6.452705	3.863382	3.511607	3.269877
17	C	2.101154	5.726729	3.050474	3.552067	3.931762
18	H	6.791819	2.863002	5.991200	7.326956	7.546597

10	C	-0.040622	0.036464
13	C	-0.028299	0.019336
15	C	-0.046603	0.050306
17	C	0.012396	0.006786
19	C	0.001495	-0.007603
20	C	0.047669	-0.000990
23	C	0.004266	-0.002251
25	C	0.007638	-0.000490
27	C	0.102318	0.009507
28	C	0.094583	-0.132050
29	O	-0.182039	-0.103186
30	C	0.170400	-0.085000
31	O	-0.172517	-0.081407
32	C	0.030919	-0.006057
33	O	-0.113333	-0.002967

19	C	6.030055	2.117235	5.364272	6.644825	6.751092
20	C	5.795167	2.102557	4.849937	5.805639	5.803372
21	H	5.777839	2.877381	5.956519	7.481633	7.529768
22	H	6.382537	2.836114	5.084824	5.814205	5.858807
23	C	5.026308	2.113991	4.495440	5.351586	5.133201
24	H	4.985885	2.840934	4.409074	4.906113	4.509845
25	C	4.756367	2.108438	4.805228	5.963833	5.776358
26	H	4.496756	2.862727	5.029790	6.133211	5.829077
27	C	5.446537	2.127265	5.344500	6.736051	6.742806
28	C	2.695210	1.711950	2.925898	4.624262	4.848379
29	O	2.153724	2.875382	3.207740	4.880561	5.098934
30	C	3.409855	2.036994	1.839451	2.824256	3.068109
31	O	4.428787	2.827498	2.687568	2.964267	3.071506
32	C	4.641087	1.759305	3.418931	4.947630	5.476861
33	O	5.369902	2.898679	3.971184	5.428414	6.115553
34	C	1.752027	3.821119	2.835430	3.415638	3.006598
35	O	2.895111	4.200974	3.623943	3.840033	3.146236
		6	7	8	9	10
6	H	0.000000				
7	H	1.771112	0.000000			
8	H	5.871964	4.849222	0.000000		
9	C	5.492336	4.333689	1.079042	0.000000	
10	C	6.232664	5.124426	2.241324	1.427046	0.000000
11	H	4.008958	2.522162	2.672439	2.218006	3.331953
12	H	7.125099	6.132280	2.696932	2.236944	1.079857
13	C	5.879802	4.679657	3.339696	2.298082	1.404340
14	H	6.543221	5.420887	4.350322	3.342263	2.219423
15	C	4.781688	3.385062	3.337182	2.294646	2.290352
16	H	4.572195	3.124381	4.345365	3.336628	3.333109
17	C	4.524981	3.122434	2.222194	1.408435	2.288300
18	H	7.247020	8.256175	7.984895	8.111611	8.249991
19	C	6.650456	7.605556	7.533866	7.530202	7.565335
20	C	5.711404	6.842094	7.659181	7.513695	7.592090
21	H	7.784873	8.313974	7.068974	7.072328	6.791677
22	H	5.519510	6.867334	8.197421	8.072026	8.285619
23	C	5.468439	6.381291	7.263082	6.939117	6.815387
24	H	5.017504	5.953835	7.469195	7.017250	6.878281
25	C	6.279381	6.894160	6.841949	6.549664	6.231330
26	H	6.606701	6.989750	6.717197	6.310677	5.772025
27	C	6.963970	7.639654	7.035615	6.957251	6.760131
28	C	5.144226	5.279822	4.114469	4.012711	4.038849
29	O	5.580606	5.338742	3.279028	3.136057	2.993155
30	C	2.926496	3.802891	5.312013	5.051393	5.445166
31	O	2.699434	4.031491	6.404851	6.107083	6.514665
32	C	4.932833	5.702892	5.438633	5.650740	6.171611
33	O	5.292428	6.069852	5.668580	6.053740	6.763445

34	C	4.303578	3.957507	4.616968	3.816608	3.453745
35	O	4.607653	4.490151	5.759978	4.936372	4.480657
		11	12	13	14	15
11	H	0.000000				
12	H	4.339858	0.000000			
13	C	3.343400	2.215033	0.000000		
14	H	4.354240	2.670782	1.079467	0.000000	
15	C	2.230374	3.333758	1.430459	2.242288	0.000000
16	H	2.688678	4.343279	2.243921	2.703343	1.079030
17	C	1.079569	3.330341	2.299054	3.342697	1.419247
18	H	8.684806	8.225860	8.696371	9.085023	8.809377
19	C	8.100496	7.577507	7.891004	8.212909	8.025826
20	C	7.745237	7.782876	7.689729	7.991658	7.639805
21	H	8.220607	6.504248	7.282280	7.473384	7.802807
22	H	8.037709	8.570695	8.337085	8.692598	8.122809
23	C	7.252543	7.017762	6.738031	6.904420	6.780528
24	H	7.099067	7.202308	6.586374	6.693251	6.508962
25	C	7.293096	6.232766	6.304663	6.402336	6.632531
26	H	7.219698	5.707434	5.750616	5.698066	6.250375
27	C	7.841986	6.639195	7.098933	7.309666	7.458743
28	C	4.959136	4.140155	4.528471	5.036064	4.761799
29	O	4.509451	2.992452	3.672666	4.213195	4.127063
30	C	4.786413	5.985854	5.427595	5.978588	4.983531
31	O	5.542090	7.107961	6.344745	6.832598	5.770425
32	C	5.930614	6.400238	6.696335	7.356626	6.523231
33	O	6.207773	7.004733	7.396957	8.150608	7.133204
34	C	4.141352	4.028130	2.861487	3.047402	2.872955
35	O	5.100092	4.994434	3.709368	3.625714	3.716205
		16	17	18	19	20
16	H	0.000000				
17	C	2.232957	0.000000			
18	H	9.315456	8.482619	0.000000		
19	C	8.484199	7.834258	1.079865	0.000000	
20	C	7.930919	7.560147	2.235363	1.425170	0.000000
21	H	8.432114	7.700249	2.678922	2.224216	3.342322
22	H	8.331020	7.987259	2.698091	2.241063	1.078292
23	C	7.013651	6.932921	3.336805	2.294176	1.414509
24	H	6.580926	6.811191	4.344199	3.335610	2.223091
25	C	7.028984	6.800787	3.332551	2.287570	2.287842
26	H	6.645682	6.595618	4.342900	3.330911	3.330390
27	C	7.984746	7.397484	2.220546	1.408705	2.298297
28	C	5.450213	4.488079	4.222595	3.556217	3.756895
29	O	4.962513	3.858174	5.278060	4.641307	4.893443
30	C	5.225722	4.767084	4.522641	3.822445	3.079511
31	O	5.821226	5.645396	4.794279	4.124616	3.040998
32	C	7.080185	5.907024	2.993778	2.854825	3.054846

33	O	7.714673	6.328003	3.535921	3.692801	3.965129
34	C	3.097311	3.516025	6.591547	5.653304	5.147695
35	O	3.669918	4.536238	6.785069	5.769790	5.088262
		21	22	23	24	25
21	H	0.000000				
22	H	4.351804	0.000000			
23	C	3.343877	2.223400	0.000000		
24	H	4.353235	2.672654	1.078011	0.000000	
25	C	2.241049	3.328287	1.412628	2.225811	0.000000
26	H	2.699435	4.336199	2.223381	2.679778	1.079639
27	C	1.079005	3.340549	2.301622	3.344162	1.427691
28	C	3.262044	4.531183	3.360055	3.885014	2.814906
29	O	3.941624	5.683657	4.359339	4.779121	3.656327
30	C	4.871069	3.270362	2.853140	2.839291	3.494692
31	O	5.557880	2.872806	2.952694	2.678635	3.991205
32	C	3.872913	3.342455	3.651861	4.316938	3.800338
33	O	4.734232	4.053145	4.728246	5.370101	4.913423
34	C	5.560988	5.744122	4.073570	3.765018	3.965737
35	O	5.797623	5.650096	3.848870	3.304886	3.899048
		26	27	28	29	30
26	H	0.000000				
27	C	2.239488	0.000000			
28	C	2.993547	2.977047	0.000000		
29	O	3.529459	3.880438	1.164848	0.000000	
30	C	4.004297	4.042237	2.650783	3.505331	0.000000
31	O	4.589026	4.602357	3.788817	4.683569	1.201294
32	C	4.584872	3.364033	2.568879	3.546684	2.555628
33	O	5.708280	4.340012	3.513500	4.334466	3.400223
34	C	3.571973	5.038645	3.058405	2.964841	3.150868
35	O	3.434867	5.174610	3.805254	3.896613	3.515701
		31	32	33	34	35
31	O	0.000000				
32	C	3.257942	0.000000			
33	O	3.976625	1.140023	0.000000		
34	C	3.889687	4.995240	5.937715	0.000000	
35	O	3.963158	5.575057	6.586021	1.145149	0.000000

Table S6. Orbital energies and the HOMO-LUMO gaps.

Structure	HOMO energy (Hartree)	LUMO energy (Hartree)	HOMO/LUMO Gap (Hartree)	HOMO/LUMO Gap (eV)
Fe2PCO0-1T	-0.20424	-0.03020	0.17404	4.74
Fe2PCO0-2Q	-0.19653	-0.08013	0.11640	3.17
Fe2PCO0-3Q	-0.19709	-0.08096	0.11613	3.16
Fe2PCO0-4Q	-0.20559	-0.05365	0.15194	4.13
Fe2PCO0-5Q	-0.19871	-0.06527	0.13344	3.63
Fe2PCO0-6T	-0.20147	-0.08539	0.11608	3.16
Fe2PCO0-7S	-0.20429	-0.06417	0.14012	3.81
Fe2PCO1-1Q	-0.20875	-0.07767	0.13108	3.57
Fe2PCO1-2Q	-0.21084	-0.08042	0.13042	3.55
Fe2PCO1-3T	-0.20534	-0.07643	0.12891	3.51
Fe2PCO1-4Q	-0.19991	-0.06956	0.13035	3.55
Fe2PCO1-5Q	-0.20152	-0.06209	0.13943	3.79
Fe2PCO1-6Q	-0.20335	-0.07630	0.12705	3.46
Fe2PCO1-7T	-0.20726	-0.09242	0.11484	3.12
Fe2PCO1-8T	-0.18775	-0.05688	0.13087	3.56
Fe2PCO1-9S	-0.20541	-0.05005	0.15536	4.23
Fe2PCO1-10Q	-0.19833	-0.08499	0.11334	3.08
Fe2PCO1-11T	-0.19574	-0.08117	0.11457	3.12
Fe2PCO1-12T	-0.19776	-0.05487	0.14289	3.89
Fe2PCO1-13T	-0.20696	-0.05544	0.15152	4.12
Fe2PCO1-14T	-0.20195	-0.06836	0.13359	3.64
Fe2PCO1-15T	-0.20066	-0.07036	0.13030	3.55
Fe2PCO1-16T	-0.20436	-0.07867	0.12569	3.42
Fe2PCO1-17S	-0.19926	-0.06875	0.13051	3.55
Fe2PCO2-1T	-0.21167	-0.06506	0.14661	3.99
Fe2PCO2-2Q	-0.19218	-0.04735	0.14483	3.94
Fe2PCO2-3Q	-0.18153	-0.03012	0.15141	4.12
Fe2PCO2-4T	-0.20694	-0.06551	0.14143	3.85
Fe2PCO2-5Q	-0.18603	-0.03239	0.15364	4.18
Fe2PCO2-6Q	-0.19359	-0.05921	0.13438	3.66
Fe2PCO2-7Q	-0.19104	-0.05833	0.13271	3.61
Fe2PCO2-8T	-0.20321	-0.06871	0.13450	3.66
Fe2PCO2-9Q	-0.19265	-0.06077	0.13188	3.59
Fe2PCO2-10T	-0.20200	-0.07049	0.13151	3.58
Fe2PCO2-11T	-0.20141	-0.06548	0.13593	3.70
Fe2PCO2-12S	-0.20492	-0.05313	0.15179	4.13

Fe2PCO2-13T	-0.20149	-0.07375	0.12774	3.48
Fe2PCO2-14T	-0.20339	-0.05441	0.14898	4.05
Fe2PCO2-15T	-0.20630	-0.05469	0.15161	4.13
Fe2PCO2-16S	-0.20276	-0.05102	0.15174	4.13
Fe2PCO2-17S	-0.19881	-0.07876	0.12005	3.27
Fe2PCO3-1S	-0.20362	-0.05828	0.14534	3.95
Fe2PCO3-2S	-0.20261	-0.05812	0.14449	3.93
Fe2PCO3-3S	-0.20038	-0.05994	0.14044	3.82
Fe2PCO3-4T	-0.18169	-0.05166	0.13003	3.54
Fe2PCO3-5T	-0.18066	-0.04760	0.13306	3.62
Fe2PCO3-6T	-0.17744	-0.04523	0.13221	3.60
Fe2PCO3-7T	-0.18274	-0.04501	0.13773	3.75
Fe2PCO3-8Q	-0.17258	-0.04118	0.13140	3.58
Fe2PCO3-9T	-0.18211	-0.04424	0.13787	3.75
Fe2PCO3-10Q	-0.17362	-0.04573	0.12789	3.48
Fe2PCO3-11S	-0.19793	-0.03949	0.15844	4.31
Fe2PCO3-12Q	-0.20061	-0.02697	0.17364	4.72
Fe2PCO3-13Q	-0.20066	-0.02997	0.17069	4.64
Fe2PCO3-14S	-0.19497	-0.03994	0.15503	4.22
Fe2PCO3-15T	-0.19431	-0.06754	0.12677	3.45
Fe2PCO3-16T	-0.21111	-0.08023	0.13088	3.56
Fe2PCO3-17T	-0.20183	-0.06899	0.13284	3.61
Fe2PCO3-18S	-0.20420	-0.08697	0.11723	3.19
Fe2PCO3-19T	-0.19538	-0.06389	0.13149	3.58
Fe2PCO4-1S	-0.18052	-0.03716	0.14336	3.90
Fe2PCO4-2S	-0.18191	-0.03850	0.14341	3.90
Fe2PCO4-3S	-0.18227	-0.05234	0.12993	3.54
Fe2PCO4-4T	-0.17997	-0.06147	0.11850	3.22
Fe2PCO4-5T	-0.19475	-0.04669	0.14806	4.03
Fe2PCO4-6T	-0.19682	-0.06537	0.13145	3.58
Fe2PCO4-7S	-0.19923	-0.06708	0.13215	3.60
Fe2PCO4-8Q	-0.18638	-0.05204	0.13434	3.66
Fe2PCO4-9S	-0.20589	-0.06789	0.13800	3.76
Fe2PCO4-10S	-0.20458	-0.05948	0.14510	3.95
Fe2PCO4-11S	-0.20271	-0.07130	0.13141	3.58
Fe2PCO4-12S	-0.20757	-0.07606	0.13151	3.58
Fe2PCO4-13S	-0.20567	-0.05994	0.14573	3.97
Fe2PCO4-14S	-0.20045	-0.07219	0.12826	3.49
Fe2PCO4-15S	-0.19176	-0.05876	0.13300	3.62
